# Non-Random Association of Ultraconserved Genomic Elements (UCE) with Human Genes

**DOI:** 10.3390/ijms27167214

**Published:** 2026-08-13

**Authors:** Larisa Fedorova, Yuriy L. Orlov, Oleh A. Mulyar, Alexei Fedorov

**Affiliations:** 1 CRI Genetics LLC, Santa Monica, CA 90404, USA; lvfedorova3@gmail.com (L.F.); amulyar@crigenetics.com (O.A.M.); 2 Center of Digital Health and AI in medicine, I.M. Sechenov First Moscow State Medical University of the Ministry of Health of the Russian Federation (Sechenov University), 119991 Moscow, Russia; y.orlov@sechenov.ru; 3 Agrarian and Technological Institute, Peoples’ Friendship University of Russia (RUDN University), 117198 Moscow, Russia

**Keywords:** bioinformatics, computational biology, genetic variation, evolution, DNA conformation, ultra-conserved

## Abstract

Ultraconserved elements (UCEs) are among the most evolutionarily conserved DNA sequences in vertebrate genomes, yet the biological mechanisms underlying their extraordinary conservation remain poorly understood. Using the recently developed dedUCE database comprising 12,813 human UCEs, we performed a comprehensive genome-wide analysis of their distribution relative to protein-coding genes, transcription factor (TF) genes, and long noncoding RNA (lncRNA) genes. UCEs showed a highly non-random genomic organization, with approximately 40% occurring in clusters within 20 kb genomic intervals. Non-KRAB transcription factor genes exhibited a striking sevenfold enrichment of UCEs compared with random expectation, whereas KRAB zinc-finger genes displayed an approximately tenfold depletion. Beyond TFs, UCE-rich genes were predominantly involved in developmental regulation, chromatin remodeling, RNA processing, and embryonic neurogenesis, whereas similarly large UCE-poor genes primarily encoded membrane proteins, ion channels, and synaptic components required for mature neuronal function. UCEs also demonstrated strong positional bias, with approximately fourfold enrichment near the 3′ ends of protein-coding genes but no comparable distribution pattern in lncRNAs. Although lncRNA genes showed only modest overall UCE enrichment, a small subset contained numerous UCEs. These findings demonstrate that UCEs preferentially associate with master developmental regulators rather than downstream neuronal effector genes, providing new insights into the functional organization and evolutionary conservation of the human genome.

## 1. Introduction

Ultraconserved noncoding elements (UCNEs) are widespread throughout the genomes of mammals and other vertebrates [1,2]. Thousands of these highly conserved genomic regions, typically longer than 200 bp, are located within intronic and intergenic sequences. Remarkably, many evolutionarily distant vertebrate species share virtually identical UCNE sequences despite having been exposed to the same mutation rate as the remainder of the genome [1,2,3,4,5]. In most cases, individual UCNEs show no sequence similarity to other UCNEs within the same genome.

Despite two decades of investigation, the reason why these long noncoding DNA fragments have remained unchanged for hundreds of millions of years remains unresolved. Theodosius Dobzhansky’s famous statement, “Nothing in Biology Makes Sense Except in the Light of Evolution” [6], appears paradoxically difficult to apply to UCNEs because their biological functions remain elusive. Several population-genetic studies have suggested that UCNEs are subject to exceptionally strong purifying selection [3,7,8,9,10]. However, this interpretation appears inconsistent with the observation that nearly all mutations identified within UCNE sequences have no obvious phenotypic consequences [3,11] and are absent from the ClinVar database [12].

Numerous hypotheses have been proposed to explain the functional significance of UCNEs. Some studies suggest that UCNEs serve as sources of noncoding RNAs [13,14], whereas others emphasize their roles as long-range transcriptional enhancers [4]. However, previous analyses demonstrated that fewer than 20% of UCNEs overlap known noncoding RNA transcripts and that the overall extent of overlap is close to random expectation [5]. In a recent comprehensive review, Snetkova et al. [4] critically evaluated the evidence supporting enhancer functions of UCNEs and concluded that the uninterrupted conservation of DNA segments extending over hundreds of nucleotides remains difficult to explain. The authors proposed that ultraconservation is likely maintained by multiple evolutionary and functional mechanisms rather than by a single process. Additional hypotheses suggest that ultraconserved elements contribute to higher-order chromatin organization and three-dimensional genome architecture [15]. Nevertheless, no specific three-dimensional DNA structure or protein interaction has yet been identified that adequately explains their role in genome organization.

The relationship between UCNE sequence variation and biological function remains particularly enigmatic. Although approximately thirty thousand polymorphisms have been identified within human UCNEs, only a small number have been associated with disease phenotypes or measurable biological traits [3,16]. Likewise, Snetkova and colleagues noted that “there has been no direct demonstration that loss of any ultraconserved enhancer results in reduced viability, fertility, or fecundity” [4]. As emphasized by Habic et al. [3], “the biological functions of most ultraconserved regions remain poorly understood, and their contributions to human disease are largely unknown”. Although strong purifying selection is widely invoked to explain the long-term preservation of UCNE sequences [3,7,12], the molecular mechanisms responsible for this extraordinary conservation remain unclear. The scarcity of pathogenic variants within UCNEs suggests that additional factors may contribute to the maintenance of ultraconserved DNA. Understanding these mechanisms may provide important insights into genome stability, mutational processes, and the biological systems that limit the accumulation of deleterious genetic variation.

Progress in this field has been constrained, in part, by the limited scope of earlier UCNE databases. Until recently, available resources captured only a subset of ultraconserved sequences present in vertebrate genomes. In 2025, Cummins and colleagues introduced dedUCE, a comprehensive database of ultraconserved vertebrate elements that includes both noncoding and protein-coding ultraconserved sequences [10]. By incorporating elements as short as 100 bp, dedUCE contains more than three times as many ultraconserved elements as UCNEbase. In addition, the database provides detailed evolutionary information for each element across hundreds of mammalian species as well as numerous non-mammalian vertebrates.

**Figure 1:**
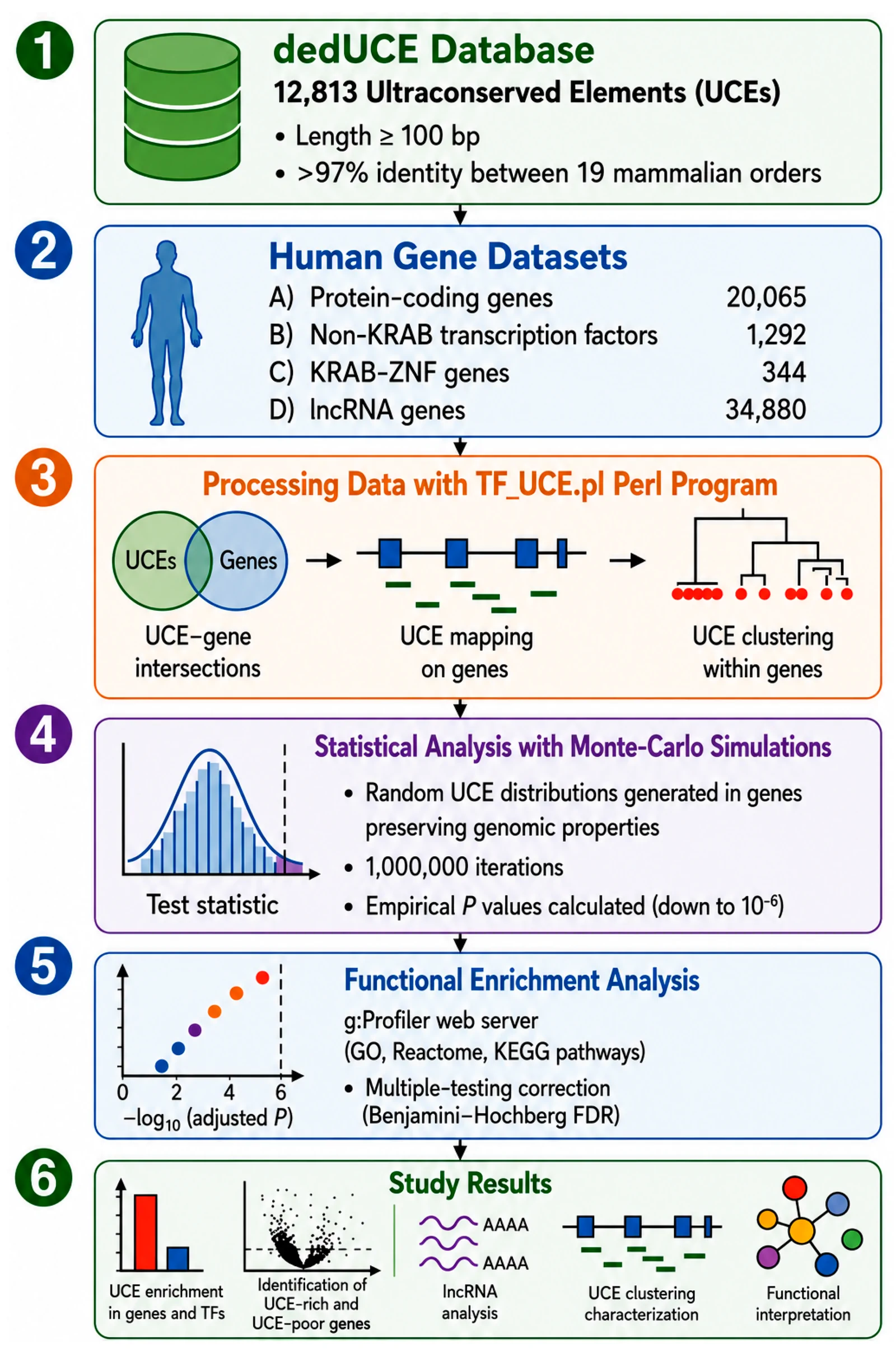
Overview of the computational workflow used for genome-wide analysis of ultraconserved elements in human genes.

In the present study, we used the dedUCE database [10] to perform a comprehensive bioinformatic analysis of the relationships between ultraconserved elements (UCEs), protein-coding genes, and noncoding RNA genes in the human genome. Our primary objective was to identify non-random patterns of UCE distribution that may provide insights into their biological functions and evolutionary significance. By examining the genomic locations of 12,813 ultraconserved elements, we sought to identify classes of genes preferentially associated with UCEs and to gain further insight into the mechanisms responsible for the remarkable evolutionary preservation of these sequences. The workflow of our research is presented in Figure 1.

## 2. Results and Discussion

### 2.1. Databases of Ultraconserved Elements: Differences Between dedUCE and UCNEbase

In our previous studies of ultraconserved elements, we used the public UCNEbase database, which was established in 2013 and contains 4272 human noncoding ultraconserved genomic regions longer than 200 bp [17]. Recently, a new public UCE resource, dedUCE by Cummins et al., 2025 [10], became available. By shortening the minimum length threshold to 100 bp, dedUCE substantially expands the catalog of known ultraconserved elements and provides a more comprehensive representation of ultraconserved sequences in vertebrate genomes.

A major advantage of dedUCE is the extensive phylogenetic analysis used to define ultraconservation. Each element was evaluated across hundreds of mammalian and other vertebrate species, providing a robust measure of evolutionary conservation. In contrast to UCNEbase, which is restricted to noncoding regions, dedUCE also includes protein-coding sequences that meet stringent ultraconservation criteria. Protein-coding sequences constitute approximately 3% of all entries in the dedUCE. In the present study, we analyzed the subset of dedUCE sequences satisfying the following criteria: (i) sequence identity exceeding 97% in at least 50% of species representing 19 placental mammalian orders and (ii) sequence length greater than 100 bp. This dataset contains 12,813 human UCEs, more than three times the number represented in UCNEbase.

Previous work demonstrated that ultraconserved sequences possess a distinctive dinucleotide composition that differs markedly from that of the genome as a whole Fedorova et al., 2023 [5]. To compare the sequence characteristics of dedUCE and UCNEbase, we calculated genomic signatures (***ρ***) for nucleotides ***x*** and ***y*** (representing A, G, C, T) calculated as following: ***ρ_xy_*** = F***_xy_***/(F***_x_*** × F***_y_***), where F***_x_*** and F***_y_*** denote the frequencies of individual nucleotides and F***_xy_*** represents the frequency of dinucleotide ***xy***. Values of ***ρ*** > 1 indicate overrepresentation, whereas values of ***ρ*** < 1 indicate underrepresentation of dinucleotide over random expectation.

As shown in Table 1, both databases display a characteristic excess of GpC dinucleotides together with the depletion of GG and CC dinucleotides relative to genomic background frequencies. However, these compositional biases are more pronounced in UCNEbase than in dedUCE. One possible explanation is the inclusion of protein-coding sequences in dedUCE, which are subject to additional constraints imposed by codon bias and protein amino acid composition. Consequently, the dinucleotide profile of dedUCE may be viewed as a partially diluted version of the UCNEbase signature, reflecting the contribution of a broader spectrum of genomic sequences while retaining the distinctive compositional properties associated with ultraconservation.

### 2.2. Uneven Chromosomal Distribution of UCEs and UCE Clusters

**Figure 2:**
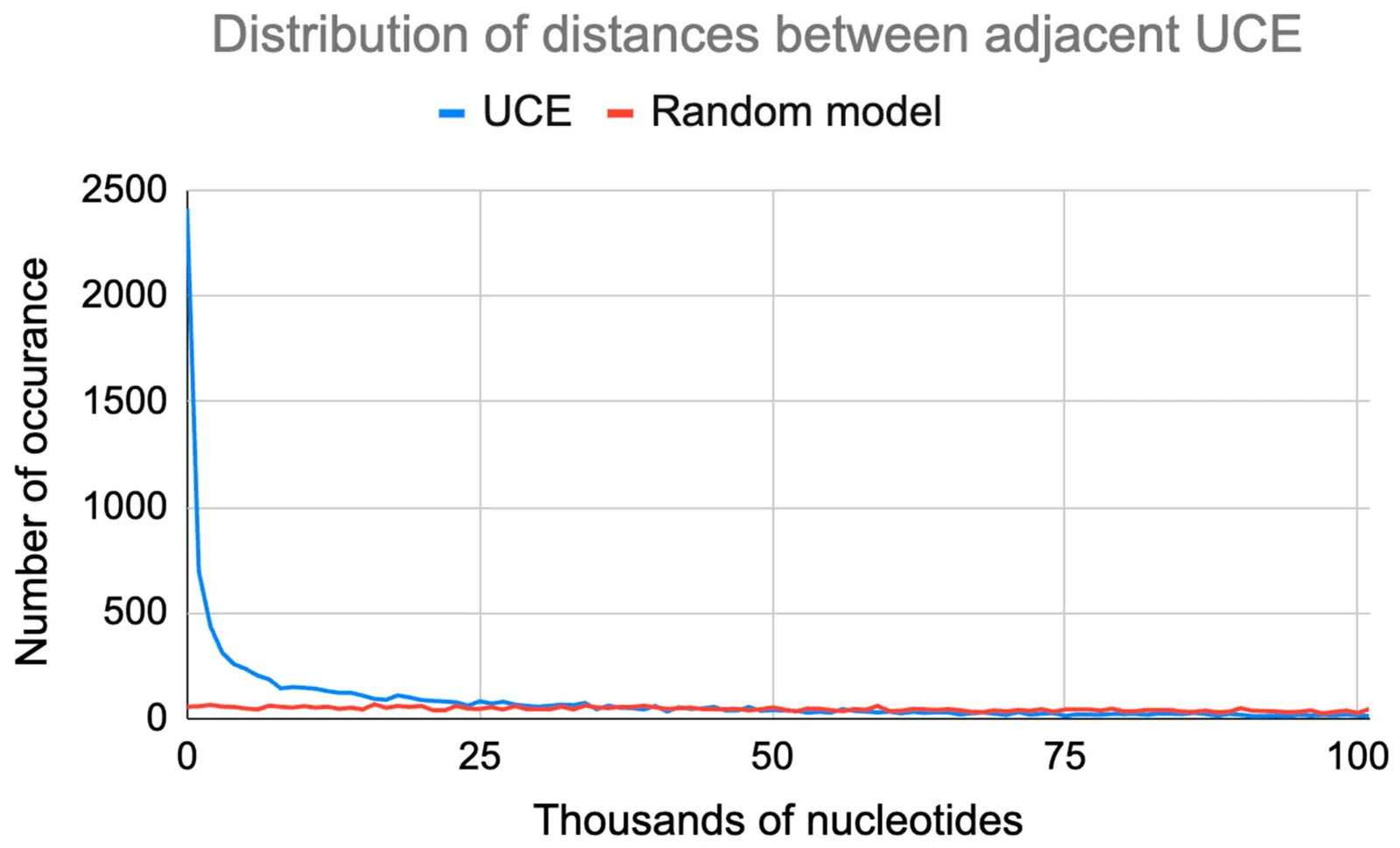
Distribution of distances between adjacent UCEs and comparison with a computer model in which 12,813 UCEs are randomly distributed throughout the genome.

UCEs are not randomly distributed throughout the genome; instead, they show a strong tendency to cluster within specific genomic regions, as previously described [4,10,18]. This pattern is illustrated in Figure 2, which shows the distribution of distances between adjacent UCEs (nearest neighbors) and compares it with a computer model in which 12,813 UCEs are randomly distributed throughout the human genome. On average, a UCE occurs approximately every 230 kb in the human genome. However, the observed distribution shows that many UCEs occur in clusters, with several elements located within 20 kb of one another. Overall, approximately 5000 UCEs occur closer than 20 kb to another UCE, above random expectation, comprising 39% of these elements.

**Figure 3:**
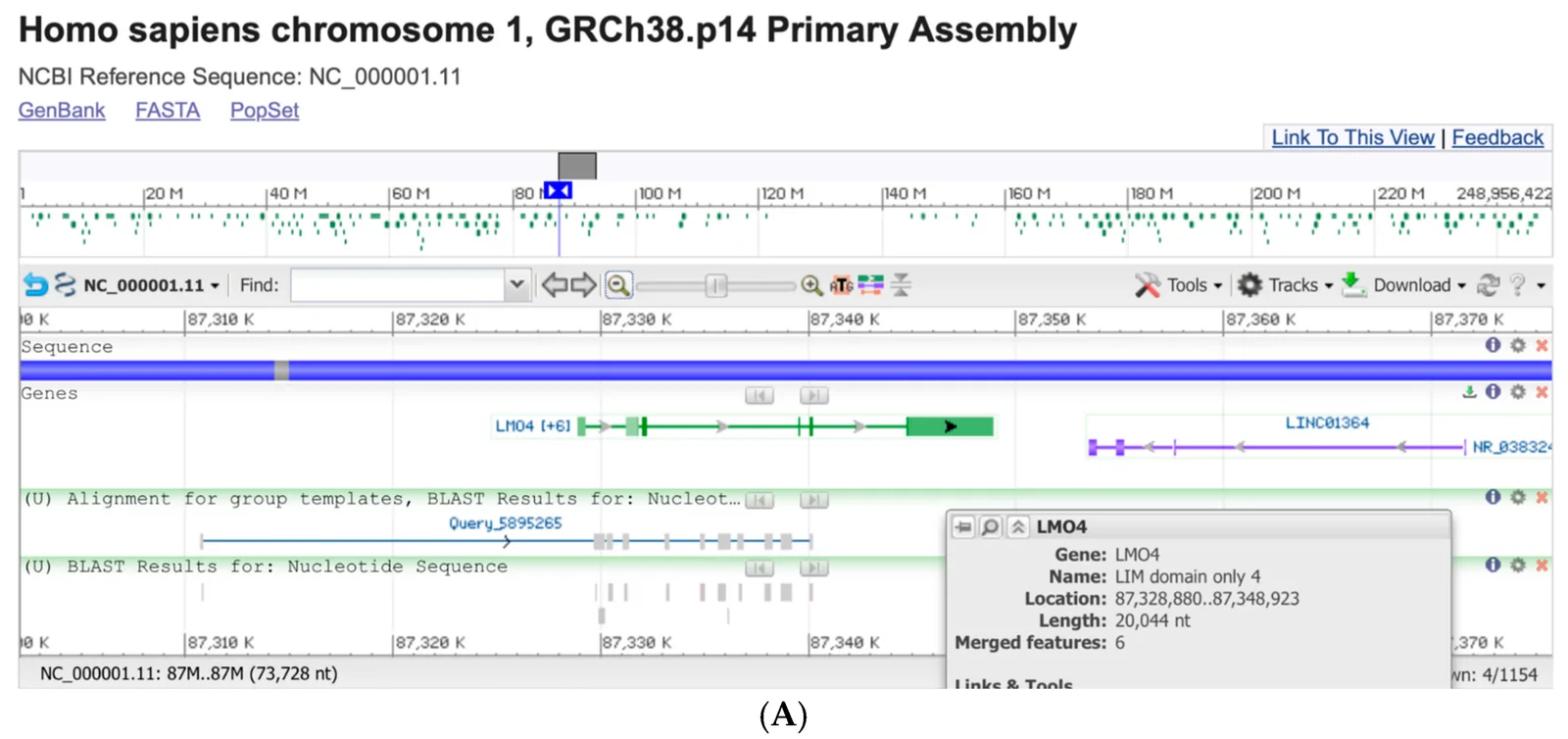

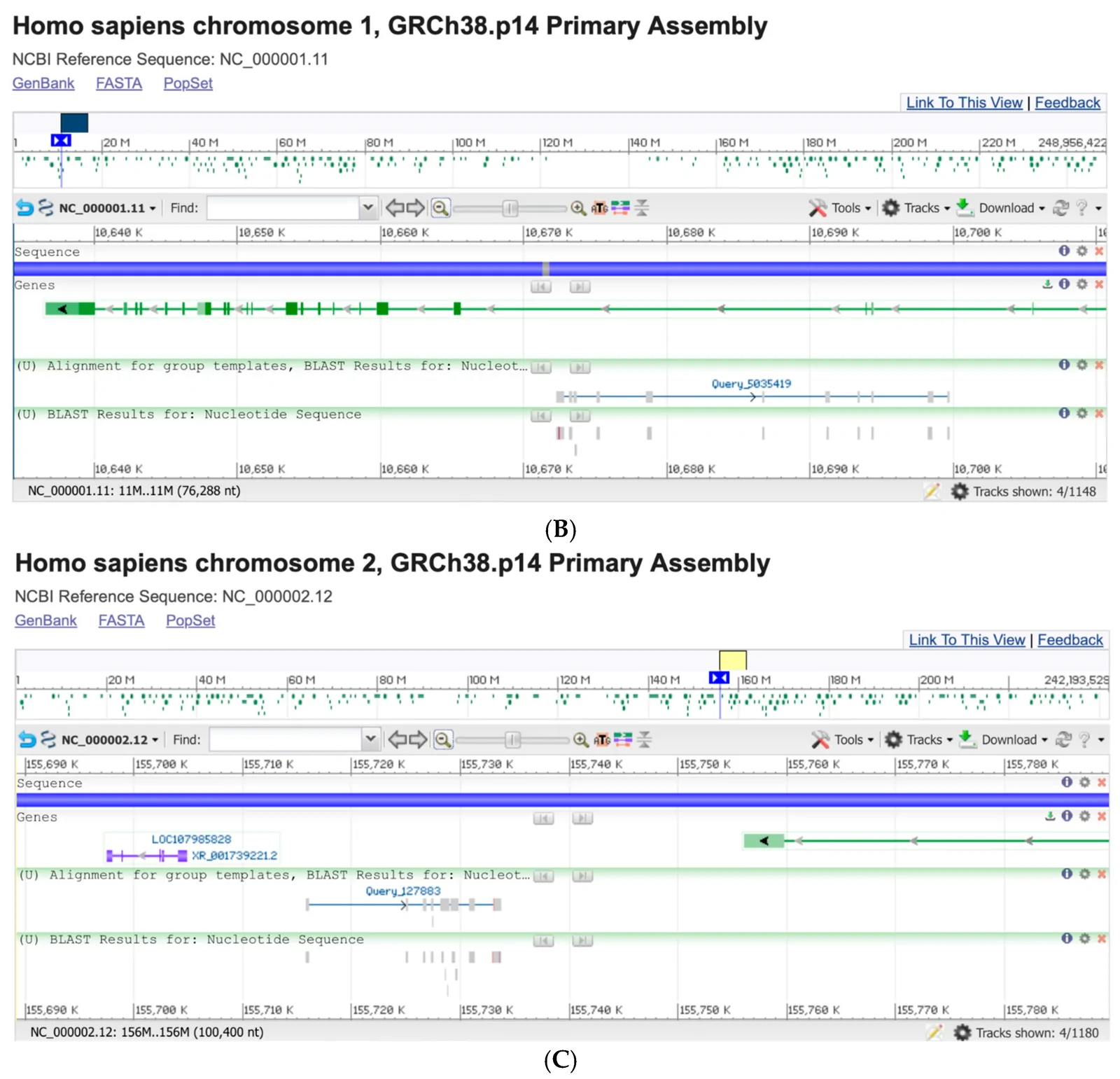
(**A**) Visualization of UCE cluster #4 inside the 5′ end of the LMO4 gene (Query track); (**B**) UCE cluster #1 within the middle part of the CASZ1 gene; (**C**) cluster #16 inside an intergenic region. Query UCE clusters are shown as gray rectangles beneath the genes. They are concatenated with each other by a gray line.

We identified the most prominent UCE clusters in dedUCE, defined as genomic regions containing more than 10 UCEs within a span of no more than 25 kb. This cutoff for UCE clustering was selected after evaluating several parameter values in order to obtain approximately 50–100 top UCE clusters, a number that is feasible for detailed manual analysis. Then we computationally scanned the human genome and identified 52 clusters meeting these conditions. For each cluster, we examined the positions of UCEs relative to one another and to nearby genomic features, including exons, introns, and ncRNAs, using NCBI BLAST (version 2.17.0+) and the Gene Viewer online tools (Sequence Viewer version 3.51) [19]. For this manual analysis, all UCE sequences within a cluster were concatenated and used as a BLAST query against the human genome. A representative example of this analysis is shown in Figure 3, and the complete set of results is provided in Supplementary File S1.

The details of these 52 UCE clusters and their associated genes are summarized in Table 2. Of the 52 clusters, 48 are located within protein-coding genes, two within lincRNAs, and two within intergenic regions. In six cases, two or three clusters occur within the same gene; consequently, 37 protein-coding genes contain the 48 analyzed clusters. The average length of these genes is 217 kb. Among these 37 genes, 31 encode DNA-binding proteins, primarily transcription factors (TF); one encodes an RNA-binding protein (MBNL1); and five encode proteins not known to interact directly with nucleic acids. Overall, genes containing UCE clusters predominantly encode TF or other DNA-associated proteins (84%). Monte Carlo analysis demonstrated that the localization of these 52 clusters within protein-coding genes is highly non-random (*p* < 10^−6^). We also observed a strong tendency for clustered UCEs to localize within terminal 3′ exons. (Table 2, column 7).

### 2.3. Association of UCEs with Transcription Factor Genes

We further examined the association between UCE and TF genes by analyzing a complete set of well-defined TF genes together with all dedUCE sequences. Specifically, we examined 1292 TF genes from the HumanTFDB/Lambert catalog after excluding entries classified as KRAB-containing C2H2 zinc-finger proteins. KRAB proteins represent a distinct biological system rather than general transcriptional regulators: they frequently bind repetitive DNA and mediate strong transcriptional repression associated with genome defense functions.

Among the 1292 non-KRAB TF genes, one-third (440 genes) contain at least one UCE, whereas two-thirds (852 genes) contain no UCEs (Supplementary Table S1). In total, 2595 UCEs are located within non-KRAB TF genes, whose combined genomic length is 88,646,446 nucleotides. Among UCE-containing TF genes, 271 harbor two or more UCEs. The largest number of UCEs was observed in NPAS3, a member of the bHLH transcription factor family, which contains 81 UCEs. Given that the human genome (GRCh38.p13) comprises approximately 2949 Mb, a random distribution model predicts approximately 385 UCEs within an 88.6 Mb genomic region representing the total length of all non-KRAB TF genes. Thus, UCEs are enriched approximately 7-fold within non-KRAB TF genes relative to random expectation. Monte Carlo analysis demonstrated that this enrichment is highly significant (under a random model, the number of UCEs counts more than 500 within non-KRAB TFs gives a *p* value < 10^−6^). TF genes are generally long, with an average length of approximately 69 kb, and belong to multiple families. Among them, the principal homeobox cluster genes (HOXA, HOXB, HOXC, and HOXD) are among the shortest. These 39 HOXA through HOXD genes show particularly strong UCE enrichment when their small average length (11.4 kb) and the total of 124 UCEs within them are considered.

We also examined all 344 KRAB-ZNF (Krüppel-associated box zinc-finger) genes. In striking contrast to the non-KRAB TF genes, only a single UCE was identified within KRAB-ZNF genes, whose combined genomic length is 11,612,873 nucleotides (Supplementary Table S2). Under a random distribution model, approximately 50 UCEs would be expected within an 11.6 Mb genomic region. Thus, despite sharing the C2H2 zinc-finger DNA-binding domain that characterizes the largest TF family, KRAB-ZNF genes exhibit a marked depletion of UCEs. Because we counted only one UCE, the regular statistics are problematic. Yet, Monte Carlo analysis indicated that this depletion is highly significant. The depletion of UCE in KRAB genes is 10-fold less than random expectation with *p* value confidence interval 0.05 and 5-fold less than random expectation with *p* value 0.0005.

### 2.4. Association of UCEs with the Entire Set of Protein-Coding Genes

We next analyzed a representative set of 20,066 human protein-coding genes with a combined genomic length of 1408 Mb. The association of UCEs with each gene is presented in Supplementary Table S3, in which genes are ranked according to the number of UCEs they contain. A total of 8562 UCEs were located within protein-coding genes, whereas random expectation was approximately 5800 UCEs.

When the 1292 non-KRAB TF genes were excluded from the analysis, the number of UCEs within genes decreased to 5967, whereas the random expectation was approximately 5420 UCEs. Thus, non-TF protein-coding genes exhibit only modest enrichment (~10%) for UCEs. Nevertheless, several non-TF genes contain substantial numbers of UCEs. The highest number was observed in LRMDA, a 1.1 Mb gene involved in melanocyte differentiation, which contains 86 UCEs. We therefore examined the 100 non-TF genes with the largest numbers of UCEs; their functional characteristics are summarized in Table 3.

The data in Table 3 are consistent with the conclusion of Cummins and co-authors [10] that UCEs are preferentially associated with genes involved in brain function and development. However, these genes are typically very large and contain multiple exceptionally long introns. Consequently, the apparent enrichment of UCEs within this subset may be influenced, at least in part, by their extraordinary genomic length. The average size of our set of the 100 UCE-rich non-TF genes is 482 kb. Notably, the transcription of a 480 kb gene by RNA polymerase II requires more than three hours. In contrast, genes requiring rapid and high-level expression, such as globins, ribosomal proteins, and heat-shock genes, are generally much shorter and therefore cannot physically accommodate large numbers of UCEs.

Finally, we analyzed the 100 longest non-TF genes that do not contain any UCEs (UCE-poor set). The complete list is provided in Supplementary Table S4, where genes are ranked by genomic length. The average size of these genes is 819 kb. Functional analysis revealed strong enrichment for nervous-system-related processes (Table 4). The 100 genes are strongly enriched for proteins involved in the organization and function of the nervous system, particularly those controlling neuronal connectivity, synapse formation, axon guidance, and neuronal signaling. This is by far the dominant feature of the dataset. Despite their exceptional size, none of these genes contains a UCE. Thus, some brain-specific genes contain UCE clusters (Table 3), whereas other brain-specific genes avoid UCEs (Table 4). We next compare these two gene sets (Table 3 and Table 4) in detail.

### 2.5. Functional Analysis of UCE-Rich vs. UCE-Poor Genes Associated with Brain Functions

Overall, our analysis demonstrated that UCE-rich genes (Table 3) are primarily involved in developmental patterning, neuronal differentiation, RNA processing, chromatin regulation, and transcriptional control (e.g., *CHD7*, *NIPBL*, *HDAC9*, *BCOR*, *KAT6B*, *AUTS2*, *RUNX1T1*, *LMO1*, *LMO3*, *LMO4*, *TOX,* and *TOX3*). A majority of these genes encode nuclear or chromatin-associated proteins. In contrast, UCE-poor genes (Table 4) are enriched for membrane proteins, receptors, ion channels, adhesion proteins, and synaptic proteins (e.g., *CNTNAP2*, *PCDH15*, *DCC*, *NCAM2*, *SHANK2*, *GRM7*, *GRM8*, *KCNQ5*, *GABRG3*, and *CHRM3*) involved in neuronal communication. Accordingly, Table 4 is enriched for plasma membrane proteins, extracellular proteins, receptors, and axon guidance molecules.

Many genes in Table 3 occupy upstream positions in regulatory networks and control the expression of numerous downstream genes (e.g., *CHD7*, *BCOR*, *RUNX1T1*, and *HDAC9*), whereas genes in Table 4 primarily mediate downstream neuronal functions. UCE-rich genes are preferentially expressed during embryogenesis, neuronal differentiation, and organogenesis, whereas UCE-poor genes are predominantly expressed in mature neurons during synaptic transmission and neuronal connectivity. The enrichment of UCEs in developmental master regulators is consistent with the hypothesis that these loci are under particularly strong evolutionary constraints. An additional notable distinction is the enrichment of RNA-processing and alternative-splicing factors among UCE-rich genes (*RBFOX1*, *NOVA1*, *ELAVL2*, *ELAVL4*, *CELF2*, *CELF4*, and *MBNL1*), whereas such genes are largely absent from Table 4.

A functional enrichment analysis of the genes in Table 3 and Table 4 was performed online using g:Profiler web resource [20] with Benjamini–Hochberg FDR correction for multiple testing. The most significantly enriched GO terms for UCE-rich genes (*p* < 10^−6^) were associated with gene expression, transcriptional regulation, RNA metabolism, nucleic acid metabolism, and biosynthetic processes. In contrast, the most significantly enriched GO terms for UCE-poor genes (*p* < 0.03) were associated with membrane proteins, synaptic structures, postsynaptic density, and neuronal connectivity. Complete g:Profiler enrichment results are provided in Supplementary Table S5.

### 2.6. Distribution of UCEs Along ncRNA Genes

We investigated the association between dedUCE elements and a dataset of high-confidence lncRNA genes. This dataset comprises 35,899 genes with a total genomic length of 1042 Mb. We identified 4976 UCEs within 2022 lncRNA genes. Under a random distribution model, approximately 4527 UCEs would be expected within a 1042 Mb genomic region out of the 2949 Mb of the available genomic sequence. Thus, only modest enrichment (~10%) of UCEs was observed within lncRNA genes.

A complete list of all lncRNAs is provided in Supplementary Table S6, in which lncRNA genes are ranked according to the number of UCEs they contain. The ten lncRNA genes with the highest numbers of UCEs are listed in Table 5. The highest number of UCEs observed in a single gene was 67, found in LINC01122 (ENSG00000233723), which spans 927,882 bp. In total, 63 lncRNA genes contained ten or more UCEs. We examined the distribution of UCEs within the LINC01122 gene in greater detail. Interestingly, all identified UCEs are located within introns, with virtually no overlap with the exons of this lncRNA gene (the total overlap is only 38 nucleotides). The distribution of UCEs along the gene is approximately uniform, with UCEs present at the 5′ end, the 3′ end, and throughout the central portion of the gene. In contrast to the intronic UCEs, the exonic regions of LINC01122 are considerably less evolutionarily conserved. The most highly conserved region of the mature transcript is a 370 nt central segment that shares 82% sequence identity with its mouse ortholog, whereas the remaining portions exhibit substantially lower conservation or no significant sequence similarity. Several studies have associated LINC01122 with cancer [21,22] and Alzheimer’s disease [23]. The potential functional contribution of UCEs to the regulation and biological activity of lncRNA genes remains an intriguing question that warrants further investigation.

The genomic clustering of UCEs described above (Figure 2) might be the reason for the presence of numerous UCEs within particular lncRNA genes. Alternatively, some lncRNA genes may be selectively enriched in UCE sequences because these elements contribute to biological functions. Because the functions of most lncRNAs remain less characterized than those of protein-coding genes, it is currently problematic to evaluate associations between UCEs and lncRNAs.

We also examined the association between UCEs and piRNAs, the largest class of small noncoding RNAs. No significant tendency for piRNAs either to preferentially overlap with or to avoid UCE sequences was detected.

### 2.7. Locations of UCEs Along Genes

Table 2 demonstrates a preferential localization of UCEs within terminal 3′ exons. However, this observation should be interpreted with caution because different regions of genes and transcripts vary substantially in G + C content. UCEs are highly A + T-rich sequences, with an average G + C content of approximately 37%, whereas protein-coding sequences are G + C-rich (~53%). In contrast, 3′-UTRs (~45%) and introns (~42%) have G + C contents close to the genomic average (~41%). The 5′-UTRs are the most G + C-rich regions (~60%) [24], largely because they frequently overlap the boundaries of CpG islands. Therefore, the observed enrichment of UCEs in 3′-UTRs relative to 5′-UTRs may, at least in part, reflect differences in the nucleotide composition of these regions.

**Figure 4:**
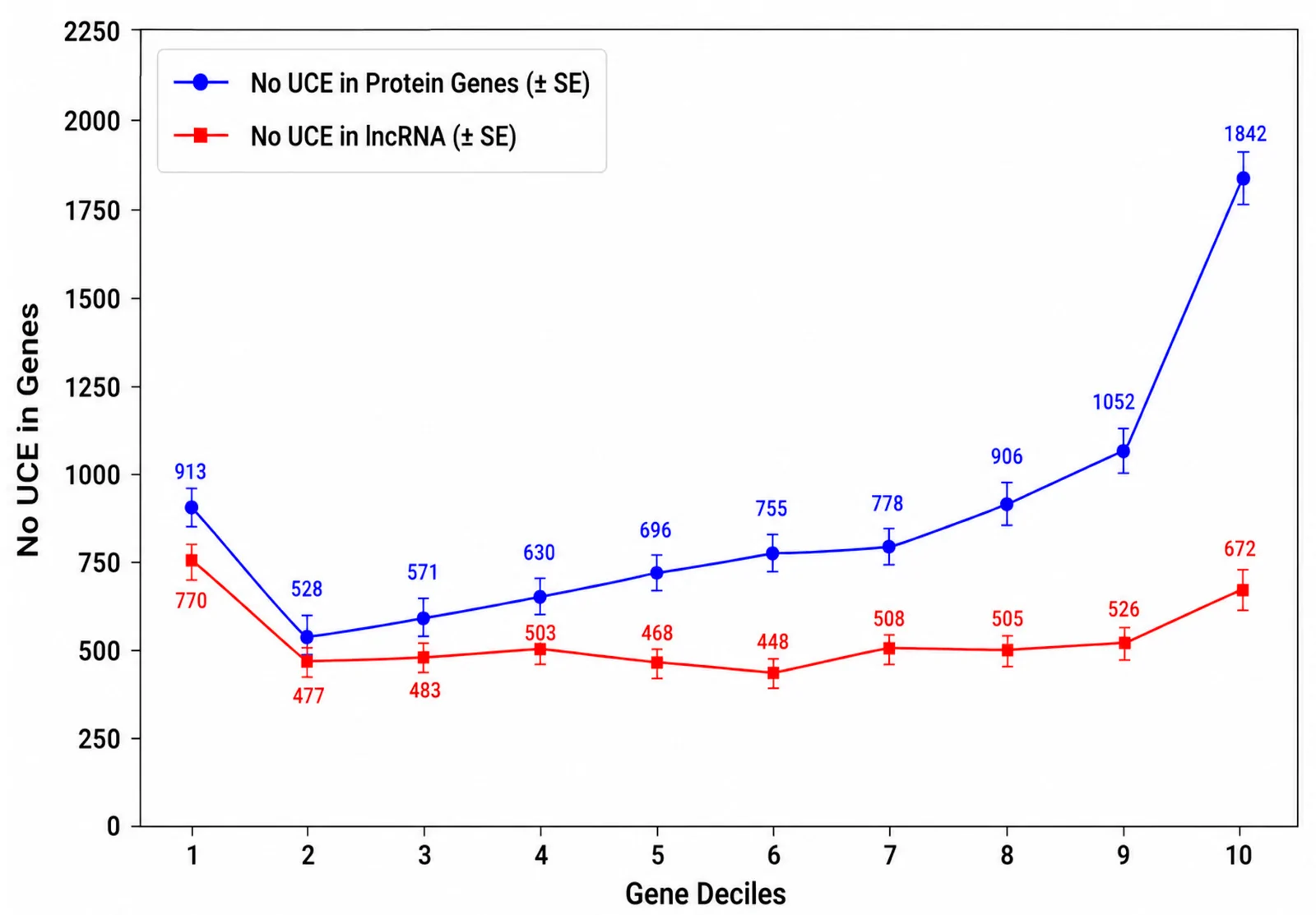
Number of UCEs inside gene-body deciles (bins: 0–10%; 10–20%; …; 90–100%).

To further investigate the positional distribution of UCEs, we analyzed their locations within 20,066 protein-coding genes. Because gene lengths vary dramatically, ranging from a few hundred nucleotides to several million nucleotides, genes were normalized to their relative length and divided into ten equal-length bins (gene-body deciles: 0–10%, 10–20%, …, 90–100%). The number of UCEs intersecting each bin was then calculated. The resulting distribution is shown in Figure 4.

The first bin, corresponding to the 5′ end of protein-coding genes, contains approximately 70% more UCEs than each of the next three bins. Thereafter, the number of UCEs gradually increases toward the 3′ end, reaching its maximum in the final (90–100%) bin. Overall, we observed a statistically significant fourfold enrichment of UCEs at protein-coding gene 3′-ends compared with the middle portions of genes, as well as a twofold enrichment at 3′-ends relative to 5′-ends. The same analysis was performed for the distribution of UCEs along lncRNA genes, also shown in Figure 4. In contrast to protein-coding genes, lncRNA genes showed no preferential localization of UCEs within their 3′ portions.

### 2.8. Association of UCEs with Enhancers and Regulatory Sequences

The association between UCEs and enhancers has recently been comprehensively investigated by Cummins et al. [10]; therefore, we did not repeat this analysis in the present study. Nevertheless, several points merit emphasis. Individual UCEs generally share little or no sequence similarity with one another and collectively represent a highly diverse repertoire of DNA sequences. Consequently, enhancer elements are found within a subset of UCEs, rather than being a common defining feature of ultraconserved elements. Consistent with this observation, no short oligonucleotide sequence (e.g., 10 mer) is present in more than a small fraction of all UCEs (Fedorova et al., 2022 [5]). Furthermore, UCEs are typically much longer than the core functional regions of most enhancers, suggesting that enhancer activity alone is unlikely to account for the remarkable evolutionary conservation of these sequences. We previously proposed that the exceptional conservation of UCEs may instead be related, at least in part, to their distinctive higher-order DNA conformations (Fedorova et al., 2023 [25]; Crossley et al., 2024 [12]). It is possible that these extended genomic regions provide a structural framework that contributes to long-range gene regulation in addition to conventional enhancer functions.

## 3. Materials and Methods

### 3.1. Databases

**dedUCE,** the Database of Ultraconserved Elements, Cummins et al. 2025 [10], was kindly provided by Drs. Mattick and Cummins upon request. The database contains 12,813 human ultraconserved element (UCE) sequences and consists of two files. The first file (merged_cumulative_97_50perc.fa) contains FASTA-formatted sequences with identifiers, whereas the second file (merged_cumulative_97_50perc.bed) contains the genomic coordinates of these sequences in BED format. A small fraction of evolutionarily conserved repetitive elements is retained in this dataset. In the dedUCE FASTA files, these repetitive regions are indicated by lowercase letters.

**UCNEbase** was originally downloaded from the paper by Dimitrieva and Bucher 2013 [17] and was extensively used in our previous publications on ultraconserved elements [5,12,25].

**Human Transcription Factor Dataset:** Human transcription factor (TF) annotations were compiled by integrating information from several publicly available curated databases:

• Ensembl (GRCh38/hg38 release) [26]: This was used as the primary source of genomic coordinates, gene biotypes, and gene models. Protein-coding genes were extracted using the Ensembl BioMart interface and GTF annotations.
• Human Transcription Factors Database (HumanTFDB) [27]: This provided a curated catalog of human TFs, including classifications based on DNA-binding domains (e.g., homeobox, bZIP, and zinc-finger families).
• AnimalTFDB v3.0 [28]: This was used to cross-validate TF annotations and obtain information on TF family classifications and domain structures.
• UniProt [29]: This was used for functional annotation and confirmation of DNA-binding activity based on reviewed (Swiss-Prot) entries.
• NCBI Gene [19]: This was used as a supplementary source for gene-level functional descriptions and annotation validation.

Transcription factors were defined as proteins that directly bind DNA in a sequence-specific manner and regulate transcription. Genes encoding proteins that function as transcriptional regulators but lack direct DNA-binding activity (e.g., co-activators and chromatin remodelers) were excluded. To be included in the TF dataset, proteins had to satisfy the following criteria: (1) Presence of a recognized DNA-binding domain (e.g., Homeobox, bHLH, bZIP, or Forkhead) and (2) Experimental or curated evidence of sequence-specific DNA binding, as documented in UniProt or TF-specific databases.

KRAB domain-containing zinc-finger proteins (KRAB-ZNF family) were excluded from the primary TF dataset because of their large number (~350 genes), predominantly repressive regulatory functions, and distinct evolutionary and functional characteristics relative to canonical transcription factors. These genes were therefore compiled into a separate dataset based on domain annotations identifying both KRAB and C2H2 zinc-finger motifs. As a result, the final TF dataset in BED format contains 1292 genes, whereas the KRAB-ZNF dataset contains 344 genes. All described datasets are available in the Supplementary file Datasets_UCE4.tar.gz.

**Human Protein-Coding Genes:** The representative human protein-coding gene dataset consists of 20,065 genes. It was derived from the Ensembl human gene table for homo_sapiens_core_115_38 (release 115, GRCh38 assembly), which includes genomic coordinates, primary assembly information, and the ensembl_representative_gene_id field. Coordinates for this representative gene set are available in the file human_protein_coding_genes_hg38_representative_primary.tsv.

**Human Long Noncoding RNA Dataset:** The human long noncoding RNA (lncRNA) dataset was generated using the GENCODE Release 49 annotation for GRCh38/hg38 [30], which provides high-quality manually curated lncRNA annotations.

Only genes annotated with the lincRNA biotype (long intergenic noncoding RNA) were retained. In addition, only confidence levels 1 and 2 were included, where GENCODE defines level 1 as experimentally validated and level 2 as manually annotated loci. Level 3 loci, which are based solely on automated annotation, were excluded. Analyses were further restricted to standard reference chromosomes. The resulting file, highconf_lncRNA_hg38_gene_level.bed, contains 34,880 high-confidence lncRNA genes.

**piRNA Dataset:** The piRNA dataset was downloaded from the piRBase database [31] as the file hsa.v3.0.fa, which contains 8,592,561 human piRNA sequences.

### 3.2. Data Processing

All datasets used in this study were generated and processed programmatically using publicly accessible resources and custom scripts [20].

All computational analyses were performed using custom Perl scripts provided in Supplementary File S3. Specifically, we used the following programs:

The program **UCE_BED_process.pl** was used for the calculation of UCE clusters and modeling random distribution of UCE along chromosomes. The output is presented in Supplementary Table S7.

The program **TF_UCE.pl** was used for calculation of the intersection of TF, KRAB, all proteins, and lncRNA datasets with dedUCE. The output files are presented in Tables S1–S3.

The program **GENEquantile10.pl** was used to analyze the distribution of UCE along gene-body deciles. The results are in the GENES_UCE file. All described Perl programs are available in the Supplementary file Programs_UCE4.tar.gz.

### 3.3. Statistical Analysis

*p*-values for the over- or under-representation of UCEs within genes were calculated via Monte Carlo simulations using custom Perl scripts (**MonteCarloTF.pl** and **MonteCarloKRAB.pl**). These programs use a random-number generator to produce simulated positions of UCEs within and outside genes while preserving the same overall frequencies observed in the human genome. The Monte Carlo simulation was repeated 1,000,000 times to estimate empirical *p*-values as low as 10^−6^.

Standard errors for distribution of UCE inside gene deciles were estimated using a subsampling approach. We generated 1000 random subsets, each containing 50% of the original datapoints, recalculated the statistic for each subset, and used the standard deviation across the 1000 values as an empirical estimate of resampling variability. For this purpose, the Perl program **GENEquantileBOOTSTRAP.pl** was used.

Statistics and a functional enrichment analysis of the genes in Table 3 and Table 4 was performed online using g:Profiler web resource [20], which utilizes multiple-testing correction methods (specifically, Benjamini–Hochberg FDR correction).

## 4. Conclusions

1. Approximately 40% of UCEs are clustered in groups within genomic regions of up to 20 kb.
2. Non-KRAB transcription factor genes are strongly enriched for UCEs, exhibiting an approximately 7-fold excess relative to random expectation and also relative to the remainder of protein-coding genes. In contrast, UCEs strongly avoid KRAB-ZNF DNA-binding protein genes (10-fold depletion).
3. Protein-coding genes harboring multiple UCEs are typically exceptionally long, often spanning hundreds of kilobases. Many of these genes are involved in brain development and developmental regulation and generally do not require rapid, high-level expression. However, another large class of exceptionally long nervous-system genes lacks UCEs entirely.
4. In genes related to brain functions, UCEs preferentially associate with those controlling neuronal development and gene regulatory programs, whereas they are largely absent from genes whose principal functions are neuronal signaling, membrane excitability, and synaptic transmission.
5. UCEs show an approximately threefold increase near gene 3′ termini compared with the middle portions of genes.
6. Overall, lncRNA genes show only modest enrichment (~10%) for UCEs across 35,899 genes. Nevertheless, a small subset of lncRNA genes is highly enriched for UCEs.
7. We support our previous conjecture that UCE fragments may represent a special DNA conformation due to their GC-poor composition and specific dinucleotide distributions [5,12,25]. This specific 3D structure of UCE may be critical for the regulation of TF and other master-regulated genes.

Regarding further study perspectives, note that the role of UCE in non-coding RNA should be explored on new data in model organisms [32,33,34]. New T2T genome assembly provides a wide range of plant and animal genomes for the re-definition of UCE sequences [35]. UCE-based genome comparison provides a new approach to distant phylogeny estimates [36,37].

## Figures and Tables

**Table 1:** Genomic signatures (***ρ***) with standard errors calculated for UCNEbase and dedUCE sequences versus the whole genome.

Dinucleotide	Rho Genome	Rho UCNEbase	Rho dedUCE
CG	0.24 ± 0.001	0.27 ± 0.002	0.374 ± 0.002
GC	1.02 ± 0.001	1.30 ± 0.004	1.22 ± 0.003
TA	0.74 ± 0.001	0.76 ± 0.002	0.74 ± 0.002
AT	0.88 ± 0.001	0.92 ± 0.002	0.90 ± 0.002
CC/GG	1.24 ± 0.001	1.08 ± 0.004	1.13 ± 0.003
TT/AA	1.12 ± 0.001	1.12 ± 0.002	1.15 ± 0.002
TG/CA	1.20 ± 0.001	1.28 ± 0.003	1.21 ± 0.002
AG/CT	1.16 ± 0.001	1.10 ± 0.003	1.10 ± 0.002
AC/GT	0.83 ± 0.001	0.84 ± 0.003	0.81 ± 0.002
GA/TC	0.99 ± 0.001	0.94 ± 0.003	0.96 ± 0.002

**Table 2:** Association of 52 UCE clusters with neighboring genes. Non-TF genes are highlighted in green.

Cluster	Gene	Length (kb)	Location	No. UCEs	No. Exons Intersecting	No. 3′-Exon Intersects	No. 5′-Exon Intersects
1	*CASZ1*	160	middle	12	1		
2	RNF220	247	middle	12	0		
3	*NFIA*	386	3′-end	13	6	6	
4	LMO4	20	5′-end	15	2		
5	*lincRNA*	67	5′-end	11	2	1	
6	*PROX1*	58	middle	11	1		
7	*ESRRG*	634	3′-end	12	4	4	
8	*BCL11A*	103	3′-end	11	6	6	
9	*BCL11A*	103	5′-end	12	2	1	
10	*MEIS1*	139	middle	11	1		
11	*MEIS1*	139	3′-end	15	5	3	
12	*lincRNA*	121	3′-end	11	3	1	
13	*ZEB2*	136	3′-end	12	6	4	
14	*ZEB2*	136	middle	17	0		
15	*ZEB2*	136	5′-end	11	2		
16	INTERGENIC						
17	*FIGN*	133	3′-end	13	9	8	
18	FIGN	133	5′-end	13	2	1	
19	*FOXP1*	629	middle	14	2		
20	*MBNL1*	222	3′-end	11	11	6	
21	*SOX2*	3	overlaps	12	4		
22	*OTP*	15	overlaps	11	2	1	1
23	INTERGENIC						
24	*NR2F1*	11	overlaps	11	5	1	2
25	*ARV2A*	439	middle	11	0		
26	*TFAP2A*	23	3′-end	11	3	1	
27	*TFAP2B*	25	overlaps	11	5	2	
28	*UNCX*	4	overlaps	12	0		
29	*HOXA3-7*	45	5′-end	13	8	2	5
30	*FOXP2*	603	middle	16	2		
31	*FOXP2*	603	middle	12	2		
32	*FOXP2*	603	middle	17	3		
33	*EBF2*	203	middle	12	0		
34	*ZFHX4*	186	5′-end	14	1	1	
35	*ZFHX4*	186	middle	11	4		
36	*ZFHX4*	186	3′-end	12	6	6	
37	DENND1A	550	middle	11	1		
38	*ARID5B*	195	5′-end	14	2	1	
39	BTRC	203	middle	11	1		
40	*PAX6*	29	3′-end	11	4	1	
41	*HOXC9-11*	5	overlaps	11	5	1	4
42	*DACH1*	439	middle	13	0		
43	*NPAS2*	869	middle	13	0		
44	*MEIS2*	212	3′-end	15	6	4	
45	*MEIS2*	212	middle	11	1		
46	*MEIS2*	212	5′-end	13	5	0	
47	*NR2F2*	14	overlaps	15	6	3	1
48	*HOXB4-6*	5	overlaps	11	7	2	3
49	*HOXB6-9*	5	overlaps	11	7	0	4
50	*ZNF521*	290	middle	11	0		
51	*ZNF536*	488	middle	11	0		
52	*POLA1/ARX*	303	3′-end	13	1	1	

Table 2 NOTES on gene functioning: *CASZ1* contains multiple C2H2 zinc finger domains that bind DNA. RNF220 is a regulatory E3 ubiquitin ligase that controls protein stability. *NFIA* (Nuclear Factor I A) gene encodes a DNA-binding transcription factor. (LIMO4 Domain Only 4) a transcriptional regulator that does not bind DNA directly. *PROX1* (Prospero Homeobox 1) gene encodes a homeobox transcription factor. *ESRRG* is a metabolic master regulator. A direct DNA-binding transcription factor. *BCL11A* gene encodes a C2H2 zinc finger transcription factor. *MEIS1* gene encodes a homeobox transcription factor (TALE class). *ZEB2* (Zinc Finger E-box Binding Homeobox 2). FIGN (Fidgetin) gene encodes a microtubule-severing enzyme (AAA ATPase family). *FOXP1* (Forkhead Box P1) gene encodes a DNA-binding transcription factor. *MBNL1* (Muscleblind-Like 1) gene encodes an RNA-binding protein. *SOX2* gene encodes a DNA-binding transcription factor. *OTP* (Orthopedia Homeobox) encodes a DNA-binding homeobox transcription factor. *NR2F1* (known as COUP-TFI) a DNA-binding nuclear receptor transcription factor. *ARB2* cotranscriptional regulator A” → ARID2. binds AT-rich DNA. *TFAP2A* (AP-2α) encodes a sequence-specific DNA-binding transcription factor. *TFAP2B* (AP-2β) encodes a sequence-specific DNA-binding transcription factor. *UNCX* (UNC-4 Homeobox) encodes a DNA-binding homeobox transcription factor. *HOXA3* gene encodes a DNA-binding homeobox transcription factor (HOX family). *FOXP2* (Forkhead Box P2) gene encodes a DNA-binding transcription factor. *EBF2* (Early B-cell Factor 2) a DNA-binding transcription factor (Collier/Olf/EBF fam.) *ZFHX4* (Zinc Finger Homeobox 4) encodes a large DNA-binding transcription factor. DENND1A gene encodes a Rab GTPase guanine nucleotide exchange factor (GEF). *ARID5B* a DNA-associated transcriptional regulator (AT-rich interaction domain fam). BTRC (β-TrCP1) gene encodes an E3 ubiquitin ligase substrate receptor. *PAX6* a DNA-binding transcription factor—master regulator of eye/brain development. *HOXC9* gene encodes a DNA-binding homeobox transcription factor. *DACH1* (Dachshund Family Transcription Factor 1) involved in development. *NPAS2* a DNA-binding transcription factor component of the circadian clock. *MEIS2* encodes a DNA-binding homeobox transcription factor (TALE class). *NR2F2* (also known as COUP-TFII) a DNA-binding nuclear receptor transcription factor. *HOXB4* gene encodes a DNA-binding homeobox transcription factor. *HOXB6* gene encodes a DNA-binding homeobox transcription factor. *ZNF521* gene encodes a multi-zinc finger transcriptional regulator. *ZNF536* gene encodes a zinc finger transcriptional regulator. *POLA1* the catalytic subunit of DNA polymerase α. Binds DNA non-sequence specifically. *ARX* (Aristaless-Related Homeobox) a DNA-binding homeobox transcription factor.

**Table 3:** Functional characterization of the top 100 non-TF genes with the largest numbers of UCEs.

Functional Subgroup	Genes
1. Neuronal/synaptic/axon-guidance subgroup	NRXN1, NRXN3, TENM2, TENM3, CADM1, ADGRL2, ERC2, CADPS, SCN8A, KCNMA1, PTPRD, MCTP1, DMD, USH2A, AGAP1
2. RNA-binding and alternative-splicing subgroup	RBFOX1, NOVA1, ELAVL2, ELAVL4, CELF2, CELF4, MBNL1, MSI2, SYNCRIP, PUM1, TNRC6B, SMG6, CNOT2, RSRC1, ZCCHC7, ERI3
3. Chromatin, developmental regulation, and transcriptional co-regulators	CHD7, NIPBL, KAT6B, HDAC9, BCOR, SETD5, RUNX1T1, TLE4, AUTS2, RNF220, LMO1, LMO3, LMO4, TOX, TOX3, SOBP, ZSWIM6
4. Vesicle trafficking, membrane dynamics, and cytoskeleton subgroup	TBC1D5, DENND1A, EHBP1, AP3B1, VTI1A, MVB12B, AGAP1, ARHGAP15, FIGN, TBCA, BCAS3, PEX14, LRBA, SKAP1, SKAP2
5. Proteostasis, ubiquitin-proteasome, and signaling subgroup	BTRC, FAF1, PSMD14, MAP2K5, MAPKAP1, OLA1, RNF220, PEBP4
6. Core metabolism/DNA maintenance/general cell biology subgroup	POLA1, MGMT, ACACA, DPYD, FTO, CAMKMT, SLC25A21, MGST1, CDIN1
7. Large, disease-associated, multi-domain structural genes	USH2A, DMD, PKHD1, AUTS2, TENM2, TENM3, PTPRD, RBFOX1
8. Poorly characterized/locus-style/likely accessory genes	ENSG00000285708, ENSG00000273049, ENSG00000271793, ENSG00000285283, ENSG00000282278, KIAA0825, CCDC171, CCDC178, CCDC200, IQCH, DCDC1

**Table 4:** Functional characterization of the longest 100 non-TF genes without any UCE.

Functional Subgroup	Genes
Neuronal cell adhesion, axon guidance, synaptic organization	CNTNAP2, PCDH15, LRRC4C, LINGO2, DCC, CDH13, CDH18, CDH12, DLGAP2, SDK1, EPHA6, NELL1, FRMPD4, PCDH11X, PCDH11Y, SHANK2, GRID1, GRIP1, CDH4, CLSTN2, SPOCK1, NCAM2, SORCS2, TMEM132D
Neuronal excitability, receptors, ion channels, vesicle release, RNA regulation	DPP10, DPP6, GRM7, GRM8, UNC13C, FGF12, PDE1C, CALN1, KCNQ5, KCNMB2, GABRG3, SYN3, CHRM3, RBFOX3, ADARB2, NKAIN3
Cytoskeleton, cilia/flagella, muscle, retinal/structural proteins	EYS, SPAG16, SGCD, SNTG1, BBS9, LAMA2, CFAP299, PACRG, UTRN, KIF26B, MYO16, AGBL1
Glycosylation, carbohydrate modification, lysosomal/sulfatase pathways	GALNTL6, MGAT4C, SUMF1, LARGE1, GMDS, GALNT17, B3GALT1, ST6GALNAC3, STS
Intracellular signaling, trafficking, autophagy, small-GTPase/phosphoinositide regulation	PRKN, RABGAP1L, INPP4B, TRAPPC9, ULK4, DOCK3, MTUS2, RASGEF1B, ELMO1, SLC9A9, PSD3, ARHGAP6, DOCK1, PRKCE, SBF2, ARHGAP24, DLC1
Immune/hematopoietic or inflammatory signaling	BANK1, MARCHF1, AFF3
Secreted, extracellular, developmental, or broadly signaling-related factors	GPC5, NRG3, THSD7B, FSTL5, CPNE4, TAFA2, TAFA1
Miscellaneous/diverse/poorly characterized	CCSER1, ENSG00000250349, IQCJ-SCHIP1, NCKAP5, KIAA1217, CNBD1, RALYL, SCHIP1, LUZP2, TMEM117, SCAPER, ENSG00000287694

**Table 5:** Top ten lncRNA genes with the highest numbers of UCEs.

No.	Gene Name	No. UCEs	Type	Gene Length	Genome Position
1	LINC01122	67	lncRNA	927882	chr2_58427706-59355588
2	ENSG00000233891	45	lncRNA	901723	chr2_59198477-60100200
3	PKN2-AS1	44	lncRNA	1064648	chr1_87620802-88685450
4	ENSG00000271860	43	lncRNA	1179835	chr6_97283267-98463102
5	SOX2-OT	38	lncRNA	847371	chr3_180989509-181836880
6	ENSG00000257522	27	lncRNA	516671	chr14_28875403-29392074
7	ENSG00000228655	25	lncRNA	302582	chr2_143295420-143598002
8	LINC01837	24	lncRNA	280287	chr19_31791826-32072113
9	LINC02327	24	lncRNA	542526	chr14_28829880-29372406
10	ENSG00000295677	23	lncRNA	363036	chr1_87846958-88209994

## Data Availability

The original data presented in the study are openly available in the Supplement to this manuscript (File S1) and online at https://disk.yandex.ru/d/ZSiO0z1Dftsymg(11 August 2026).

## Supplementary Materials

The following supporting information can be downloaded at https://www.mdpi.com/article/10.3390/ijms27167214/s1.

## Abbreviations

UCNE	Ultraconserved Noncoding Elements
UCE	Ultraconserved Elements
TF	Transcription Factor
KRAB	Krüppel-associated box zinc-finger (protein domain)

## References

[1] Bejerano G., Pheasant M., Makunin I., Stephen S., Kent W.J., Mattick J.S., Haussler D. (2004). Ultraconserved elements in the human genome. Science.

[2] Stephen S., Pheasant M., Makunin I.V., Mattick J.S. (2008). Large-scale appearance of ultraconserved elements in tetrapod genomes and slowdown of the molecular clock. Mol. Biol. Evol..

[3] Habic A., Mattick J.S., Calin G.A., Krese R., Konc J., Kunej T. (2019). Genetic Variations of Ultraconserved Elements in the Human Genome. OMICS J. Integr. Biol..

[4] Snetkova V., Pennacchio L.A., Visel A., Dickel D.E. (2022). Perfect and imperfect views of ultraconserved sequences. Nat. Rev. Genet..

[5] Fedorova L., Mulyar O.A., Lim J., Fedorov A. (2022). Nucleotide Composition of Ultra-Conserved Elements Shows Excess of GpC and Depletion of GG and CC Dinucleotides. Genes.

[6] Dobzhansky T. (1973). Nothing in Biology Makes Sense except in Light of Evolution. Am. Biol. Teach..

[7] Katzman S., Kern A.D., Bejerano G., Fewell G., Fulton L., Wilson R.K., Salama S.R., Haussler D. (2007). Human genome ultraconserved elements are ultraselected. Science.

[8] Drake J.A., Bird C., Nemesh J., Thomas D.J., Newton-Cheh C., Reymond A., Excoffier L., Attar H., Antonarakis S.E., Dermitzakis E.T. (2006). Conserved noncoding sequences are selectively constrained and not mutation cold spots. Nat. Genet..

[9] Halligan D.L., Oliver F., Guthrie J., Stemshorn K.C., Harr B., Keightley P.D. (2011). Positive and negative selection in murine ultraconserved noncoding elements. Mol. Biol. Evol..

[10] Cummins M., Watson C., Edvards R.J., Mattick J.S. (2025). The evolution of ultraconserved elements in vertebrates. Mol. Biol. Evol..

[11] Ahituv N., Zhu Y., Visel A., Holt A., Afzal V., Pennacchio L.A., Rubin E.M. (2007). Deletion of ultraconserved elements yields viable mice. PLoS Biol..

[12] Crossley E.R., Fedorova L., Mulyar O.A., Freeman R., Khuder S., Fedorov A. (2024). Computational identification of ultra-conserved elements in the human genome: A hypothesis on homologous DNA pairing. NAR Genom. Bioinform..

[13] Corra F., Crudele F., Baldassari F., Bianchi N., Galasso M., Minotti L., Agnoletto C., Di Leva G., Brugnoli F., Reali E. (2021). UC.183, UC.110, and UC.84 Ultra-Conserved RNAs Are Mutually Exclusive with miR-221 and Are Engaged in the Cell Cycle Circuitry in Breast Cancer Cell Lines. Genes.

[14] Bozgeyik I. (2022). The dark matter of the human genome and its role in human cancers. Gene.

[15] McCole R.B., Erceg J., Saylor W., Wu C.T. (2018). Ultraconserved Elements Occupy Specific Arenas of Three-Dimensional Mammalian Genome Organization. Cell Rep..

[16] Leypold N.A., Speicher M.R. (2021). Evolutionary conservation in noncoding genomic regions. Trends Genet..

[17] Dimitrieva S., Bucher P. (2013). UCNEbase—A database of ultraconserved non-coding elements and genomic regulatory blocks. Nucleic Acids Res..

[18] Sanderlin A., Bailey P., Bruce S., Engstrom P.G., Klos J.M., Wasserman W.W., Ericson J., Lenhard B. (2004). Arrays of ultraconserved non-coding regions span the loci of key developmental genes in vertebrate genomes. BMC Genom..

[19] Sayers E.W., Beck J., Bolton E.E., Brister J.R., Chan J., Connor R., Feldgarden M., Fine A.M., Funk K., Hoffman J. (2025). Database resources of the National Center for Biotechnology Information in 2025. Nucleic Acids Res..

[20] Kolberg L., Raudvere U., Kuzmin I., Adler P., Vilo J., Peterson H. (2023). G:Profiler—Interoperable web service for functional enrichment analysis and gene identifier mapping (2023 update). Nucleic Acids Res..

[21] Xing C., Cai Z., Gong J., Zhou J., Xu J., Guo F. (2018). Identification of potential biomarkers involved in gastric cancer through integrated analysis of non-coding RNA-associated competing endogenous RNA network. Clin. Lab..

[22] Shang Z., Jin S., He Y., Zhu Y., Zhang H., Wu J., Hong Z., Ye D. (2025). Comprehensive analysis of the LINC01122/TPD52 axis as a predictive biomarker in prostate adenocarcinoma. Sci. Rep..

[23] Dorfsman D.A., Prough M.B., Gulyayev A.V., Caywood L.J., Clouse J.E., Herington S.D., Slifer S.H., Adams L.D., Laux R.A., Song Y.E. (2025). Linkage and association of preserved cognitive function in the Midwestern Amish at a higher genetic risk of Alzheimer’s disease. Alzheimers Dement..

[24] Mignone F., Gissi C., Liuni S., Pesole G. (2002). Untranslated regions of mRNAs. Genome Biol..

[25] Fedorova L., Crossley E.R., Mulyar O.A., Qiu S., Freeman R., Fedorov A. (2023). Profound Non-Randomness in Dinucleotide Arrangements within Ultra-Conserved Non-Coding Elements and the Human Genome. Biology.

[26] Yates A.D., Austine-Orimoloye O., Azov A.G., Barba M., Barnes I., Barrera-Enriquez V.P., Becker A., Bennett R., Berry A., Bhai J. (2026). Ensembl 2026. Nucleic Acids Res..

[27] HumanTFDB. http://bioinfo.life.hust.edu.cn/HumanTFDB/.

[28] Hu H., Miao Y.R., Jia L.H., Yu Q., Zhang Q., Guo A.Y. (2019). AnimalTFDB 3.0: A comprehensive resource for annotation and prediction of animal transcription factors. Nucleic Acids Res..

[29] The UniProt Consortium (2025). UniProt: The Universal Protein Knowledgebase in 2025. Nucleic Acids Res..

[30] Mudge J.M., Carbonell-Sala S., Diekhans M., Martinez J.G., Hunt T., Jungreis I., Loveland J.E., Arnan C., Barnes I., Bennett R. (2025). GENCODE 2025: Reference gene annotation for human and mouse. Nucleic Acids Res..

[31] Wang J., Shi Y., Zhou H., Zhang P., Song T., Ying Z., Yu H., Li Y., Zhao Y., Zeng X. (2022). PiRBase: Integrating piRNA annotation in all aspects. Nucleic Acids Res..

[32] Cheng M., Zhu Y., Yu H., Shao L., Zhang Y., Li L., Tu H., Xie L., Chao H., Zhang P. (2024). Non-coding RNA notations, regulations and interactive resources. Funct. Integr. Genom..

[33] Jin X., Liu L., Liu D., Wu J., Wang C., Wang S., Wang F., Yu G., Jin X., Xue Y.W. (2024). Unveiling the methionine cycle: A key metabolic signature and NR4A2 as a methionine-responsive oncogene in esophageal squamous cell carcinoma. Cell Death Differ..

[34] Orlov Y.L., Tatarinova T.V., Anashkina A.A. (2021). Bioinformatics Applications to Reveal Molecular Mechanisms of Gene Expression Regulation in Model Organisms. Int. J. Mol. Sci..

[35] Chao H., Ruan Z., Li Z., Zhou X., Zhang S., Shen X., Feng C., Chen D., Chen M. (2026). T2T-Hub: A central platform for analyzing plant and animal telomere-to-telomere genomes. Nucleic Acids Res..

[36] Derkarabetian S., Benavides L.R., Giribet G. (2024). Reassessing the phylogeny of Cyphophthalmi with phylogenomics: A UCE-based phylogeny of mite harvesters (Opiliones). Mol. Phylogenetics Evol..

[37] Orlov Y.L., Orlova N.G. (2023). Bioinformatics tools for the sequence complexity estimates. Biophys. Rev..

