# Supplementary material for: Non-Random Association of Ultraconserved Genomic Elements (UCE) with Human Genes
Source: Int J Mol Sci. 2026 Aug 13;27(16):7214. doi: 10.3390/ijms27167214 (PMC13513235; doi:10.3390/ijms27167214)
Supplement: Supplementary file 1 [file ijms-27-07214-s001.zip › Suppl_File_S1.pdf]

chr1 CLUSTER 0 CASZ1 gene 160kb; positions on BED file: 40 - 51
>NC_000001.11:10672272-10672462
GGCCGCCCAGCCTCCAACATGCCCCGCGCCCGGGCCTCTCAGGAAGGTCATTACAAACGACTTTCC
GATTCACTGCTCCTGAAATAATTTTGTGTATTAAAACCTGAATCTGCACTTTCTGGGGCCGAAAGCCT
CGCTTAATTATGGCGGGTGTCCTTGGGACGGACAGTGATCCTTCACTCGCCAGCCC
>NC_000001.11:10672489-10672814
GCCCCCCTCCCCGGGCCCAATCTGTTTTCAAAGTGTGTCTGTCCtttattaaattgttttcttttccacattATCAGTTG
CCATGGAGACCTCATCTCTCGGATTATTTGAATTTCATTATATCTATTGATTtgggagcatttcatctttttattgtttttc
tgtcaaTTTTCAAAACGAATAACCATCTAATTTGCGTCAGTGCTGATTATGTTTGTCCCTCAATAGAGGTC
GGCAGCTGCGCTGGGGAAACAAATCAATGAAAAACCATCATAAAACTCCCCACTCCAGTCTCCAGGA
TGTGTGGGTGACATTCCACGCCTG
>NC_000001.11:10673178-10673384
tggtttttttaaataatttatgcaaTTTTAAGcatttatgtataaatttttaataagcCACCCTGGAGCAGGATGGCCATTAACATC
CAACGTCCTTCTGTCCAATGGGCTGGCGGAGAGGATCAATTTCTGAGAGTACTTTCCCTTTTTTATGA
CAtgataaaatgcttttaaagcaACTTACACaaatatggaaattttttt
>NC_000001.11:10673528-10673673
GGGGGCTGGCAGGGAGACAGCCCCAGTTCTAACTGGAACTGGCCGCCTGTCCTTCTAACAAGGGC
ATAAACTTTCATTACCCATGCACGCAGTCAAAGACAATTTAGGGAAACGCGCTGCTTGGAAAAGGAA
AACCTCAGCGATT
>NC_000001.11:10675109-10675292
CCCGGCCCATGTTTGCACAGAAAAGCTCACGGCAGGTCCCCCCTCCTGACGAAGTGACTTATTAAA
GTTTAAACAGTAATtaacagaacaataaaaataaagggatgtttGGGGAGTCGTAGCGCACACAGGGTTTTTGGCA
GTGCCAGCAGTTTTTCAGTGCCCTGCGCTGGCAGCAAAATG
>NC_000001.11:10678588-10678956
GCCGGCTCGCGCAGGGGCCGTCAGGTAAATTAGATCTGAAAGCTGACAATTTCTGACCATATTTCCT
TGATTATTTCAAACAAATGCACAGCAGCCGCTGTAAGGAGATTAAAGTGACATAAACGTCCCGAGCG
GGAGGGGGGAGGGCAGAGGATTCAGGTCACCCCCATCCGTCCCCCGCCTGACAGACGCTATATCG
CTATCCAATCCAATAAAAAATCCCCCCCTTTTGCTTTAATTAATTTTACAGCTAGCTTGCTTAATTACTT
TCAATCAAAATCCTCCTGCCATCGCAAAATTAGAGATGGTTGAGCTCCATTAGCCTAAATTCTTCATTT
CCATATAGAAAAGAGGCTGTCTGCAGAGCC
>NC_000001.11:10686667-10686767
GCCTTATTGCTTCTCCCCTTAATGGGGCCGTAACCGTGGAAACGAGGAATAAACGGCCACCAGAAAA
TGAGATGATTAAACAGGTCACCCTGGCAAACAA
>NC_000001.11:10691104-10691335
cctttttttttattagcattTGTGGAATTTATTCCCAAACTCTCCTAATCTTCCGTACACAACCATTTGATTTGCATA
ACCCAACCCCCTCTCATAAAAACTGGCTGctagtttaattaaaaaatgtaaatttgctGTCATCTCGGGGCCTGGTG
AATTAGGACGACATCGGCATTTTTTATTGCTAAAGACGTCCAGATTGATCCAGCCCTTGGCTGAACTA
TGGTGGG
>NC_000001.11:10693325-10693441
ACACAGCCTTGTCCCAAATAAATTAAATTCCTTTACCTTGAGACAGTCGCCCCGAAACCAGACTAACC
ACCTTTCCCAACAGGACGCTGCTTTCAAAAACTAAACCACCCcccact
>NC_000001.11:10694285-10694405
CCGGTACTCACCATAGTCGGAGAGTCGAAAGCCGAATTCACTTAAATAATCAACTTTCATAATACTTA
ATTAATGCCTAATTGCAACTGATTACTCCCCGAATAACAAGTTGTTTTAAAG
>NC_000001.11:10698191-10698558
CCCGTAATCCTGTTAGCCAGCAATCTCCATTCCGAAAGAGCTTCATTTCTTTGTGAATATTTGTAAACT
AGATATCTAATGGAAAATTATAGGAAAATTATAGTGAAATTCATGACGGAGAAATCCGATTATCCCTAA
TTCAATTATGCCATCTTAGCCCACACAGcacatttcccattttttcccataaataCCGATGCCAGGGGTCCCGGTAat
caaataaaattgaaaatcatcTTCCCATTAAATTCAATTAGCCGCAATTTCTAAAGCCTATCAGAGATTTTAATta

ctaaattataaaattctcCAATGctaaattgaaaccaaaaaaattgCACATCCTtatatttcagagaaaaagaa
>NC_000001.11:10699571-10699700
GTTTAATTTGCCAGGCAGCCAGGAGTGCCAGCAGGGGAGGCACccgcaaacaaaacaaaaacaaaaaagttaat
tatctttggctttttcttaataaatatccAAACTGGCCCGGCCGGATCCTCCACt

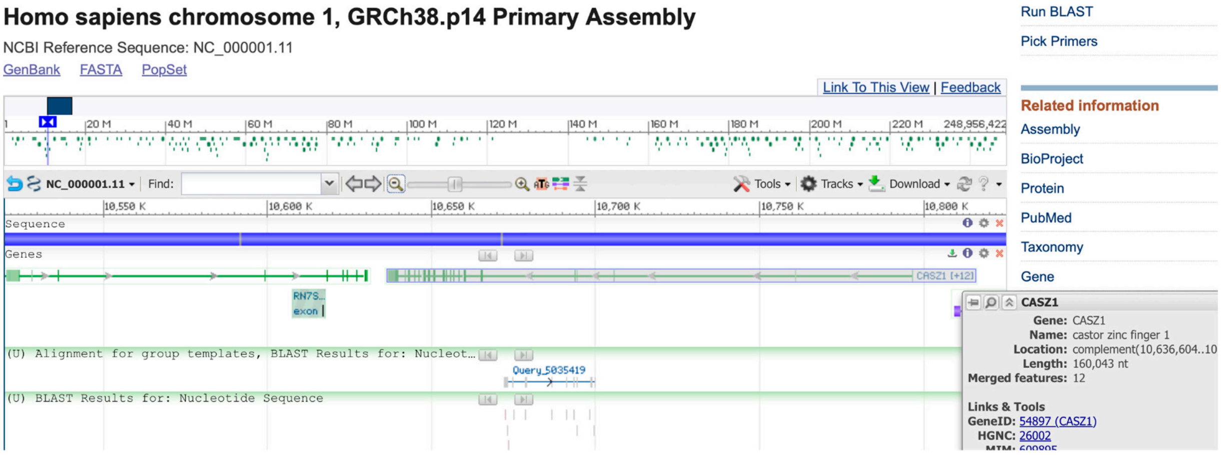

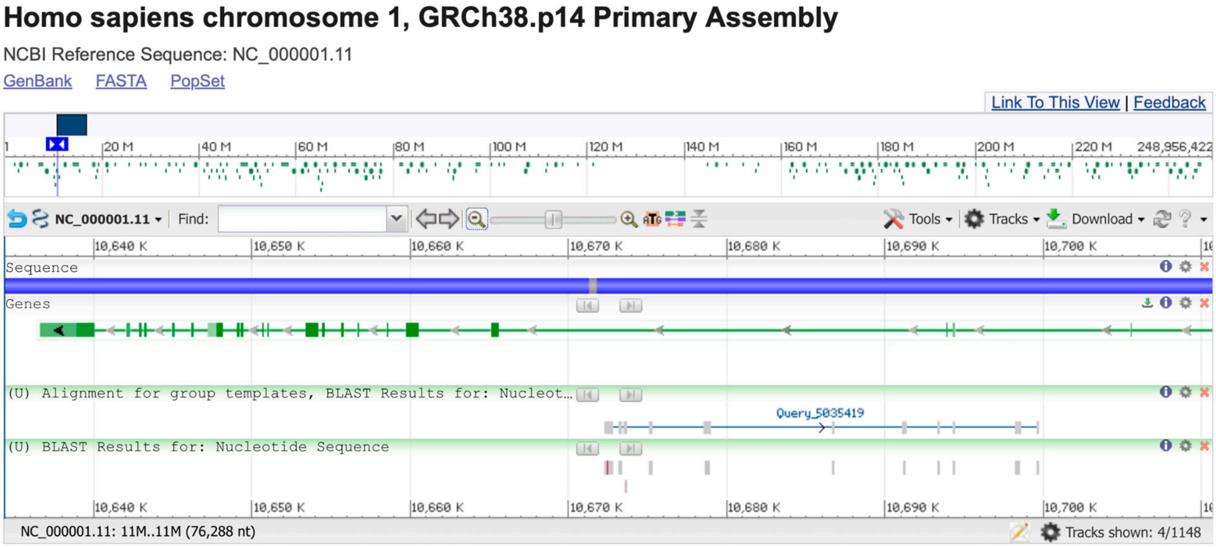

chr1 CLUSTER 1 positions on BED file: 334 - 345
>NC_000001.11:44598295-44598488
GTATTGCTATTAATCACCCCTCCTGGTCCGCTAACGAGGCTAATGCGGTAAGTGACAATAGCCCATC
TTGGTTCCTGATTTAGGGGCTCAGGACGGAAGCTGGGCTCCTGGGAGTGAAGACGTGAATTCTCCA
CGACACTAAACCTTCAAATAAATCTCCTCCAGTCAGAGAAGCCCAGGGCACTAAATCCCA
>NC_000001.11:44602782-44602951
GGAACCCCTGCACCTTCATTTCTCAGGTCACTGCTTGGCTAATGGGCTATTAATTTGCTCAATCGTCC
CCTGTGAGGCAAACTCAATTTCTGCCAGCCAGTCGTCCCTCCCTGTCGACAGCGTGATTGATTGGAG
GAACAGCTGTCACCGCCCGGCTGCCTGctccctg
>NC_000001.11:44603010-44603251
CCCATAGAGGCAGCACACCCCTGCCACCTGCCCAAGGATCTGTCACCAGCAatgaaaaatgacagaaatattt

aAGGACAAATAAGGTTTCCCAACGCAATTGAAAATGATTACAGGCAGCTACAGATGGGGCCAATCTG
ATGCGCAGCAGCAAATCAAATCGAATTTCCTTATCGGCGTATTATTTTAGGCGTTATCTGGCACGTTG
ATGCAGTTATCAATTCAATTTCAGCCGCCGGTC
>NC_000001.11:44603529-44603676
GCTGCTGCCAGGGAGAGGTGAAGGTCTCCCATTAATTCCCAGCTAAAAATGACAAATTCATCTTGGA
CGACAATTAAATGAAGTCAGTAATTGATGCTAATTGATCTCCAGATGCAAGGGCTCCCCTCAGCCTTA
GCAGCACGTGGC
>NC_000001.11:44606025-44606324
TCAACCAGGGATGCCTATTAATCCTGACAAATGATGACTTACGAACATGTAATGCAAACTGATTCATC
ATACATTCAAATCCGCTGCCCCGCCCCAGCGGGAGGCCCCAGCCAGACAAGTGTATTACACGGATA
ATGTATTTGACGTATTAATAAAACCAGCCGCATTGCTCTGGCCCGGAGACTGGGGAATGCCAATATA
CGATGACAGATCACATATTGATGAAATCCAGATGTTTGGAATCATAGTCTTGGGGCCTCGTTCATATT
GACTGAAGGTAAAAAATGCCATCTGTCAGC
>NC_000001.11:44610181-44610323
ACGCACTCACACACTCAATCAGGCGCTGTCAGACCTCTGGCCGCCTGGCATTTTGGTGAAGAATCTC
TCAGCTAAAAATTGTTGTCAGTCCCATTTCCTCGTTATCAGTGCAAGGGCTGAGGCAGCGCCCGGGG
ATTAGCAG
>NC_000001.11:44610919-44611098
TTTTATGGCCACTGTAATGGCCCAGGTCAAAAGCTTCAGGGAGCTGTATTCTGGGAATTATGCAAAC
GCGGAGCAGGCCCAGGGCCAGAGTATGGATTTTGTCATTAAACCTGGGCAGGCCATGTCGTTAGTG
CAGTAAAACCCACGTCATGACCACAGGtctcaatataaaattatag
>NC_000001.11:44617154-44617289
GGTGTCTGACAGCCGGACAGCGGGATCTGCACAGCAAGTGAAATTCCCGTCGGATCGATAAAGCCG
AAACGCTGGCGGCGACGGGTCGATGGTTTTATCTGTCTCGGCCCCGCCGCGCCCGGCGCGCTGAC
AGTT
>NC_000001.11:44617404-44617542
GCCGCCGCCCTCGTGATTTATCTGgatttttaattggaaaaaaatttGCGAATTAATTGATTTCAGGAACTTGCCAA
TTAACATTGTAATTGGGTGCGTGATAAATTCCCAAGGAGCGTGTCAGCTCCCTCCGGGCTGA
>NC_000001.11:44619374-44619678
cagtgcccccacccccacttaaGCCTTATTTAAGGCCGGCCTCGGTGTTTGGTTGGGTAATGAACTAGATAAAC
AATTCCCCAAATAGATTAAAACGAAGTGTAACTTACAAAGGCGGCTGGTGATGATGGTTTATTCCCGG
GCAGTGCACCATTTCTTTATTTCCAAGGATAATTCAGTGTAAATCACCTTAATTAAAAGTGTCAGCCCA
GCCCATGAATATTGTACTTAGGAATGCGTTTCCTGGGTCCCAGTTATAATTTAAAACCCATGGATCAC
TAGGCAGCGAGTGACTGAGCAAGGG
>NC_000001.11:44621976-44622096
aGGAACAGGGATATTAATGTTTCTGAAGTGGATCTGATTTGATACGTCTTGTTCCATTGTCAGCATCT
GTTTAGCAGGGATTTGTAATACTTTGTGGCTCTGGATCACTCATGCTTAGCG
>NC_000001.11:44624710-44624909
TTAGACAAGATTGATGGCCTCGttgccattaagaaaaaaattagccggtggcGGCTATCATATTAAAGGGTGAAGA
AGCcgtgaattattttcttaaagatcTTATCGCTTACAATGATAATTTTACCTTCCAAAAGGCCACTTTTAATGAGG
CGAAGAGCCAGGCAAAAAGTCATGATTTAATGCcgggattttttt

Homo sapiens chromosome 1, GRCh38.p14 Primary Assembly

NCBI Reference Sequence: NC_000001.11
GenBank FASTA PopSet

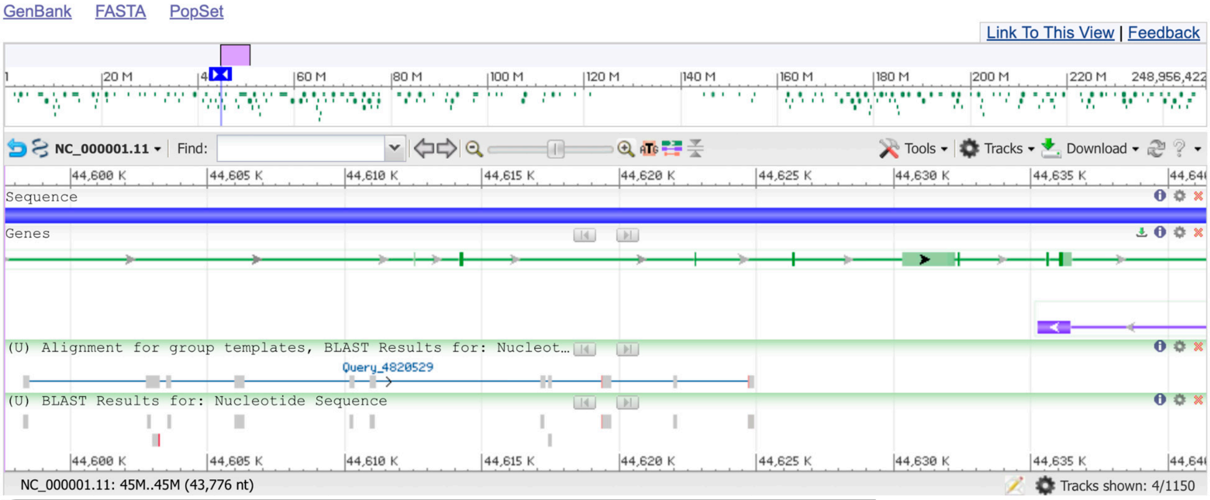

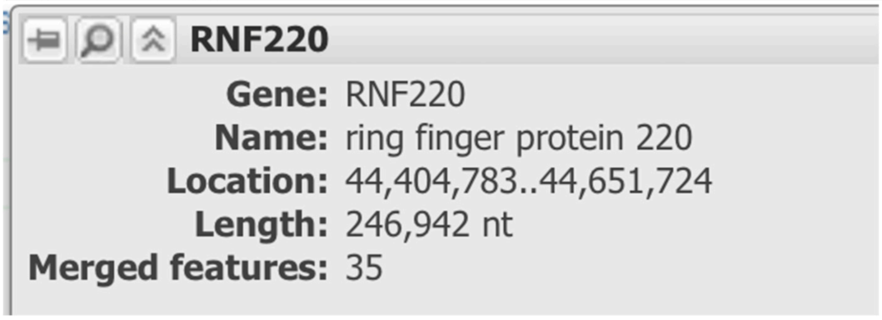

chr1 CLUSTER 2 positions on BED file: 478 - 490

>NC_000001.11:61447692-61447808
TAGAATctatttttcatgaaaaagcaGGAAATAAGCTGTCTCTTTCataaaaagatttacaaaatgTAATCATTGTTGAAA
TATTCCCTTTGAACTGCCGTGTCCACGGGGGTCTC

>NC_000001.11:61447882-61448148
GGCTTTGAtgtcttgaatgaatgaaagaaaagcacaataaaaaaGAGCTGTTGATCAAGCATAAAATACTCTTTAAGC
TGACAGAATAGCAGCGTTAACACACCCTAAGCTGCTTttagtttttcccttttttcctcacATTTTCCTGTGCTTTGTG
TACAGTGGGGGTTTGTATTTTATAAGAAGTCTCTGATCTATGTAGTATGCCATTCgctaacaaaaaccaaaat
aggaTTGTTTCTGTCCCACTTGGTTGCCTGACtacc

>NC_000001.11:61452735-61452872
GGAGTCAGCAACTTCTATTGATTAAAAACTAACCTTTAAAGTAACTCTGAGAGTGTGTGGTTagcatttaa
atttaaacatgtcATGAGTTGATTTGTGTTACTGAATACCAACTCAGAAGGAGTAACATTCACatc

>NC_000001.11:61453016-61453120
GCAGAGTGAAACACAATAATTAGTCCTTTGTCTAACTTTCCCAAGTGCCAGAGAAAGACAGATTAATT
AAATATACAACAATGTTGGTTTTTGGAGTGGGGGTC

>NC_000001.11:61453222-61453404
AGAGGGTTTCTTGCTAATGATAGTCACGTCTGGGTCTGTCTGGTTGCTCTTAGCAACCAGACATGAT
GTAACACCAGGATGAACTTGAACTTGGGGGACTTGAAAAGAGAACAATAGACCAGAGCAATCAATAA
AGCCTATTTCAGTGAAGGATTACAGAGCTGCTCTGGGGTAACCTTGTC

>NC_000001.11:61456099-61456375
ggaaatggGGAAGatggtctgttttgacagaaACTGACAGGAATCAATCAAAACAATCGAATTTTGAATTGAGTAA
AGTGCAATTTCATTGGATAGCTAAATATCTTTGTAAGATAGAGATTGttgaaaattctattttgtttttctagtcctTTCA
CCCCAGGACTCTAAATTATTGGGGTAAAAAACAGCCTTGCAAGAAAAAGGGGagctatttttgctttttatgttttttatt
gttaaaCTTGTATCCCTTtaaaaactgaaggaaatta
>NC_000001.11:61456970-61457192
GCTGATCAGAGAAAGTCTCTGCATGTTCTAGTGTTAGtaactaatttttatatagttAATGTAGGATAAAGTAGAG
TGCATTAAGACACAATATTGTAATCCCTACTCTAGGCACTTGCCTTTAAACTATGTTTTTCAGCCCTTC
AGAAGGGTTCTACTACTGTCCTATACAATCAAGTAACTGAAATTCTTGGGAAGACACTTTGCTCCTCA
TCTTTCTCC
>NC_000001.11:61457195-61457321
AAacaatgttgttttgttttgtttttttccttaatttgcaCGAAAACAAAAATTCCATATCAATGTGCCTTGCCCTGGATAGCG
ATTATTTGTGGAATTGTTGCACATGCTCCTCTATTGAAAGG
>NC_000001.11:61457709-61457846
tgatttttgaatGCACGTGCGCAGGAAGGGCTCCTCTTAGAGAAGCAGTCAAACTGTGAAGCACTAAGCTG
ACCCTGCTTCAAGCAATTTTGTTTTTACAACTGTTCCTTTCACAAgcaagccttaaaaaaaaaaa
>NC_000001.11:61457915-61458069
taaaaagtatataatgcCCATTTTTATATGCACGTTTTTAAACTTCCAAGTTCTGAAAATTGTTTACTGGTTATC
tctatttaaggaaaaaaaaataaaataaaacattttggattttcataTGTGTCTGATAAGTGGTTGAATAGTCGTTTGG
>NC_000001.11:61458473-61458581
TGTTGTATAGTTTTATTGATTACACTGATTTATTCTACCCTATTTTATAATGCAGGACTTTTGTAATGTT
gtttaaatgaggaaaaatttCTGTCAAATTAGCCTAGT
>NC_000001.11:61460303-61460869
GCTTTGATATATAGCAGGTAACATTGAAGCTATTCCATAGCACTTAACTGTAGTGAATACTGTGTCAC
CAATTTTGAAATCAATTTAATGTTTAATGCAAATCCATTACATGGTGCTATTATAGGCTGACAAAATGA
TTTACACAAATGTGACAACTTGGGCTCAATTCACTCTGCTTTCCAACAGTGTAAATGCATAGCAGTGT
TTATCTGCATGAGAACTATGCACTAATCtatctgaagaaaaaaactatatCAACTTTGGTATCTACTTTCCGTTTA
CTTCAATCCTTGCCTTTTTGGTCATTGTTATAATGCCAGCTTTAGGACAGAAAGAATTATAAGAAAACC
AGCATAATACCTGATATATTAAAATGTAGTGCCTGTGAAATCTGTATTATATTGCTCTTCTGAAGTAAG
ATTTTTCTACACCGGTAGCCTTCGCTGTCTGTCAGTCAGGACCTTCTGGTATAGGTGATGTAAAATAA
CCGTACAATATTAATGCATGCGATTCCATAATGCTTAGTGAACTGTATGAATATTACTCAAAGTTATGT
TAGTCTTTTT
>NC_000001.11:61462553-61462791
TACCCTTTTAGTTTTACTGTGTTCTGTGAAAATTTGTAATTGGTTGAGAATCACTGTGGGCGTCCATTC
TTATTCAACTAAATCTCCACAGGTTTTTTGAGCTGGTGTGGATTAGTTTAACTCTTGTATTCAACCATT
AGTGCTACCACCTTCTCACATTACAATACAATTACTGGAAGCAAGTACTGCATTTCCTAtgcaacaaaaaa
ggaaaaataaaaaattgctaaTGCTATGG

Homo sapiens chromosome 1, GRCh38.p14 Primary Assembly

NCBI Reference Sequence: NC_000001.11

GenBank FASTA PopSet

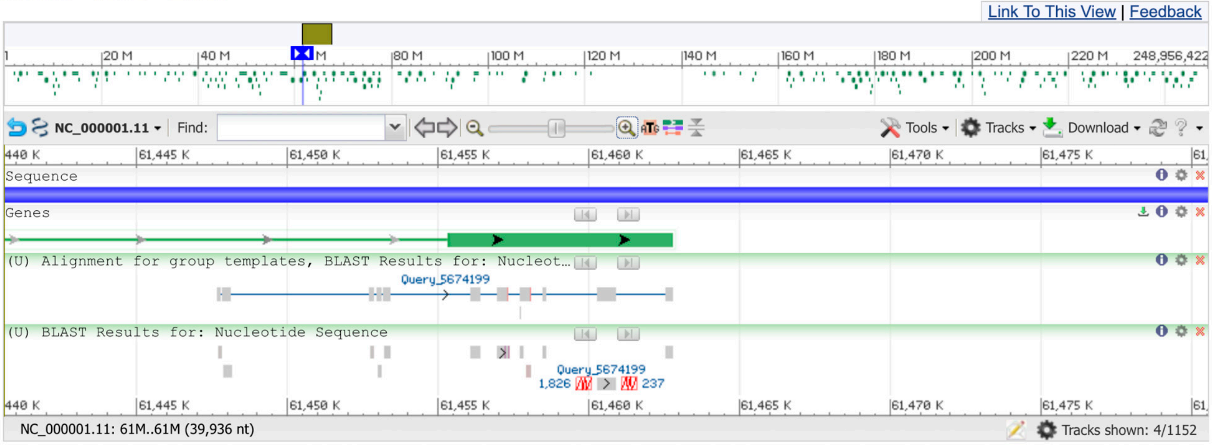

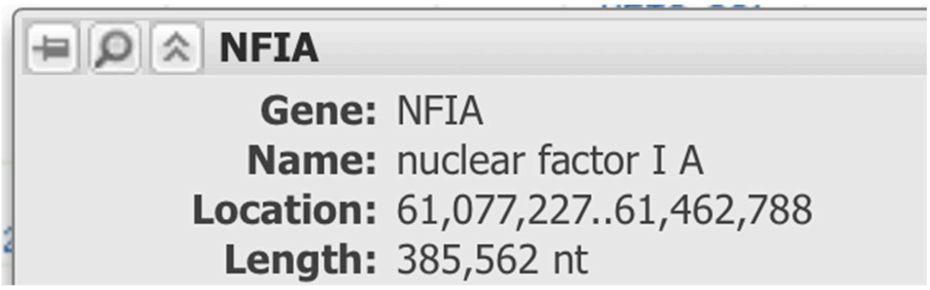

chr1 CLUSTER 3 positions on BED file: 657 - 671

>NC_000001.11:87310764-87310873
TATTACAGAAAGAAGGTAATTTTTCCTGGCCATTTACTAAAGCTCATTAAACACTGGAGAGAACAAAC
AATGCTACTCCTCAGAGACTACCATTCAAAGTCACTCTACA
>NC_000001.11:87329745-87329848
CCAATCCAGGAGAAATACGGTGAATCACCTAATTAAAATCAAGACATGAATTAAGCATCCATTTTTGT
ACTACAAATTGCCAGCGTTACGTTTGTCCCTCCCT
>NC_000001.11:87329875-87330012
GTTGTTAAAGGGGGGCAGATTTTGCCTCTAGCTGCCAGTTCTGCTTTCTATTTCACTGACTATCCCAG
GACCTAAATTGCTTGGCTATGTAATTACTAGAGTATAAGCCTATTTAActtaaaagctttctttttttt
>NC_000001.11:87330014-87330207
tttttttccaactctaaATGAAACTTGATGAGGGCCCGTCCTTAGTTATCAGGGGAGTCAGTTTCTGGTCAGTC
TTCTTGATTCATCTCTTTTAGATTTAATCAGCATTAAGGTATTGCTTGTCCTTAAGAGCTTTAGCTAATG
GGGTTACTGGATGCTTTTCTCAgtgtaatgtttcctttcaaaaaaaa
>NC_000001.11:87330333-87330482
AGGGCAGGAAATGCAGATTCATTTGGGTTAGGGCTGAAATTGTAACAAAAAGCAATTCCTCGAGTAA
ATGCATCAGATAAATCAATTTACATAAAGGTATGATGCATTCGGATAAATGCAATGCTAATGGGTTTTA
GAGTGGTCCTGTT
>NC_000001.11:87330484-87330590

CAAAGTCTCAGGGTTTTGTTACAGCTTAATTGGTGCAGTTCTTATTCTGGTTTATTGTGCGGTCACTG
GACACACGTTCCGGGACTCTTTCGGAGGAAATGTGTTT
>NC_000001.11:87331106-87331315
TTTAAATTGCTGATCAAGGGCAGATTAGTGAGACCAAAGTGGAGAGTGTTTGTATAGGAAGTTAACA
GGCATCCTAAAGTTCAATGATCTATTATTCTTGCCTGTGTCCTGAAAACCCAGAGAAAACACTCATTG
ATCttcaaaatgaaatgagatttggAACAATAATGGGAGATGGCGGTCACCACAATGGCATGTGAAAGGTGCTG
>NC_000001.11:87333121-87333317
TAATGGAAAGAATTCAGGAGGTCAGGTGCCTATAGGATCTTTTCAGGTGAACTCAAGCTACTTAAACT
CATTAATTTGAAAGGAGGCATTTGAAGGATTACACATTTAATTTGAGTAAATGGTTTGATAGACTGATG
CACATGAATGCTTCTAGGAGGTGTTCTGTTTCAACAtccagtaattaaaaaaaaaaccc
>NC_000001.11:87334829-87335019
GCTGCCTTGCTGCAGCGAATCCGCACAATTAAAAGCTTTAATTAGTGGCTCCTCCATTTTCTTAGTTC
TGATGGGCTTTATCTAATGAGATCTGGTCTCTGGCTTAGATCTGCTCCGAATGACGTGTCCGCAATG
AATGAGAGCTGCGTCGCaaacaaaacatttaattaaCAAAATTGGCCTctaagag
>NC_000001.11:87335671-87336037
GAGTTCATTTCCCTGGTAATCAACAGCAAGCTGCTATATTCAGAGAATGCTGTCCCTTTAATTGAACA
GGCTAGGTAATTAAATGGCACTTTTCCAATAGGACGTGCTAGGTAAATCTAATAGTTGGTTATTTAATA
TCACTTTGAAGTCTGCCCACTCAAGCACAATGACAGCACAGGAGCATGTGAACTattagctagaaaaaaaa
aaaaacaggatctcaaaaattaattaaatcgCATGCAATTTAGGGGGAACGTTTATCTTGATTTGTCATAACAGAAT
TAATTCAAGGCCTCCAATTCACTTGGGGGCAGATCTGTTACTCTGAATTAACCAAGCGTaatttggctgatttt
tttttt
>NC_000001.11:87336109-87336216
TCCGAAGTACCTTTAATGGACTTGGCTCCAGGCATAATTTCTGGGTTGCATTTCTTTGTTACATTTAAT
ATAGCTGGTGCAGAATTTAGATTAAATATAGGAACCTG
>NC_000001.11:87336649-87336815
TATAAGGAACCTCTTGATGTTTGTGATTGATTGCATTGCCCAAATGCTGAAATATTAACGACCTTCTTG
GACTGAGGACCCAAAGAAAGGAAACTGAAACCGATTCTGTCATCACAATTAAAGACTCCCCTGGCTT
TAATTAGCCTGACCTGGGGTATATCTCCCC
>NC_000001.11:87337926-87338231
TCTGTAACTGTTCTGATTCTGCAAGAACGTGTCAATGTGTCGTGGATCTCAGACTAATtagttttgaaatgga
gttttgatTGAACTCTTCAAATCGATGCTGCTGCAAAATTTGTTTCTCGGGCTTCCTATTAAAAGCCATCTT
AAGGGGCAGTTTTACTACTCTCCCTGTCGTTCAGTCAATACATTTAATAACAACTTACAGGCTATTTTC
AATAAAGTGGCGACAGCAAATTAGTAGTTTGAACATGACAAGCCTCTTCTGCAAGACCAGATTCTGAA
AACtcaaagcaattatttttata
>NC_000001.11:87338679-87339203
tccttctcccccctcctccccccatGGCAGATGTTGTGGTCTCATTTGATTGCATAGGAAAACTGCAGTGATACAGC
AAACACCCAGAGAGATATGATGACAAATGGGTCCAGATCCCGGTAAATTACTTTCTGCTCAAATCGAA
ATTTCATTCAGTTTGTTGCTAGCAGAGATGAAGTAATCTAAATTGTGGACTCAGGAGCAGATAGAGGG
AAGTTGGAGCAAGCATTTCATTatcaagagagagagagaaggttaGGATGAAAGAGATGAGCAGAGGCAAATCTT
AAGTGTTGACACCATGATTGAAAAGGTTAACCCATACACATTATATATCAACCTGGATAGCAGAGAGT
TTCTAATGGAAAGTCTGCACTGATGGAGGTTTACGGGAGTTTCAATACACTGAGCATGATGTCTTCTT
AGATATACAATCAAATCCTCTTAACCCTTTTGATGACTCATATCTGAAGATATGATTAAAGTACAAAAG
AGTTCTTCATATAATTTCAAGGACACAATGCATT
>NC_000001.11:87340061-87340216
ACCTGCCGGAATCGCCTGGTCCCGGGAGATCGGTTTCACTACATCAATGGCAGTTTATTTTGTGAAC
ATGATAGACCTACAGCTCTCATCAATGGCCATTTGAATTCACTTCAGAGCAATCCACTACTGCCAGAC
CAGAAGGTGAATACCCAGCA

Homo sapiens chromosome 1, GRCh38.p14 Primary Assembly

NCBI Reference Sequence: NC_000001.11

GenBank FASTA PopSet

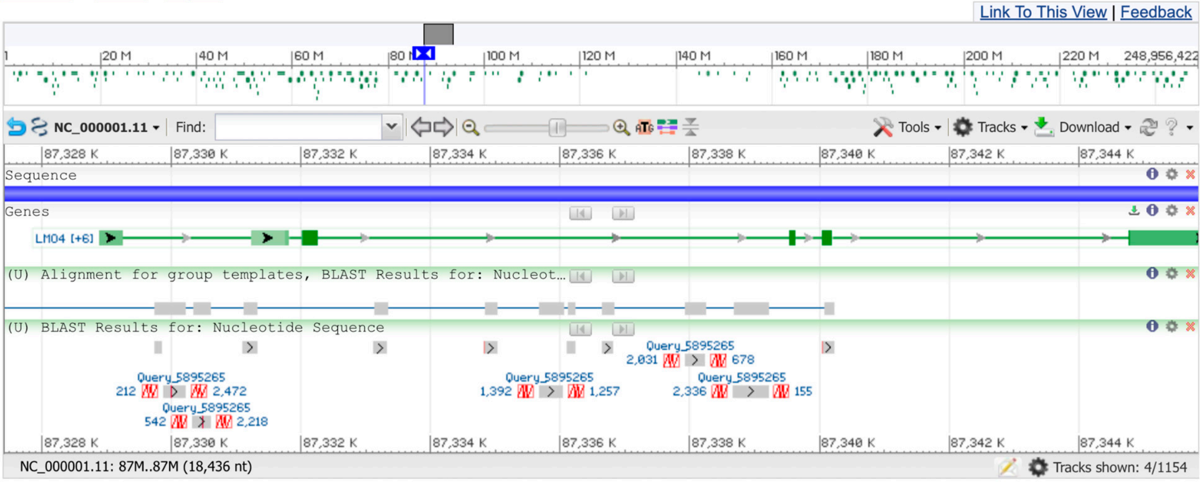

Homo sapiens chromosome 1, GRCh38.p14 Primary Assembly

NCBI Reference Sequence: NC_000001.11

GenBank FASTA PopSet

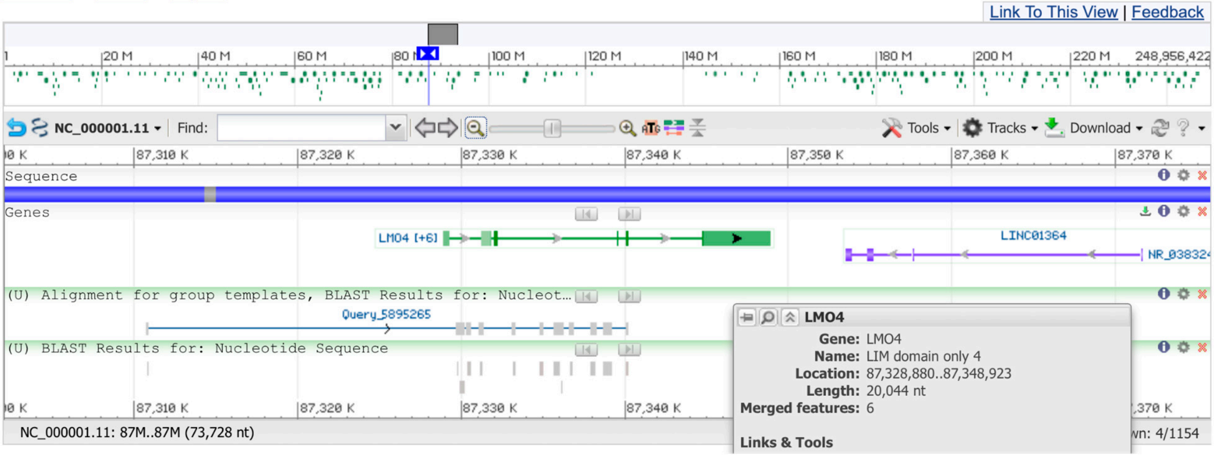

chr1 CLUSTER 4 positions on BED file: 772 - 782

>NC_000001.11:90828881-90829160
GTAGACATGGAGTCAAGTAGACTAGATTATTAGTGTAGTCATTGTGCCTTTACAAAATCCTGAAATTA
ATGAAACGTGCAAAAGAATCTGCAGTTCTAATTGCACAGGTGTTGCCTTATGGCAAAGCTTTATCATC
TGATTACACCAAGGGGTTATTAGTGACTTTTTATATACAGGATGAGCCTATGAAATGTACCTTAGCAA
CAGCTCATTAGTTTAGGGTTGTTACTGTATTATTTATTACAGTCAGCCTTCCCCATTATCTAGATTGAT
TCATGG

>NC_000001.11:90832099-90832329
GAGCAGCACTGTGCTACTCCGTTTATTTTTCTGACAGTAATTTAGTGCTTGCTCTTTCCCACACTTGT
CATTCCGTGTTTTTCCTCACATTAGAAGCCTGTAGAAAAAGGACAGTAATTAACGGTGCAATACATAC
TCATGGAAGCTCTTTCATCATATTGTGATAATTGTGCTCCTTGTCTGTCAAGGTAACCTCTCAGTTTCT

AAGCTTCATGCAGCATTATTTGTCA
>NC_000001.11:90833727-90834344
cgcAGAGATTATATCAGTAACTGCTAAGAAGGTCATGATAAAATGAAAAGAGTAAACCAGGGATACAG
CTGGGGACTTTCCTGTTCTTTTCCTTCCCCTGATTAGCAAATCAAAGCTGCCTGAATGATTCACACTT
TTCACCTGATTTTTGTCCTTTTCACTAATCATGTAGCAATTTTCTCTTGACTGACAGTTTTCTTTATAAT
TGGCCCTGATCTAGTAGGGTTCTAATAACCAGAAAATCCCCTCTGTGGGTCCAAAGGCTCTGGCGAA
TCgtctctcttctttccctgggCCATCTGTGGGCTGTGGCAAGCCACGTTTGTGGGAATAGAAGCTGCCTAATCT
AAATGTTCAATAATGGTGTGAATATTCTATGAGGAGTGGAGTTAAGGACTTGAGGTAGCAGATTCTAA
TTACTGTTCATTTGCCAGAGTATCAAAGCCTAATTACTGTTCATTTTATGCTCCTGAGGAAAGGCGAA
GTCAAAGCAGGGTTGTGCAGCCGCCCCCAGAGATCCCCAGTAAGCCTTTATCTGTCTTTCTGTTTAA
ATAGGTTGAGTGTTAGTATGTTAAATTACATCCTAGAAGCAAACAGATTCCACAAACAAACACCGT
>NC_000001.11:90834937-90835367
GCTGGCTGCAGTGTGAATTTACAAGCTGCAGACAACAACCTAATGAGGGAGCCAAGCTGTTAGGCG
GGCTGCTCGCTCCATGATGAGATGCTAAATGAAAAGCTGGTGCGACACACGCAGTTTGTTATCATTC
AATCTGCTTGAAACCGCAAACTGGTCATTATCCAGCCATTATTTTAGTTGATTTACAATCGCAGTGGA
TGAAGCAATTGCTGTGGCTTGTTCAATCCCCTGGCGGGAAGATGGGAACCAGCTTGTAATTTGCATT
TACACGATCTGACTGAGCAGCTTATTAGATctggatatttattttccacttaaCCGATGTTTGATTACACTTTAATAA
AGTACAAGAAAACTGTCTGGCTGCCTGCGTCTGATTGATCCTGCCGGGTGGTTTCTCTGGGATTTTT
CGTAATGAAACAAGAGA
>NC_000001.11:90840391-90840514
TCTGAAGGTGGCACATTTATGCTTCCAGGAACAGATGGGGACACGCATTTTCCTTGCAAATACTTTTG
AAATTGCTCTACTGGTAGCAGTATAAAAATGATATACAGATGCCAGGGCCGGCAT
>NC_000001.11:90840561-90840686
GGCTGTCAGTTTTGATTGATCTCTACTATACCTCAGGAACTTGTTGACTAGGTAGGGAAACAGTGCAA
ATAATTCACTGCAGCAAGTCAGATAATTCACACAACCAATCGGTCTGCTTCACTTTC
>NC_000001.11:90841087-90841370
GATCCGTGCTTGAAAGGACCTGATACATGCAGAAAAGCAAGATTTGCTAAATGCTCTAATCTTCAATC
AAATCTTACATCAATACAGACAAAGTTTGGTAATGAGAGATTCAATGAAGCATGCGGGAGAAAGAATT
AATCCTCTTGGTCTAAACTGGCTTCAACTTTGAAGCTTGCTCCGAAGGAAAGACAGTTATCTAAATGC
TAAATAAGAAACTTATTGTACTTTTCATGGCATGCTAAtcttttgtctatttcatctctATTATGTGTATGCTCATAGA
>NC_000001.11:90851900-90852008
CTGGACTGAAACGATCAATTAGCAGCCCGAAACGCACTGTGAAGTAAATTGTCACACTCTCTATTACA
GGATGGTTATCATATATTTATAAGGTGTGTTTTCTGCAAT
>NC_000001.11:90854563-90854682
GTATAACGTACTTGTCTGCCCAATTAGCTAGGACAATTATTAAAATTCAGAGGCCTAATTATGTCTCAA
TTTAATGCTGGCAAACTTGCATACAGAGCAGTAATTAAaatcctctcttt
>NC_000001.11:90854737-90854838
AAACCAGTGAAGCGTATTGTGCAACATGACCCTGCATTGTTAAGTGTGTCAACATATTAACCTGAATT
CCCTTCAGCCCCCACATAATCACATTTGGTGGA
>NC_000001.11:90856670-90856877
CTTGCTCTGAGCCTCTTTGGATGGCAAACTGAAGTGCAAATCTGTGTTCTGACTTGTTCCCGTGGCT
GTCAGCATGTTTTCCTCTTGTCAGCAGCAATTGACTGGTCAGGCTCACCCCTGTACAGCAATTGTAA
CAGCTCATTGACAGACTAGTGACCCAGCTGGACCTGATAATGGAAAACTATTGATTTTTGTGGGCTC
CACGAA

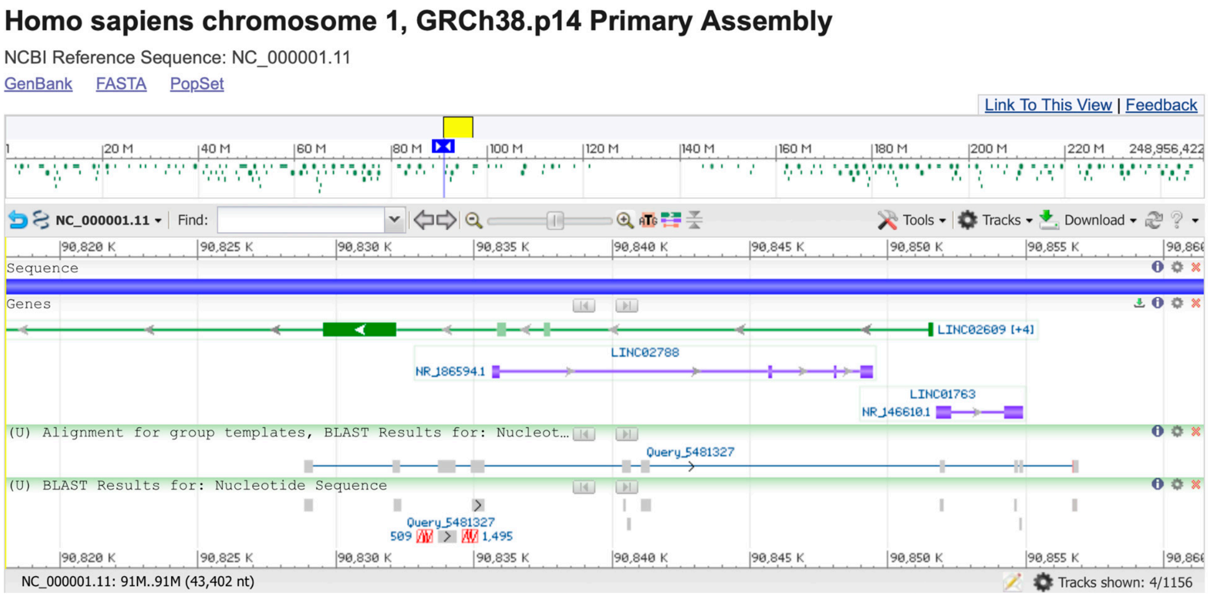

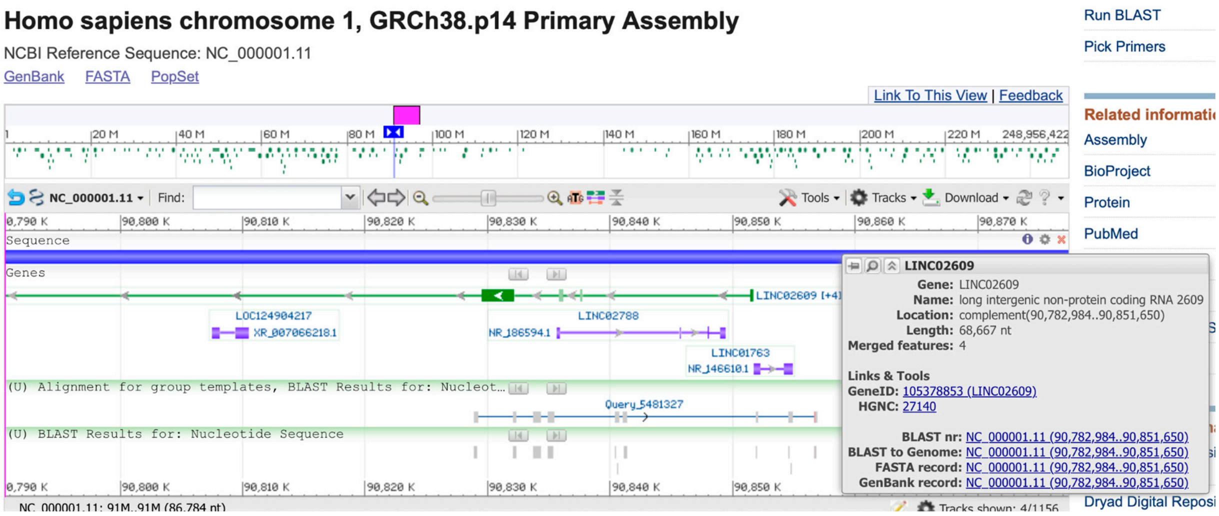

chr1 CLUSTER 5 positions on BED file: 1120 - 1130

>NC_000001.11:213994960-213995088
tttttacagGTTATTTCATGCTGAAATTATGCCTATTTGCATGGATAGTCATTCTTTAAAGCTAGCCACAGAT
GCAGTCCTAGGGAGCACGTAGATGTTTTTACAGGTGAACCGAAAGAGATGGGAGC
>NC_000001.11:213996257-213996654
ttttttttaatcttaatccATCTTAAACCAGTGgagcagaaatatttaaaaatgtttcatttcaagCAGAGTGCATAATAAATTGCA
ATAATTGTAATGTGCCATAAATCCCAGAGCCTATGCATTTTGCATTTGATTCAGGATTGAGGTCAGGA
AATTggagaaatttaaagaaaatgattcatCAGTCCTTTTGTTCTGTTGGCCAGGGTCCCGGGATTCTTGAGCTG
TGCCCAGCTGACGAGCTTTTGAAGATGGCACAATAACCGTCCAGTGATGCCTGACCATGACAGCACA
GCCCTCTTAAGCCGGCAAACCAAGAGGAGAAGAGTTGACATTGGAGTGAAAAGGACGGTAGGGACA
GCATCTGCATTTTTTGCTAAGGCAAGAGCAAC

>NC_000001.11:213996745-213996855
CGCAAGCTGCTGAAGAGGGCGAACTCGTATGAAGATGCCATGATGCCTTTTCCAGGAGCAACCATA
ATTTCCCAGCTGTTGAAAAATAACATGAACAAAAATGGTGGCAC
>NC_000001.11:213996892-213996993
GGCTCCGAAGTACATCAGGAGGATATATGCAGCAACTCTTCAAGAGACAGCCCCCCAGAGTGTCTTT
CCCCTTTTGGCAGGCCTACTATGAGCCAGTTTGA
>NC_000001.11:214002594-214002827
ccctTTACCTCCTTAATATTTATTTGtgctcattttctttcctggCCTTGAATGGAGCTTAGCTCGTGTTCAGTACAG
CTGTATGTTTACTGAATCTATTCCATCATGAGTCATTGTGCGTGTGTAAGTATCCTGGAAACAGCTAG
TGCTTTCTTGGAAGAACAGTTGCTTTTCAGCACAAGCACTTAAAAGGGAAATTAACCAATTGGTCAGT
TCAGATTTattttgaggag
>NC_000001.11:214004051-214004220
AAAATCCTTAGAGAAGCTTCTAGGAAGGGCCCTTAATTGACCTTGTGGGGGACCACATTGATTTTCTC
CACGTGCATCTTCATTTCTGATAAATTATAAAGCCATTAATTTGCTGAGGAAATGGCAGGGCCAGGCT
GCGGCACAGATGTGACCAGAGCCATCCCAGCTC
>NC_000001.11:214005725-214005895
TATGCAGTAGCTGCTTAAAAACAAAAGTGATGATTGTCTCTTATTTACAACTTAATTTGTTGTTGATGT
AGAGTACACTGAGCATAAGGAGAATGAATAAAGTGACAGATTCAGGACACATTATTCAAATGAGGATA
TGAAAGCTGTCGGCCTACAGCTGCAGCCTCCCT
>NC_000001.11:214009088-214009278
CAGTGTTAAATGATGTATCTCATTCCCTCCACCCCTTGAGTCAACTGCTGCCTAGCCAGATTAAGGTG
TCAGATTGATTTGTTTTATACATCTTTTGACCATGCTCATTGAATATTTAGGAAGTTTCTTCAGCCCATA
TTGAGGCTGAGATGTCCCGTGGGAAGCATTAATCAAAGTCACAGAGACTCGT
>NC_000001.11:214009745-214010098
ctattttttcctctacagTACTCTTGTCCATATGAGACAATGTCTTGTAACAATGCAGAAGCCTAATCTCCATGT
CAAAGCAATTTTCATTCCCCAGTGCACAGCCTGCTATCATTTTgtaatgttttgtttcttattctaaaagaattaaaaagg
aaCAGTAAGCCGTCACGGGGGCCTGTAGTCCTTATCTCAGTGTCTGGAAATTTGGACAGTGTATTTTA
CTGCTGagataaaatggaaagaactcCAAGTTCAGCAAATCGTAATGGGTTTAAGTTCTATTGAAATCGGCAA
CCAGAAGATCAGATAATGGGGGTCCTTCAGTTGTCTTTTTAATCGGGTTCCCCGC
>NC_000001.11:214010099-214010233
AGGCTGAATAGAGACAGAGCAGACACACAGAGTGAAAATATAATTCTTGGATAGGTTAAGTACATGTT
TGAACTCTTGCAAGCAGAAGCGATTTGCTGATGACTTAATCATTTTCTGGTCAATTATCTGTAAGG
>NC_000001.11:214017166-214017296
aaaaaaaaaagatgttttgtgGCAGCCTAGGGAGGGTCTCATCTTTCCTTCAGAAAATAGTTCAAGGCTCTTCT
GTCAAGCTTCCCTACTTAGAGCTTTTTCTCCTCCTGCTTCATAAAGTTTAAAGGGG

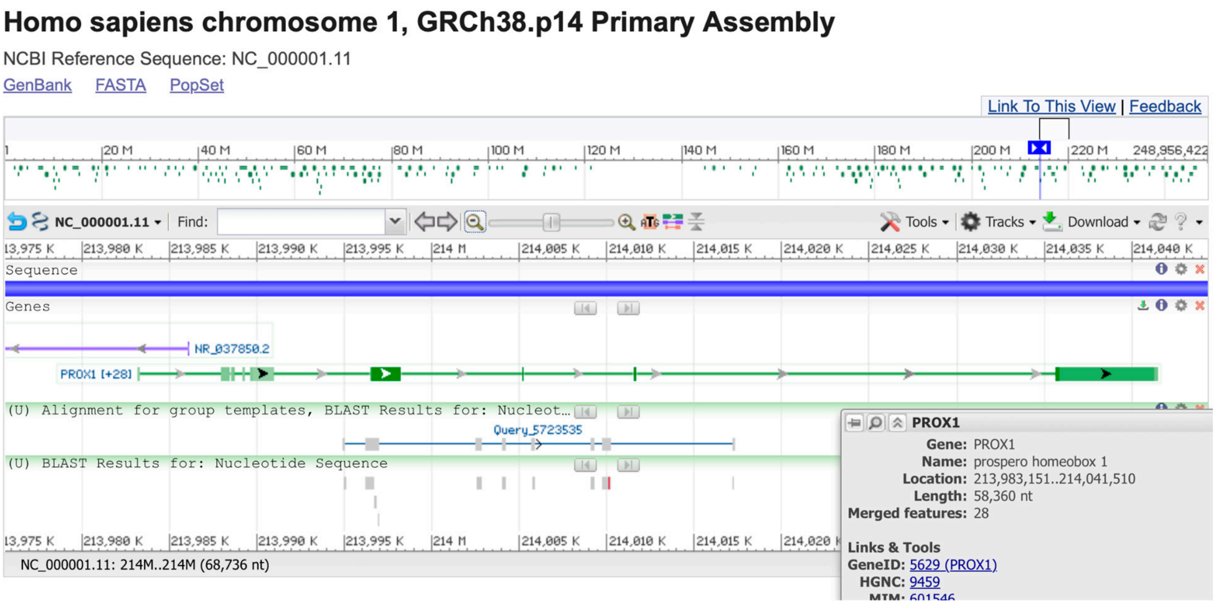

chr1 CLUSTER 6 positions on BED file: 1166 - 1177

>NC_000001.11:216503285-216503474
TCATCCTGAAAGGCATAATAATTTGAGTTACACATCTGAACTGATTGGAGTTACAGAATCATATCTCCT
TTTTGCATTTaacaatatatacaatataaaataaatacatttacacaAATGGACATTTGCTGGAGCACACAGTATGGTA
CACATCACAAAAATATACAATTGATTGCTTTACAGATGTGA

>NC_000001.11:216503556-216503794
ATAAgcagtttaaatttttgtccTTTTACGATCTTTGCACATAAGACTGCCATAAAATGTTTTCCTAGGCAGGTAA
ACAGAGAcgcttaaataattaaaatacaatcaCCAACAcccattttctcttctataagaaaaatagcagttttaaatttttatatcttttatgtt
ATCATTTAAATGCAAAATAGTGGAATAAATACAACAATCTGATCTGATTGAAACACAAGTGTAAC

>NC_000001.11:216504356-216504489
aagggatGTGCCAATTTCTGAACTCCTATCACAGCTATAAATTTCAAGTTATTGACCATCTGAATGAATTG
CTAATGATGATTTATCTAGAATCACACTACAGTCACTTCTCTACTGAGAAACCACAATCTAC

>NC_000001.11:216504895-216505008
TAAGGAATAGAATATACTACTGTAGTCTCCTGAGGAATTGTACATCCAATATTGTCTCTAATTCTACCC
TGTGTCTAAAAGCACACACCACAAGAATCCAAACACCACAAGAA

>NC_000001.11:216505094-216505195
tttgttaggATTACAATTTTAATTACATCTGTGTACATAGAATAAGACTGTTCACATAATACAGGGAGCAGC
TACTAACACTGGTCCATTGGTATAGCATT

>NC_000001.11:216505974-216506156
CAAAGCCAGCACAAAATTGCagtattcttatttctctttagtattgcatgaatgaataaagcTTAAACTATTCCCTGAAAAAGT
TACATAGTAGCTAAACTAACAAATACCTGGAAGACTTGAAAAAACAATTAAGGAATCAAATGGCTGTA
ACTGATCTAGATGGAAAAGCAATGATAAGA

>NC_000001.11:216517909-216518042
ACAAATAGGAAAGTACAACTCCATTGTTGAAAACAATTTCCCTCCTATCAGTCTAAGGATTCATGCAT
CAATATTGTAGATGAACTGTTAATtacaaaatcaattaaaaattatttctaaaaacagTCACGCG

>NC_000001.11:216518098-216518410
CCAAATCACAAAGTCGTCATCCACAAGTAAGCAAAAGCAAtctggtttttcatttttcagcGCGGCTGAGCCCAGC
CTGAGATTGATCGTCACATATGGCCCCAGAAAACAAACTGTCAATACCTTGAATGCTGCAGAATTTGA

ACAAATAGCCGTCCGCCCATTCAAGCCACTCATTGCATCCCATTTAACAGATTTTATTGACAGTTTCC
TTCTTGTAATTAAAGGGAAAACTACTGGCCGGCAACCAAGATAAAGTTCAAAGATAGGGTCAAAACAA
AAGCAGATGGAGGGGCACGGTGTGACTGTCAG
>NC_000001.11:216521613-216521787
TCTCCCTCCCAGACACTGCTGTTAGTAAATAGATTTACACATTCTCCAATCTCCACACATCTTTCTGCC
GAATAATTAAAAGCAGGATGATTAGTTTATTGAGTGGAAGGAGGAACTTTTTTATTGAGGTCCATCCA
CGATTTTATCAGGGCCAGCACTTTTACGGTTGTCAGG
>NC_000001.11:216521791-216521947
TGCAGGCATCCAATTTTTCTTATCTGCCTGATTAATACAAACGCTGTTTCAGGACACATTAATTAACAG
ATACAAATCTACGTTCTCATGGAAGGATCAATACGGAGAAGAAAAGAGGCATCAATGTGAATTTAGTG
CTGATGATGGAGAAGGCAC
>NC_000001.11:216532144-216532354
gctgcctgaTTCTAATACACCTAATTAGCCATCGGCAGACTCTTAATTGCATACATTAGGATGATGCCATA
GTGGAGATTAATTCTAAATATACCCAAGCAGCTGGTTAAGATGCCATGGATTACAGTGTGGACTGTAC
TTTTCCACTTAATTAGATATCAAAATTAAGCAGCAACAAGCATTTTGACATAACGGGGATCAAGCCGC
TGC
>NC_000001.11:216532371-216532531
CACTAAAGTGACCGCTAAACAGAGCTGCACAGATGGAGGGATATTAAATGCCGCACTGGAAagtaatttc
aaataataaaatatcaatatttacattttaattaccCCTACTGAGAGCTGCTCTGTCATTTTCACTATTGCCGACGCAGC
AAGGGGTAGT

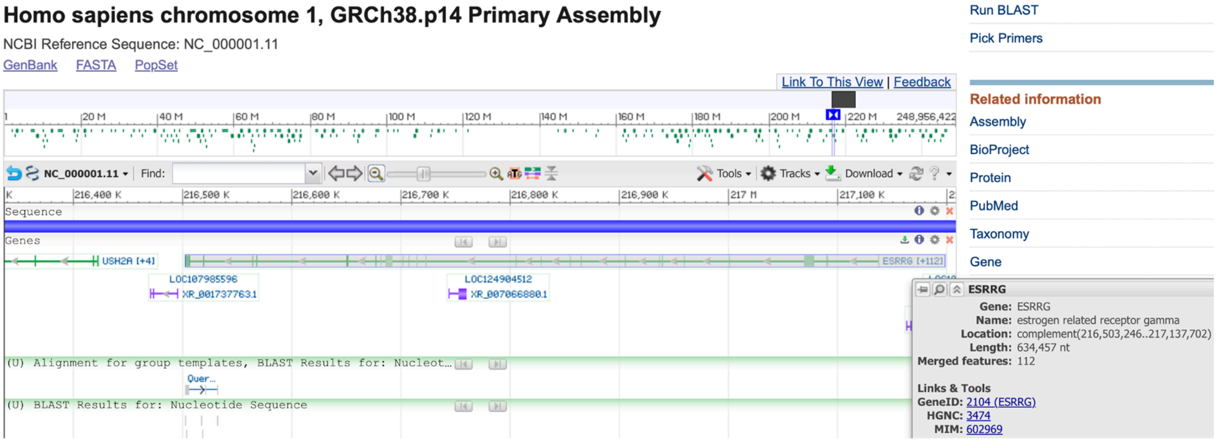

Homo sapiens chromosome 1, GRCh38.p14 Primary Assembly

NCBI Reference Sequence: NC_000001.11

GenBank FASTA PopSet

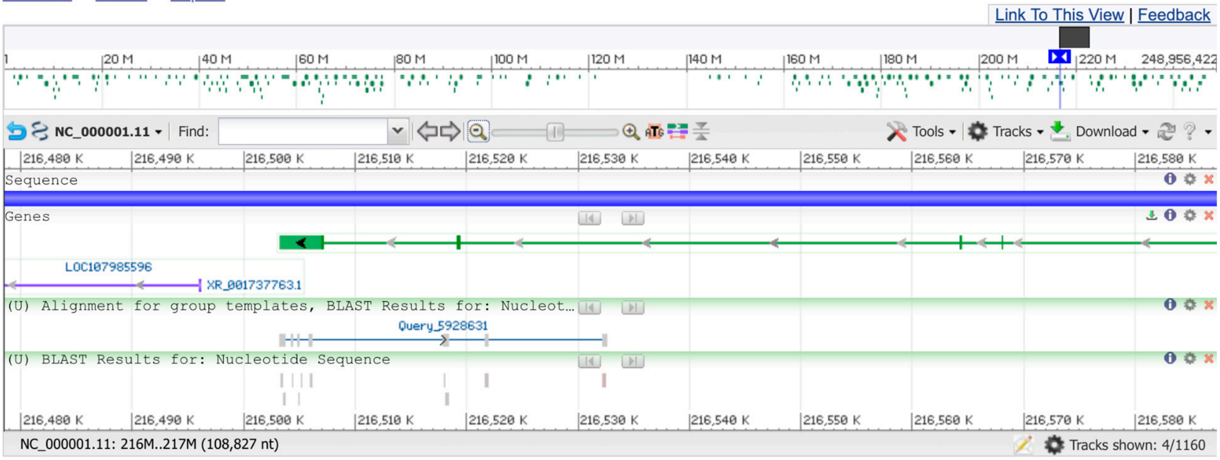

chr1 CLUSTER 7 positions on BED file: 1243 - 1256

>NC_000001.11:228610264-228610385
aaaagccTACAGCACCCGGTATTCCCAGGcggtctcccatccaagtactaaccaggcccgaccctgcttagcttccgagatcagac
gagatcgggcgcgttcagggtggtatggccgtaga
>NC_000001.11:228612505-228612626
aaaagccTACAGCACCCGGTATTCCCAGGcggtctcccatccaagtactaaccaggcccgaccctgcttagcttccgagatcagac
gagatcgggcgcgttcagggtggtatggccgtaga
>NC_000001.11:228614746-228614867
aaaagccTACAGCACCCGGTATTCCCAGGcggtctcccatccaagtactaaccaggcccgaccctgcttagcttccgagatcagac
gagatcgggcgcgttcagggtggtatggccgtaga
>NC_000001.11:228616987-228617108
aaaagccTACAGCACCCGGTATTCCCAGGcggtctcccatccaagtactaaccaggcccgaccctgcttagcttccgagatcagac
gagatcgggcgcgttcagggtggtatggccgtaga
>NC_000001.11:228619228-228619349
aaaagccTACAGCACCCGGTATTCCCAGGcggtctcccatccaagtactaaccaggcccgaccctgcttagcttccgagatcagac
gagatcgggcgcgttcagggtggtatggccgtaga
>NC_000001.11:228621443-228621564
aaaagccTACAGCACCCGGTATTCCCAGGcggtctcccatccaagtactaaccaggcccgaccctgcttagcttccgagatcagac
gagatcgggcgcgttcagggtggtatggccgtaga
>NC_000001.11:228623663-228623784
aaaagccTACAGCACCCGGTATTCCCAGGcggtctcccatccaagtactaaccaggcccgaccctgcttagcttccgagatcagac
gagatcgggcgcgttcagggtggtatggccgtaga
>NC_000001.11:228625905-228626026
aaaagccTACAGCACCCGGTATTCCCAGGcggtctcccatccaagtactaaccaggcccgaccctgcttagcttccgagatcagac
gagatcgggcgcgttcagggtggtatggccgtaga
>NC_000001.11:228628144-228628265
aaaagccTACAGCACCCGGTATTCCCAGGCGGTCTCCcgtccaagtactaaccaggcccgaccctgcttagcttccgagatc
agacgagatcgggcgcgttcagggtggtatggccgtaga

>NC_000001.11:228630386-228630507
aaaagccTACAGCACCCGGTATTCCCAGGcggtctcccatccaagtactaaccaggcccgaccctgcttagcttccgagatcagac
gagatcgggcgcgttcagggtggtatggccgtaga
>NC_000001.11:228632627-228632748
aaaagccTACAGCACCCGGTATTCCCAGGcggtctcccatccaagtactaaccaggcccgaccctgcttagcttccgagatcagac
gagatcgggcgcgttcagggtggtatggccgtaga
>NC_000001.11:228634867-228634988
aaaagccTACAGCACCCGGTATTCCCAGGcggtctcccatccaagtactaaccaggcccgaccctgcttagcttccgagatcagac
gagatcgggcgcgttcagggtggtatggccgtaga
>NC_000001.11:228637092-228637213
aaaagccTACAGCACCCGGTATTCCCAGGcggtctcccatccaagtactaaccaggcccgaccctgcttagcttccgagatcagac
gagatcgggcgcgttcagggtggtatggccgtaga
>NC_000001.11:228639333-228639454
aaaagccTACAGCACCCGGTATTCCCAGGcggtctcccatccaagtactaaccaggcccgaccctgcttagcttccgagatcagac
gagatcgggcgcgttcagggtggtatggccgtaga

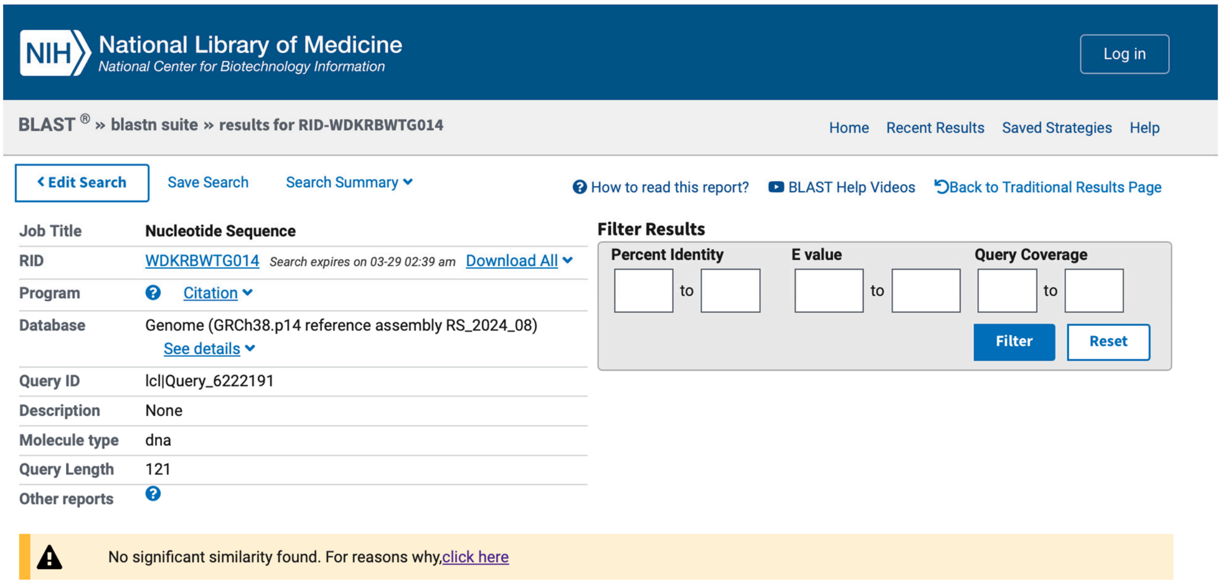

LOOKS LIKE REPEATS!

chr2 CLUSTER 8 positions on BED file: 1622 - 1632
>NC_000002.12:60440379-60440488
GTGTTCATTCTCTTACATTTAgattaattataaaacattgtcACCAGAATATTAAAGTAATGTTTAATACACAGAAG
GGAATAAGGTACTCTGGAAAATGCATAATTTC
>NC_000002.12:60440496-60440643
GATTTTGCCAGTTGCATGCAATATGATCTTTTGATTGCCAGCTATAGAAATATTGTGACTAAGCTTTCa
ctagctttttattttgcaaccATCCTGTCATAGCACTTTGAAAACTGTAAGCTTggcccatcaaaaaaaaaaaaaa
>NC_000002.12:60457088-60457189
AGAAATAATCCTGAAAAAGACTGACTGGCCTTCTCTTAAAACCTGTTATTTTCTTGCAAGTAATTTACA
GAGTGTGTCAAATGAGGACACAAGAAGCTTAC
>NC_000002.12:60457203-60457669
tttttttattttttcaataaagggACAAAATGGGTGTATGAACAGGTTAATGCAGACAACTGCCAAAAAAACACAGA
CAGTGGTTTTTCCAATAGAACTTAACAAAGACcagaaacaaatacaataaaaagccAGGTTGTAATGACCTTTG

GtcatctaaataaaaaaaaaaataaaaacaaagaaaaataaaagatcaaattAAGTGCCTCTGTTTTGAACAGGGCACATA
AGCAATAATAAATAGTGACTCCCAtagtaaaagataaaatttcaagTTACGACAAACAGCTTTCATTACAGGAAT
AGAAAAGGCCAATAACAAAATATTCTGCATTGCCATTTACAAAAAAGTATTGACTAAAGCGGGCTTTC
TCTTTAATATGCTTTGCATATGAAATTCTTTCCAATCTAAATATAAAGCACCATTTAGTTTTTGGCAAtga
aaaaaactgcaaaacattg
>NC_000002.12:60458033-60458277
tttttttttccactaccAAAAAAGGTACATTGATACCTTTTAAGAGAACAAGCAACAGTTAAAAATACAAGCTTCA
ATATAAATACTATAGTGCCTAACACTAGATGAACATTTAATTCAAATACCAttctagaaatacagaaaaaagaccA
TAAATGTATTTTAGCATAGGAATCAACATGAGTGTGCATTTTCCTATATTTAAGTACTATATAATCTTAa
acctttccccaatgtatgttt
>NC_000002.12:60458301-60458422
GTGTGTATCCAAGGCATAGAATTTCCACTaccatttttaaatggataacAAGTCTTGTAACACCACCAAGACAAT
GGAACCCTAAAATGCAGTTCCCCCCTAAACATAatgaagtgttttt
>NC_000002.12:60458652-60458794
ttttttttcagcattcTTGCAACTTTTCCCTTAAGTATAGACCTGTAAACTGGGAAAATTGTACAGTGCACTTAAT
TGTCCTATCTGAGCAGGTTTATTTTATACTCAACCTCTGTATCTCTGATTAGAGAAAAGATACAGA
>NC_000002.12:60460387-60460850
GGGTATTAATATACCTCTATTCAGTTTTTATATCATTATTCAACACTCGATCACTGtgccatttttcatgtgtttctcc
aGGGTACTGTACACGCTAAAAGGCATCTTACAAATTTCACATTTGTAAACGTCCTTCCCCACCTGGCC
ATGCGTTTTCATGTGCCTGGTGAGCTTGCTACTCTGGGCACAGGCATAGTTGCACAGCTCGCATTTA
TAAGGCCTTTCGCCCGTGTGGCTTCTCCTGTGGACAGTGAGATTGCTACAGTTCTTGAAGACTTTCC
CACAGTACTCACAAGTGTCGCTGCGTCTGCCCTCTTTTGAGCTGGGCCTGCCCGGGCCCGGACCAC
TAATATGGGGCGTGCTCCCTCCACTTCCCGTGCCGCTGCGCCCCGAGATCCCTCCGTCCAGCTCCC
CGGGCGGTGTGGAGAAGCGCAAACTCCCGTTCTCCGAGGAGTGCTCCGA
>NC_000002.12:60460982-60461162
GTCGAACTCCTTCTCGAGCTTGATGCGCTTAGAGAAGGGGCTCAGCGAGCTGGGGCTGCCCAGCA
GCAGCTTTTTGGACAGGCCCCCCGAGGCCGACTCGCCCGGGGAGCAGCCGCGGCCATTAACAGTG
CCATCGTCTATGCGGTCCGACTCGCCGGCCACCGAGTCTTCGTCGCAAGT
>NC_000002.12:60461820-60462152
tgcagaggagggaggggggGCGTCGCCAGGAAGGGCGGCTTGCTACCTGGCTGGAATGGTTGCAGTAACC
TTTGCATAGGGCTGGGCCGGCCTGGGGACAGCGGTGGGCTAGACGTGTTCCCTGCCAGCTCTCTA
AGTCTCCTAGAGAAATCCATGGCGGGAGGCTCCATAGCCATTGGATTCAACCGCAGCACCCTGTCA
AAGGCACTCGGGTGATGGGTGGCCAGGGCCATCTCTTCCGCCCCCAGGCGCTCTATGCGGTGGGG
GTCCAAGTGATGTCTCGGTGGTGGACTAAACAGGGGGGGAGTGGGTGGAAAGCGCCCTTCTGCCA
G
>NC_000002.12:60462171-60462462
ACTGATCCTGGTATTCTTAGCAGGTTAAAGGGGTTATTGTCTGCAATATGAATCCCATGGAGAGGTG
GCTGGGAAGGACATTCTGCACCTAGTCCTGAAGGGATACCAACCCGCGGGGTCAGGGGACTTCCGT
GTTCGCTTTCTAAGTAGATTCTTAATCCATGAGTGTTCTGTGCGTGTTGCAAGAGAAACCATGCACTG
GTGAATGGCTGTTTGCAAGTTGTACATGTGTAGCTGCTGGGCTCATCTTTACCTGCAAAATAATACAA
CACCAACATCAATGTTTAATCA

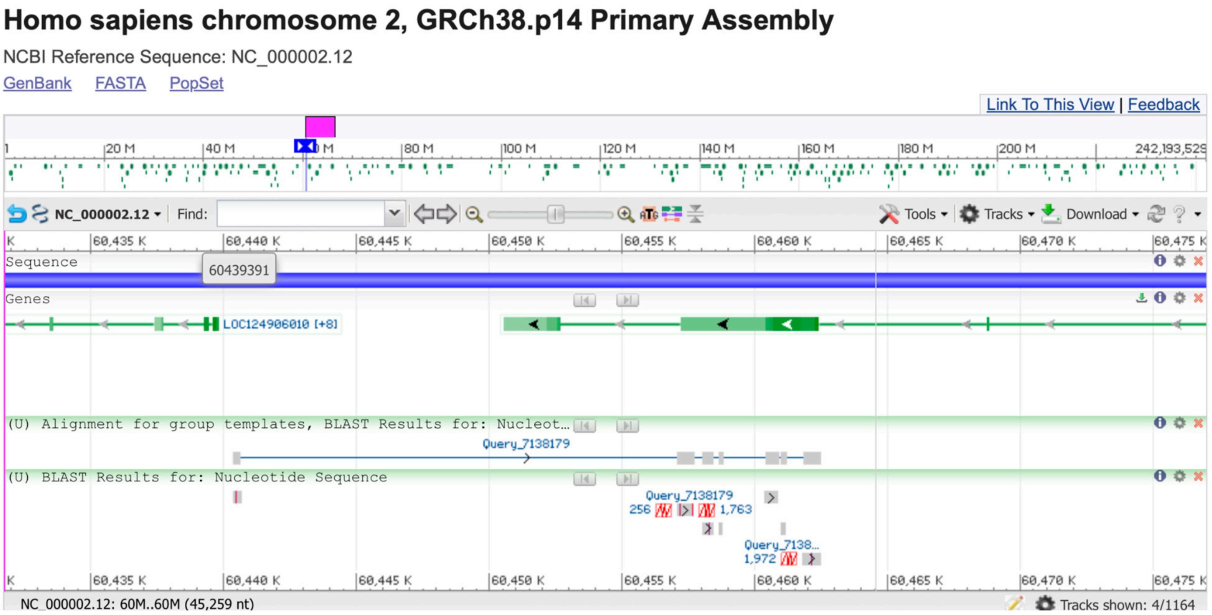

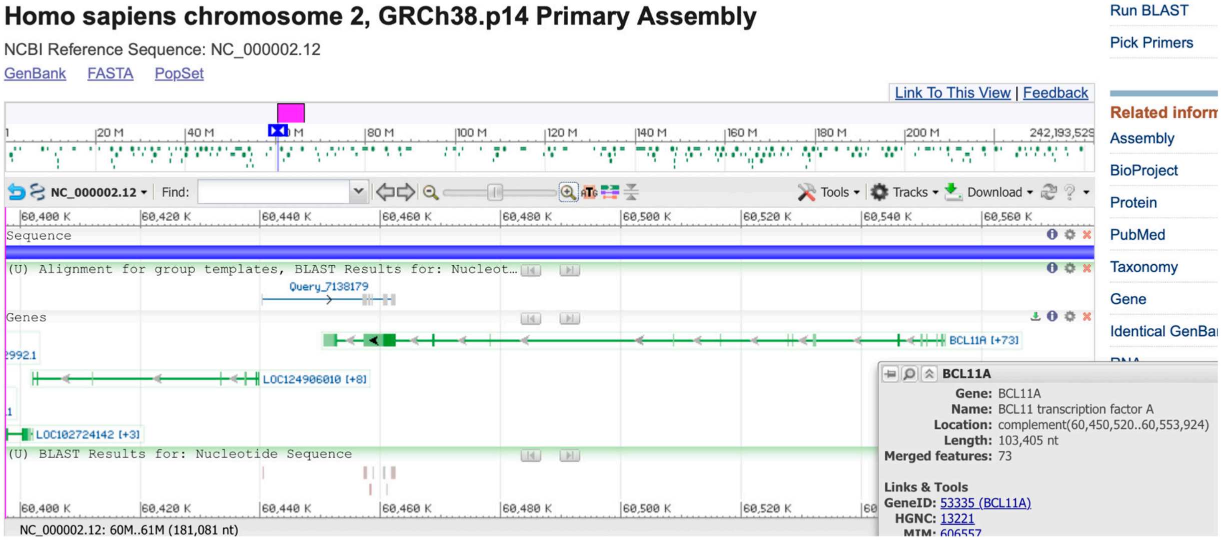

chr2 CLUSTER 9 positions on BED file: 1633 - 1644

>NC_000002.12:60533569-60533884
cCATTTTCAAATAGCAAATTATCCATTAGATTTCATCATATCTTAGTAAAGCAATTACATCAAGAAAATG
ATAGTGCGTGTGGAATCAATTATGCATGCAGTTGGCCGGAGACCAAAGGCTGTGAAGTCGGAGAGG
GCAGAATCGCTcgacacacacaaataaaaagccCCTGGATCTCACAGATGGACCCCATCAGTTCATCTTACT
GCCTAAAAAGTTCATGCacttaataatttatttgtgttCTGGATACTGTTTCCCAGCTGAATATTAACAACTTTAAA
CTGAAGCATGCTTTTAGCATGGTATGGATG

>NC_000002.12:60534756-60535335
TCTGAGGAGGAGAGCCACTGAGGCAGCTAATACACAAAACATGATCAAAAGTGATTAACAACAGCTT
CATATGCAAATTACATTAATGAAGGAGTGCAAAGTCATCATGTAAATTAAATATGTCAAATCTCTTGGT
GAACTTTTAATCTTGAGAAGAAAAAAcactgctttaatttttttacatcatCAATTTTGCAAGATTGAAAATCTAAGAA
TCTTGGTGCTATAAAcaattttcacctttttttctccttcagcagGCTGACAGGCCAGCCTGATGTGCACCGAATGTAC

ATGTTAATAGGCCAatcaaaaatgcaaaacaattcgCAATTACTGGAAGGACCTAATGGTCATTAGAATTTACA
ACTAGGTAATGAACTGAAGTCTGCCCACACCATGCATATTCATAAAGCAATTTAGCCTTTGAAGATTA
AAGAAGTGCTTAAAATTCAAATGAGCTTTAGCAAATTATCAGTAAGATTCATCCAAAAAGCAAAGGGT
TTTTCTTCCTatgatttcctcatttttttaatgcaaaattgTAATATCTTCCAGACTGAGCTTAAACTAAACAT
>NC_000002.12:60537028-60537170
gctaccAGTCACACAGCTCCAAGATCAGAAGAAATACATAGTCTTTGAAGCTGCTCAAATGTTTTAGTTA
AAAGGACAGCAGTTGTTTTCAAAGATCCAAGAGTATCTTTATCAGAATTAaatttttccttattgatttttt
>NC_000002.12:60541447-60541548
TCAATCATGCAATCACTGTGACAGTTCTATTTACAATTTGTCTTAGAAAGAGTTTCTCTGACATTGTAC
TTTGACAAAACATCCAAGCTGCCAGTTCACAA
>NC_000002.12:60545169-60545361
GAGAGCACAGAGAAGCATTTATCATTTAGCTGAGGAGGCAGGCTGCAGGGAGCAGCCGTTCCTGTC
AAACCAACCTTTTGAAATTTCCAACATGCCCTTAAACTTTTCGAGTAAGCATGTCTGTGCGCTTCAGC
AACTGCCCTCTCTACTCTGGCCTGGCCAGTCGAAAATGGCATCTTAAAGTCAAGGTCA
>NC_000002.12:60545914-60546214
CctctgaaaatgaaaagaaaacatgcaaacaGCTTTTCTCCTTGCTTCTCATTTACCTGCTATGTGTTCCTGTTTG
GGGCAAATTCCTCTAGATGACGTTGATAAACAATCGTCATCCTCTGGCGTGACCTGGATGCCAACCT
CCACGGGATTGGATGCTTTTTTCATCTCGATTGGTGAAGGGGAAGGTGGCTTATCCACAGCTTTTTC
TAAGCAGAGGCTGCCATTGCATTGTTTCCGTTTGTGCTCGATAAAAATAAGAATGTCCCCCAATGGG
AAGTTCATCTGGCACTGCCCACA
>NC_000002.12:60547568-60547773
ACTGAAGCAGAAAATGTACAAGTCTTAAACAGTTAATCAGTCTGACCTTCTTTAGCAAGCTGCCAATT
TTACATTTAATGAACTTAAAGCCACAGAGAatcaaaaaatagattttaatttgAGTGTAGAATATTGGAACAGATTT
TGATAAAGTGCAAAAATCCAGTGAACTcaggttaaatttatttcaaccCTACCCAGGCTG
>NC_000002.12:60548478-60548689
GACTGAACTGATTTACAATCAAAACCGGGAAATGAAGTGGTAATTCAGAGGTTGCTGATTACACCTCC
ACATTTCACCTAATTAATTTTATAGGAGGCAAAGTTCTTGAAAATAACAAGATGATCAAATGTACAGAT
ATAAAACCTGTAACCTCAACATTTTCTATGCTCTTAACGTAAATTATTGCTAATTAACAAGAATTATATT
CTAA
>NC_000002.12:60551885-60552042
GCGCTCGGTCCTCTGTCTGTTTGTTGGCAGGAGGCTCCCGCACACGCGGTGCTTGTAAACATTCAG
ACACGAGATTTTTCCCAATCAAAATCCACTTCCACTTTTTTTGaaaatcttccaaaaaatagTAAGAGGAGGGa
gttcctctctccctctc
>NC_000002.12:60553159-60553291
TCCTGCGCGCTCTCgtgattattaataattattattactattattgggTTACTTACGCGAGAATTCCCGTTTGCTTAAGTG
CTGGGGTTTGCCTTGCTTGCGGCGAGACATGGTGGGCTgcggggcgggcgg
>NC_000002.12:60553295-60553481
ggcggcggcggcggcggcgggcggacGACGGCTCGGTTCACATCGGGAGAGCCGGGTTAGAAAGAAGGAGA
CtccagagaaaatatcttcatcagTGCCTTTTGACAtccaaaataaattagaaataatacaaagatgGCGCAGGGAAGATGAA
TTGTGGGAGAGCCGTCATGgcttttttttaag
>NC_000002.12:60553546-60553806
gagagagagagatgaaaaaaatggcaaaagccCCCCTGAGCTGCAAGTTCAAGTGCGGACGTGACGTCCCTGC
GAACTTGAACGTCAGGAGTCTGGATGGACAGAGACACACAAAACATGGGCAGGGCGagcaggagagaa
ggggaggagggaaggggaagctCACACCAATGGACACACATCAGGGGCTGGACATGAAAAAGAGACCAGGA
CAAGCCAATGGCCAGTGCGGGGAGGGGGAGGTGCGGGGCGGGGGGCTC

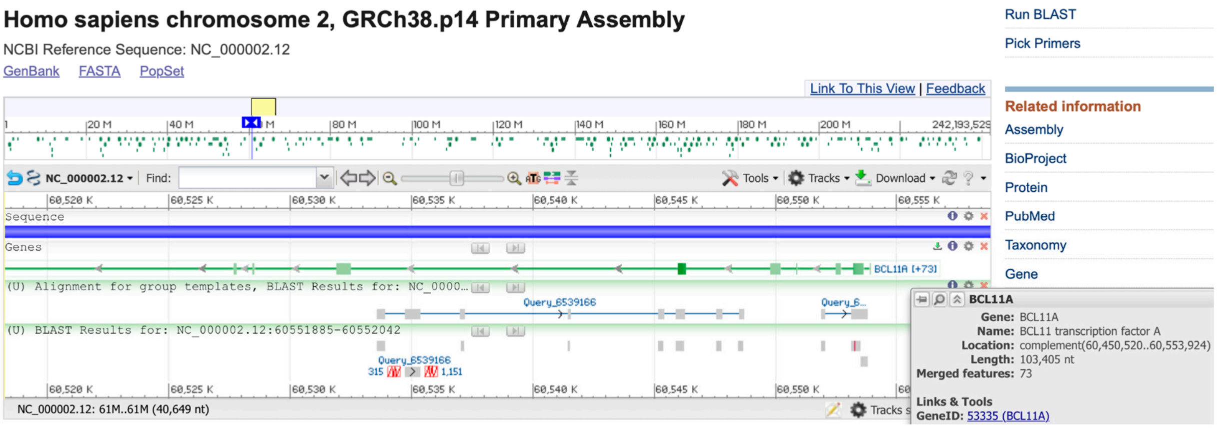

chr2 CLUSTER 10 positions on BED file: 1727 - 1737

>NC_000002.12:66507953-66508126
TAGACGAATTGTAGTACAAATTCCTTTCTTTGTTGGTTGGCCTCTTTCACTAGACCATGGCctataaaatttt
gtaaaaacaattcttaaatattatttgaaagggCAAATGGCCCACGCTGAAAGGTTTGACAGTTTGACATATAGTGCA
TGGTTTTGATAGCTCGGAAACT
>NC_000002.12:66508780-66508925
TGGCATTTCCTCATAGCATCAGCAAAACGAGCATAAATCACCCACTGGAGCCTACTGCTATCCTAATG
AGGCTGTAAGCTTGTTTATGGACTAGcatcatttttatgattatttccaCCTTTTCAGTTTGCCTTCATTTGGCGGC
>NC_000002.12:66509306-66509543
AAGGAGCAGGGTTTATTGTTTTAGGCTTTTCTGAACATGCACCAGTATTTACACTGGATTGAAAGCAT
TTGCTTCCAATGAAAATCTTGTCTTAGATTACAACCCAGCAGTGTAATTTCCCTCCCAGATGGGAGTA
AAGTAGCTGTATTTGCCTAAAAACAGTAGGAAGAACAAACTGTGCATTAAGCCAAGGCTGTTTTTGGC
TTGAGCTAATGATGTAATAGAAACCTTTTATTC
>NC_000002.12:66512053-66512318
TTgctcttttggttttgtttatatccAAAGGCATTCTATTTGAATAAAGCATGTCAAGGTAGCATCAGTCAAAATTTGA
TTCGCTATGTTTGCAGGTGATGGCTTGGACAACAGTGTAGCTTCCCCCAGCACAGGTGACGATGATG
ACCCTGATAAGGACAAAAAGCGTCACAAAAAGCGTGGCATCTTTCCCAAAGTAGCCACAAATATCAT
GAGGGCGTGGCTGTTCCAGCATCTAACAGTAAGTGGATTCTAAATGACATAT
>NC_000002.12:66517850-66517961
TATTGAAATCCTAATGGGATGGGTGATGAATTTTCTTAGGTCAGGTAAACTGAAAAGGCTACAGCTGG
AATTCTTCACCCAGAAGAATCGGAGTTAATACTAATGATCATT
>NC_000002.12:66521552-66521664
CTGATCATTTTAAAGCTAGTAATAACCAGAATCCTAAAATTCTtatgaataaaatgtaattttagtaATGTTTCCATA
AACAGTGGTAATGGTGGCATTTTAGTAGGCAGTG
>NC_000002.12:66522564-66522704
GTGTTTAATTGAACAAGGAATGAGTAGTAAATGAAAGCTGCTGCCTGAAATCCCACTGTGAGCATGTT
TTGCACTTGGCTCCCCAGTGAAAAGGCAATTTGTGAAATTATGTGATGGAGTAATTAGATGAAGCCA
GGGCT
>NC_000002.12:66522779-66523039
TGGCAAGTCGGCTCAAagcttcttcatttatttgtaaaCAGAATATTTAGTGACCGCATAGGAAGTAGCTGTAATC
ACCAGCTCATAAAGAAACCACAGCAGAGCTTTTGGATTTTTGCAGAGTGCTCATGTAAAATTTAAGCT
GGGAGGCAGAATCCCTGTTAATGTAATTAGGATCTAATTTACCACCCTTTGCACACAGATGTAATAAT
GTCTAGATATTTATTCTATCTTTAAGCATGTTACATATACCTCAGTGC

>NC_000002.12:66523137-66523299
GGAAAGAATGTCTGCAAATGAGGCAGTAATGGACAGTTGCCGTTTATGTACTGATGGATTTCTCAAT
GGTTAAGTAAATTAAAGCAAATGCTAGAAAAATCAATAGGCCAGCATAGTCTTGGTTTTGCCTCATAG
ACTTCAGGACAGAATGACAGATGTGGT
>NC_000002.12:66537109-66537296
TCCACGTCCCTGTAGATTTTAATGTCCCTACAGACTGCTGGCAGCCTGCTTCCAGCTGTGGCAGGCA
TGATGCAGTGAATTGCTTTTGAATGTTCTTTACTCAAATAGGCAGTAATTACAAGCTTAAAGAGGTTTA
GTGGTCCAGTAAACTACATAATCACCCCTGAATTAGCCTGGGCTCATCAGT
>NC_000002.12:66537314-66537418
AGTCTGCTGTTTGTATGCCGTCTTGATCACATAGCAACCTGATCAGCAGGTGTGGAGCAGCCAGAGT
CCCCCATAAACACAACTGTCTACTGGGGACAATGATC

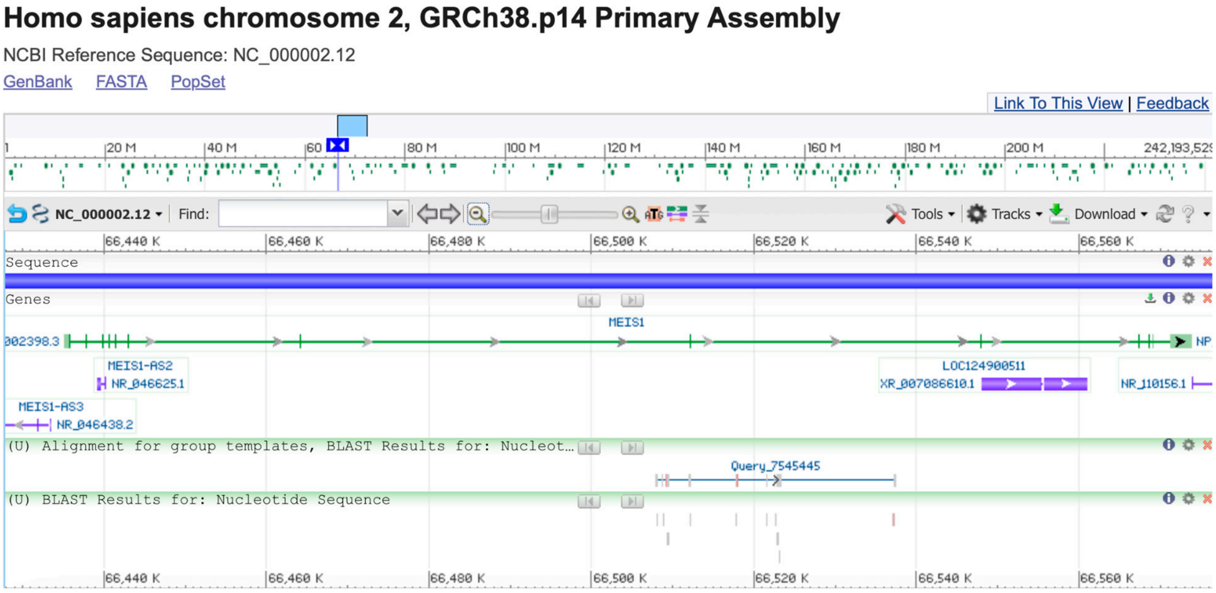

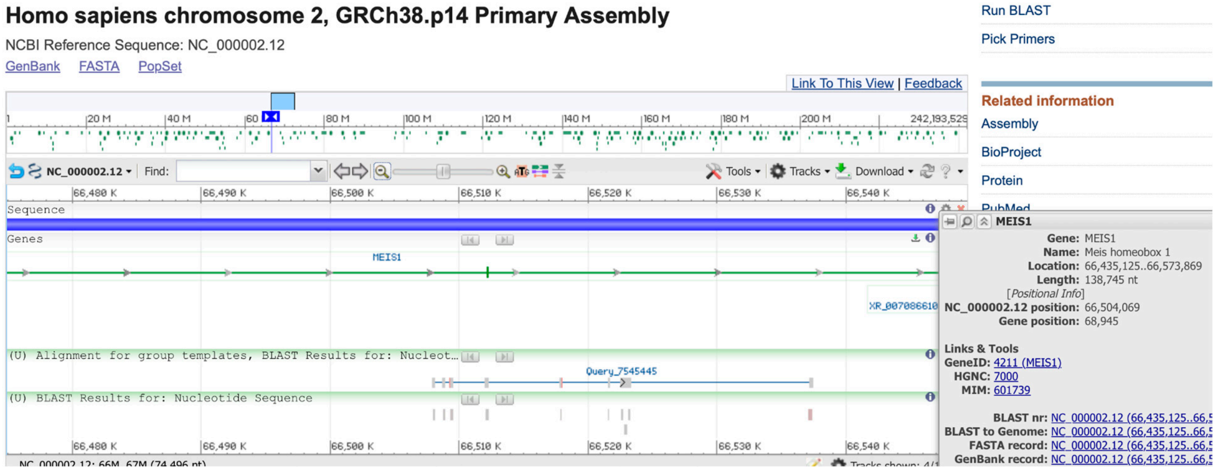

chr2 CLUSTER 11 positions on BED file: 1738 - 1752
>NC_000002.12:66549105-66549231
CCATTACAGAAAATGTAATTTCTGTCAATATAATCCCATGTATTCTTCAATTTAGTATTTTAGAGATAAC
AAGCTACTTACAATATATTTGCCTTTGGAGGATTAATTTACAAAGTGCACTATCTG
>NC_000002.12:66565932-66566076

gttATTTCACATGGTGTTTGTGCTACACCTGTGCTTATAATGAGAATGGAGAATTAATCTAACCTTCTGC
TCACATGATTCCAGTTTTCATGGTTTATACAAGATAATGATAACCTGATTTGCAACACAATTTGTTGTA
GAcct
>NC_000002.12:66567799-66567925
ctgagtttgccTTCCCAGCTCTTTCTGCTACTATGACCACTCCCTGGTGCATGGCTTGGTGTGGAATTGCT
GTAAACTTTATTACCTGTCAAAAGGGCAAAGGGGTCAGGATTGCTTGTGGGACTC
>NC_000002.12:66568998-66569263
TTGACTTTGCTCATTTTCTGGCCTCTTCTTGGATTTTTATCTCCCTTCTAGGACCTATGAGTGGAATGG
GCATGAATATGGGCATGGAGGGGCAGTGGCACTACATGTAACCTTCATCTAGTTAACCAATCGCAAA
GCAAGGGGGAAGTAAGTACAAATGGGGTCTTTGTTTTCACTTTGTCCTAGGAATATTTTTCCTCTTGC
ATTTTCTTATGTTCTCCTTGCCCATAGCTTCATGCttgttcattcctttccattaaacTCC
>NC_000002.12:66569358-66569569
TTGTACAGTACTGACCACATGACAAAACAAGAAGCAGTCAGGAGGTGGGGGAGTGGGTTGTCATAG
CAACAGACTGATTTGCAAAATGTAAGCAGTCTGCAGCAGTGCaaggagagggaagagcatgTCCCCAAAGT
GTCATAAATCTGTCTAACCGCAGTTGATGCATGAGTTACATTTCTACACTAACCTGCAAGACACCGAA
AAGCTA
>NC_000002.12:66569760-66569903
GGGCTTAGCCCAGGCTAATTGACTATATCCAAATTAAGTATGCCATCACTTGCAGTGTGACAAATGGA
TTTGACTTATTCAGTATACAAAAATAGAGATCATTAATGCAATCATCAGTGGCAGGCCTAGTGAAGTG
GGCCTAG
>NC_000002.12:66570228-66570485
CAGTCTTATTACCTCATCATGGAATTCTCTAGCTTAgttaatttaaatattgtttcttaGTTTCTGggtcaattaaatttaaatg
atgtATTTTATGCTTCGTGACCAATTAAATTACTAggttattacaaaaaaaattatcatctttTTTGATTAAAGAGCTGTG
GGTACAGTATATTTTATAAGCAATTTTCATTAGTTCAAAAATGTTCCTTTAGGCTAGATTAAGCAGCCA
TTCATTGTTAGAGCCTGGAGAC
>NC_000002.12:66570492-66570662
GAAGGTGTTCATCGTATTCACAGTGCACTATTACTTAGAACTAAAGCCAATTGAACCTACTTAGCAAT
AGCGTTATGCCTTTCACCCTTGATGATTATGGAGCTTATAGCTCTCAGAAACAATACACCTGTCAGTT
TCCATCAACTATAGCAATCCATGCAGAAGACAAG
>NC_000002.12:66571277-66571486
CGGGGTGGTCCAATGGGTGTGAGTATGGGACAGCCAAGTTATACCCAACCCCAGATGCCCCCCCAT
CCTGCTCAGCTGCGTCATGGGCCCCCCATGCATACGTACATTCCTGGACACCCTCACCACCCAACA
GTGATGATGCATGGAGGACCGCCCCACCCTGGAATGCCAATGTCAGCATCAAGCCCCACAGTTCTT
AATACAGGAGA
>NC_000002.12:66571684-66571974
GTCAATTTGGGGGACATGCTAAATAACTATATAAGACATTAAGAGAACAAAGAGTGAAATATTGTAAA
TGCTATTATACTGTTATCCATATTACGTTGtttcttatagatttttaaaaaaaatgtgaaatttttccACACTATGTGTGTTG
TTTCCATAGCTCTTCACTTCCTCCAGAAGCCTCCTTACATTAAAAAGCCTTACAGTTATCCTGCAAGG
GACAGGAAGGTCTGATTTGCAGGATTTTTAGAGCATTAAAATAACTATCAGGCAGAAGAATCTTTCTT
CT
>NC_000002.12:66572024-66572300
ctctctctccctctttctagCCTGGGGCTTGAATTTGCATGTCTAATTCATTTACTCACCATATTTGAATTGGCCTG
AACAGATGTAAATCGGGAAGGATGGGAAAAACTGCAGTCATCAACAATGATTAATCAGCTGTTGCAG
GCAGTGTCTTAAGGAGACTGGTAGGAGGAGGCATGGAAACCAAAAGGCCGTGTGTTTAGAAGCCTA
ATTGTCACATCAAGCATCATTGTCCCCATGCAACAACCACCACCTTATACATCACTTCCTGTTTTA
>NC_000002.12:66572301-66572652
GCAGCTCTAAAACATAGactgaagatttatttttaatatgttgacTTTATTTCTGAGCAAAGCATCGgtcatgtgtgtatttttttcat
agTCCCACCTTGGAGCATTTATGTAGACATTGTAAATAAATTTTGTGCAAAAAGGACTGGAAAAATGAA
CTGtattattgcaattttttttgtaaaagtagCAGTTTGGTATGAGTTGGCATGCATACAAGATTTACTAAGTGGGATA

AGCTAATTATACTTTTTGTTGTGGATAAACAAATGCTTGTTGATAGCCTTTTTCTATCAAGAAACCAAG
GAGCTAATTATTAATAACAATCATTGCACACTGAGTCTTAGCGTT
>NC_000002.12:66574607-66574853
GCATGATGTTCGACTGCTTTAGAAGCACAGGAAAGAGAAGTAAACGCGCTACAAATGGGGAATTACC
CTCGTTTAGCATTAAAATTCATTTAGATAAAATTGCCACAAACATTTAAATGGGAAGATTAGTTCCCCC
TCACGCCTTATTGCACTCGCTCAATGGTTCCGTTAAGAACATTTTACACGTTGGAAATTCCGTCTTCT
CAGACGGCTTGCTGTTTCCTAGTTACCCACATTCAAAGCAAA
>NC_000002.12:66575568-66575722
ATTATCCAGTGTCGGAACTGGCTCCCTTAATCTGATGCTTAAATATTTGATGTGGAAAAATTACCCATA
AAATTAGTTACTAACAATTTCAAATTGAAATCACTTATCGAATTATAAACGCATTAAAGCTGTATGGAT
GGTATTAGGAGTTCCT
>NC_000002.12:66578683-66578907
AAAAGCATGCTATTTCTGCACCGTCATTTATCACTGTCACCGCATAATGATTCCCCTTGCAGCCCCTT
ATTGATGTTTGTAATTGCATTATCTCATAAAGGAGGATGATCAATGAAACCGAGCCGTGCATTCTGGG
GTCAGAGGAAGCCAAAGTACTGTTTGTCCCCTTTAATACAACAAGTACTCATTATCTTTAGGTCTGCA
TTCAAAAAATGATCTGATCT

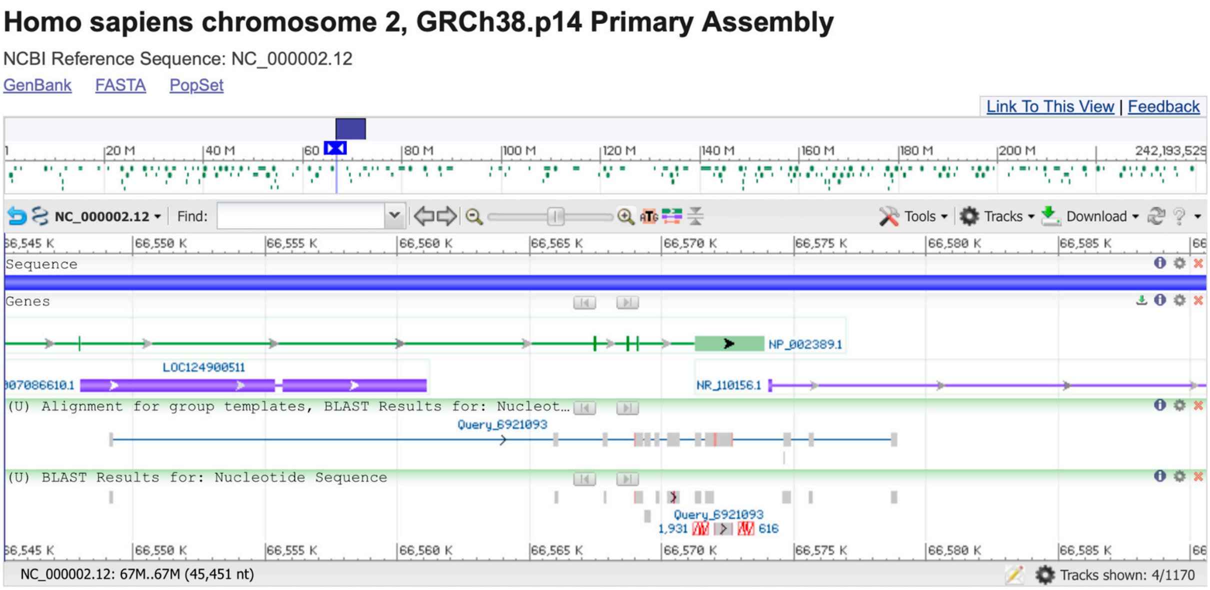

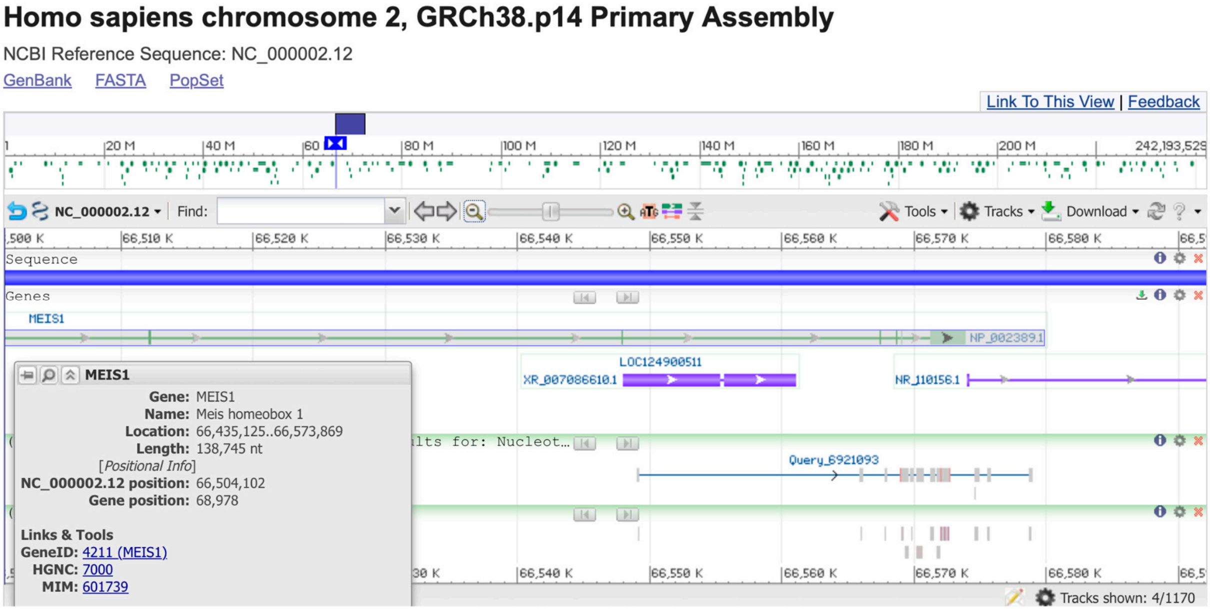

THE SAME GENE AS PREVIOUS CLUSTER

chr2 CLUSTER 12 positions on BED file: 1754 - 1764

>NC_000002.12:66671688-66671846
ATTTATACAATTAATTAATGTTTGCTTGACCactttttccaatttctttccGTAGCACATAAAAGAGAACCTGATAT
AAATTTCTCTTTCAAAACAATTGACTTAGCAACTGCTGACCTTCTGTTCTGTCATTCTAAGGATTGATT
GGATATTGTTG
>NC_000002.12:66679557-66679755
CTGCTGGTGTTTTCAGATTAAGATGGGGGATTAAGGAATTTTACTCAGCAATTTGAGATGCTTTGTTA
GGGACTGAGGAGATTTGAGCTGCTTTGTTCTCAAACCACATGTTAGTGAAAAAGCAAACAGGCACTT
AATCCTGACAGACAGCAGAACTTGACATCAATTTGCCCCTTGGGAGGTGACCAGGAGTTCTCT
>NC_000002.12:66688789-66689033
aaaaaatgcaatgaaaatttATGACCTTAACATTCCTTCAGGGTCAAGCAATTTATCAAATTCTCTCACTTTCTG
GTTTCAATTACACAGCCATTGTGGAAGGAACTTATTTTAGCAGGTTCATTTGCCTCAGTGGGGAATAC
AGGACTTTGGCCTAATGCTTCACCTTTAGTGCAGGCCATTTGAGCCTTAGAACAAATACCAATCATCA
CTCTTTGGGAAAAAAGAATCTGCATCGGCGTGG
>NC_000002.12:66689037-66689185
TTGGAATGGGGTTTCTGTCAAGGTAAACGGTTGTCGGCAATGACTGTGGTAGAGTGGCGATAAATCC
CATTTTATTGCTGAAAAATTCCAGCTTCTCTCCATGCCTGACAGCTCCCCACATTCTCATACCCATTTG
GGCAATTTCTGC
>NC_000002.12:66692055-66692173
tttttattgagacatcCTTTGAAAAATGGCATAATATAAATAGTTTTACAAAGCAACAATATAATCAATCTCTAA
ACTAGAATATAAATCAAGGATTTCATTAACTGCTGGTAGCCA
>NC_000002.12:66692174-66692389
TTTAAATTGTAGTCTTGTCCATaatgtctatctatctatgtatgtcCAAGTTATATTAAAAACCTTTAAATGGCTTTGT
AATATCAGCTACATCTAGCTATTATAATAATTAATGGTACTGTtttataaatcaatacatgtattCTTGATAAATTCA
TATCCAGTTAATggtgtaatttaaataaaaaattaatgtaataaagGACATACAGAAG
>NC_000002.12:66695397-66695750
GGAGCATTTTGCTGTCTTTGTCAAAGTGAATGACAACAATTTGGCCAACTCTCTCTGAGCTGCACTCA
ACAGTGACAGGCAAATTATCAGAGGCCCTGCACTTCTTATTATCAGAGGAGAAGCCATCAGGGGGAC

GCATGGAGGATACCTCTCCCTTGTCACTATTTGTCAGTGGGACAAGGGCCACTGCTATGGGTAGCTG
ACAGGTAATTTCAACAATAACAATTACTCCTATTACTGGTGTCATAAACAATTCGCCCAAGGCATGAC
AATAGCAAAGCTTTATAATTCAATGCCATATAAATAAATGCCAGCAGGTAACAAGTAATTTAGCTCCAA
TCAAGCTTTCACCC
>NC_000002.12:66698025-66698407
ACTTGATCATATAATGCAATCCAGCAGTAATCAATTTTACCTTCAAtatcttgcttttgttttcaaaatgctAAATAAAC
AATACATAATGCATCCCTCATCAGCCAAACCATGGAGGCTCTGTGCTCAAATAACAGGAAACATATTC
AATTTTCCTAACAGAAGACAGTTCATTAAGCTGTGCCACatcaaataaaactttaatttctCCAGCATCAGAGTCG
CAATGAAAGCAATTGAAGAAGATGAGCCATATGGTACTTCTATCAGCAAACACATATTGCTCATTCCC
CCAAAGTTTTCTAATTCTGCTCCCCAGACCTGTGTAATATAGATAATGTGCTCCGAGAGCAGCAACCA
TGAGCTATCTAGTGTACACTTC
>NC_000002.12:66699850-66700045
ATGACAATGGTCTATTGCAATGTAAAACACGCTGGAGGATAAAAAGGTGCTTTTTGTTACCATTTTAT
CAAAGCTGTTCATCTTCGAGCTGCAGGCAGCATTTTGCTGGAATTGCAGATATTTCTCTGTCCAGGCT
TCTTTGTTTGTGCATctcatttgcatatatttatctCCAGCTGAGGGTGCGTTCTGCTC
>NC_000002.12:66700159-66700326
GAGTCTCTTCCCCTATAAATGGGCACCATTTGTGTTAAACAGCCTCTTGTCATGATAGATGCCTCCAG
AGGTCAGGGGTTAAAGTTGATTTGAAGGTCAGCAGTAAAACAACAGACAAACCAGACGCCAAACTGG
TTCCTTAGCTGTCGGTGGGAGCTGTGGTACAA
>NC_000002.12:66700500-66700632
GCTGGGGAATTAAttccacttatttatttaagGCGTGTCAACTCACTGCCTAAACCTGTTTCAGTGTCAAGATGG
ATAAAACTTTTATGGCTCATAAAATAGAGCCATTCATCTCAATGTTCTTTGtggtgg

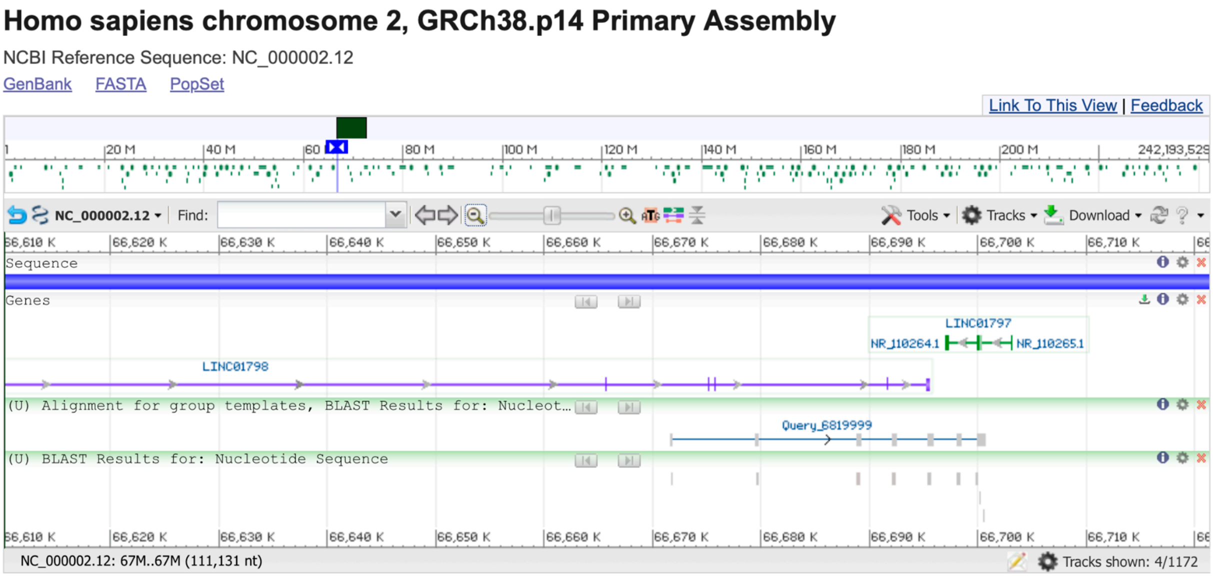

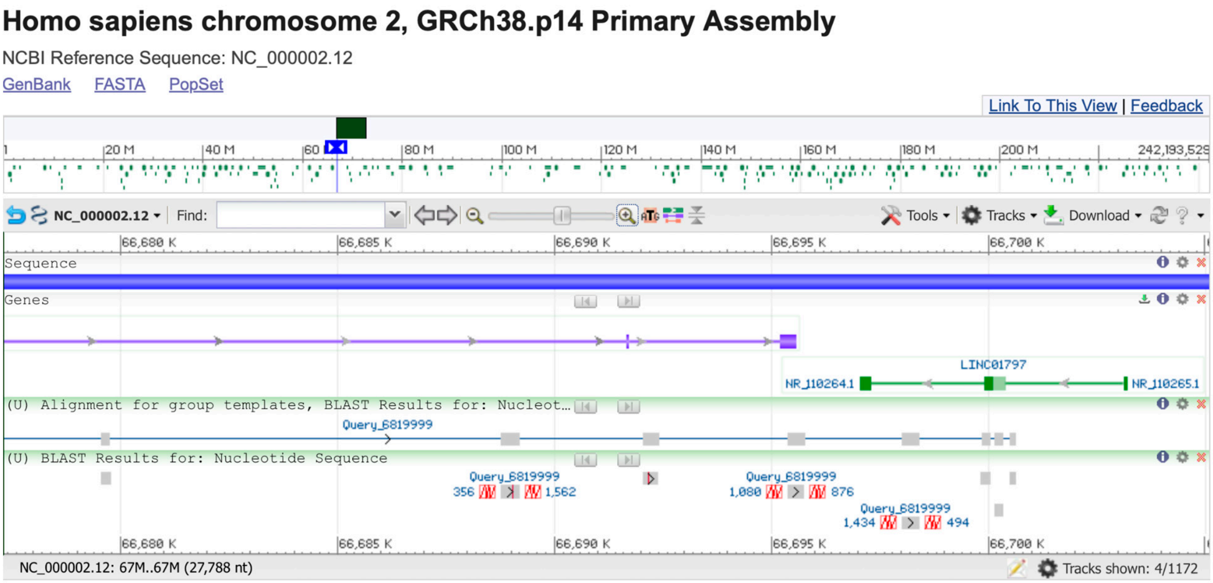

Intersect with 2 lincRNAs and overlaps with their two exons

chr2 CLUSTER 13 positions on BED file: 2044 - 2055
>NC_000002.12:144384614-144384776
tttaacatggatacaggaaaagaaaactctccaataaaaatattgtctaaaaagtttgttttgtctgcaTGATTTactaaatatgtacaatttcaaTT
CACAGCGAAGGTAACAAAGATTTAAACAGCCAACATCACAAATGTCTcaagttctaaaaaaaa
>NC_000002.12:144387770-144387920
TGAATAATATAACCtcacataaaaatacacaatctGGAATCAGGATCAGTTGAGAAAAGCTGTAAAATTGCTGT
ACATAggtatggaattttaaaaaacagttattGGTACCAGTCAAAATTATTGCTAaactaaaattatatgaaaaaa
>NC_000002.12:144387973-144388520
AAGGAAAACTCTCTAAAAGTACAGAACTCATTAACTACATTCTTAGTTTGGCTACATTTTTATTCGAGC
ATggtcattttcaaaagaaattacaaattgAAAATTCAATAATACTTTTACAAAGACAATTCAGGAAAGATTTTTGTC
ATGGTATCATACATTGTATTTAACAGTCCCTCTTTTTAGCTAAAAAACAAGATGAAGAAAGTGGAATTT
TCAGTCGAAAATACAGTGTTTTCACAAGATCGTATCAAAAGTTCCCAAATTTGGAATTTCCAgtaattggaa
aaaaacaaaaataaaataataaaattgaaaaatcccATCTCACAATTAATGTTCCAAAACACAATAAATGCTCTTCTC
TTTACGTAAAATTTGCCCAAATGATCAACGTCATGTTCctttttactaaaatatatctatatattgaaGAACTATAATAC
TGTACACTAcagtatgaaataaataaaattaggaaatataaaatgagccacataaataaaatgttatttgacctaaaattaaatgaatgcaaa
aaaaatt
>NC_000002.12:144388567-144388698
caaaaaagtacagaaaataattGCACAAACATATGTAAACTAAGGAACTGCTGCCTATTCTTCTAATACTGACA
TGGGTGCTTCAAAGAACAGGGTGAGCTTAACACTGAAGCTGGTGCAAAGGTAACCCA
>NC_000002.12:144388737-144388905
AAACACAGATTAAAAGGTTTGCATATAAGGCTTTTAAAACCACCTTACAAaggctttctttatttctcatctTACTTT
TTCCTTCACGTCCAGGTCACTTTAAGACTTCGGTATCTTAAATTCACACATGCATCACTTCAAGttccttca
cagaaaaaaaaaaaatt
>NC_000002.12:144389159-144389288
ATTTTCTAGTTGTGCTTAAAGTATAAATGCTATTAAACACAGGAATTAGTCTCTGAACCACACAGTTTT
TAGGCCTTAACACTGTACAAAATTTTTGCATAATGCAGTTTTTAACAATACCCAGCTCCA
>NC_000002.12:144390818-144390924

TGCCAAGCCTAAACACATCTGGTTGATTCCAGGCCACAAATAGCACTGGAATGCCTTCAACACCTTC
CATAGCTGCCATACTTGAAAGTTTATTTCCAAAatagaa
>NC_000002.12:144396376-144396529
GGAAGCTCTAACCAGTTAGGCAAAGTCACTCATACCTGTGTGTTCGTATTTATGTCGCAGAAGGGAA
CTGCTTTTCTGGAATGTCTTGTCACATAAGTCACATGCATACATGCCACTCTCTGTCTTCTTGATCTTT
TTGCGAGACAGACAGGA
>NC_000002.12:144402261-144402691
AGCAGCAATGACACAAAAGCCTTTTGCAAAAAGACAGCACAGAAACGGTATGACAACTGCTAGGGTG
TGCTGAATCTTCCCACATTCCTAGTGAGACAGATGGTGTTACATGGGACACAGGGAGGTTTGTTCAA
ACAGCAGCAACCTTCAAAGATCAAGCAGAGCCCAGCCCGCCGAGCGCCATTGAGGGACCAGCGCT
CATATGTTGCTGAGTTGCATAAACAAACAGCAACAGCTGCTTTGTCTCCCCCCACAGCCCTCAGAGG
ACTACTATAAACTTTAGTTGTGCCACTGTTAATATGATACAATCATTTTAATTTACTAATTGTAAATGCC
ATGAGCAAATGAGGGGACTTTTCAACACCAAAGCTACCATTCATAATGCGCCAACACAATCACACTTC
TGCATGTGAAATTTAAAGCGGTTATG
>NC_000002.12:144403563-144403906
CGAggtgaaaaatggaattaaaattgCTGAGATGTTtaataatgtataattaaaatgctacaatttcattcactttttgtggaacataaaaatga
aactaaagtCAAGTTAAAGtgcaaataaaatttctaaattagaAATTAACACACTCATTCGATCGTGAACATAAGACTA
TTAAATCATATTAGAAGAGACCATCAAAAAATCATTTAGACCTCAATTAAAGCTATTACCATTAAAAGA
TCTGCATCTTCAAAATGCCatgaaaaattaataatgattGCCAATCAAAGCAATATCGTTTCTCTAAGGGGTTAT
TATAGAAAGAAATCACTTAAAACCA
>NC_000002.12:144404009-144404150
TCATTCTTCTCGTGGCGGTACTTGATGTGCTCCTTCAGTGATGTCAAGCGCTTGTAGCCCCGGTCGC
AGTAGGGGCAGGTCAGCAGTTGGGCAAAAGCATCTGGAGTTCCAGGTGGCAGGTCTGTAGccgagag
acagaga
>NC_000002.12:144404669-144404806
AATTTCACAATACCCTAAAATTACAGTTCTAATCTTTGCTCATGAAATTCCATGCCTCTGCAGCTGTTC
TTCAAATTTTAAGGTAAACACCCAGGCATGTAGTGCATTTGTAATTGTAACAGCTGCAGAGGTCAGTG

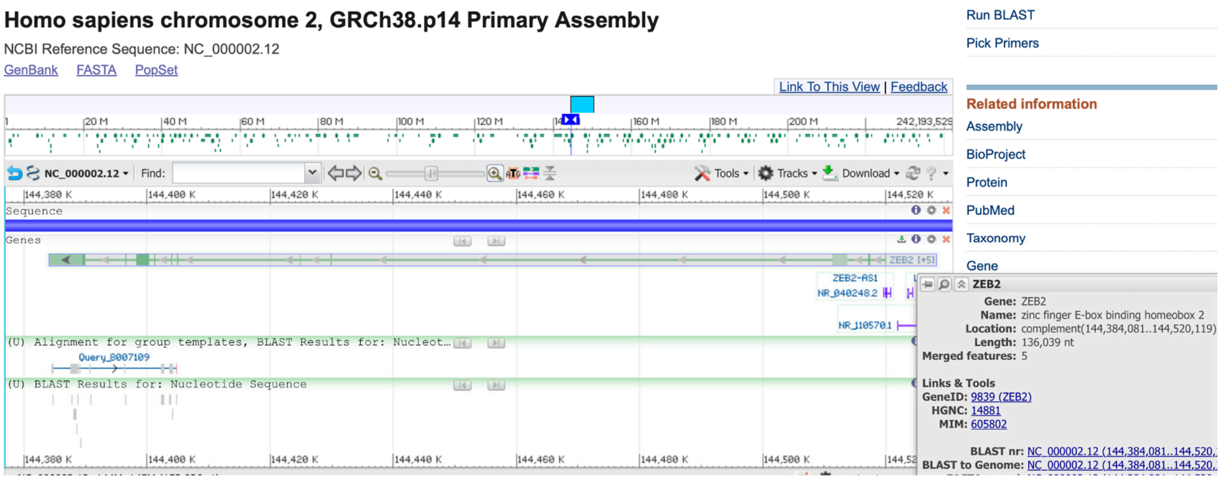

Homo sapiens chromosome 2, GRCh38.p14 Primary Assembly

NCBI Reference Sequence: NC_000002.12

GenBank FASTA PopSet

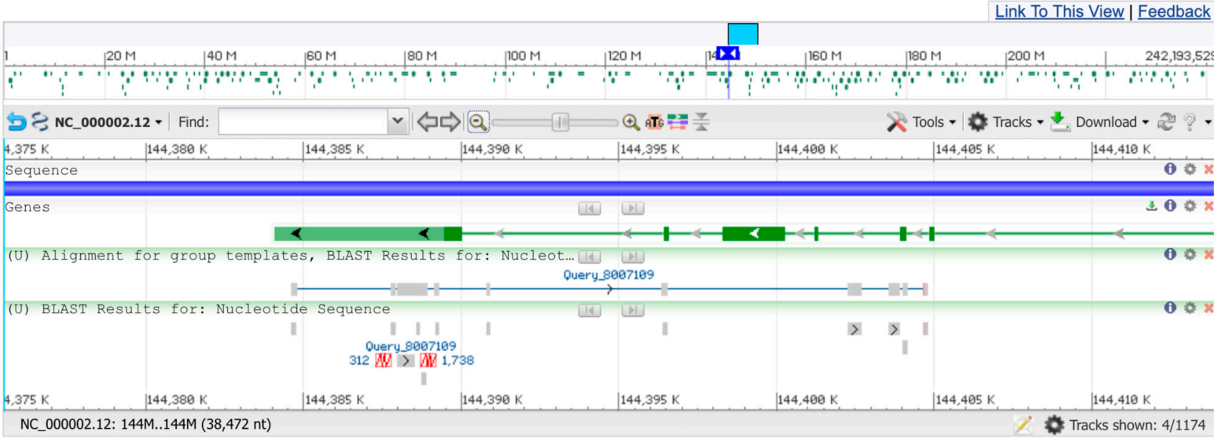

chr2 CLUSTER 14 positions on BED file: 2056 - 2072
>NC_000002.12:144427184-144427320
GAGGCCAAGATGCAGGTGATTTATTTAAGTCTGTGTCAATGATAATGATGTCATGGTTTCCAGCACAC
TGTGTCCACCCCATTGTGGACGAAAGAATGTCAAGGACAATTCCAAACACAAAACTTTGATTGTGCT
>NC_000002.12:144428094-144428548
ttattaattattCTTCTAAGGGTGGAAATTCTAGTAATTGGTCCTTACCCTTGATTTctagatattaaaatttaaagcatCA
GAACATTCATTCTTATAGCCCTGCATATCTAGTTCATTAACAAATATGAATAGCTAACACCAGCTCATT
TAAATAGTATGTCTTACATGAGTTCTCGCCACACTCTGTTTCTGTCACACTGCTCCCAAGAATCATTG
GCTCTTATTATACATGTAAATGgctatcataaaaataaaaatttcatttaagatTGTTAATGACTTCTGCAGCAGTCAG
ACTTCCTTTAATGATCATCCTCTGCCCCTAATTAAACGTATTTATCTTTCAAGCATCTTTGGCTGAGTT
CTCCCTCATAGTTGAAAATCTAAAACCTTAAGGCTTAATGGATATAACGATAAATGTTTATGTGTGGGA
TTGCTGAGATTCGAGAAA
>NC_000002.12:144430757-144431094
CTGCTTTTGCAGCCCTCAGTCCCAGCAATGCTCAGTAGCTTTTCTTAAAATAGACAGCCTGTAAATAA
GGTCTGTGAACTCAATTGAAGGTGGCTGTTTCTGAATTAGTCAGCCCTCACAGGCTCTCGGCCTACA
TGCTAGTACATAAATTGTCCACTTTACCACCAGACAAGAAAGATTAGAGTAATAAACACGGGGCATTA
GCTCAGCTAGAGAAACACACCAGCCGTTACGCACACACAGGATTGCCAAGAACTGTTAACCCAACTct
ccagaaacacacacaaaaaaacaagttAGAACCATGACATCATGGGaacaagagggagagagaaag
>NC_000002.12:144431253-144431744
aaaaaaatgaaaaggattgtGTGTCAGGTTCATAATGCTTTTGGAGAATTCTGGAAACATGTCTAGTTACAAAT
TTTAGTCAGTCATATCTGTTTGCTTTGACAGGTTCCCAGGGagtaaaaaggaacaaaaagagagagatttttttttttg
tcatgtgaAGCTGGGGACAAAAAGATTACACCGTGCATATCTGCTCATAATATGATCTTTGTATAATCCGC
GGGTCTGCACTTGCCTTCCGAACAACTGCACAACAggtgcaaattaaaatgaaaacggCAGCGGAGCCGGGC
AAACAGAATCTGCTGACCTGGTTTGAATACCAATTGACGGGGGAAACAAAACCTTTTCAAGAGGTGT
GACCACAAGGGCTTAGGCAAGTTCAGGCTGGGTAATGCCCTCagatctacaaaaagagagatattCTAACATC
TCTGCTCTAGCTGTGTGGTCCAGATATACCTTAGTACAATCAATaatgtgctt
>NC_000002.12:144431854-144432006
TACAACAAAAGCTCCTAAGGCCTGGGAAGCTTTCAACCCACCTCCTAAAAACTGATCTCTGGGCACA
GGAGGAATAAAGAAAGGTCCAGAAGGAGTGAATAAAATTATCTAAGGCTGGACAGGCATTAAAAGGC
Ctttatcctggaaaaaaa

>NC_000002.12:144432121-144432242
agagataaatgaTTTATGAACAGCCCTTGAGGAGTCCCAAATATCAGAACAATCTAAACAAGCAATGAAGT
CATGTCTCACCATATAGGGTCAGGAGTACAGCATCCATCACCTTCACAGG
>NC_000002.12:144433105-144433286
CACAAAGAAGTGGGAGCCCAAAGAATTCCTTTCTGCAATTCAATAAACAAGAGGAAAGATGTACTGTT
TGCTGGCTCAAAATAGACACTAATTTTACATCTGTCTCTGAGGCATAGCAAAAAGCCAACTAAAGAGC
AGTAATAAATGTCCAAAAACAGCATCAGTATCCAAAACTCTCACA
>NC_000002.12:144438885-144439133
TGCACCTCACAATTGACAAAACCAGACAACGGTGAAAGGGACAGTGACATGGCAGACACAAAAGCT
CAACACATTATGGCCACTCTAAACAGAGTACAAAAGGAAGGGAGTCAGTGATGTGTAAACCAAACAA
TGTGAGGggaaaaatattgaataatttctGTCATGAGGCACTGACTACAGCCAGGCTCTAATTGAATTAAACTCC
CATTTATTTGCTCAGGCCTAAACAACAGgctgggaggaag
>NC_000002.12:144439146-144439278
aaaaaaaacttaaggttTGCTCAACTGTTCTGAGGTACCACCTTTCCTCACCCCTGTATAAATCCACCATCTG
TGCTCACAAGCACACATGTTCCCTCTCTCAACAAATGCATGGAGACCCTACCAGGCACA
>NC_000002.12:144439319-144439907
GCCCCAGGTCTAAAGGAGCAAAAGGGGTAGAAAAGATCTTTTGCTCATTGCCCAGGTGAGACGGCC
TGGTGCGTCCTCTCAGCCTCGACACGCGTCAagtaaaagaaaggttaaaattagaaaattcaatttatttgatAATTTCCC
AGAATAATTAGAGTTAATATGGGTTAATTAATATGCAAATCTCTCAGCAGATGTTCTGCAGCAGGGCC
TGTGTGAGAAAACAGCCTTTGCTTTCTCCTACGTGATTTTGGCTGTCAGTTATTGGGTATAATTACTG
TAACTAACCGAATACACACAAAACCTTTGGAATATAAAATTGTTACAGTTCTAGCTCTGCACAAGCTG
CTACTTTTCTGCAATAAAAGCGAacaatttctttcagaaaataggagcctttttgttcctttcttgatttaaaaagaagaaatgaatcaaG
TAAATGGCTTATCTTTTTCTATGCATAATTAGGTCAGTATTATATGAACATTCCAATGAGCAGTGGTAC
AAACGTACATATAAAATGATAGCTCAAACCACCTGCAAAATcacataaaattgttttattcagaatcttttttttcc
>NC_000002.12:144443267-144443700
GAATCCACAAAGTTTTTTAGCTAAAGGGCCATATGccctattttaataaaaaggaatccataaaaaataaattactttcacTA
AGTATATGCAAATTCCAATGTAAGCAATCAGTGCAAAATTAATTTTGCCAGAACTGATTAAAAGCATTA
ATAAATCTATATGCAGTATTGCTTCAATCTGATACTGTAAATTTATTATACTTCCTGTAAGATTAATTAT
AATGCTACAATAACATATTACGATGCCAGAACATATTACTGTACATTCTGTTAATAACAGTTAAGCGTA
ACCTGAGTTGTAATTATGGACTGTAACAAAGTAATTTGATAGTGATTGTTTTCTTTAACTGTTAATGTTA
GATGggaaataaaatgtgtatgtgcttgtgttcattatattttctctttaatacaATACCTAATTAAAATC
>NC_000002.12:144444308-144444772
GTGGCAAACAGGTTTCTGGAGAAGTACCAAAAGAAATTATTCAAGTTAAGCTTTTTTTGGATATATTCC
TGATTAAGCctgaacacaatgaaaaaaacagtGTGAAGCACAATTCTGAATGGCATCTGTGCTTGGAGCTTCA
GAGGACTGCTGACTGTCATAAAACTCTGTGTGTCACCAGCTGGGGGTCGGGGATTTAAAGATGTCA
GGGGGACCTGGCCTCTCAGATGGGATAAAGATCAGAAGGAAAGCTGTCCAGGTGATGCCCAGGCC
CCTGAACAGGCCTATCTGCACTTTCATTCAAATGATAATGAGTGTGTAAATCACATCTGTTCCCCCTC
TGTGCCTGTATCCTTGTCACCTGAATAGCATCCCCACCAGGTATCAAATGAGCTACAGgaaataggttttcttt
ttatccaaCTTCACtctgaaatgagaaaatgagcACCCTGACAAGGAAG
>NC_000002.12:144445300-144445427
AAAGTGAGTAAGTGTGAATGCTTAGGTACCTGGGGTATGGAAAGGTGGTAATTAGCACAGCGGGATA
ATTGATGTGTAGATTGCAGCATGAACAAATGCTTTGACTTGTTTGTTCTTCTCACTCTTc
>NC_000002.12:144452433-144452810
CAGATATTATAGATAAAGTAAATACAGCATCACCACAAAAGGCTAAACACTTGTTAAACTGTCTGGTTT
TAGAACATTTCAGCCATACAACATTATTCAACTACTCATCACTGTGAGAACAGGCAATCAGGAAAGCT
AATTTCTTAATTAGCTCTGCTCCGGCGGTAATGATTGCAGATCGTGTCAACTAAAATCTGTTTGGATTT
CTGTAAATTGGAGTCCGACAAGCCTGCAGTTCCGTCGCCAACCTGATAACTCTCACCCCcctgtcttttgtttt
ccttccagcCTTTTTCCTGTCGCCATCTCGGTATTTAATCTGACACCGCCAGGCAGCGAGAAGGGCTTAC
TCTTCCccttaaagagaaagagagag

>NC_000002.12:144452845-144452987
GGGCTGGCTACACAGGAAACCTGGTCCCAACCTAAACTACTGACAAAAGCCTTTAATCTTTCCCACA
TTCCTCAAGGCATTCTCCTTCTGTAATAAGACATATTCCTTTCAAGTAAATCCTGATAagggaattcattttttt
>NC_000002.12:144455038-144455235
GCTGCAAAGTTACCTGACTCAGCTGCTTGTAGAAATCAAATTCAGTCAGAGATAACAGCTAATCATTT
GTCACAGTAAGAAGCAATTCCCTGAAATCGGAAGGGAAAACCTGCCGCTTCAATCCGCAGGTCTGTC
AGATTGCTAGAAATTACATTCTGCTCTGACTAACCTCTGCACTTGGATTGCTAACAACAAGC
>NC_000002.12:144455276-144455381
GCTTTTGATTCGTTCTGTTCCAATAACCTGAGAAAAGTTTATGGACATGCCTTGCTGCCTGGACCTCA
ATTTCCACCAACCTGCACACTGTTTTGAATTTATAGC

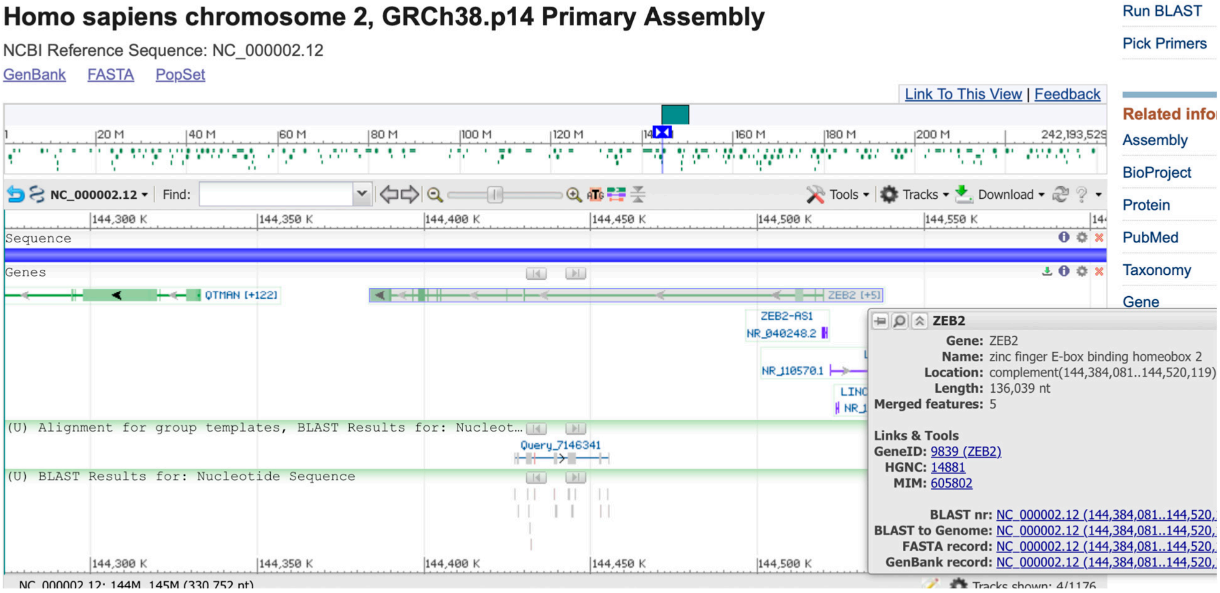

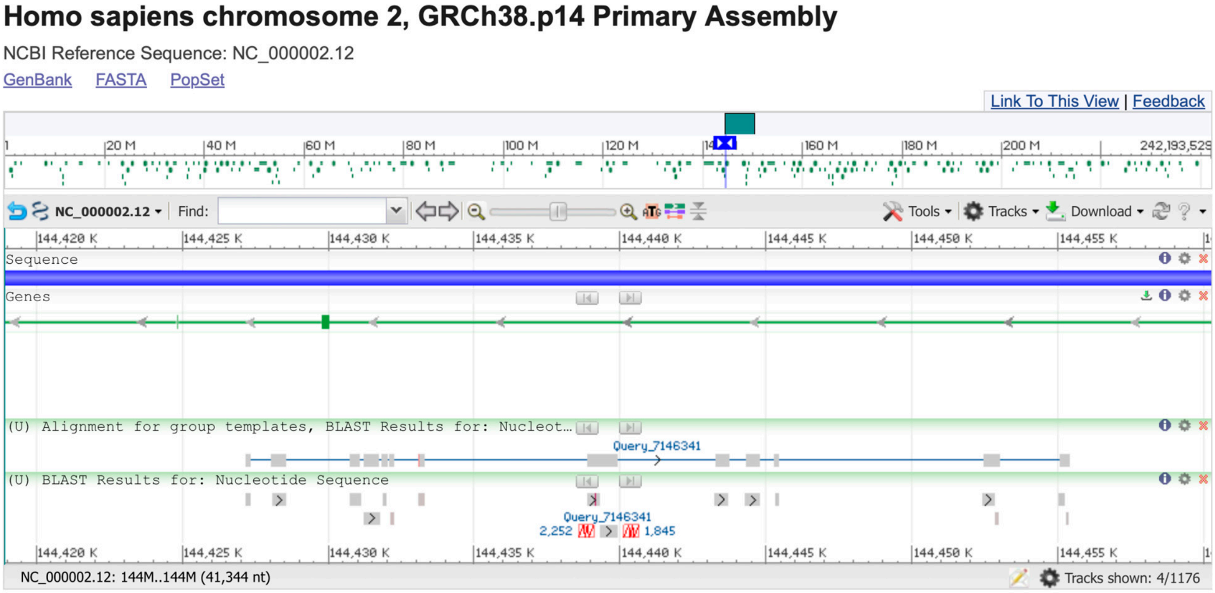

The same gene as the previous cluster!

chr2 CLUSTER 15 positions on BED file: 2079 - 2089
>NC_000002.12:144489536-144489819
tatttcatttataaattactagAATGAtgctattatatattattaatttctgCTGTTCTCCAACTCTGCTGCAGCAGTGAGCTGCG
GATGTTGGAACGGTGTAATTATGTTCTAGTAGGGTAGCCATCCGTTTCCTTTCAAAAATTTACAGGAT
TCTTAATCACGCTGCTCATTTGGGTGCGCAGCAGCGAGGATGGCGAAACACAAACAGGGCACGTTC
GTTTACATAGGAACACCTGCTTAGCATGATAGCAGATGTCAGTCAGTAAGcagaacacaaaag
>NC_000002.12:144494595-144494909
aaggctacTGTTCCTTTAATGCCCAGCTGGTTCTGAGATGTAATAAAATTGACATATTGATTCCTTTGAAT
TTACAAATGCCTGTTTCataaactccatctccaaagccTAGGCATCTTTAGAgcactgtcattttttttctccaaatggaTCAATT
ATGTGCCCACGTAATCCTGTTACCTGTGAAATCCATGCATACTTCTCCCATTTGCTGCAGTTCCCAGG
CGGACAAACTTGCATTATGTACGCATAAACAAACAGCGCATTTTCCTGATATTAGAGATTGTTTTCAAA
GTGAAAGACAATCTCTGGGG
>NC_000002.12:144499965-144500675
gggagaaaaaagatacagaatgcatggattgattttcaaattacctcaatattttgaaaacattttcaccTGCATGCTGAGAATGCTAAG
AATGGCAAGGAAGGCAATTGTGCATTTGCACAGCCTCCACtgcattcatttataaattacctcaTCACTGACTGAA
ATTAACACTGATTCTTACAGGCAATTTCTCAATTTCCAAtgctaattacattttttttacttttaaattctgTACCACCTGTT
TTGGGCAAGACATCTTAGGCAGCGCGACCGCATTTAATCACAATTTTAATTAACCGCCCTGTAGATGT
GAAAAAGAAGCAATTATAATGTAGATGAATGATGATTTTTCTGCATTAAATTGTTTTGTTTCCCTGGTA
TGTTGATTGTAACACAGCACACAAATACAGTAACtgtacaatttttttctattgtaatttAGTAATTGTTTTTGGAAAAC
CAGTTTGGGAATGAGTGACTTCATCTGTTTACAAATTGATTGATACTGTTCACTCACTGATTGCTGCG
GCGTATATTTTGGTATTGCACAGCCTAGCATAAAATATAATTAGAGATGCACAGGCTTTGCAGCACCA
TTTTGGAATCTGAAAATCAACTGTAACTGAAGTATGTTTTCCTTGAGCATACTAGTATCAGTTGGGGC
TGATGAAATGATAATCAAAACAATGGGCTTTCCAATTCCA
>NC_000002.12:144503378-144503483
CCTTTGTTCACAGCTTAACAAAACTGTGCCGTCACACCACTTTAGTTCAACAGTGCCTGTCGAATGGG
CATCAGCAGATCAGCCAATGCCTGTCATACACAGAGC
>NC_000002.12:144504473-144504986
GGCTGTCTCTTTTCCAGTCTCTACCCTGTGAGTTTCTGGCCTGCCAATTTCAACTTGAAGCTTGACTG
GGCTGATTTGATGCAAATACCTCCAGGGAAAACCCTGAAACTGATGAACAGTTATGTCAAGTCGATTT
TGCACCATCGGGAAAAGGTAACCTCTgcgagaaaaaaaacaacaaggtTAAAGAGACAAGGGCTTTTCACGT
GTAATACCTCTGCTGCACATCTCGTTCACTGTTTACCATCCCTCAGAAATTGTAACTGTCAGAATTAG
TCCTAATTGCAGATGCGAAAGCTGCCATTATAATAAAAACAGAGGCAGTGCAGGCATTAAATGTAGC
AGCCTAATTTGTGAGACTACAGAAAAGGTAGAGTAAATGAATATTTCATTACTTTAATTACCAGGGACT
ACTCTTTTCTTGCTGTCTTATGATAAGTGTTGCTGTAACTAGCAACAGCAAACATCCACTAAAACATAA
AGTGCAATAATCGTCCTGCTGTCAGGATCACC
>NC_000002.12:144507878-144508002
AGCAAATGATATGATTTGCTAAATTTTGGTCtttgatttatatttatgtacTCCTGCTACTGCTAAGGTTTTGCCAA
CCTCAATTAGATCATGAATGAGATCATTTTAATTCAGCCCCCAAACT
>NC_000002.12:144508981-144509083
ttgccaATTATAAACCACCATTTGGAGGGCTTATGAGCAATGTAAGTCCACCTCATCTAATTAAACCACA
TTGTTTTAAAAGCTTGAACAGTTTTCATGCCT
>NC_000002.12:144509251-144509372
GTACCTCACTTAACAAAAGCCTCCTTTTGCAAACAGACTCCCACTTTCCCCGCAAGTGTCCAAAGGT
GTTTACTGAAGTAAATCTGCCTAAATCCTTCATtgcttgggtcatgggggtgga
>NC_000002.12:144510718-144510982
tctctctctctttcttttcatcttgGCTTTCTGTGGCAGAATGCAAATGGAGCCTGCCGGCGGTGAAAGGGCTGAATT
GTGATTAAGAAGAAGAAGAGCTCCTTTCTTTGTTCTCCCCTCAGGGGATGTTTACTGGGAGAGACAC

AGCGGATCAGATATGAAAGGCATTCAGGTCAGCATTGATGTGAGCGAAAGTGAAATCATACCTAAGG
CATTCAAAAGACCGCCGTGTCAGGGGTTCAGCTAACGGTGCAGCtactgggt
>NC_000002.12:144511992-144512250
CTCTTTGCTCTGAAAAGACAACCAAAGAGCAATCAAAAAGGTGTCTGACTTGGCCATTCAGACAATTG
CCCCCTTGTGGGAATGCGGGAATGAATGGTACAGTGGACACCCCAGCACCATTGACGTTATAAACAT
GTAAATGAAACACATTAGGGCAGACAATCAATTGTCTGTGAGCATTGCAAACCTGCACTCCCTCTCTC
TGTGACAGGCACAAAATAGTGTCTACCTTGGGAAGCCACGGTGCTGAGAGGAAAA
>NC_000002.12:144517263-144517493
GGGCTGCTTCTTACCGTTTTTCCTCCTGGGATTGGCTTGTTTGCGCCTCTTGCACCGGGGGCCATCC
GCCATGATCGGCTGCTTCATTGATAAGAGCGGATCAGATGGCAGTTCGCATGGACTCGGCGCCCTG
CTTCGGCAGCACGCAGGCTCGATCTAGCAACCAAACACAGCGACAATGTGGGCATCGCCCGCGCC
CATTGAAACGCGCGCGGGCCGCCCAGGGGAGC

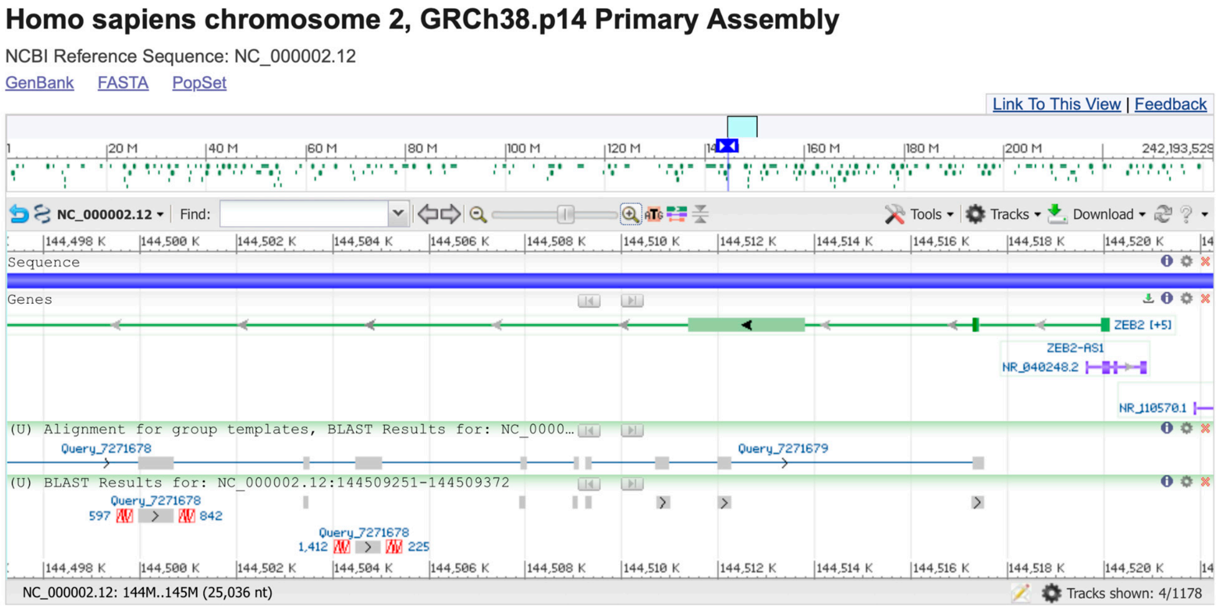

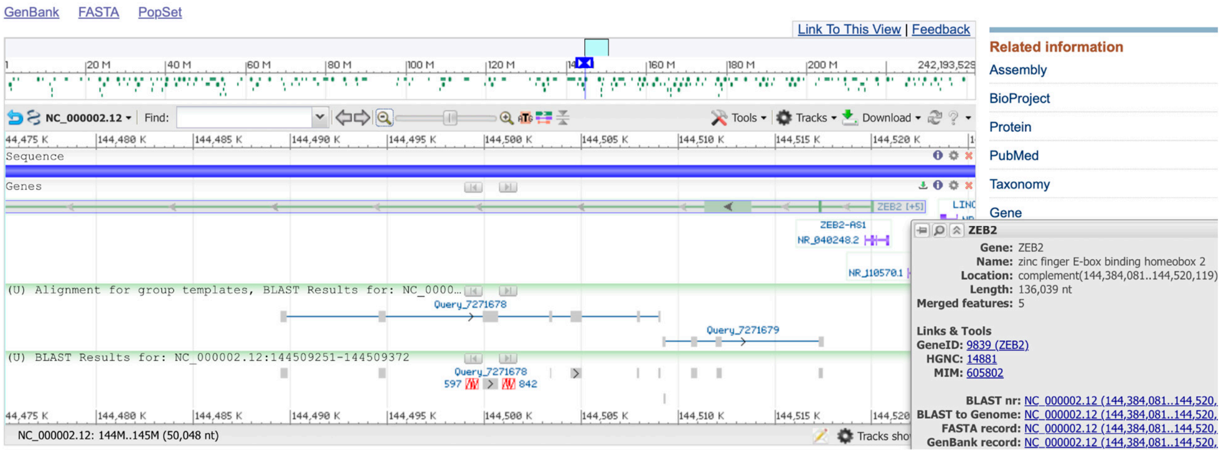

The same gene as the previous two clusters!

chr2 CLUSTER 16 positions on BED file: 2203 - 2214
>NC_000002.12:155715813-155716087
CACAGAGCTAGTTCAAAGGAACTAATGGAATAATTAGTCCCAAAAAGACCAAAAAGACTGAAGAATTC
ATGATGTCATAAAAAGCAGAGCTACTTAAATtggattagaaaataaaaatgacatgaaCATTGTAAATATTCACAG
ATCACATCATTGACGTGTAATGTGCCGTTAAATGATGTCAAATTAAAGGGGAAATTACATGTGTTAGA
ACTAGGAtgaataaaaagaagacatttaaagaTACATGGTCCTTCAAGTGAAAATTCCTAAC
>NC_000002.12:155724977-155725166
TGCAGAAGAAACCAGCTCAAAAGCAATTTATTATTAATGCCTGAATATTCTTGCTAAACAGTTCTGATT
ATTTCACAAGAAAGAGGTCAAGGTAGTGGAGAAAGGGATTTAATCAAATAAACTCTCAATCTCTCTCC
AAATAGCATTTTCATCCAGCTGCATGTAAATTCATCAGATGACTAGcctcca
>NC_000002.12:155726593-155726790
ACACTCTGATACAAAGCTTCACCTTGTCTAGTGctattccatttaatttttattaactgCCTGAACATATGCCATTCTA
GAAGAGGGTAATAGTTACAAAATGTCAGCAGAAACATTTAAACTGctaaaatgtaaatgattttataaatttgtaaTGA
AAGGTAATTGTCATGTGGGAAAAGTAACAGGAAAACAG
>NC_000002.12:155727341-155727468
GCCATGACAAGATTTGCAATTCTCTACGTTTATTTACACACCATCACCCCAACTAGACATCACATGGA
AGTGGCATGGTCACAGCTGACCCCAGGGTAGTCCCTGTCTCTGCCGATCCAATTTCATC
>NC_000002.12:155728243-155728501
tggaagacagaaagaaatccaaaacctagTTAAATAAATTGTTCATTTATTCCTTCCCTTTTATGCACCAAGATCTA
CATGTGGTCATTAATTATAAAGAGACTATTATAGCACCATATTTTCATTCAATACGATGTCCTGGTATA
TATCAAAGAAGTCCCATAATTAAAACAGAAGGGCCACAATGACAATTAAAATTACAATCTATATGGGG
AAAGAAAGTACAGTTCTGCAATTTTTCACCCTGTCACTGTCAGC
>NC_000002.12:155728531-155728646
AAAGCAGCCTAAATTCTTCTTCACCAATGCAACCTCAAAAGCATTATCAGCACCTAAGCAAAAGCCTG
TGCAAGTATAATTTAATACGAAGCCACAGTTTGCAAGTAATAGCGTA
>NC_000002.12:155728740-155728880
GTCCTAGGTGAAGAGGTCCTGTGTGCAGGTCTGTCTGTAAGCCTTACTTCCAGCTGCCAATCTTTAC
TTCACAGTGAAGAAAAAGCCACATAGCACCTTGTTCTTACTCTTGTATGCTGTCATCCCCTGAGCCTG
ATAAC
>NC_000002.12:155729177-155729486
CGCTCATATATGAAGCATGCAATGGAGATTGAGAAGGTTTTGTATAATAAAACTGTGACATGgtcatttttc
cagttttgcaTTACGGAGACACACTCATGCATGgtgctttttaaattcttattatgTGTGAGGTCAGGGGGTCGAATGTC
AGAAAGTACTGTTAATTTCACGGGGTTAGGGGAAAGTAAATTCAGAGGGAATACAGATCACTTAGCA
CAGAAAACCAGCTCCTCTGAAGTTTGGTTTTACATTTGTTAACCATATCCTTCAGCTGACGAACCATT
GAGACCACGGTGACATTCTGG
>NC_000002.12:155729559-155729725
cttcctctGAATCAATGAAGCACTAAAACTCCACACAACTGGAACTGAACAAAGCATGTTGCCATTTTGGC
TGTGATGTTTAAATTATAGAGAAAGCAGTCAGGATGTCTTAAAGTTCAATCTgacaagaaatgtaaaatatatttct
gtcttcattctcTGGGAA
>NC_000002.12:155730825-155731272
AGTTAAAACAAAATCACCAATTAGGCACATGAACACGTCTGTATTAATCTCATTAAGGTGATAGCACC
AAATAAATTGGAActatttttgtgcttttactGTGTTCCTATGGCTTTATCAGGCCCTGTGGTCATATACAAAGACC
TCGCAGCTTAGTCATTTAGAGTCATTCGGTTGTGGACTGAATGTGGCATTACCATCTATGGCTGAAAA
TTGCTTTCAGCTTTTCAACTGCACTGACTAGTCTGTAGTTAATCTTAGATCATTCACTCGCAAAATTAA
ATGAGCCAGAACGATggtctttttttatataaaataatacatcacAAAACACTTATTTGCCCTGCCCAAAAGAGAGatc
aaattcatttaaaatcCCATAAACCAGAgttgtgaatttttaattttatttagtagcAGAGAAATAGAGGGTAAGCTATCTTTCT
>NC_000002.12:155733009-155733589
tttttttaatagccTGAACcaaataatttctcatttttacagGCTTCACTTCACTTTTCTCACAGTGAATAAATTGCAACTTAT
TTCCATGAAATTCACAGATGACACAGATCTTTATTTTGCATAAAGGCATAAAGAAGTGTCTAATATCTT

CAATATTCATCTGCTAAGTGGAcatttgcattgggttagaacggAAAAAGAGCCCCAACCCTCCCGCAGTGACA
GCTGTACAAAGGGATGACATCTGACAGCTGTGACAGTGCTGAAGGGAAACTCAGATGACTTGCAGC
ATCCATGATCCAATTACAGCACACATCCCTCTGACAGACCCAGCAGCTTGACTCCAGCGTTTGGAAG
GACAGTATTGATCTCTTGTCAGAAACCAGTTGCCTGCCTGCAATTGTGCGCTGCTGAACTTTGAAAA
GCTATAGTACCCTCTTAAATGATAGGGGCAGTCAGGTTATAGTATTCAGGTACAAAGAGAGGTGCAA
AGGGTATCTGCTAAATGCTCTAAACTTGAACAAATCATAACAATATGACAGTCCAATCATACTCAAGG
AGCCCAGCTGATCTAGCT
>NC_000002.12:155733594-155733715
CTGTCTGTGCTAATGGAAAGACAGCTCTTTGTGTTTAGTGGGATGATCTTACTTGATGAAAAGGCCAC
TGCAATTAGTGCATGCTGGTTGGCTGTGCTGGGTGACAGCAGCATGGATGGTA

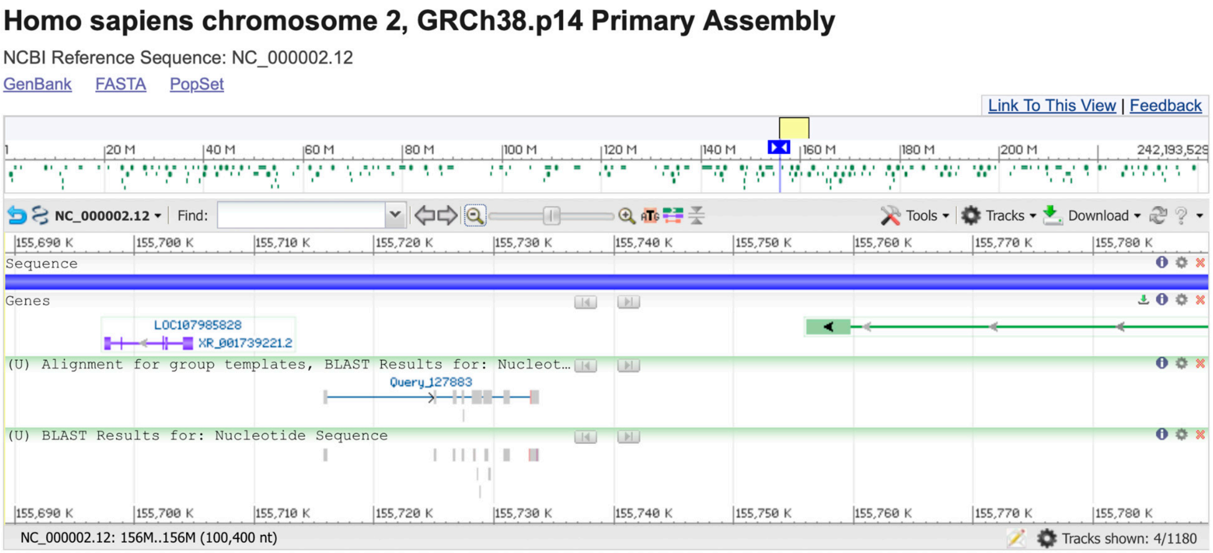

First INTERGENIC region

chr2 CLUSTER 17 positions on BED file: 2350 - 2362
>NC_000002.12:163593993-163594840
CTCCTCCAAGTACATTACTTATTCACTGGTTTGTCAGCAGTGTGCATTATTAGCGCTTGAGAGATAAA
ACTTTAAGTGTTGCTCCCAATTAGCACAACAGTGACCACGCACCATGCTCTGTGCTTAATGCCTGCTC
TCAGAGAGGAGCTGGTTTTGAAGGTCTGaggtggaggagaaaaaaaatgaaacagcttAAGCATTCATTTTGAGT
GGAGAGACAGCTCCTATTAACATTTAACAGCATTTGTGCAACTTGGTGCGGAGGTTATGATGCAAAT
GAGGAGGAATTAAAAGTGGCCAGGGGTTTGTCATTGATGGATTGCAGTCTGGCGGTGTTCACACTTC
TGCCGTGTCCATCAGAAGCGATTACTGTGTAATTAAACCTTATTTCTCTACTCTTCAGTGCATAATTAC
TTTGTGAATGTAACCATCATCTAAAAAAGCTACCAAAATGCTTTAGCATTTAGTCTAGCAGTCATTTCC
CTAGCTTGTGTCAGATATAACCATCAGACAGTGCAAAAGAACTGTCACTTTTAATAAGAggcaaaaaattaa
atgaaacagtATGCTTATTACAGGAAAATTAGGCGTTCAAGTGGTAGAGTCCTTTTCTGCTATTtgcataatttat
tcttttttgtcccTCACACCAGCAGCTTCAGGCaattttcttcacatttaaaTAGATCGCACATTTAAGGCTACTTAAGGA
AAATTATCACTTTGCATATTTTAATTGTTGTAAAGAAAGTGAATAGAAGAGGTCTGCAGCTTGGACTCT
TGAGTCGGTCAGATGCTCAACTGTCTGATTCTGGAGCTCCCCACTGCTGCACTAGAGATAACCCTA
>NC_000002.12:163601571-163601751
TGTCGGCTTGGCAACTGTGTGACTCATGCTGTCCgacaagtgaaaagaaaaccgCAGTCAGCAGCTATTCA
GCAGCAAATcattaattaattacattttaatgaacCTTAAGCGGCTACTGCGGGAGCCTTCAGAAGTTTATCAAAAA

TGAAGAATTGCCTTGTGTAAATCTTGAGCTGA
>NC_000002.12:163602628-163603222
tttttttatttgaggtatGCTTGTTCTTTTCTTATTCCATGATAGTAGCATAGCCATATACAACAAATTTTACAATAG
AGAaaacttttcataaaaatatctgCCTTGAACCACATTTTTAATTCTTCAATGGTTACTACTGAAACTAGTTATTC
TATATGAAAACTTAATTTCCCTGTGGTAGCAAGTATTTATGAAAAGGAATATTCAGTAGGGACAGTTGT
TACAATACTGagcactgaattttttttaatatattacagTGCCAACACCCAAGAAAATGAGATGCAATGGATTACAATT
GAAACTGCAACAATGAGGACAAAAAGCATTTTCAGATCTCCAAGTTAATTGTACAGCAGCTTCCTACA
CACATATGATCAACCATATAATGAtcccatcaaaataaaaaaaatctaagcagaAATGTCTCATTTACATTAATAAT
GTTCACAAATTATATGAATTCATTTTGCAAGTGATGGATAATTTAAGCTTTCATCATCTAAAgcttgtcattttttt
ccaagagaaatggattgtttcatttttaatgagtGCAATAATAACACATTTAGTTTCAGCAGATGTAA
>NC_000002.12:163603325-163603426
AAGGGGTCAAAATCTGGTCAAATCCAGCGTATTGTTTCAATTTAAGCAAATAAAATCAATATGGTCAG
GGAAGCAAAGTGCACAGAAATTTTAATTAgtta
>NC_000002.12:163604498-163604702
CTTATCATTTAGGTTGCTAATACTGTTCAGTGTAAAGACAGTCTTTCTGAGGTAGACTGTTCAAGTTCA
AGGACAAGTCTTTTTCTTACGAAATGCATGACAttcaaaaaaagacaacaaataaataCTACATGTACAATATGT
AGCAATGAGGTAGAAATGGTTATAAAGAACAATGTTTGAATATACatcagatgaaaaaag
>NC_000002.12:163605002-163605138
TACTACAGaaccattttataaatatttaacattctatAGGACTGATTCATATATTCTCACACAACTCTGTGGCAAGGCT
GACCCATGCTGCCGACATTCTACATATCACTGAGACAGGGAGGTGAAATTTTCCTGC
>NC_000002.12:163608883-163609086
AACACATTCCTTTGAGGTACTGTACAAATCACAATCACTTTCCTGAGTTTTTGCAGACAAgaacattaaaag
aaatgaactgcAGAAATTAAGGTCCAGATTACCGTTACCCTGTTTTACCTGTTCTTAGCTTAATACTGCAGA
ACAGACAGGATTGAGGAATACTGTTGCtcttaaaatggcaaaaatctgGTTTTCTGTCA
>NC_000002.12:163609097-163609289
TTCATCTCTTTAGTCCAATAATGTTTGAAGTTAAAGCTCTTTATATTAGCACATGCCACCAATGTAATT
GTgaggcaaacaaaaaataaagcaccACTCCAGGCACTTGACAGCAACTAAATCTCGAGAACAGCAGAATGG
AATCAACACACGAGGTTCATGTCCTTCTGAAATCAACACACTTGCACCTTG
>NC_000002.12:163609546-163609659
agttATCACTGACTGCAACCAAACATTTTGTTCCATTCAACATACATATCAAGCTCCTTTTGAGATATGC
TAGGCTGAATCTTGCAGAAAGCATTTTCAAAGTCTTGATATGT
>NC_000002.12:163609732-163609920
GCTTCCTGACACAAATGAGCCACATCTAGTCCAGAAAAGCCTTCTGTGCGCTGGACGAGCAGTGCAA
ACTCCTTGTCATTGAGACAGTAATTGTGCTGTGAGAGCAGTTGTACTATTATCTGGTGCCTCGCTGTG
CTGTCAGGAAGTGGGATTAAAAGTCGTTTCATGAAGTACCTCCGAAGggattc
>NC_000002.12:163610629-163610754
GGGGTCAGAGCCCTACTGGACTGGCTGCTGaatttcctttgctgttcagagGACATTAGCTGCTTCGTTGGCTT
AAATGCTAAGGATGATGTTTCAGCACTTCTGTCAAAGCCATTCCCCCGATT
>NC_000002.12:163612316-163612514
CATCTAGGATACCTCTCTTATTTGGTACTGAACACCTAGTTAGCACAGTATAATGCTCCCTGCGCAGA
ACAATATGACACAGACATAGCAGGTCATTCCATAATCCAACTCTCATCTAAAGTGGTCCACGACAGTT
TCAAATCTGCTTCGTGGAGTGCAGCAAAGCAATCTCCCAGGCAGCGCTCCATCGCTTTCATC
>NC_000002.12:163613523-163613801
AGTTTGGTAACAGAATGGCATGTTGATTCCCTTCTGACTCCCTCTTCCTTGTTTGCAAAACCACTGCA
CCAGACACTGGAAGTTAATTAGCAGGATGATGCTGTGCTAATTGTGTTAATTTACAAGGTTTTAGTCA
AAGAGAATCATGCCAGGGCTGGCACTGCTGTCATGCTTCCGACTTAATGATTAAGAAATACTGAAAA
CAAGGTGGGAAGGATTGCAGTAATTacacaataaatgaataaagcaacaggattcattgcaaatatattttact

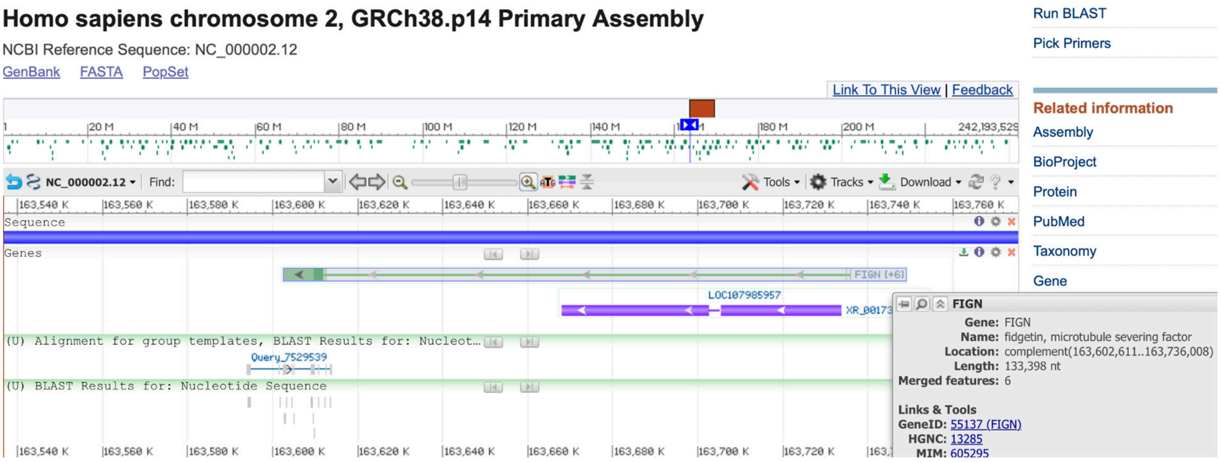

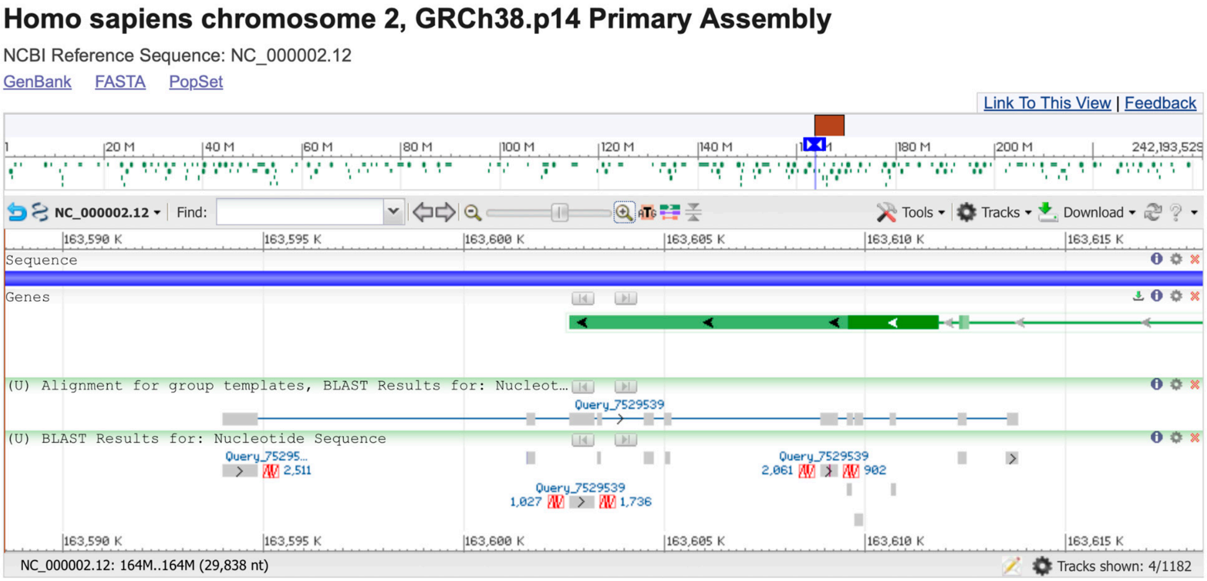

chr2 CLUSTER 18 positions on BED file: 2365 - 2377

>NC_000002.12:163711465-163711567
GTGCCACAATAACAGGCTAACCTTGAACTGAACTCCTTTCCTCAAGAAATTAAATGCTGTAGCAGGAC
TTTAAATCCCCCCACTTAGAGCTCATTTGTGCGC

>NC_000002.12:163712094-163712238
TACAAAAGATGAATCTATGGATGTGACACTTGTAAAGGGTAACCCTATTTCACAAGTGAGATgggacaaa
aaagaaagaaaaaaggccacTGAAGCAATAACTGCTCACTGAATTACCATTAAATTGTAAGATTTACAATCCA
A

>NC_000002.12:163714037-163714166
ACGGGGTCCACAGACGGCTGCTCTCCATGCAGAGAATCTCAAGCTCCATTTTCCAATCTGATTACAC
GGCTCCTTGTTCCAGGAGCCTTAAACCTTCATGGCAGTTTAccaacattttcatcacttttt

>NC_000002.12:163715908-163716053
AATGACTGTCAACTTAAAATGCCATTCTCTAATTTATATGCAAATTCTACCtctggtaaaataaaaacatattttaag
cagCTTATAGTGCCAACACATTATTCCCATGAATGATTCCTAGTAAACTATTTATGCCAACTGTGGG

>NC_000002.12:163716079-163716186
AACGAAACCACTTAAACTATAGTTGTCTGTATGAATCAGTAATTTTCATGTTtctaattaaaatgtaaacagcGTA
ATGAAGACATCAAGTTTTTAGGCAAATGTCT
>NC_000002.12:163717823-163718068
TAGAAggttattcatttaaaaatcaacgTTTCAACCATATCAAGCTGTAACCTTTCTTGAAATGTAAATGAATGGTT
TAAATGAGAAGATTAATAAAAAAGGGATGTTCCAATTCCAAGCATGGCatgctatgtatttttaaatttgtctatGCCA
GATGCTGTCAGTCAGAACAAAAAACTGTCCAAGCAGACAATCTGTGCATCTGAATGTTGATCATGTTG
GAAAGTacagggggaggggg
>NC_000002.12:163718070-163718722
gagtgTGTCAAGGATTCATGGTCCTTCATCTTTGCAGTAGAGCAAACACGTCTCTCAGTATAGCGACAG
ACAGCTGCTTCCACTTTCTAATGTGCCCTAACTTAGATCACTGAAAGAATTGTCAGCACTCAGAGAAA
CACACAAAGATAACACAGCCAGCTGCCATGGAGGGGAAAAACAGCACAGCTCAGAAGAAATGGGAT
TTGTTCCATTTGCATGATGCTGCCTCCCCTGGGCCCCTTTCTTTCAAATCCAAAGCACAAAAGACAAC
ACTTAACATATTTGCTTAAAGATGACAACCTCATGTTGATTTCATTTGGATGTAACAGTGTCAGGATTA
CAGatataagaatatattatatCTGTAACAGAGACTGACAAATGTACCCATCAGTGGAACTGTCTGATTCATCT
GCTTATACAGCCATTTTACCATCCCAAGGGTGCCATCTAAGTAGTAACTTTAGAAGACTGAAGGTCTA
ATTTTATTACACCACATTTCAACCTACTAaacaatattatttctaaaatagatCATTTTTATTGGAAGTCTCCCTGAT
GAAAAATCCAGTAGACACCAGAGAATTTATGAACTGTATGGGTCCTTTGAACTCAATATACTGCCCAT
TTTAAACAACTTTTCTTGCTGTAT
>NC_000002.12:163732791-163732931
GACCTAACTTTTAGAGGATCAATGTGACAAGGCAGTCCCTGATAGATGTTTCTCTTGATAAACGACTT
GTTCAAAAGAGACATTGTAGCTTTCATTTCCTGACCACAACACACTTTTAATTTGCAACAGCAGAGCT
GCCA
>NC_000002.12:163733390-163733688
CAATTTCTGCTCTCAGCACATTGTCCGAGCTCcgatttaaaatttataaatgtaatatccTCCTCATCACAGGCAGC
AGCCCAATCTGCTCTGAATATATATTACAGCTTTAACATCACAGAGGGAAATCGCCCATCTGGCAATC
CCATTCGCTCCAACAGCTCCGGGGGAAAAAAAGCTGTGCTCTCTCCTCCTTAAAACATTTCTCAGGG
CAAATTATGTGCATCTAAAATAAACAGTCTTCTTATTGATCGCTTCAAAGTCAACAAGTTCCAAATGCA
AAATTCAATCAAATCTT
>NC_000002.12:163734860-163735324
CCTATTTAGATGCAAAGGAAGTAAATTCCAAAACTGACATACCATAAACACTGGTGCTACTGATCATT
TCTTGCTGGCAGCACAAAAAGCTGAATTGGATTTCTCACAAGCAGGAATTCCAAAGGTTGCTTTTCAT
TAAAGCCACTTTTCCTCTCAGCTATCAAATGTCACTGCCTTGAAACGTGGGCCCTTTCGTCAGGTATT
CATTTAACCTACATGCATATAATTAAGGGGGAaagcaacatcaacaaaaaggggATCTCAGATcacaggagaagaa
agaaaggaaaaaaagcacatGACCAGGAATGCAAGTCCATGTCAATTTCACTCACTCCCATTGAAACTAACA
AGAGCTCCGGGGAGGACGGTAATAGGATCAGTGGATTGTATATACCATTAAATTATACATCTTTCTCT
CCCGTTCCTTGCCAACAATACGCCCACCACGCTGTACCCCCTCC
>NC_000002.12:163735855-163736135
AACCCGATAGGTTAGCGTAGCATCGATCCGATGTGTTTGCTGATGAATGGAAGCTAGGGTTAAGTGA
AGGTGCCTATGATTTGAAATCATTCCAAGTTTTTGAAGGGAAGAcggactgcagcctctgccatgTTGGAATTT
CAAACCCAAAACAGCTGCTTGGAAGGAGCCAGCATGCCAGAGGCTGGGCGCAGGCGCAGAGCGCC
GCAGAGCCAAATTCAAATCACTTTCACACTAAGAGCCAAAGATCAGTTTtcaaaggagagagggagaaagaga
c
>NC_000002.12:163737145-163737316
CAGTGCTGATTGTCTGTTGCTGAGATAGCCTTTTGTTCGGAACATAAGGTTTAAGAAACACACACACG
TTTTCTGGGGTCTCTTGTTAGATGTAAATGTGCGGCATCCAGACGCTTACTATGCAGTGTAAGCTCAG
TTGGTTTACTAATCACCCCGCCCCACCCGCAAACA
>NC_000002.12:163737361-163737463
GAGTATTGCCTTCAACCTATTTCATTCTAGACTAGCCAACCATCCAACTGAGATAATAAAGCAAACAA

TAACATTTGATCATGATGTCTACGAGATAATGTG

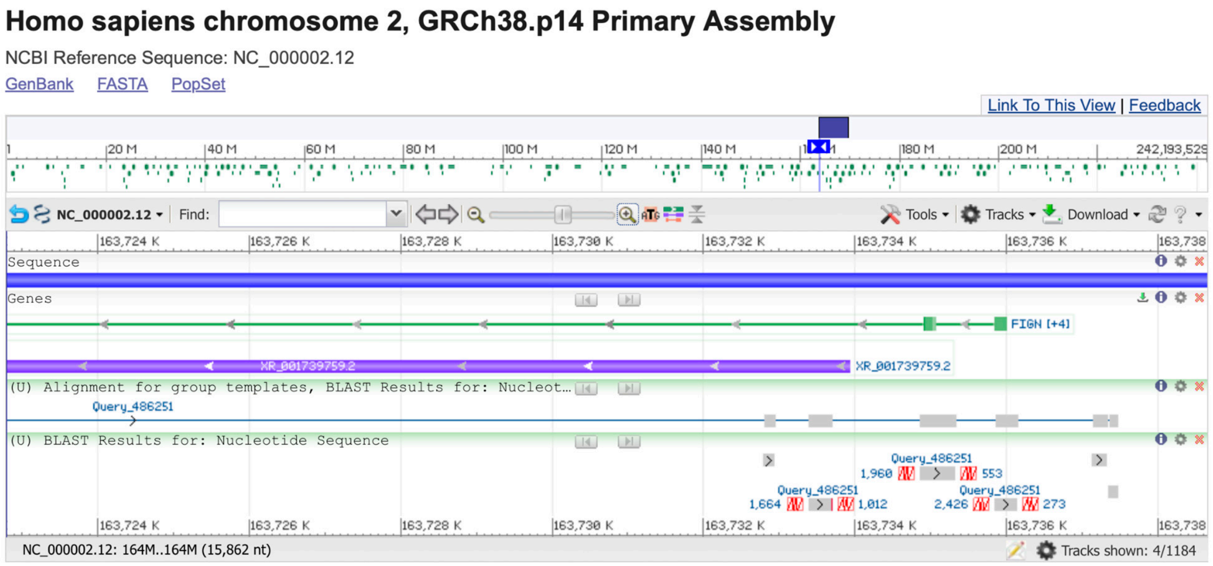

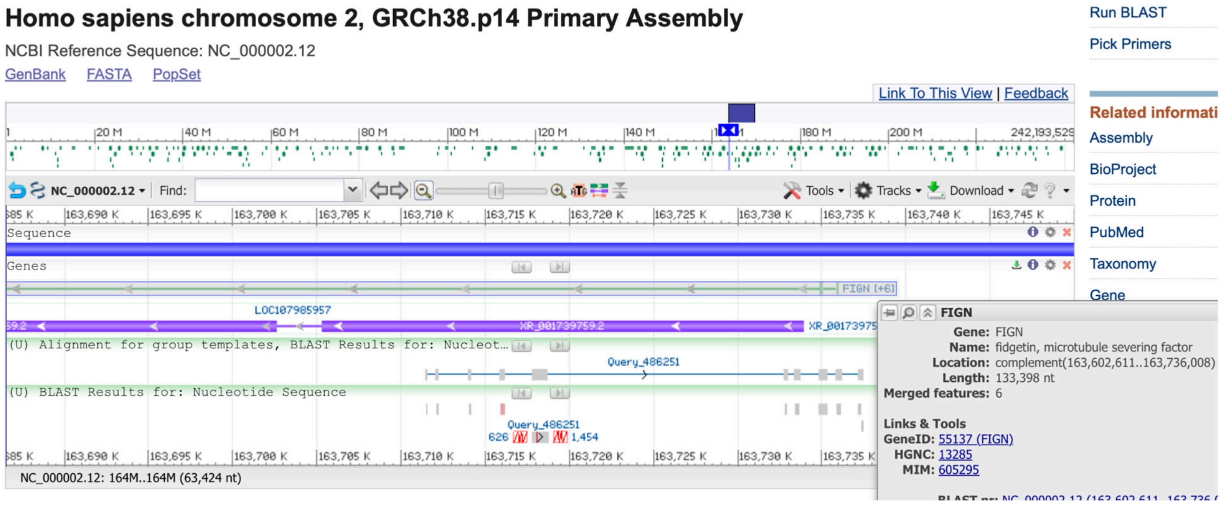

The same gene as the previous cluster!

chr3 CLUSTER 19 positions on BED file: 3051 - 3064
>NC_000003.12:71041486-71041632
CTTGTCACTTCTTTCCAGAGCTGCTGCAGTTCTGTTGGAATCATGCCTGAAACAAACAAATTGGATAA
TTAAATCAATTAACAGCTAATTCCGTCCTCTTCAAACAGCAATTAAATCAAAGAGTCAGTAAACAAAGC
CTAGTGTTA
>NC_000003.12:71042029-71042129
TATCGTCTTCCTGAAATGCTTTTCAACTTTTAACAGTCAAATTGATTATACTATGATTATTTCATTAACA
CACCTATCTTCCTCATTAATTTTGGTTTCT

>NC_000003.12:71042747-71043080
GGCTGTGTGCATTATATTCATTTGaaacacataataaaaaatgccaaggtctaaatttaatttatttttgtcatttagaatACAATGG
ATAGATAATCATCTCAACGTGATCAGCCAAACATCTTTACTTTGCTAAACTGTTAAAGATTTTAATTAT
GCTAGCATTGGAGAGCACTGGTCTAATGAGGCGATGGACTCCAGAGAATCCGAGTGGTTAAGAACT
GTGAAATGAGTCTGATAGGATTTTTTTATATTCACCGTGGATCGTTTGCATATCAAAGAGGAGTAAATT
ATTGCACAGCAGACCAATAACCTTCCCCGCCTTTCTGAGAG
>NC_000003.12:71045275-71045385
TGCAGAGATTACACTTCATGCATCACCACATTCATTTCTTTCACATTACACATGAGCTTGTCTTGTCAC
TGCCCAATTGCCCTCTTTTGGACAGCTTAACTGATGTACTA
>NC_000003.12:71047528-71047705
CTCTGTTTATCACttctgacataaataaatgagaaaaaatcagacctttgaagaagaaaaacttcatctAATATTGTTAATTAGCC
ATGACAAACGATGAAATAACATTGTAAATCTTTCACAGAAACAGCCACTAGATTTTAATTACACACATT
CATAATTTAGCAAACAACATCG
>NC_000003.12:71048541-71048646
acaaaaatttaaataaatttaaatgaaaagccagtgttttcattttaagaacAGCATGCATAAAATGATCCAATTGTTTTCACCC
CTTCCCAAGTTATCACATTA
>NC_000003.12:71050384-71050534
aaaacGCAATCCCTGGTAACTGATAAGAATGACAGTGGCAgccctttttttgtttgtcttcaaATATAGAGTTAAAgatctt
taaaaaactaaattttgatgccttttgaaaagaaagaagcacaaaATAACAACAGGGGGTTATTGCCTGC
>NC_000003.12:71050535-71050719
AGCCACAGGGGAGAAACTCCATAATTCTTATTCTCCCTTTTGAAGGCTGTTAGCAATCTGATTCAAAA
AAGCAACAGCAGAAGGGGAAACCTCTTGAGGCTGTAACCATTTCCTGTGATCATGCATAATGTGCTT
CAAAAGTGGGTTTTTACCAATTCTGTTGATCAATTGCACCTCCTTCATA
>NC_000003.12:71050720-71051254
CCCTTCTTTTTTCTGCTGTGTTTACATCTGATGTGACTCAATGACAAGCACTGTCTGAGACTGTGAAA
CAGGCCTGTCATGTTGATTTATGGGCGCATCAAACCACAACTTGTCCAAACTGAAGCTCGCAGTTCA
TTAATTAGAGAGTTGTGACTTTGAAGTCAGCTTTCAGACTGTGGTTTTTAACATTTGGCTTAATTTATA
TGCTCTTGAATGTGGATCTCTAAGGAAAAAACTGAAATTCCCTTCTTGTCTTTGAACTTCTTTTGACCG
CACAATAATCATTTCTTTGTCCAGAgtgatgcctgcaatcccagagtCATTAACGCGCATTGAACTGCCACTGAT
GAGTAAGCCCTGCTGAATTAGAAAAACAGCCAGCAAAACAAAGCTGAGAGCCTTCTGCACAGTGATA
TCTCACAATTACATGCCTTCCCTCCCTGTgccatttccattttaattactGTTAGTCCTTTAGATTTACATTTTAGA
CACTTTTTAATCTGTTCcctttttttattatagcagcacccGG
>NC_000003.12:71052501-71052638
CTGTGGTTTGAAAGTAAAATATGTATTGTCGGTACCTCTTTAGGCTGTTTTCCAGCATGTTGTTGTTGT
AAAAGTTGAAGCTGCAACTGTTCCTGTTGTTTTTTATAAAACTCTTGAAGCTGCTGCTACAAAGGAAA
>NC_000003.12:71054486-71054708
CACTGTATCTGAAAGGATGATGTCTTGTTCAGATAAAACACTGACATTAAAATGACAGGCTGTCTTGG
CCAAGGTTACACAAATTGTATTCAAGCGGATACCTAATGACAATGCTATGAATTCTGCAAGCTTTCTA
GAATGTAATTTAGCTATTCAAAATAATCACTGCACTTGTGTTTAATTTGCAGACTACATGAGACCCATT
TTGTCAGTCCCCGTAAT
>NC_000003.12:71055081-71055228
CATAATGTCTTAATATGCAGTTTATCCTGACTTTTTCACATGATTTGTCCTGTCATTTGCATAGCGAGC
TCTCATTCCTAATTTCACAGTCATTATGCACTGATGTCCCGAtttctcagcatcactaattTTGATGAACTAAAAA
TG
>NC_000003.12:71057534-71057669
TGTTTTATGAGTTAACAATGAATCATAGAAGGCAATTTATGAAGAActagaatgattctgtctagctGCCTTCAGG
GatcagaaattcaaattaaacttCTGGCAGACATTTAAAGAATGCTAGCAtacaaaact
>NC_000003.12:71060638-71060789
CCCTGTGTCATCTTGTTTTGTCAAGACATCAAATAGAATTGGGAAGTACAAAATAACAAGCCCTTGAG

AAATCACAGAAGCCGACCTTTTTGATGTTGTTGATTTACCTTGTTCTCAGTTATTTGAACAATGATGTT
TCAGATTTAAGGGC

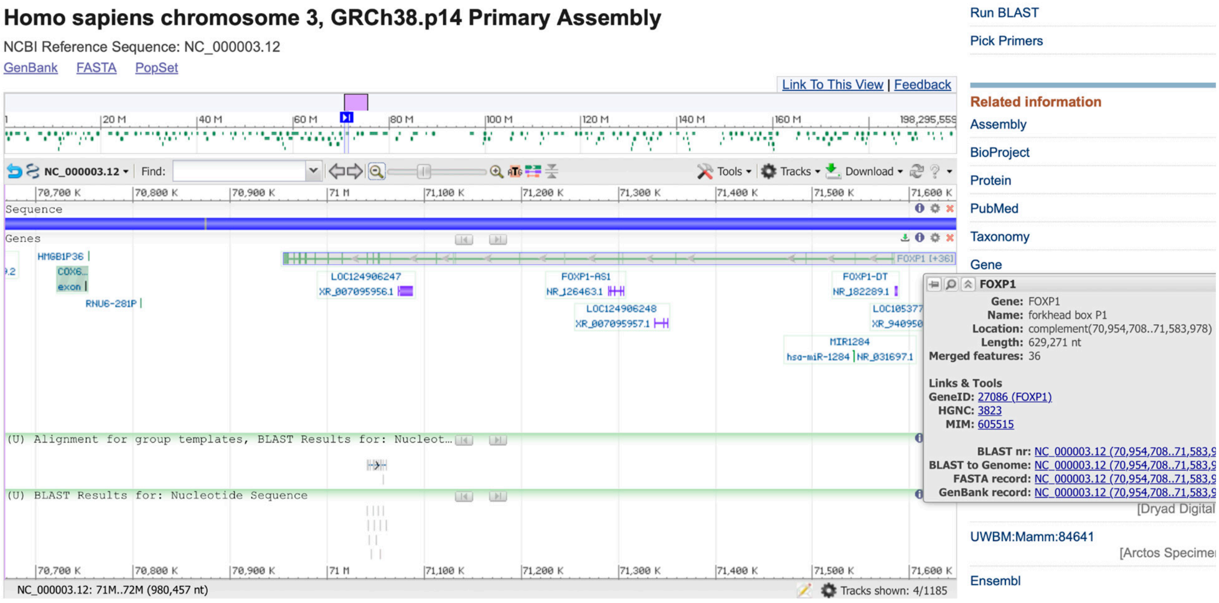

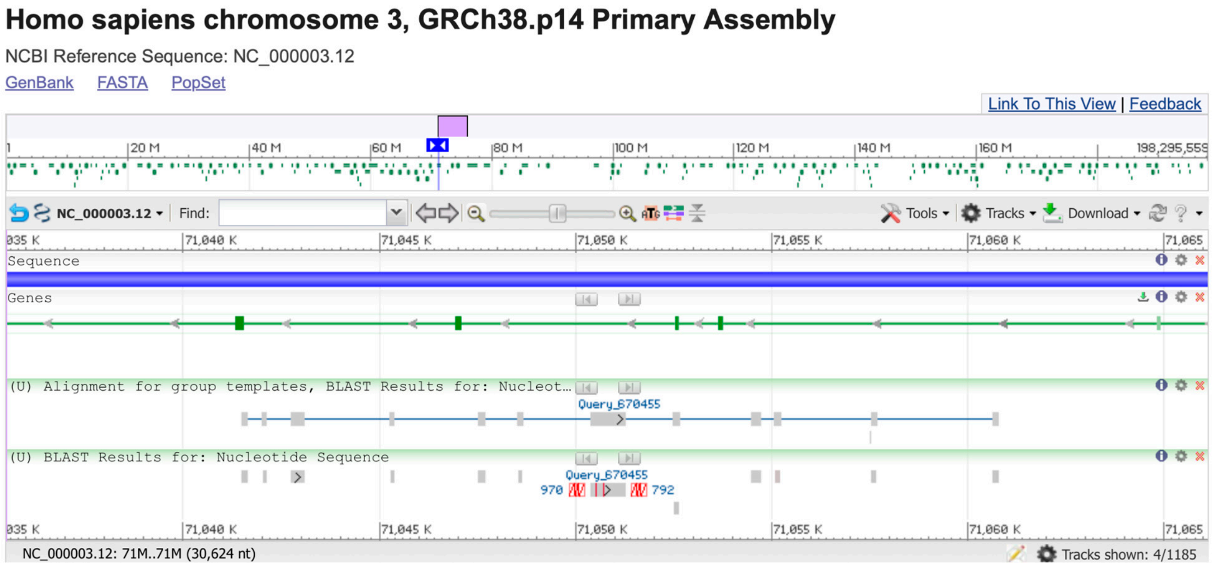

chr3 CLUSTER 20 positions on BED file: 3312 - 3322
>NC_000003.12:152446474-152446821
AAGAGGAGTTATCCTCCCAATAACAACTCAGTAGTGCCTTTATTGTGCATGCTTAGTCTTGTTATTCG
TTGTATATGGCATTCCgatgatttgtttttttatttgttttttctcacCTACCCAAAAATGCACTGCTGCCCCCATGATGCA
CCTCTGCTTGCTGTTTATGTTAATGCGCTTGAACCCCACTGGCCCATTGCCATCATGTGCTCGCTGC
CTGCTAATTAAGACTCAGTCGGCTGTCAAATCACTGAAGCGACCCCTCGAGGCAACCTTTGACCTGG
TACTATGACCTTTCACCTTTTAGCTTGGCATGTAGCTTTATTGTagatacaagtttttttttt

>NC_000003.12:152447609-152447750
CACTAAACAGGGAATTCCTCAAGCTGTACTTCCCCCATTACCAAAGAGGCCTGCTCTTGAAAAAACC
AACGGTGCCACCGCAGTCTTTAACACTGGTATTTTCCAATACCAACAGGCTCTAGCCAACATGCAGT
TACAACA
>NC_000003.12:152455406-152455617
GTTACATGCCACTGTTCCCATAataacaatttttcttctttgttcaaaTGAATTTTCTAATTACACGTGTGATGGGATG
TTTtaattcttttccctttcaaaTCCACCTTCCTGTTGCAATGCATGATGGGCAGGCTCAATATTGTGCATGACAC
CCGCTACAAGTGTTGGTAGGTGCCAGCTTTGTTTCTTGATTATCTTAAACCTATG
>NC_000003.12:152456194-152456397
TTCCATGTTGCCATGGTTTCCATATTGCTACACTCTTCTGTATGTTCGCTTATATGATTTTCCCTCCGA
AAGTTCCCATGGTGCACGGTGCTACGCCAGCCACTGTGTCCGCAGCAACAACATCTGCCACAAGTG
TTCCCTTCGCTGCAACAGCCACAGCCAACCAGGTTTGCTAATTTACagctttgtttcttaaaaaacaa
>NC_000003.12:152458028-152458145
TAGCTGATGCCACAAAATGCAGCCTGCATGCATGCAGAAGTTTATAGCTGGCCCTTCATCTGCATCG
ATTGGTGTTGAAGGTCCTTGGTATGTTTCGACAGCCCCATCCTTGATGCT
>NC_000003.12:152462732-152462858
GATTCTATTATGTATTGGTACGTCTGTAGACcaagatataatttttaaaaataagtttatttctttCAAGGTTTACAAATAA
CAAAGGTGCACCTTGTATTTAAAATTGCCATTATAGATGAGAG
>NC_000003.12:152462977-152463081
TTTTAAAGCACATCATGGCTTTAAGTACCATGTTGTTAAGGATTCTCATGAAGTGCCATAGACTGTAC
ATCAAATTAGAGTATTATTTCTTCAGTGTTATTGTT
>NC_000003.12:152463875-152464051
GTTATAGAACTTTCCATACTTCCAAGTTTACTGCAAGTTTTTATGCTTGAGAGAGATGCTTTCTAATAT
AAGACTGATGTGTTGATTTTACTGATTGTACTGTACATCTATTAAAGCCTTAGATTATTACATTACGGG
TTGGAACCCATACCAATGTAATTTCAATCGTGTTAAGA
>NC_000003.12:152464348-152464723
AGTGTAAGAATGTCAGTCATTGTTAGTTCTTGTCTAGTTTTCATTAAAAGAACAAAGATCTTTTATATG
GatatcttataaatatataatcattgcTAAGTAAGAAGTTAAGTTGTTGCTATCGCAACAATCCTGGCAGACAATTG
AGTAATATTTTgatgatttattttgtttgtaattaGTTATTATAAGAAGATCTAGATCCTAgatattagaataaaatttattttctactgt
atccatttcaaatgttaaaatattgtttaatatttttgaaatcccTGAGTATCAGGCCTTGTTATAAATAAGCTGCATAATCAAT
AAATAGAACAAGGGACTTTTTGTTGATAATCCAAATACTCAAAGTTTACGTAA
>NC_000003.12:152464788-152464936
GTCTAGGGAGAAGGTTGACTTTTTGCACTTCTGTATATAGTCAAAAGAGAGAAACCTGTATAATAGTA
AGAtcttattttgaataaaaacGTCTATAATTACAAGGAGTTTTGTTAAGGCTAATACAATGACAGACTGAGCAA
AATTG
>NC_000003.12:152465598-152465777
TGTGCTTGGTTTTACTATTATGTAATCACAACTTACTTTCTGCTTGTAGTtgcttaaaattatgtattttgtcTTGGG
CTGCAATTTgttttatgcttattttattattactgcaGTAGTTGACTTTGCTGtatggaaaaataaagtgaaattgcCCTAATAAAA
CTTCTCTTTCTTAA

Homo sapiens chromosome 3, GRCh38.p14 Primary Assembly

NCBI Reference Sequence: NC_000003.12

GenBank FASTA PopSet

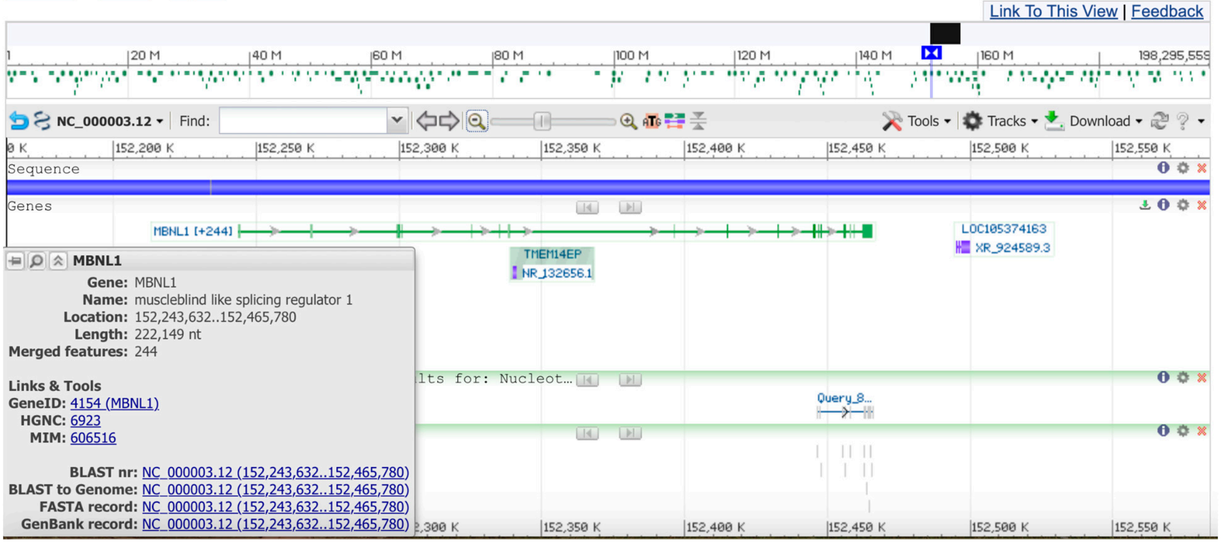

Homo sapiens chromosome 3, GRCh38.p14 Primary Assembly

NCBI Reference Sequence: NC_000003.12

GenBank FASTA PopSet

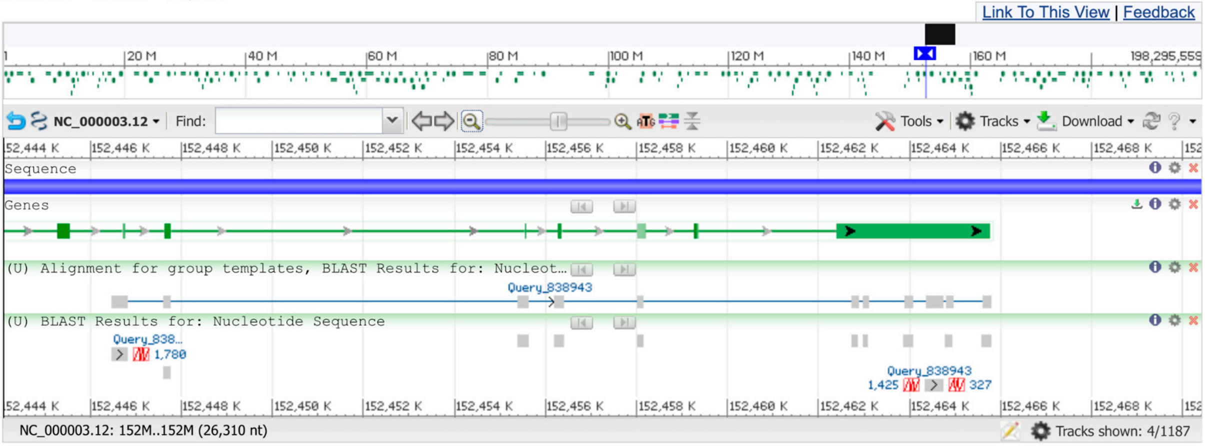

UCE overlaps with exons!

chr3 CLUSTER 21 positions on BED file: 3462 - 3473

>NC_000003.12:181692767-181693055

AGAAATATAACTCCAAATAGTACATCTTTCTGGGTTTGTTTCTAAATTAGTCCCCACACTTAATTATGA
GGATGAGAGGGATGCCTTATTCCTCTTTGTTATAGGCTAAGCTCTCAGTAATCCCAAACAAAAGAGC
CAGTTCTGGAAGATATAGGATTTGCTAGCAGGATTATGGTCTCATAGATAATGATGTGCAGTCAGCA
GAAGCGAagctttattttaattcaaataagcTAGTAAACAAACCACTGACAAGTCTGCTGGTGAGAGTAACTGGCT
GCTGTTGCC

>NC_000003.12:181699161-181699390

CCCCTATTCTATGGCTTCAATAAACAGAACCCATTACCTCACGGCATCTTTTGTTGGCCCCATACAAG
AGAGAACAAAATGGTTATTCAGGGGAACGCTTTCGATTTTTATTGTGGGTAGCCTGCACAAAGAGCC
GTGTTATTGTATTTAAAAGAACCATCTGTAGCAGAAACAATGGCATCAAATTATCTTTGAATACCCACT
GAAAAaacataaagcttttttttttt
>NC_000003.12:181699534-181699640
ctctctcttttaaaaaatcagccatttGGGGTTTTAAGCCATAAACGATTTTTCTTGTTGCAGGGGATTGCAGTGGCA
AAGCTAGGCTAGGTCTTGGAGGCTGGTG
>NC_000003.12:181699823-181699925
TCGCTTTTTTACCTGCCAATCAAACTGCTACAAGACAACACCCTGATCTGGCATGGACATGTAAGTAG
CTTGCAACCCAACTTTGACATTCACTGCTAGCTC
>NC_000003.12:181701240-181701415
TGATTGACAAGAGAAAAGGTGTTGAGGGCAGGTCGCGCTGTCTTATTTACAGCCCACTGCTTGCTGT
AGAGTCTTTGTTAGGACTGGAAAGCTAATTTAGTGCTGATCAAAAGCACCACAGAGTTAAAGGACTG
GTAATGACTGCTAATTTCATTTCAGCCTCATGCATCCCCAA
>NC_000003.12:181707882-181708010
tttttcagaaaattgagTTATCAAGGCAGTAATTATTTCACGGGGAGATAAAACTCTCATAGCCCTAACTGTCA
AATAGGGCCCTTTTCagattttaattacaaaataaaattagtctgCTCTTCCTC
>NC_000003.12:181708042-181708282
CCCCCAATACTGGTGGTCGTCAAACTCTGCTAATTAGCAATGCTGAGAAATTCCAGTTAACAAGGAC
ATTCTCCAAGACTCTGCAGGTTCCCTGCCGTTTGCCTTCATTTCCATAAGAAGATTAAGAGAGGAGG
GGAACACACTCAAATGCAGATGCAGAAAAGAAGCGTTTTTTAACAAGCATCATAATAGTAAGATGCTT
GGCTAGTTCTCACCTAATTACTGCAAGTTAAACCTCTA
>NC_000003.12:181711914-181712032
CCGTCACATGGATGGTTGTCTATTAACTTGTTCAAAAAAGTATCAGGAGTTGTCAAGGCAGAGAAGA
GAGTGTTTGCAAAAGGGGGAAAGTAGTTTGCTGCCTCTTTAAGACTAGGac
>NC_000003.12:181712484-181712590
AGCGGCCCATGAATGCCTTCATGGTGTGGTCCCGCGGGCAGCGGCGCAAGATGGCCCAGGAGAAC
CCCAAGATGCACAACTCGGAGATCAGCAAGCGCCTGGGCGC
>NC_000003.12:181713754-181713881
aaaaaaatgttttaatatttgcaaGCAACTTTTGTACAGTATTTATCGAGATAAACATGGCAATCAAAATGTCCATT
GTTTATAAGCTGAgaatttgccaatatttttcAAGGAGAGGCTTCTTGCT
>NC_000003.12:181714318-181714431
AATCCATGTATATATTTGAACTAATATCATCCTTATAACAGGTACATTTTCAACTTAAGTTTTTACTCCA
TTATGCACAGTTTgagataaataaatttttgaaatatggACAC
>NC_000003.12:181722132-181722316
ttttttttttttggctgtcaCTGGCTGGCAAtactcattttgtgtgtgtgtgtgttggtttgAAAAGCAGGAAGAAATGTAATGAATTG
AGTGCTGTCAGAGATGTCTTCTGTTTGAACAATCTGCGATGGGCTGAATGGCAGGGACTCCCTCATC
CAGTGCAGGTGCAGTAGAgctggga

Homo sapiens chromosome 3, GRCh38.p14 Primary Assembly

NCBI Reference Sequence: NC_000003.12

GenBank FASTA PopSet

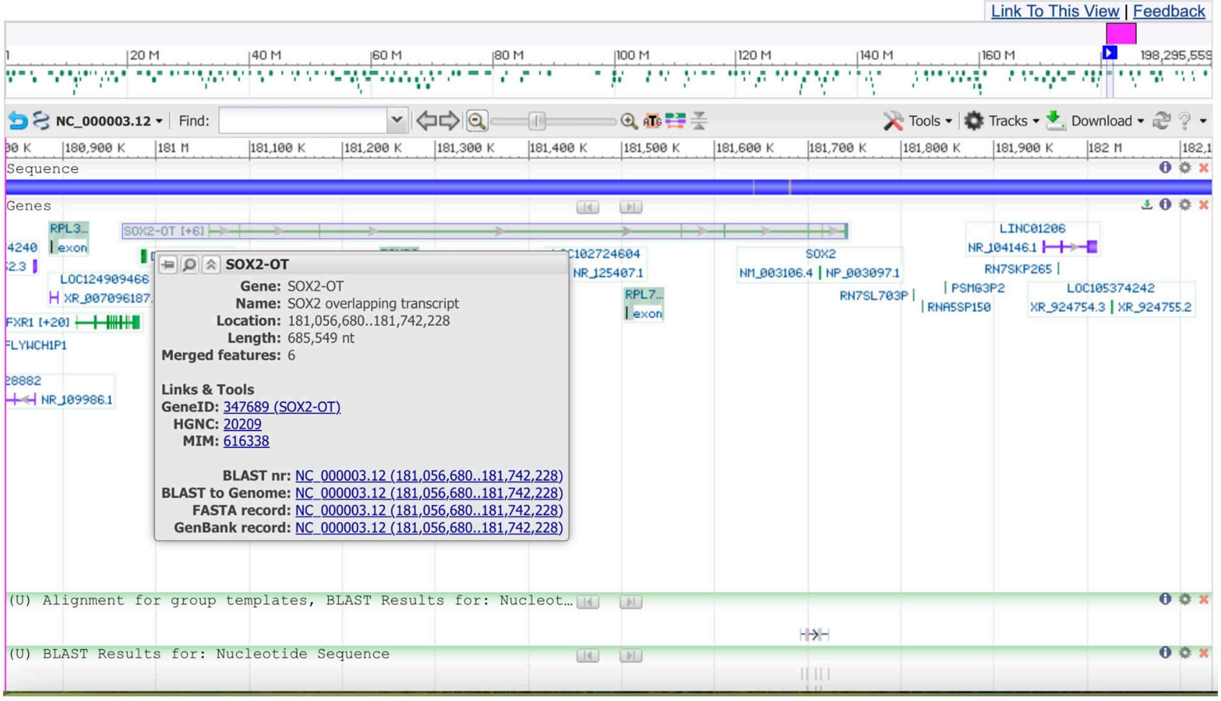

Homo sapiens chromosome 3, GRCh38.p14 Primary Assembly

NCBI Reference Sequence: NC_000003.12

GenBank FASTA PopSet

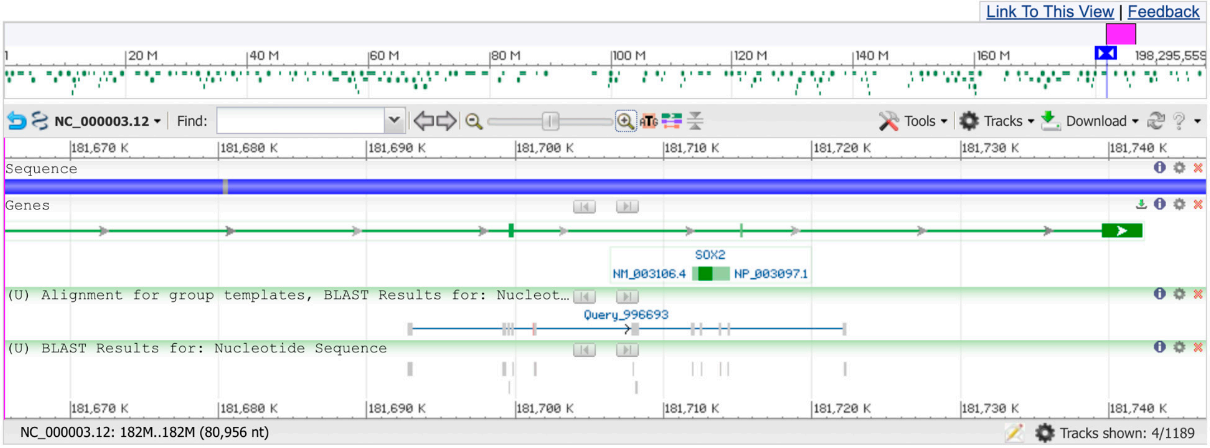

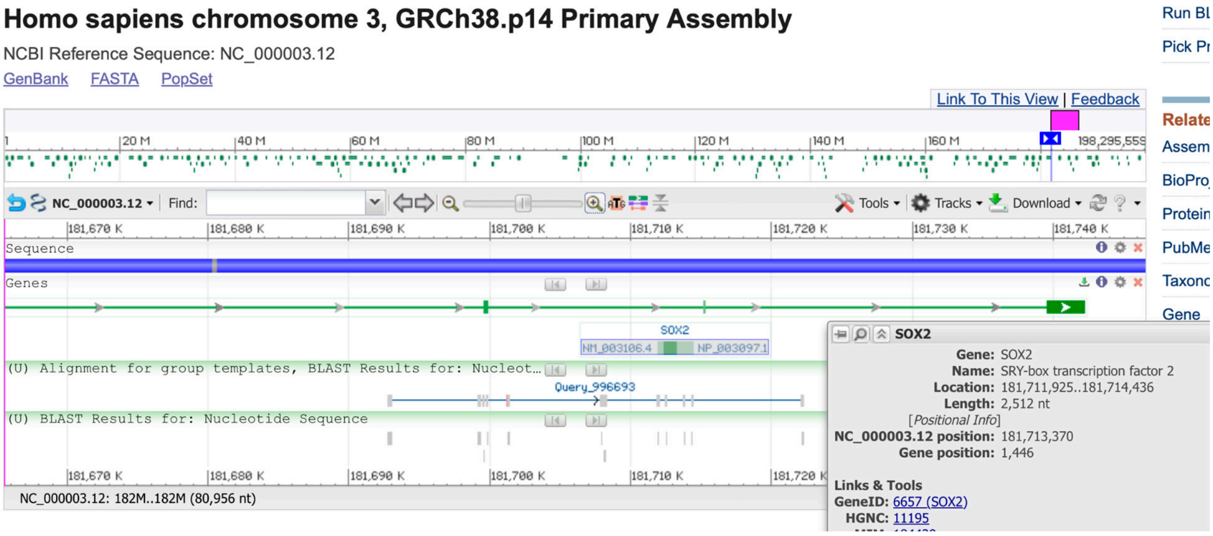

UCEs overlap with SOX2 exons

chr5 CLUSTER 22 positions on BED file: 4185 - 4195

>NC_000005.10:77628825-77628938
AATTCCATAAATAAACACAGACATTAAGTTAAAACATATCACAAATTGACCAGTATGTAACAAGAATGC
TTTATCTACACAAAACATCCTATTTCACATATTTGTTCATTCTT
>NC_000005.10:77633849-77634091
GGAGGAAATCTGGGGAGCTGTATTAAAGCCTAGATCTCAGGTTCATTTCGATCAGCCAGCCGTGAAC
TCTGGGCCGAATTCATTACAGTTTAATAGTgctgggagaggaagaaaatgcaATTTCTCATTCcattagaaaactaga
aaatactcTCAGTTTGTCCCCCTATTAAGATGTGGCAGGTGACAGGGCCATTCTGGGGGACACGGTCAA
TGGAATTACAGCACACTATTTTTgttca
>NC_000005.10:77634226-77634350
CATAATTTGCATGATTCTCACATTGCTGCTTAATAAGCCATTCCAGGTTATAAATAATTCTGAAATTGG
TTTCTAATAATGGCATGCTATAAAAGGAATGGTTTTATTACACTGGCCAGAAAAG
>NC_000005.10:77638491-77638825
GGGCGAGAGGCGCGCACTCACCTAGCCTGGCGTCCAGGAGGTCGGCATGAGACAGCATCGCGCAC
CGCTCCAGGGCGAAAGCTGTTCCCCCCCAAATTTTAGCggctttaagttatttaaaatagatataagCTATAAGCTA
TacgatatagatatatatagatcacctaaatgaaagaaaattcgGTTTGTGGTTAAAAAGGGGGCTTCTTGCATTATAATG
TCCTTTAGAGAGACGGGGAGGTGCTTAACAGTTGAATGAACCCGTGAGCAGCTCGGCGAGGGGACC
AATCAGGAGGGCAGCCTGCAAATGTCAGCGCCCGGAGAGTCCCCC
>NC_000005.10:77639546-77639814
ttattttcctcttgaaTATGTTAAACTACTTGAACAATTTAAAGTGCTTTGCACAAGTGAAGCGAACCATTTCTAA
TGTTCTGATTTTTCAGAGCCAGCCAACAACAAAGTTTAGATTAGTAATATATTTCTAACCAGAGTAATT
TTCAAGATCATTAGGCATTTATGTAATTAGTGCCAAATCTATAAGCTTTTATTACGATTATTAGAGTAAA
ATCAGTATAAATTTGTACTAATTTACTACTGTTTAGACTTGGCTGTCAAGCCT
>NC_000005.10:77639815-77639915
GAGGTTCTTATTGTAAATGATAACTGAGGAAATTAAGTGCAAAATTCTGTACCATTTCTGATCTGCTCC
TAAACAATCCGTTTCAATTGTATTTGTAGCA
>NC_000005.10:77641174-77641399
tgtaTCTTTCAGGTCGATAAATGAATGTGTAATAGTTAATGTGAAGACACATCAGAGTAATAACAATGGG
ACGAAATCAAAGGATTATCCATCTCTGTTAGTAACCGCTGAATAACAATGTTGCGGCATGATTGATAG

CCCGCTTCATTTTATGACTTTTCTATTGGTCTTGATTTTTATAGCAGTATAAACAAACTAAATCATTTCT
TGCAAGACTTCAGAGCA
>NC_000005.10:77641797-77642047
CAATGGCCCCCAAATCTTCGGAATGACAAGAATGTCTTCAGAAATCACTTCAAAGCAAAGTAAAAGGA
TTTTCTATGACGGATCCAGTGATTTTTCCTCAATAAATAGGTTTTAGATTGTTACACACACGTAGAAGT
GGAACAAAGGGAACTACCAGAAAATATATTGGTTGCccctaataaaatttatttagacAGTTTACTTCTCATAAAA
AATAGAGATTGAATTTTGTACAAGATCCTGCCAAGGG
>NC_000005.10:77644307-77644507
TTATCTCTTTCTATAACTAGATATAGCGCTAATAAGACTGCATCAATCTTatctagcaaataaataaaatagacaa
acaaacaaattcgCCTCCAACAAGTTAATGCGGTGTATCTGAGTGGAAAGTAGATATTTTCACGCATTCTG
GAGATGAGGAGTGACTTTTaataaaatcacaagaaaaacaaatgtaattctGT
>NC_000005.10:77645059-77645224
GCGGCTAATTCCGGGCGTCTATATTCACTCAATTAGAGAAATCTACAGAGAAGATCGATCTTCCATCT
GCAGACATGCCAGCTTAACAAATTAGATTCTTGTTTCGTGCAGGTGATTTGGTGACAGTTGGGGAATT
AGAAGTAATAAGTTGTTGTGTTTGAGCCC
>NC_000005.10:77645330-77645550
gccgtcgccgCATCCCCCGCCGTAATTAGTGCTGCTGCCCTCCATGTGGGCTCGATTAAACCGTGATTTA
GCcgaaagaaatataattatggctgcaaataaaataatcagcATTGAAGAGCGATTTCCTTAATGAGATGGAGCGGTTG
CACGTCACGGAGTAAAGGGGTCTCATTAAGAGGTGGTAATGAGGCTTGGGTGGATGGTGCAGTTAC
AAAAT

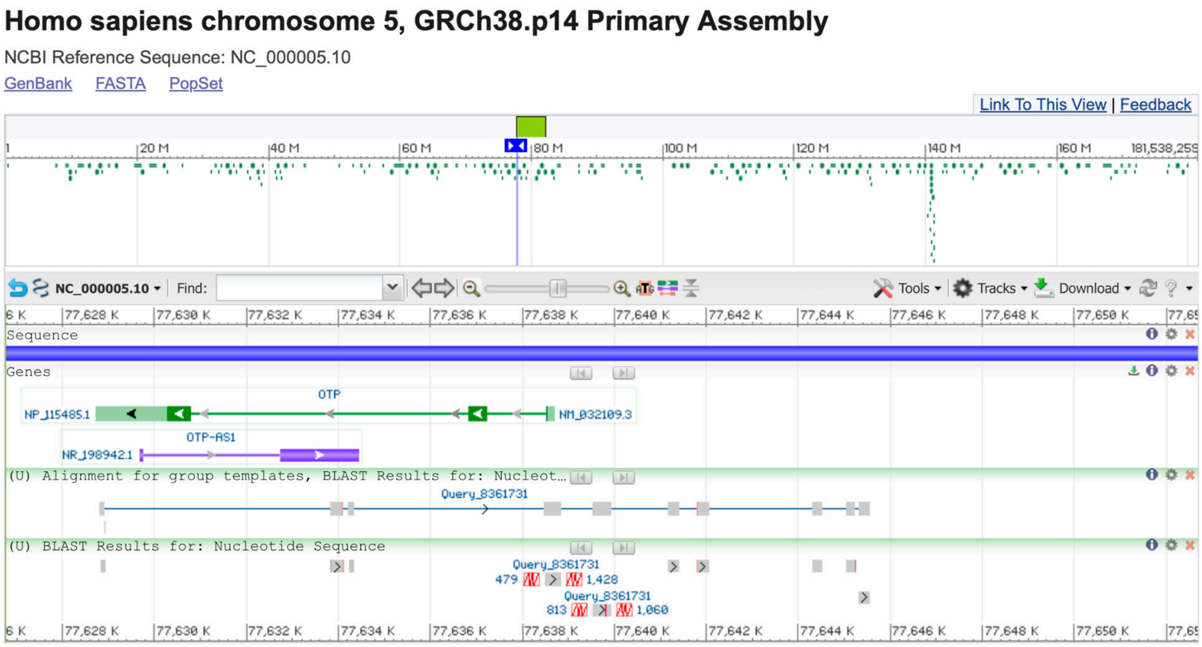

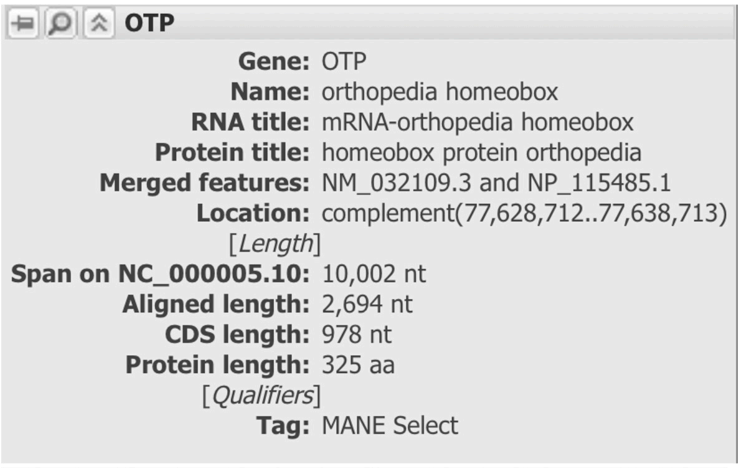

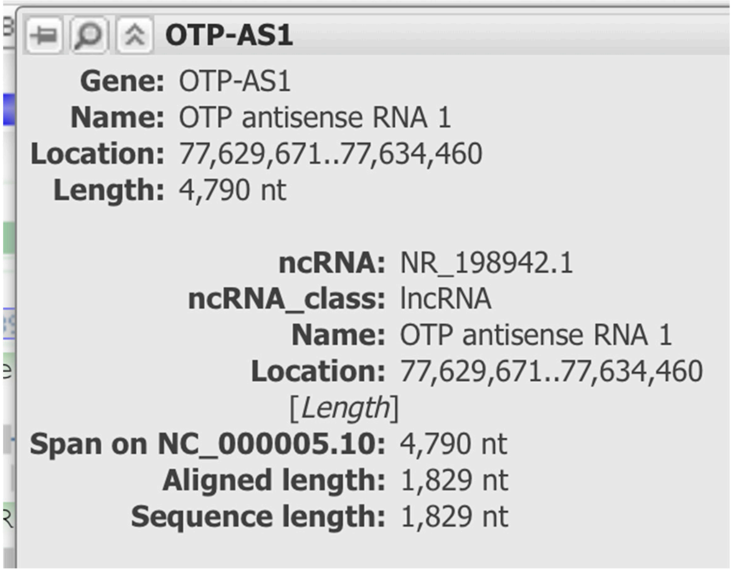

chr5 CLUSTER 23 positions on BED file: 4201 - 4211

>NC_000005.10:77845272-77845407
GAGTTCTCATTATTGAGGAGTTTCATaactctttaattcatttttaatgatgGCAATAAACATGGTAGTCATTTTGGAA
CAATAGCTAAAAATCATAGGGCCATTACTGTCCATTAAAACAGTGTTTACAGTTCA
>NC_000005.10:77847682-77848147
ACTTCCTTGCTCTTTGAGAGAGCAGCGGTATAAATTGGCtctaataaatattagctgctagATTAGGCGAGGTGA
AGCTGGCACCGTGCTCTATTTTCGACCTGTAGCCTCTGCGAAGCCGCCAGTTCTGCGCCAGATTAG
CCCCATCCATCCAGTGTCAATGGTTGTCTTGTTGAGAAAATCAGTGAGTAAACAGGGCTGACAGAGA
GGGGGCCCAGCTGATTACTCATACAAATAGACTGCAAACTCCTCCAGGAGAAGCTGTTTCCAGAGAT
GGGTTTCTCGCATTGACAAGGCTGCTTCTCCCAGGATCCTGTGTCCTGCTGTTCCCACTTTGTCCTC
CATCAATCACACAATTGTGTCCGGGATGTTTTATAGAGTCACTTAATATCCCTGCCTTCATCTCTATTC
ACTTAGAGAATCATCTGCCATCAGGGCTAAAGGCTTTCTGCAGGATAGGACTGG
>NC_000005.10:77850046-77850258
TTTAATGAAAGAGGTTCCTGTACCACATTATTAGCAATAAATCTACACCATTCTTTGTTGGTTAAAATC
ATTATGATCATCATCGCTATCATTACTGAACTCATTATTCTTGTGTTACTATCAGAGTAGATTTATGATA

ATACCCACCATTACGCCTGAGTTAAAGAAGGTGTAAAGGTTTGATTAATAGCTGCTTTCATGTTTTAAT
ATTc
>NC_000005.10:77850860-77851097
CTCGCTTTGTCTTCCTCGTGCCCTTTCCTGGCAAAAATGGAGACATCACATCCTTGCCCAGCCCCAG
CTCAGTATTTATGAAGGTTTTCTGACAAATAGGCAGGCTGGCCACGCATGGGTGACTGCCTTCATTC
CGCACTTGGCTTGTTTACATAGGACCAGCACCATTTCCACGGATTAAACAAATCACAGGGAGAAGGA
GACATTTATAACCCGCCAACGAGGCCACAGGCAGTT
>NC_000005.10:77852100-77852588
TTCCCTGCAAAATAGCAATGGTTCCGAGAAAAGGATCAcaacaaaatattgaataaattccctttaatttatttgatcatTC
CACATTACGGGGCTATGAATTGCTCAAATTTGATAACAAGTACTCAATTCAACTCATTATGTAAGTTAA
ATTAGACTTCGATAAAATTTCAGTGGCGCGCAGTGGTTCCTCGAATGAAATGTAATTTGTGCAAGAAT
TTGTTGTATCTGTCACAAATATTTCCAATTAGCGACCGTAATTATTTGATAGTTTCTGCAATTATGGCT
TAAACGGGTGAATTTGGGACTGTGGAGGACCCTAATTGGCCTAAATTTGATATTTTGCATATTTGCAT
AATGAGGAAATTGGTAGAAAattgtgttcattcatttgtgaAAAATCCTATGAATTTTATGAATTTGAAAGCTCTGT
TTGTGCAGACTGTGACTTTGCCTGCTGATCCCTGAGGAGCTGACGCTGAACGCC
>NC_000005.10:77853024-77853128
TCTCAGTAAAAATGTTTGATCTATGGCGCTGTCAATTTAAAGTGTTTAAAGAGAAGCAAAAGCATAGA
TTTTAAGCTGCTTTGCCCAGCACCCATTCATCATGC
>NC_000005.10:77868761-77868875
GAAATAAACTTGCGCACCTCATAAAAAGAACCAGGAATGATGCTACCATTTCATGGGATATTACACCA
TAATGATGATAATCCTCCCATTTACATCTGATCTTCCACAGACTTA
>NC_000005.10:77869142-77869472
AGGCTGTGAATTTCTTCTGCCAACCTGAGCTGAAAGAAGCTAGAAAACTCTGCGTATTTTACTTTCAG
AGTTAATGGGAATAAAATACCTCAGTGGATGAGTGCAGATGTGTTTGATACAAATAGTCATTTTCCTA
TTTGCTGCCTCATTGAGCATGATGAATGATTGGAGTCAGAGAAGCGGCGTGATAAATGGCAGCACCT
TGGCTCCATTGCATGCCCTATTGATTCTCCTTCTTTATTACTCCTACAACCCAGCTGGTTGCTTTGCC
CTCCCCACAAGCCGTTTCTTGGGGGAGGGATTGCTTCCTAGAAACCCAATTTATGGAGA
>NC_000005.10:77874466-77874605
GCTGGGAATAATGAACCGCTGCACCCCATTGAAATGTAATTACACAGCCCTCTTGTTCTTAAACCTAT
AAGGTGAAAGAAAGATGAATAGTGAAAGTAAAAACCGCTGACCCTTCAAACTTTAATTTACATTATGC
ATT
>NC_000005.10:77874606-77874743
GCTCGCCCTTCTGATAGGTTTTAGAAAGGTCATAATTTAGGGTATTTTGCCATTGATAAATGATCTGTA
TGTTTGCGTTCACACGCACCGATTGATTTGTTATAGCCAAAATATTGTTTCTATAACAACCCAAGGGC
>NC_000005.10:77875103-77875459
GGGCCCAGTTGATGCATTATGCATCAGGGCTGCCTGCAACTGACAGCCCCTCAGCCAACGTCGACT
TGAAAAGCAATGGTGCTAATCAAAGCCGACACATTTGAAtgtccagacacacacacagagaaatttaCTAATCAAA
AAGTGTATTTCCTAGGGCTGTCTCCAGAGAAATCCGCGAGAAGAAATTGGAATAATTTATTAGCTTAC
AAGTTTTCGTTGACAGTTAGCCCAAAGCTATTTATCTCTGTAATAGAGAACGTCTCCAATGTGGCAAA
ATTGACCAAATCAAACAGCTTTGATCAGAAAATGAATCTGGCAAGGCTGCCTGTCGGAGGGTCCCAG
GAGAAAgaggg

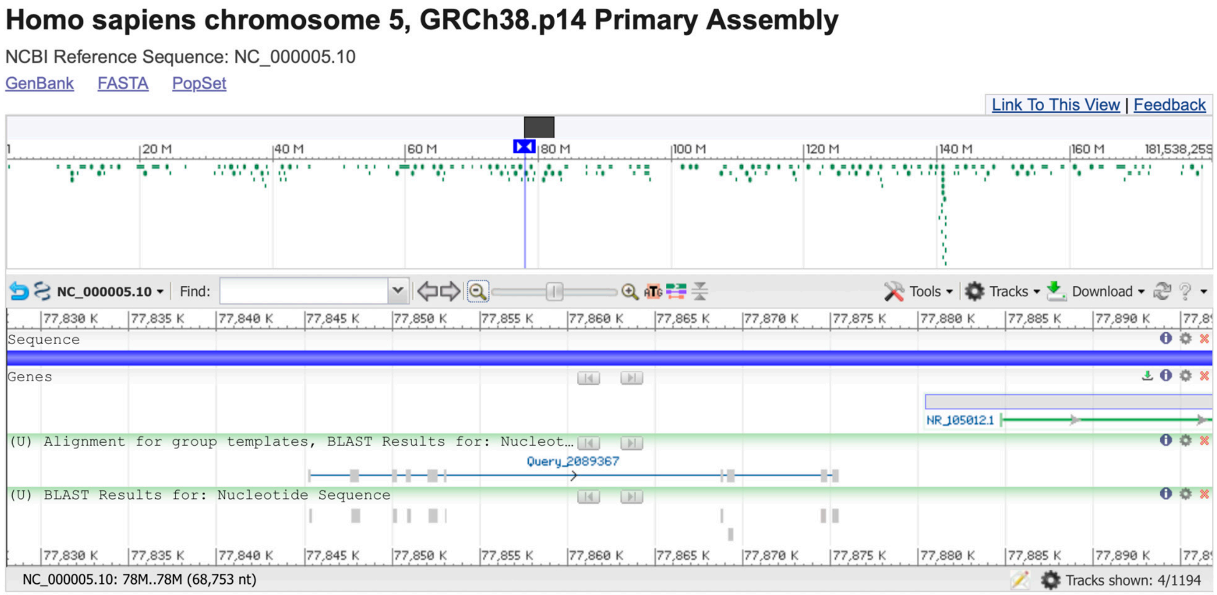

INTERGENIC region second time!

chr5 CLUSTER 24 positions on BED file: 4399 - 4409

>NC_000005.10:93573880-93574124
AAGTGCTATTAGATTTATGGTCTCTTTTTCGACACCCATCCAGAGTAATTAGCACATATGTTCTAAATA
GATGATAGTTTTGTGAGCAATAAAGCAATTACCCATCGTTGGAGCTGACAGTTCCTCCAACTAAACTC
CAACCAGCAGCTAATTGGAAAAGTCATTTCAGCTCCATTGTCTGCAATTAGTCCTGCTAAACTACTAC
ACCCCAACTAGCTGGTTTGAGTTTAATTTATTCAATGTT
>NC_000005.10:93583044-93583267
CCCCCCGATTCAGGGCTCTGACCAGTCAGTCGCCTTTGATTATCAGTTGCCAGCAGCCCTTCTCTTG
GCTCCTTGACACGAGCTCCATATAAGGCAGCGATCTCCATAGAAACGTGTCAGTTTCAATAGTAGTG
TCAAAGTTCACTATATACAGACATTCGCGCAGATCCCCCTTTCGGAAACATTGCTCTGCGAGTCCTCC
CGTTTAAACGCATTCAATTtt
>NC_000005.10:93584984-93585127
GCAGCTCGGCTCCCCCCAGCGCTCCCCGGGCCCAAAGATATGGCAATGGTAGTTAGCAGCTGGCG
AGATCCGCAGGACGACGTGGCCGGGGGCAACCCCGGCGGCCCCAACCCCGCAGCGCAGGCggccc
gcggcggcggcgg
>NC_000005.10:93585272-93585514
ATCGAGTGCGTGGTGTGCGGGGACAAGTCGAGCGGCAAGCACTACGGCCAATTCACCTGCGAGGG
CTGCAAAAGTTTCTTCAAGAGGAGCGTCCGCAGGAACTTAACTTACACATGCCGTGCCAACAGGAAC
TGTCCCATCGACCAGCACCACCGCAACCAGTGCCAATACTGCCGCCTCAAGAAGTGCCTCAAAGTG
GGCATGAGGCGGGAAGGTGAATAtttcttctctgcttctctccc
>NC_000005.10:93587046-93587187
aaaaaaaaaaaactttggtcAGCTTTAAGAACTCAAAGCATGCTTACGTTTTGCATTCAGATGGTTAATGAATC
CAGTGTTTTTGCAGTTGAAAGCTCGTGCATTCACTGGGCAAAAGCTGGCTGCAGGTTGCAGTGGCAC
>NC_000005.10:93594408-93594611
aaaaaattgtcctgtgtctatgtatctatatctgttttgtattttttctggttccaaACCAGATTTCCTGTGATTCTATACTAATAATTTT
TGATATAACCCTTTGCTTCTTATAATGAGTGCGATATATGTTGTCGAGGCTGTTCTTCAAGAATTAAAA

TTGAagtgaaaatttaaacaaaaataaaagaatttagcaAAA
>NC_000005.10:93598195-93598400
AAGCCAAAGAGTTGTATCCCTAAATGAGTTAAAGACCCATTTACCTCTGTCGTGTCCTATTCATCAAG
TCACAAGTTGAGAGTTCCTAACATGAAACTATTTGTTTGCTTACACAACCAGCTGGGCGCAATTTCAC
GTCTGAGATATCTATCTGAAAGCGCCTCTCATTTATTATCTCTCCTTGTCAGCGAATCCATTTTCCAGC
>NC_000005.10:93598931-93599031
GCTGCACGTGATTTCAATTTTGAAGCACAAAGAAATAACATGCAATGCAGAAAATAGGTTTATGAATG
TAattgagaggaaagaaaacaattaacacgGT
>NC_000005.10:93600530-93600678
CAGCttgctttgttgttttaaaatatgcatgtcTCTCACTGGAATGCCAGTCACGAGTGCAAACTTTGTGTCCAGCCTC
CCACATGAAATGATCCTTTTAAAGGGAACTCGATATGTCTCAATCTGCACTTCACTCAAAGCCTCTGG
G
>NC_000005.10:93602048-93602155
CTCCTCGATTTTACTCGGCGGCACTTTGTTGCAATTACGCTCCATGCTGAGACCAATTTTATTGGTGA
CCTATTTTTCATATTTGCAgcattttatgttaaaaacct
>NC_000005.10:93603431-93603650
AAAGTTTagttgggctctttttgttgttgtttaattccACTATTAGGAAATGAGGCCTACctttgaattatttcattatgatttggccttttggg
ggaaggggtgggaacTATAATCATTAGtggctttttattatttctacaaTAAATGGCTAGATTCTTTGTTGAATAAGCTGC
TTGAATGTTTTGCAGATGTTCCACTTAGACCAAATCTGACCC

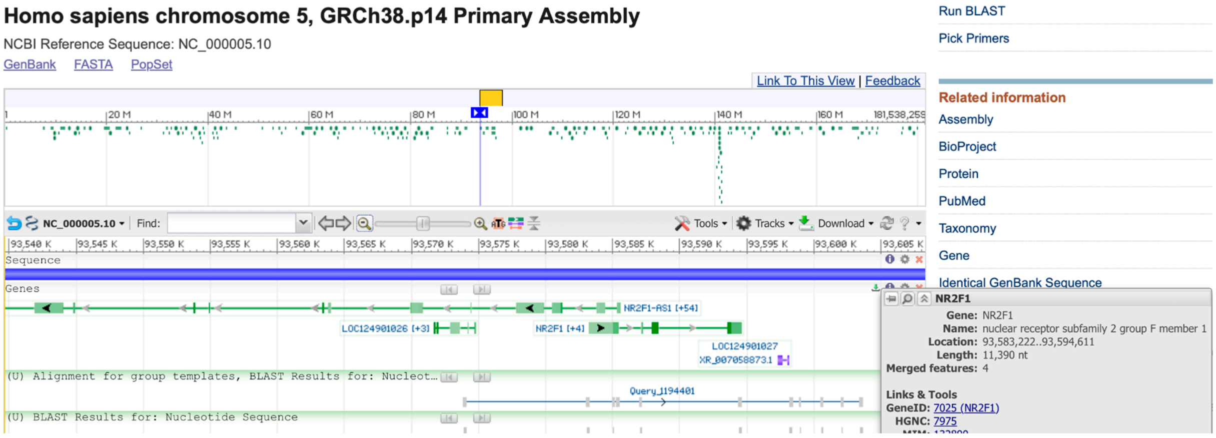

Homo sapiens chromosome 5, GRCh38.p14 Primary Assembly

NCBI Reference Sequence: NC_000005.10

GenBank FASTA PopSet

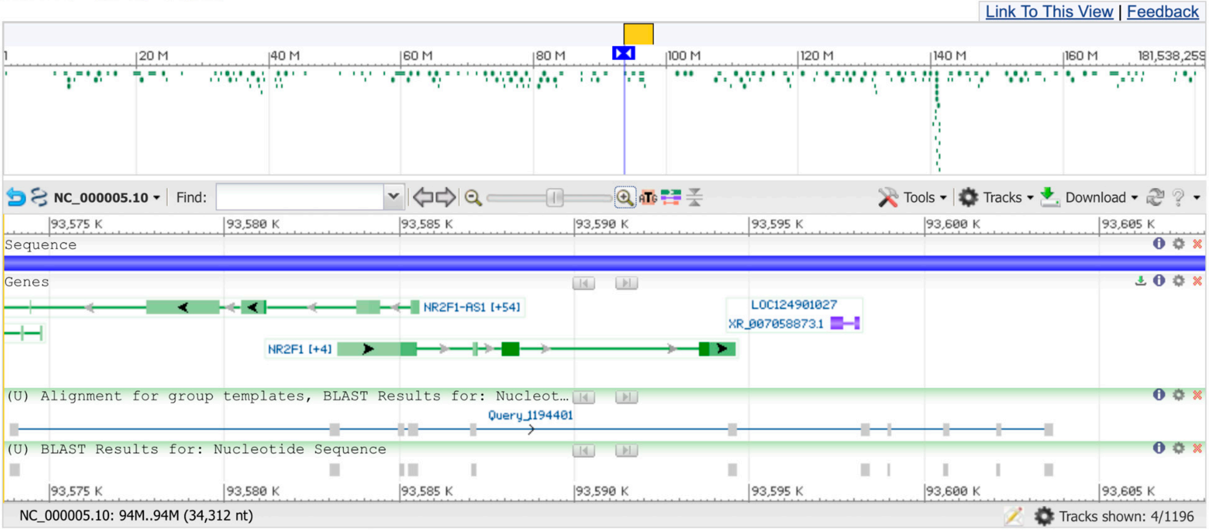

chr5 CLUSTER 25 positions on BED file: 4417 - 4427
>NC_000005.10:93883496-93883608
AGCTATTCAAAAGTCTTGTAATTCATTGTGATCACTGTCAGGTAGACAGATTGTCACTTGGTGATTTTC
TACAGTTGGACACACTTGACTAGTTATTAAAATCACATTTGAA
>NC_000005.10:93886973-93887268
CTTCAAATAGCTCATCAAGTTAGTCAGTAAAAGAAAACATGCCACAAAAAGTTTTCAGAGGACCACCT
GTTGtctattatttttacatagcACTTACTCATAAATCATAACACAGGGTAATTAATCAAGCTGTCAAAGAGAAGG
TCAAAGGTTATATGGTAGAGTCAGAAGGTCATGCATGGGCTGATAGAGGTCAGGGTCACACTGCATT
CCTCATTATCAAGTTGGGATGAGAGACGGCCCAAGCAAACAAAAGTGAAAGCAGATGGAGAAGtgagt
ggataaaaaaaaaaaa
>NC_000005.10:93887761-93887993
TGGCCTGAATTAGATGCAGAATACTGTGCAAAATTAGCTGTCACCCTTTGCTCAAAATCTTTGTTAAT
CTCATTTTGAATGTTTGCAGCTTTATCAACACATGCATCAATGTCAGCTCCTTGGTTACTGAACCGCT
GTAGTTGGAAgaagatataattaaaatgttttggcACACAAAAGGTGAAATTgtatcttcccttgttcccttaGGAGTCTGTAAA
ACATGAAGGAT
>NC_000005.10:93889594-93889708
TGTCCCCAAgaacatgaaaatattaataatttatattgctCATTAGATGGCAAAAACTCTTTAAACAGATTTAGAGACA
GATTAATTTTATAATCACAAAATGCTACACTACCT
>NC_000005.10:93890632-93891140
GATTTTAAACTAATGAACACAGTGACACAGGGCTCTGAAGTAAGCCATGCATAGTTCTAAGCCAACCT
TAGAATTTCAACCCTTCCAGCCCTGGCTTAGATCATACAGGCTCTACTAGAAATGTCAAGAATGTGAA
GAGGCGTCTTTAGCCAAAAGGACACAACTTTAAGTACATAATGCTAGCTTACACTGGTTCGTTTATTC
CCCTCTTCATTAGTGAGAAAATGGACTCTCGTAAATGCTTGTTAAAATCCCTACCTACACTCCTCTGG
TTAATGTGAGTGATCCATTTGTGTTCATTTCTGCCTGACAACGCTTACTGGAAGATTAATTGCCCCAT
GTCAAACTGTAGCAACCCTTGTccccttctcatttccttttctctatgcTGTTGAAGTAATAATGGACCTTGTCTTTTGT
TTTAAGACTGGGATTAGGAGAGAGTGAGTTTTTCTTCAGACGACTTTATGGAGTAGAGTTTGGTTGAA
TACTTATAAGATTTACCGTG

>NC_000005.10:93891605-93892481
gGAACATTAAGGAGTTTTATGTTATCACAAAACCTCAGAAACTACCAAGCAGAAAAGAAACCTTATTAT
GAGTAACTGGCAGTATCTCATTAAATCTTTCAATTGTCCAATCGTCCACAGCAGACCTCCAAGAGACT
TGTATATTATACTCATTGCAATTAACCAAACAAAAGATTTTAACCGCCAGAGCATTCGGGAAATGTTTG
GTTGAGGCTGAAGAAGTGAAATTATATTCCAGGGTTGGCCAGATGTCACAAGGGGTGATATGCATGT
GCTCATTTCATCTGCAGCTTTGTGCTGGACCTGTCTATTTACAGCACTACAGCTAAGCACTCTGAAGG
CCTATTCACTCATGAATCCTTTCAGAAAGTGCTGAAGCACCCCTTAAGCCCACTTAACTACCATTTTC
ACACACTCTCCCAGCTCTCCTTTTTGTCCTTGCTTACATTACATCAAACACAGGAACAAAGCAAGAGA
ACAGAAACTCAGAGGCAGAGAATAGACCCATcacaaatattaatttgaaaaggTGTTGAAGTGCAGAATCTGCT
TTTATGCACAAGGACAACTTGCATTTTTTGTGTGAGATTCTCTTAGCTGCAATAAGCTAGGTTTTCAGC
CAAAGAGAGGCAAAGACTCAAAGTGCAATTATACACAGGGAACTGCTTCAAATCAAACAATGCTCCG
AACTGCTTTAGATCTATAGTGATAAAGACTTGGCAAGCACTATTAAATAGAAGCCCTATATGAGATGC
AGAGTTCACTCTATGGATGCATAcaaaagagaatacaaaaagaaTACTTTTCACACAAAAGTAAAACTACAAT
TTCACTTTTAATTCACTTGCAAACAACACTTtaatacaatttcttttt
>NC_000005.10:93893009-93893549
ATTATTGGTGCAACAGTTCCGATTAAATTGAAATATTACTACCTTTCTTCCATGAAAATACATGGTTCT
TTTTATCACCACCTTCTGTCTGATTAAAAAATGCCATAAAacctaattttaatttaaatccaGCCTGATTTGCAGGT
GTTCCTGGGGGTTACAAATTTATCAGGAACATAACTGCCACTGCCGAGATCAAAACACTGCACAGCA
CGCCAGCAACTTCATGGGCCACAGCCACTCAATGGGGAGCGGCCTCCTCACAGGCTTTATGATGAA
ATACAAAACAGCCAGCAGGAATCTATATCTTATTCAGACCTGAAGAAAATGTTGCCCCCTACCCTCCC
CcaaaatgcatacacacatacaattacTGATAATCTGCTTACACTGGCAGACTGTGGTGGGTTTGCTAACATTAA
CTGCAAACAGTCTTTTGACAGAAAATACGACTAGTCAACGGGTGTCCAGTAATGGTTAAATGAATGCC
ATGTTATTATCTACCACACatatagaacaaaataaaaaagtagaatgaaT
>NC_000005.10:93894541-93894892
GTACGTCTGATCTCTCAAAACCTTCAAAATCTATCCTTGATCTCAGCAAACAGAGCAGAGCACAAtgttat
ttatgcattcatttatcttttcatcaAGGGTGGCTTATGTGGTCTTTACTGACTTTGGTTTCTCATCATCATCGCAGAT
CAGCATCTGCATTGTGTGCACTTGTAACAGGGATAATGGATCTCACTTACAATACCTACAAACTCAGC
TGAGAGCCGTGCAAAGCAAATGGCCGTAAATGCACCCATCCTGGCCCAAGTCctgatgaataaaaatgaagat
cTTGTCAAATAATAAAGTGACAGGTTAATTTACAGCATGGAATAGTGTGAACAGGGAAAG
>NC_000005.10:93895098-93895263
GGGCTGCTGTATCAAACAGTAATAAAGAGCCTCAAGCAGTTGTCTGTGCTCATGCCAGTTTTCATGC
TTTAATTCAGCTGCCCTGTGGTTCTTAGCTTCAACATTGAGATCTGCACGGCCACCAGAGATAATTAT
TTCAACCTTCAGGCCATCAGGCGTTCATTT
>NC_000005.10:93902433-93902582
ATAAAGGGTAAATAAAGCAGCCAGGGCTTGAGATAGGGTACAAAGAACGTTTCCATGGTTACATGCT
TTCCCATATCAAATTAGAGCAGAATTTGTGTACAAGAGGTTTGTTGACAGCCTACATAGGCCATAAAA
AGAATAGAAGGCTA
>NC_000005.10:93912082-93912190
aaaaaaatcaattctagattgatttttgtgtatatatcgTTATTGTCAGAGGATTAATTGATGAATTTTTCATTTGATGAGTC
AGTTGGAATAAGCTCTGAGGCACTT

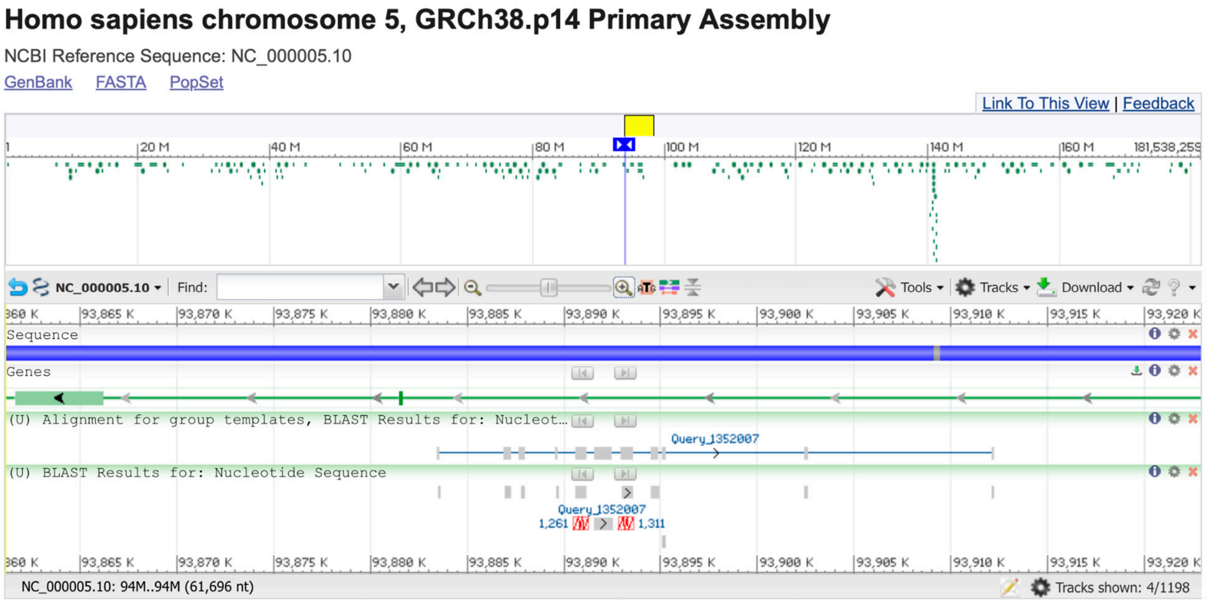

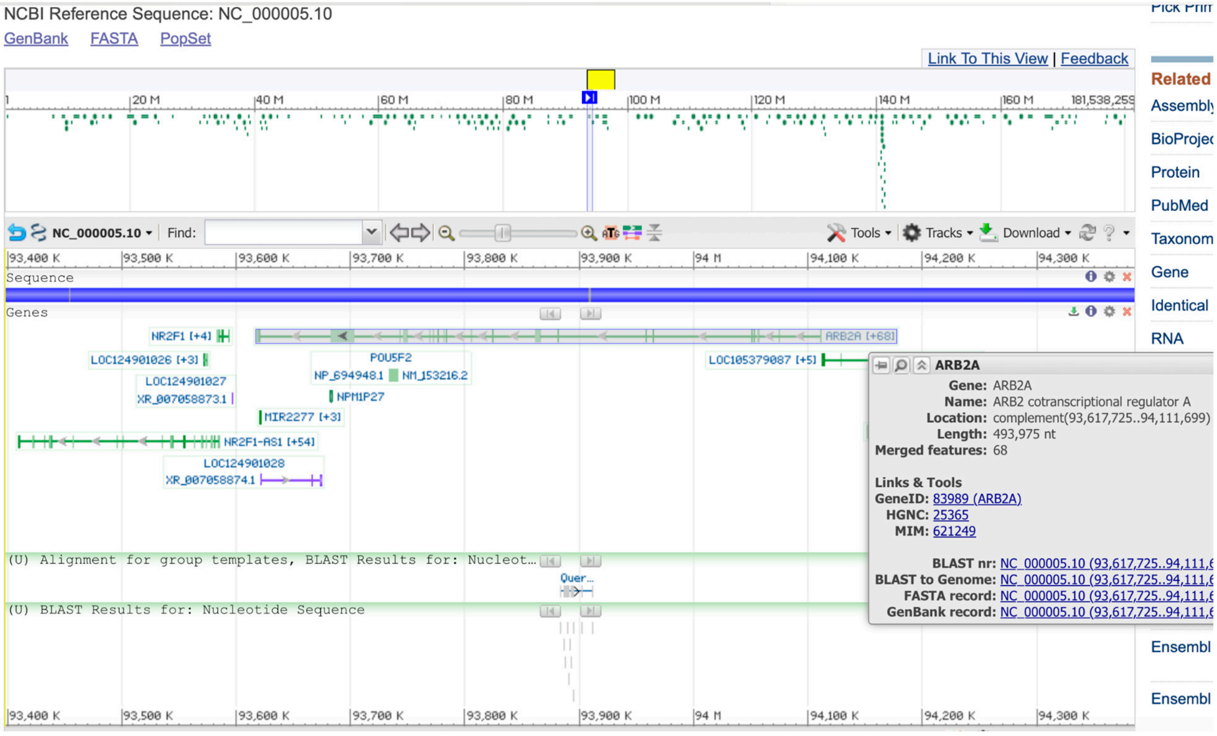

chr6 CLUSTER 26 positions on BED file: 4897 - 4907

>NC_000006.12:10381071-10381219

tcccttcaGCTGCAATGATGTATGTTACTTTTTGGTAACTGGATTTCATTAACATTTATGAACTCTCATAAAGTAGTAGAAAAAGCAATTTGTGTGGAAGAATTTTCCACCTCATTAAACAGTGTTCTTTTGGGGGTCAAGCtgatat

>NC_000006.12:10390012-10390115

TCGCAGGGGCTGGGACTTGATAAAAGGAGacagttttctgaaaagatttgaTTGAAATGGCGTGTGCCAGGGCT
GATGGGAGCCAGCGAGGGACAAAGCGCCG
>NC_000006.12:10390116-10390245
GAATCCATGGACACTCGAGCAATTATGCCTCCACGCTGAAGGTGGATTAGCGCGCTGGAAAGAAGC
ATATGTTTGGCCCGGGGCGACACTTCCCCCCGGCTGAGCTTAGAGAATGGGAGCGCGGAGAGC
>NC_000006.12:10394429-10394659
tttggaattttgataaaTGAATATTCCTAGCAAgtgaataaaatgtattcaattaaCTAAAACCCTGTGCACTTCTGAACTG
AAGTGGAATCGACTGTAATTGATTTATTTGAAACCAGAGGTTAAGGACTGAATTAGCTAAGTACAACT
GGGTATTTCAAGCCACTGACTTCTCAAATGTCAAATcccttctattttaaatattatttcagctGCGGGGCATTGCAGT
C
>NC_000006.12:10396823-10397038
AGACACAGTGGAATATCTAAATGATACCCTGCTCTGAACTCCAAGTTGAAAGTaagtttttattatacttgagGG
AAACAATGGGTTCAGCTGCTTATTGCAAGGAGCAATTGCCAAGGGAGAGTTAAACTCAATTACTGTAA
CCATACTGAATAAAAAATACTGCCAAGAACAAAAATACGCCTGGGTAAAGACAGccactgaataaaaaaaa
>NC_000006.12:10400439-10400542
TGCCTTACTTTGTAGCCAGGAGCATGTTTTTTCTTGTCACTTGCTCATTGGGATCGGAATGTTGTCGG
TTGAGAAATTCAGCTACTGCTTTGGCAGGAAATTC
>NC_000006.12:10401055-10401262
TCCACTGAGTCTGTCTGTGTTGTTGATTTTCGAAtctttggttttaatttcctGAACCAGTTGGTTTTTACTGCAGG
GCTTCCTGCCATTAGCTCCAGCTCCGGAAGAACGCATGCGCCAATTAGAGCATCAAAGCCCTCTCC
GCAGAGCTTGTTTCCATAAAATGGAGCGGATCACAAACAATAACAGTTCTCCAGAAACTGCCTC
>NC_000006.12:10405794-10405946
gctctaGGTTTTGGATAGATCACTTGTGACATTAATAGCTCTGGGGAGGCTAATAGAGACAATAGGAGTT
AAACGAACTCTTGAGAGATTACTAATAAAAGAGCCAATTATCATGTCGACTTGGAAAGAGCATCTCTT
AAGATTTATCCCTT
>NC_000006.12:10406749-10406879
TGCCTGTAAGGACATGCTTGGAATGCAGAAGGAAATGGCTTACCTTTCTTAATTACAGTTTGATCTGG
GATGTTAATACCCGGGTCTTCTACATGCTGCAACAAAAGGATACACATGGATGTAAGTGTAT
>NC_000006.12:10407554-10407660
AAACGTGATTAGAAAGTAAAATGAAGTCATGTTTTCTAATGCAGAAACTGACAGGCAATATAATGTCT
GGCTGTGTTTACAAATTAGCACCAGGGCTGCTGAGATA
>NC_000006.12:10407695-10408010
ccatttaaaaaactattgCTTCCTTCTCTAGTCTTTGTCCAACAATAATACTGATTCGAACATTTTCCATTCTGT
CTTGGATTTATGCTCCTGTTAATTGTGCCTGATCGCAATGATTCCGGGTGGATGGTACTAAGTTGCTT
TATTACAGGTTTTATTATACTCAATGCTCTCATTTGTAACTTGCCTAAAGTGCTTCTGGATTTAAGTATA
ATTAAATTGAGAAATGTTCAGAAATGACTACTGCTGCAGTTCTTGTGTTTTAACTATATGGGGAAATAT
GATCCCTTTATGCATGCACCCTAAACCCATAAA

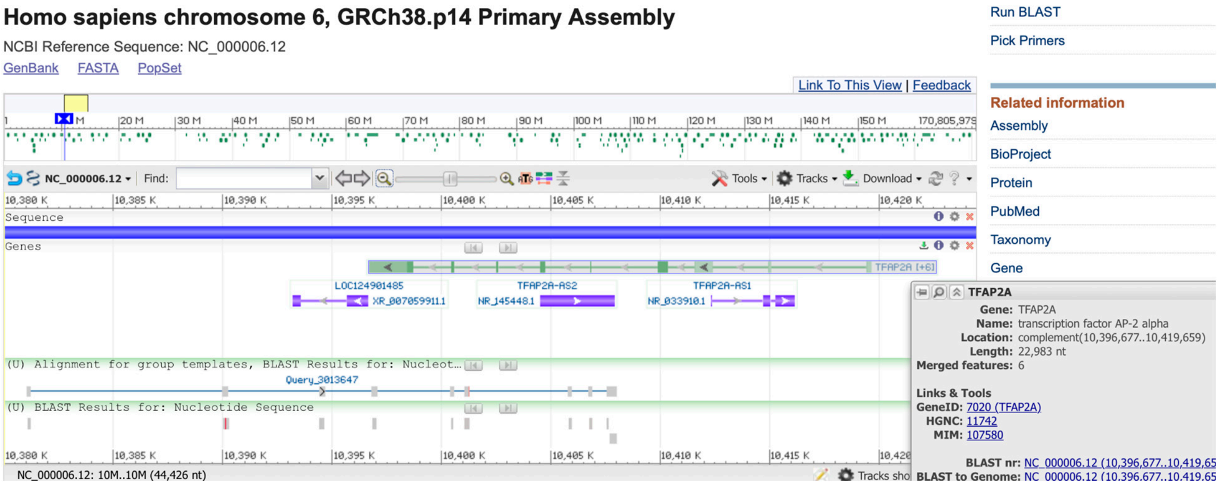

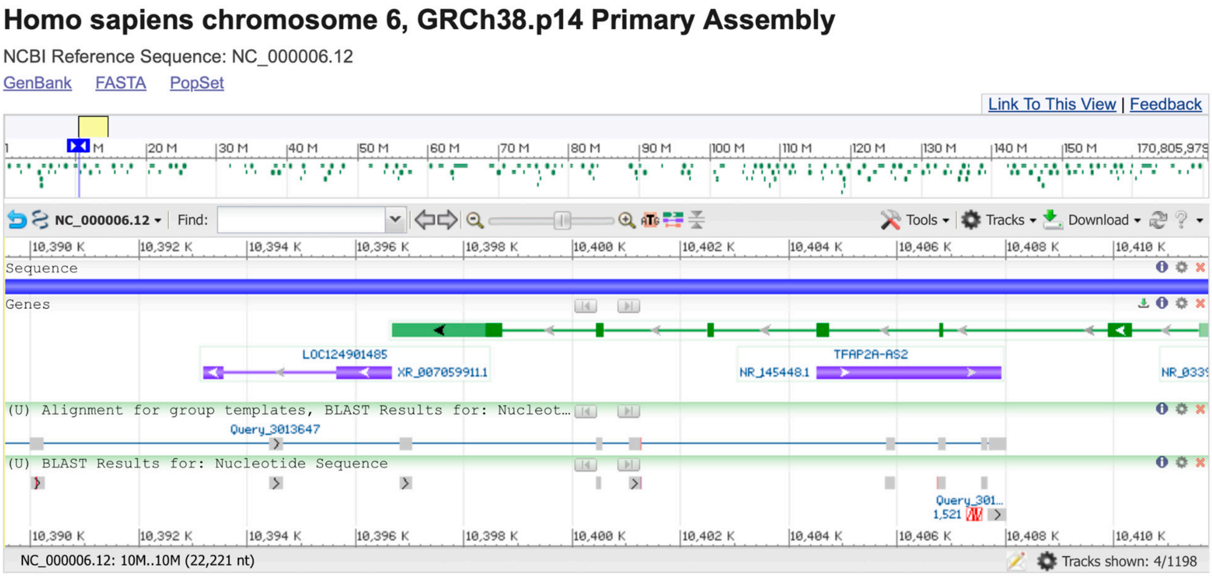

chr6 CLUSTER 27 positions on BED file: 5044 - 5054

>NC_000006.12:50818730-50818980
tttgctgaGGCTTACTTGTCCAGCCTATTGTTTGAGACAACAGATATAAGTTGCGATGGGAGAGGAGCGT
CGGATTTGGTGTGTATCCCCCATTTCCAATTATAGATGGATCATTACAGACAGCGGAGTCCTGAGAA
GCCAGACATCTGCTCCTCACATGAATGCACTCACCTCCTAGAGACCAGGCTGCCATCATGCTCTGGA
AGCTTGTGGAGAATGTCAAGTACGAAGATATCTATGAGGTGAGTCG
>NC_000006.12:50821705-50821830
GCTCGCTGCTGCTTCACGCTCCATTTGTCCTCTATTCTCCCTTTAAAGTACTCGTGTAATATTAATAGT
CATGAATATCAATTATTATGAGGCGGTGTAGGCAAGCGGAGGGAGGAAGGGGCGGC
>NC_000006.12:50826887-50827103
TACGAGTCTGAGTTGTTTTTCTGCGCTTCCGCATGCAGTCCTAATTAAATCTTTACGATCCTCCCTGT
GACGATTAAGCGCCAGCATGCTGCGTGAATATCCTGTACAAAAACCCAGCAGGCGGCCGTAATTAG
ATAGAAAATCAGACAAGAGCCTTCTCATTAACTACCACAACTACCCCACGTCGCTGATGAGGCGTTTA
TACATATTTACGTA

>NC_000006.12:50828569-50828671
ATTCTTTAATATCCTGTCAATTTGAATAGTGATGTTTTTATATTCACAGTCAGTTGAAGATGCCAATAAC
AGCGGCATGAATCTATTGGACCAGTCTGTCAT
>NC_000006.12:50829238-50829400
CCTACATATGCATTCATGATTTAGTGTGACTGTGGAATCACCCCATACTGTTAAAGGGCATGTGATAT
TAACAAATGTTTGCAGTCTCTTGTACAGCTGTAAGCaaaataattaactaattaaacTAATTAAATGTAATTACA
ATAACTCATTTTGGGTGTA
>NC_000006.12:50831170-50831396
CTCTCTTGTTATGTTTGCTATCTGGGCTTAGCTGACATTTCTGAAGTAATTTAGTTAAGAGGAAATGAG
TTGTTCCCAGTAGAGAGAGCAAACGAGTGGAGTGAAGGTAGCCAGCGTGATAAATGGCTCTTTCATT
TGAAGATCTCCAGAGCTCGTTtacggctaattttctgtattggcCCCCTTTGAGCTATTAATGCAATCTGCAGGGA
CCGTGGCCCCTCTC
>NC_000006.12:50831465-50831638
GGAGTAATGAAAGGTGCTTTTTAAACTCTCCTAATCTAAACCGGCAGAGGGTGGTTAATACGATTTGA
AGGGGGCTGGTCAATGAGCAATCAATAAAGTAATAATGAGAAGGATTCGGTACATTAACAGTCTAAA
AATCCAACGAgacattaaaaaatcattcttaacTCTCA
>NC_000006.12:50837913-50838061
TCTAAACTTTGTTTCAGGAACACTCTAAGGTTAATCAGAATGTTAATTCTTGCAATTTCAGAGCCAAAT
CGAAAAATGGGGGGAGATCTTTGCGAGAAAGGCTAGAAAAAATCGGTTTGAATTTACCCGCGGGCA
GGCGCAAAGCAGC
>NC_000006.12:50841016-50841182
TTGTCCCGTTGACAGcgtaatttaaagaaatatatggaAGGCTGAAAAATCATTTGCTCAAATTTGTCCAGATGT
TTTTGAGACGTGATTGGAATTTGATTGCCCCTTTCCGCAGGGTCAAAAAATATTAATGTCCCAGAACT
TTAATTGCTTACAGACCCTGGTT
>NC_000006.12:50843242-50843397
GCCGCGCTCACGGCCCTGCAGAACTATCTCACCGAGGCGCTCAAAGGCATGGACAAGATGTTCTTG
AACAACACCACCACTAACAGGCACACGTCTGGGGAAGGCCCAGGTAGTAAAACTGGCGACAAGGAG
GAGAAACacaggaaatgaaaaat
>NC_000006.12:50844320-50844470
tgaaTACGTTGATTAGTATGTAATTCCTTCGGAGGGGTATGTACCATttcattctcttctgttttccaaaGCTTTGCCC
GCATGGTTTATGAGCTTCTTCAAAGAGGACTTGGGCTTCCTACACAATCTATAaggtacagagaaaaaaaa

Homo sapiens chromosome 6, GRCh38.p14 Primary Assembly

NCBI Reference Sequence: NC_000006.12

GenBank FASTA PopSet

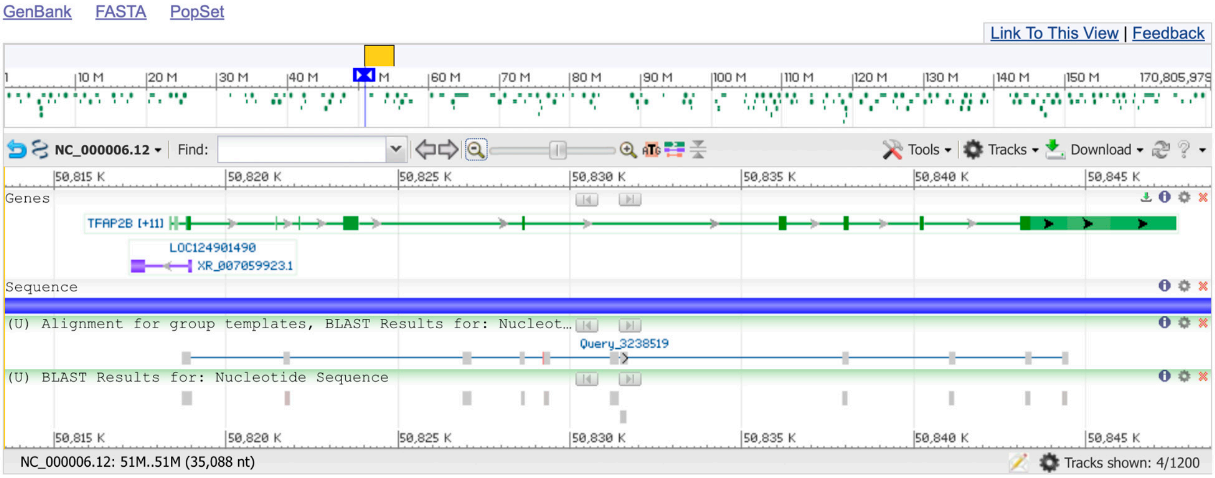

Homo sapiens chromosome 6, GRCh38.p14 Primary Assembly

NCBI Reference Sequence: NC_000006.12

GenBank FASTA PopSet

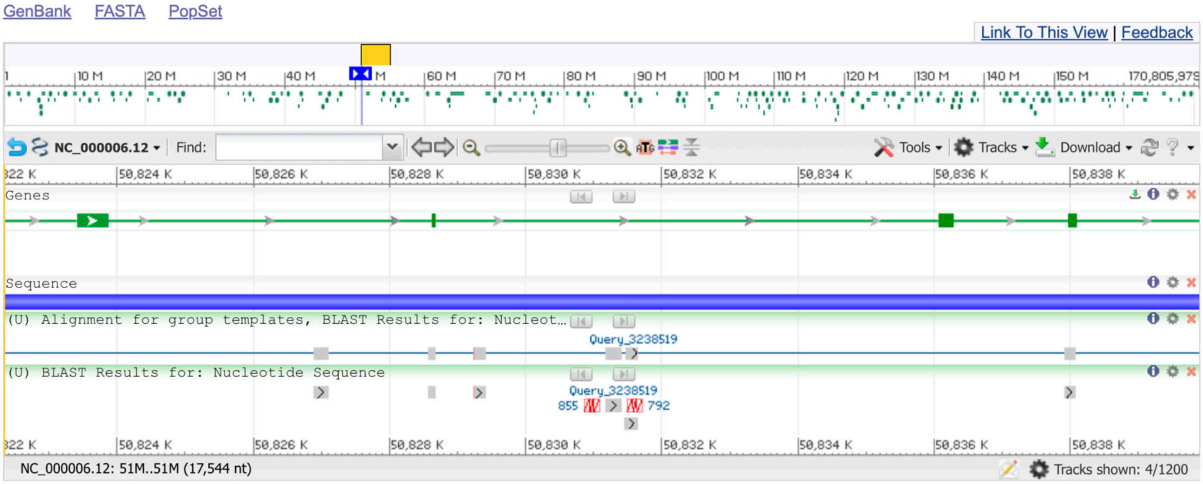

chr7 CLUSTER 28 positions on BED file: 5412 - 5431

>NC_000007.14:1226313-1226531
AGATTTACTCTCTACAAAGAGAGAGCAGCCCAGACAATTCATTAGGCAGGCGGCTTGTAAATTAGAG
CTAAGTTAACCTGATTTCCCTTAATTAAAACATCTTTTCTCGTTTACGATGTGGATATAAGTAGATCTC
CAGGGTTTTGAATTTTCTGCAACAGCAGATGGTCAGCTAGAAGCAGATAATAGTTAACGCTTTCTCTC
TACCAGATCTGAAC
>NC_000007.14:1228374-1228518
CTGAAGGTGTTGCAGAAATGAAATGGGGGTTATCTGAAGATTTGGttacattttctctctcattgTGTTAAATTAC
TAAATTGAAGTTTTATAATAAGGAATAAGTCAAATTGCAAACCCCCATCAAATGGAGCTGTGATTAAC
>NC_000007.14:1228699-1228873
AGTCGACAAGCACCTCCGATTTCCTGATTACAATTAGACTCCTGATTTGGGCTCCTTCAAGCATTGAT
AATTTTCGGCATATTAAGCACGTTTTAGCAAAGGTGATAAGTCAGTTTTAattgaaatgaaattattattctgATGG
AAgtttggttattattattaaaaaggcac
>NC_000007.14:1228915-1229022
TTTTTGAAGGGCATTAGGGTGTCAGGATTGAACGAGGTAATTTGAAGcgaattactttttctttctatcagaAATGA
AGTGAATTAAAACTCAAAATCAATCGCCT
>NC_000007.14:1229248-1229491
CATCCAGTTCAGCAGCGGATCAAATCACCTCCAACAATTAATTTAGATTTAATTCTGTAATTATCAGCA
AATCGAGTAATGCGCAAGTTAATTTAGATAGTTAAAAATTAACTTGCGTGAAGTTAATTGAGTAATTAA
AACCCTCAACTGCCGCCTATTAAtttgctaattaaaaataaatttgattttgtgTAATTTAAAGTAATATTGACATCAGTG
TTGGATGGGcgattttattagttt
>NC_000007.14:1230631-1230766
GAGGTCAAGTGACGGACGGATTGTATGCTTcttcataataatattaattaaacaATTTGCATGCTAATAAGGTAAC
AATTATCAGGGCCTCTGTTGAAAGATTCGGGTTATTACAACACGCCAATCTGAAGGCCA
>NC_000007.14:1230774-1230880
AGAGTGAGAGGCGAGCGAAATTTCAACTCAAATTAACATTCTGTCAGCTCCAGAAAATGGGATAAATA
AAGTCGAATTAATTCATTATAATAAAGAGAGATGGGCG
>NC_000007.14:1232346-1232476
AGCGGATGATGAATCTCCTATTATTGGATCAGTCTATTTGCCGCTCAATGTCTCTCTGTAATTGGAGC

AACATCACTTTAAAGGTTCAGAGAGTACAGCATTTCTGGTGAAAAATCCCCACAACACGGCG
>NC_000007.14:1237649-1237872
AGGGCCGGGCCGCTGGGCTCCTGCAGAGTTTTGATATGAAAGTAATTATTTTCCGGTGGCTGCTGC
GGGGACTGGGTTTCTTGCCCAAAGGGTTATTATACATTACCGATTTCTAGTGATATGGAATCACATAA
ACTTCCTACAATAATAGAATTAGCATGAAAGGCTGGGGTGTCAAATTGAAATTGGAGAATAATAGCGC
CGGCAGCTGGGGGAGTGCGTG
>NC_000007.14:1237924-1238119
ACTCTTTGTCAGCGCTCTTGTAGCCGGCTATATTAAGATAGATGCCCAATGATATCCCTCATTGTTCT
GTCGCTTATATTGATGTTGCTGCGTTATCAGCCTATTGATCATGTGTCGCCTGTAATAGGACAGGCG
CCGCCAACGCGACTCTTTACAAAAGCCGTGCACATTTGTTCTAATAGGGTCCGGGTCCCC
>NC_000007.14:1238400-1238505
gtggaaTTATGTTTACAGCCTCGTTAAATATCCCTGATCCCTCCTGGTCATCAGGCctttatttgcaaataaattgaa
aataatctgAAGGACAGCTTTCCTCCT
>NC_000007.14:1238510-1238642
GGGCGCAAATGAATTTCGGAGCCCGAGTCCctttaataatatttacataattttcgCTCAGTGACTCTGATATTTGC
TCTCTAGGAGACCGAGCttataggaaaaataattagaTCAAAAGAAAAGTGAGAG
>NC_000007.14:1238965-1239113
ccattAAACTGTATTTAAAGTGtcatttaaattatgaaattgtGGCAGAAAATTGTGCGTAAAATGCCGGTTATTTTA
TCTAAGGTGAACAATTTGTCTGGCAGCGATAAATCGCCTCCTTCCTTCAATTTGCAGCTGTTCATTTC
GG
>NC_000007.14:1239176-1239587
GTATCGCTGGCTGCACACCCGTGTCCTTAAGGTGAAATTACTGATTTACTTAAAcagtttagcttttttttctgaaag
cccAAAAGCAGCAGGCTGTGTGATTCTAGACAAACTATCAATCGATCTGGTCGAGCCAGCCTCCAGGA
GATGTATCCTTCATGAATCATACTTTATGAATTCAATTTCTACCAGGAGAAATTTTCAATTTGAACGCC
GGATCATTATGGGAGAGTGTAAATTGTTTTTGCTCTATTATGCATCCGAcgccatcatttttctttctaattacgGAG
GTGTCAGGTCGAGCTGTCATGCAGGCGCTGATTTTCAATTTAACTGATCATCATACATTCATTTTCCC
ATTTGATAAATCTGCTATCTAGACGGCTCGCCATGCCTAGAATATTAAGA
>NC_000007.14:1239827-1239989
acacCCTCCAAAACGCTTGTTTTCTATTACACAACTTCCAAATTAGGATATCAAGCTTAATTAATGCTAA
TTGAGTTCTTCAGTacgtgttatttttatttaatagaaacaAATTTGCACAATTAATTATCGACGTAATTTCAAGAGCT
TCATTTGCAAGGC
>NC_000007.14:1240694-1240913
AGGGTgttgtttctttgctgatttatttttaacCCGAGGTTGTACAAGCCCCTGACAGTTTGGATAAATTAGCCAGGCC
TCGCAGTCTCCTAATGAGAGGGTATTATTTCGGCACCTCGGAAGGAGCGTGAAAAATGAGAGAGACT
CCATCCGGAGGTGTCGGATCGATTCAGGATCAGGGTGTAAATTATATACAACTAAATATCCAGCCGC
CCATAC
>NC_000007.14:1241960-1242071
gcagaaatgaaaaatcagTGCAGTTACATCGTAAAATTTGGATTAAGAAGCTTGAATTTAATACACACGCACC
ACATTTATTAAAGCATTAGAATAATTGAAATTTGCTGC
>NC_000007.14:1242075-1242212
GCCGTCCAATCTGTTTAAATAATTCTATATGGGTGTCTTGTTGTGATAAAAGATTATCTAGAATTCTAT
TAAAGGCGCAGGAGCCTGCTTTGTAAATCCTATTTGGGGACTTTCAATAACTATCGATAATCTCATCT
>NC_000007.14:1243220-1243352
GCGAGCCGCCTCTTTCCTGCTAATTATCACTTTATGAAAAGTGTTTCATTAAGAAACTCTGCTTTTGAT
TAATTATCGACAGAATGCTgaccagaaagaaatgaaattaatctCTGACCCCGTCTGGGTAAC
>NC_000007.14:1243478-1243620
ATAGCagtctttgttcattttaaatttattaaacataatgcacttcattttcaaacatttactgtttctttttaatttcactcAGTGTAAGTACAGAAT
AAaaccttttttaaacatttaagatATTGAAAACCCATATACG

Homo sapiens chromosome 7, GRCh38.p14 Primary Assembly

NCBI Reference Sequence: NC_000007.14

GenBank FASTA PopSet

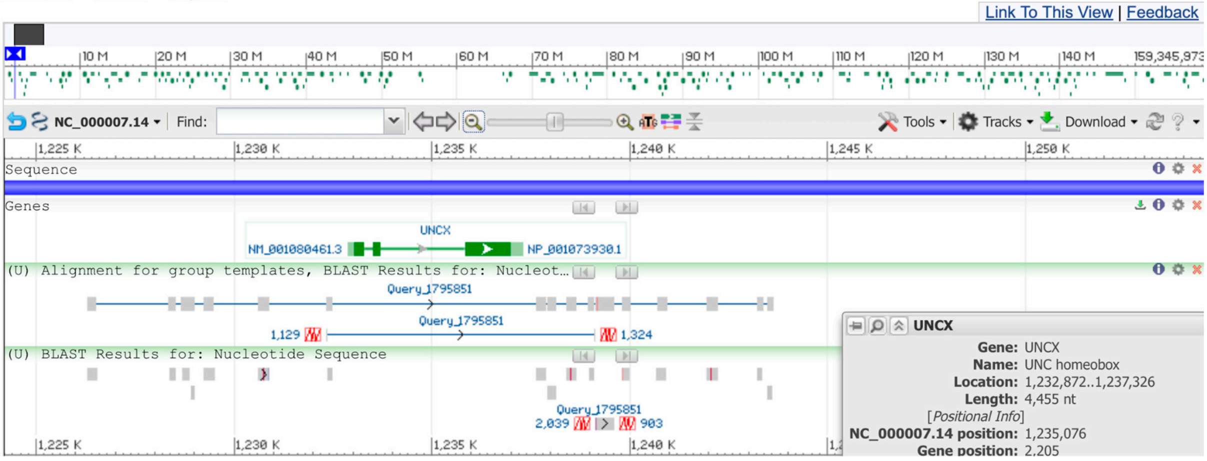

Homo sapiens chromosome 7, GRCh38.p14 Primary Assembly

NCBI Reference Sequence: NC_000007.14

GenBank FASTA PopSet

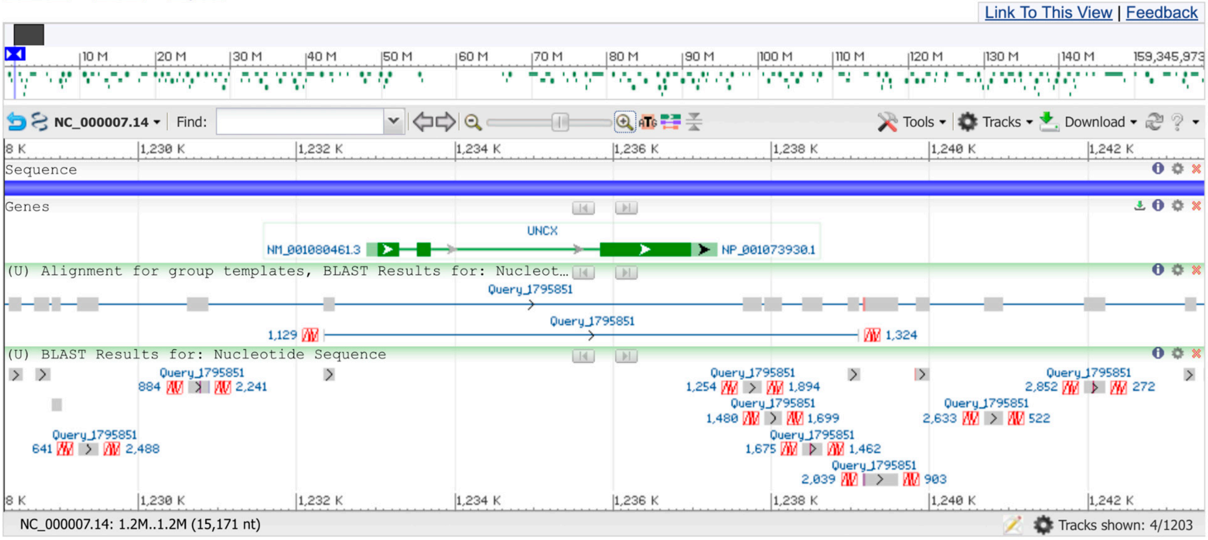

GENE UNCX is inside cluster and NOT intersect with UCEs

chr7 CLUSTER 29 positions on BED file: 5579 - 5591

>NC_000007.14:27127032-27127141

CTCCACAGACTTTTCCCAGAGAATGCCTTTCAGAATTGCTGTTGAATTACAAAGAAGTATTTATTGTTT GAGGTGATTAATGATTCCTGGGAAAATTGCTATTTTCAGC

>NC_000007.14:27129647-27129785

gtggggtggggaagagcaATTGGACatcatcattatataataacctgACACCAAGTTCACGCAAGATACATAAAACGGC AAAGAAAATGTTTCACAGGCTATTGACAACGGGAAACACCCGAGAGCCATAAAGAATGG

>NC_000007.14:27130657-27130791

GCTGCGCGTACTCCTCGAAGGGAGGGAACTTGGGCTCGATGTAGTTGGAGTTTATCAAAAACGAGC
TCATGGTCATTAATTTGTGAAGTgcaaaaatactaatttttctcGCGTTGTCGTTTTTTCTGGGCTTG
>NC_000007.14:27133747-27133938
TTCCCTCTTGAATGCAACCCTGTCCCAAGTTTCAAAGTGACCGAACAGTGACACCGTGtgcattttgtttcttatt
aatcTTACACATTGACAGTCTTTGTTAAATCACAAGGCGCGCCCTTCACTAGCCGACATTTTCATATTTG
TTAGACGCACTGACCTGAAGTTCACCTCGGCCTTGGACTTTGCGC
>NC_000007.14:27136545-27136649
GAATTGACTGTGAACTTGTACTAAAGCGTGCTCTGCTGGCGATTCCTAGGGTGTGCAGATTTATCTTC
TCTGCATTTACTTAACCCGGCAGTGAACTGCGCGGG
>NC_000007.14:27140149-27140472
AAATTGACAGTTTGAATGGCCAGGCGGCACACGTAGCCTGCAAAAGAGTCAAATGGAGTCCAGCGT
TAGTGAGATTATATGTTATGTGGTATATAATGTTGGATGTCAACTccccaaaaccataaaacttaCTTTAATGG
CCCCACGTGACGTTTTATAGCCAGTGAGCCGATCTGTCTGTGCTATGGATGATTTTACGATCTAATTC
ATAGACAAAACCCTATTCATTTGGCACCCAAATGTCATATAGCCGGAACTGGGGCTTATAAAGTTTact
gttttataacttttaaaaggaaagacGGCATCAGTGTAAGCAGTCG
>NC_000007.14:27141154-27141287
TAAAACAGCCAGACTTGGACAATATTTGTAACTTTGTTCACAAAAACATACATCACTGAAGCTGCGCT
TATAAGAGCCACTTCCAGAGTTCGTGCAAAGGGTCCTATAAAGGCACGCAGGGACACACCGCTTG
>NC_000007.14:27141862-27141975
GCCATGCTCATGCTTTTCAGCTTATTATCTTTTTTCCACTTCATTCTCCGGTTTTGGAACCAGATTTTA
ATTTGTCTCTCGGAGAGGCAAAGAGCATGTGCTATTTCAATCCT
>NC_000007.14:27143425-27143853
GAGCCCGAGCGGCCGACGCTGAGATCCATGCCATTGTAGCCGTAGCCGTACCTGCCGGAGTGCAT
GCTCGCCGAGTCCCTGAATTGCTCGCTCACGGAACTATGATCTCCATAATTATGCAACTGGTAGTCC
GGGCCATTTGGATAGCGACCGCAAAATGAGTTTACAAAATAAGAGCTCATTTGTTTTTTGATATGTGT
GCTTGATTTGTGGCTCGCGGTCGTTTGTGCGTCTATAGCACCCTTGCACAATTTATGATGAATTATGG
AAATGACTGGGACATGTACTTGGTTCCCTCCTACGTAGGCACCCAAATATGGGGTACGACTTCGAAT
CACGTGCTTTTGTTGTCCAGTCGTAAATCCTGCCTGATGACCTCTAGAGGTAAACTCGTGCACTAATA
GGGGAGTTGGGTGGAGGCGAGGGGG
>NC_000007.14:27143964-27144072
CAAGGGCCGGGGTCGAATTGAGGTTACAGCCCATTATGGCAAAATTATTGCATTTCCCTCGCAGTTC
CATTAGGATGTACCAATTGTTAGGCCGTCAGCTGCCGATCG
>NC_000007.14:27147708-27147903
AGGCTCCCGGGGAAAGTGGGATTCACAAAATAGGAACTCATTTGCGCGCCCCTCTGCAGGACTGTG
ATTTGTTGTGTATTAGTACATCTGGCTATAACTATTAGTAGTCATCGAACTGGTTTGTTTCTGGATGGC
CGAACGCCAAAAGCGACAGCAGCAAATCGCACCAGCTGACGCGGCGGCGGCCAATGGGAG
>NC_000007.14:27152564-27152761
GAGCGGAGCAAAAGAATGCGGCTCTATTCTCGCaagggaaattataaaaaagTTCATGTTCACGGTTCTCAT
CCACATGACCGACAGCGGCCAATGGAAGGGCCGAACAACTCATAAAGTTGTATTGCAAAGTTGTAAA
TTTTCATAAACAACAACGGATTTATGACCCTTTCCCCATCACTGAGAGGAGGCAGCTC
>NC_000007.14:27156426-27156696
TAGCCGCTTCTCTGTGAGTTGGGAGCAAAGGAGCAAGAAGTCGGCTCGGCATTTTGGAACAGAGAA
GCCCCCGCCGTATATTTGCTAAAAAGCGCGTTCACATAATACGAAGAACTCATAATTTTGACCTGTGA
TTTGTTGTCCGGCAGCTTTCAGTGTCGGTTTTACGAGGTAGAGTGATATATGATAACATTACACCCCC
AGATTTACACCAAACCCCATTTTCTTTTGGACGGAGCTCGCCGCAGCACGTGACCGCCCACATGACC
G

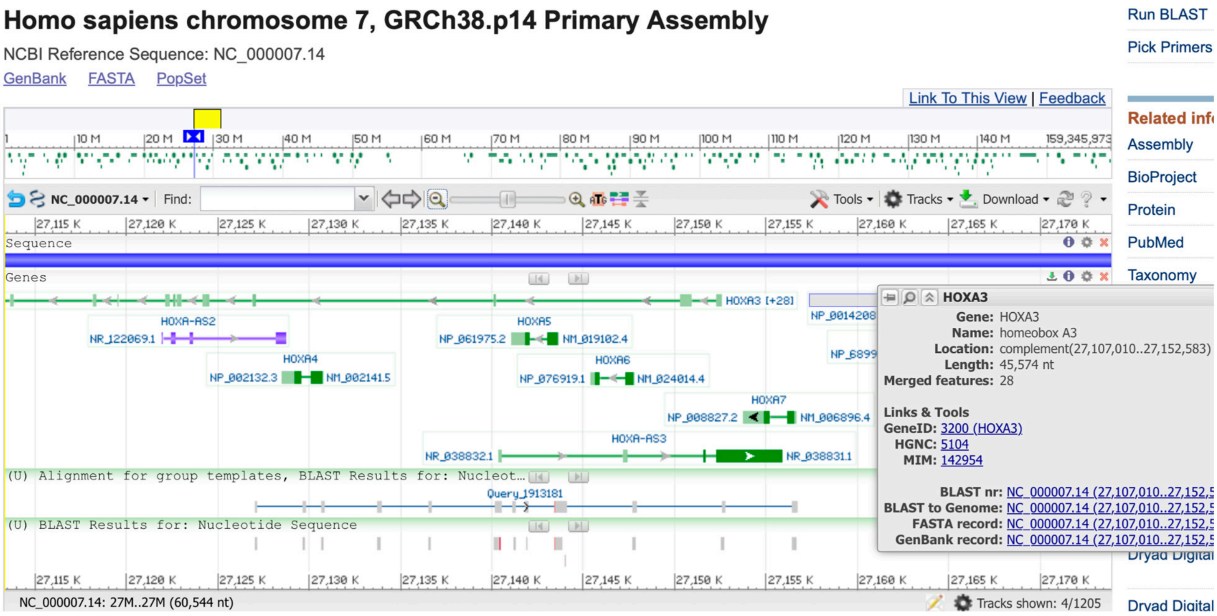

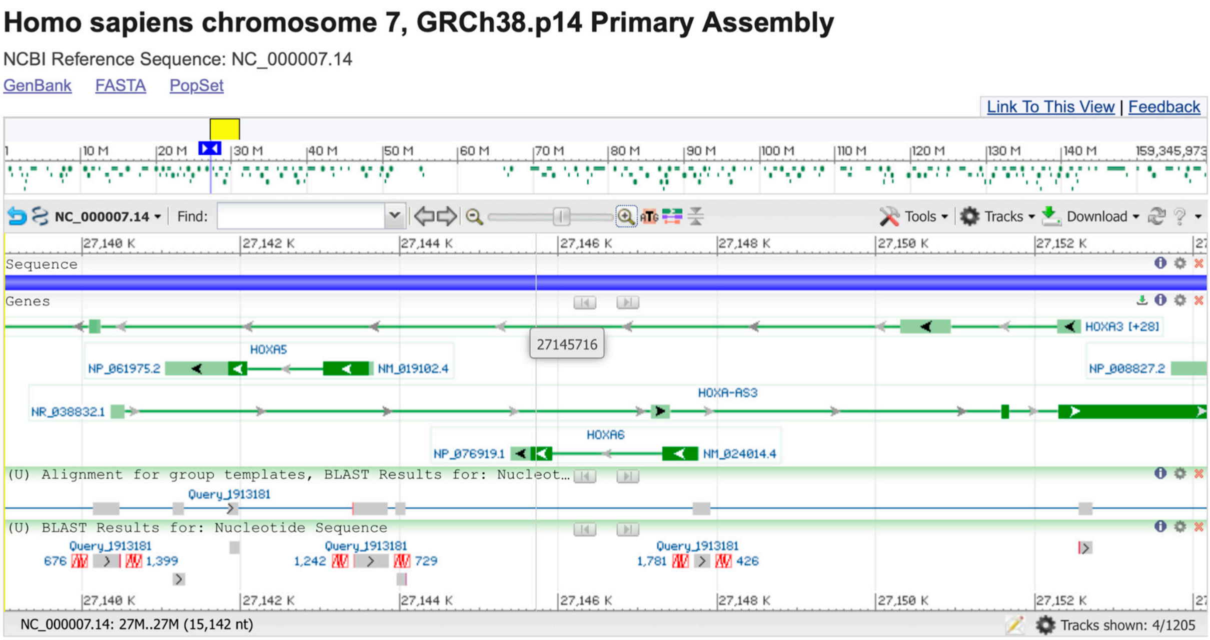

Genes homeobox A5 and A6 are inside the intron of gene homeobox A3. UCE cluster intersects with all exons of these 3 genes A3, A5, A6

chr7 CLUSTER 30 positions on BED file: 5843 - 5858
>NC_000007.14:114412670-114412980
GGAGGCACCTATGGCTTGATAGCTGTGTGAAACTGTTGCCTACAGAGATGGGCTCTTGACACTGTCT
GCCAGCCTGAGGCTGTCCGCTTTGTGTCTGCTTTAATTGAAAATTAATAGTTAGGGGGTTGCTATGG
AAATGATACCATTGCTTGCAGACAGCAAACCCAGCTTTTTGCAAAGAGACACTAGTGGTATTGTTTGT
CCTTGTTGACCTCAGAAGTTCAAAAGcctggaggaggaaaggagatgcaaatattttacttaaaaaaatgaatttacatatttataa

caatatttttGAATGTCAGAG
>NC_000007.14:114413619-114413737
AATAGTGCAGCCTCAGGGGTTGAAGGTGAAATGTGAAATTTGCTGTAATTACTTACCCAAAATGTCTG
TATTAAAGGCTATTAGAAGCAGTTATTTTAAGTACTTCTTTATACATCTT
>NC_000007.14:114414217-114414515
ataaaatcttaaaactccaTGTGGTGTTGTGAGCTGGTAGCAGATGGACTTTCTTTTGCATCTGCTGTGTACA
GACTTGGCTTTGTACTCTTTTCCTGGGGGAGAGAAAGtctttcttctctgctagttgattCCTGTTTTTGTGAAACAG
CTAAGAAAACATCTGTGGCAGGcagaaaaattgacaaaggattGACAAACACCATTAgctgaaaattttccatttcattgcaa
CTATGCATTGTTTACTTTACTGTAAATATCTTAATACATCATCCTCTGAATATGCTGGCAGAGA
>NC_000007.14:114414524-114414834
ACTGTGATTTCAATTAAGGTTAGTAATTGATGGTATCTAGTGTTCAAAGGCTGAAGCTTGATTAACATC
TGCTTGGACAAATTGTCATTGTGaagtggttttgatgtgcattgaAGATTATTTCTTTCTGGCTTATCTGATGTTGT
GGCTGTGAAATGCTTGTCTTgggtttgcttatttttgtaaacTACTTCCTTTGGCTGTAAATTGCAGAGCACTGGA
GCTTTACCAAAAGTGCAGTGTATAATGGTAAGCTTGTCCTAATAAGGGAAGCAAGAAGTGTATTTATC
ACAGACATGAAAGCTAACCG
>NC_000007.14:114414950-114415254
tgaagcACTTACTTTAGAAAGATTATGGTAAGCATGCTGGCTCAGTCTTGAACCTTTGTCACCCCTCACG
TTGCACACCAAAGACATACCCTAGTGATTAAATGCTGATTTTGTGTACGATTGTCCACGGACGCCAAA
ACAATCACAGAGCTGCTTGATTTGTTTTAATTACCAGCACAAAATGCCATCAGTCTGGGACGTGATCG
GGCAGAGGTGTACTCACAGTAGTGTAAATACTGCTGTAAATAGTTGTCTGATGGTGGCTTTGACAGT
GAGCTAGCTTCTGagttttcccttcttttta
>NC_000007.14:114415630-114415895
aaaaaaatctgcaagatCTTGAGAAGAATAAAGACAGGGTTCCTGCTTTCATGGTTTGATAATAACCATGTCA
TCTTGCTTTTAGTAAATGCCCCAGTATGTGTCAGGGCGCAAGTTTTTGAAAAGGGAGTTTGACCACAC
GTTTGGGTGCCTCTCTGTGCCGTGCTGTGTAGAGCAGATGTTTATTTTGTGCTAAAGGGGAAACTCT
TTTTGCTAAGCTTGTACAAACATGAAAGGAGTCAGGGCTCAGATgatctttgtagtt
>NC_000007.14:114416171-114416305
TAGGGCTACAGTAATTGCCTGATGACAACTGAAAATGATAGAATAATTGTCAAGAGAGACATTTTTAA
TGGGCAAGGTGATTTAAGTATGCATGAACAAGCTATGAGGGAAGATGAGCTTGTTTATGATGCAAG
>NC_000007.14:114416997-114417791
AAAAGGTGGATAGATAATTTGTTTACTTTATTATTCATGAAGGCAAGTAAGGCATGGTATTCTGCTATT
GTGGGCATATTATTAACATTTCATAGGGATTTGTGCTGGAATGTGGAATGCTGCTTCTTCATAATTTAA
TACTCCTATGCATAATGAGGTTTGTGATTAGTTGATAGAGAGAATTGGCCCAACTCCATTCTGAAAGC
AGATAATTTACATTGTTCTCTAGAGTCTAGAATCTAATAGGTTCTTTAGTGCAATAAAATTAAATGCtaca
tgttttaaatttcagCATACAAATCCCCCTggcaattttctgtttttaatttttgccttttgtttcctctaaaacaatgaattttaaaaattgtttctag
aACATATATTTACCTAGCTCTTTTATTTAGTATGTACAAGTCAATTAGCACTGTTCATTAGCAGAATTGg
gtatttgttttaaagtttaacCAATCACTTTGAAATGCTAATTATGATGTAATTTAATTGCAAGTCCTGTAATGATGA
TGCTGATATTATCGACATAAATGATGCAGTTAAGCAGTGGAATCTCATTTGCATATGCTTAAAGCAAG
TTGAATTGGGCTGTTTCAATATCACTTTGCTTTAATTTTCCACCTCTGTTCACACCAGCCCTTTGGGTT
TTTAAAGCTGTGGTTTCCTATTGATGATCAGtgctttcatctttattttaatgaCAAAGGGGCAACAACTGACACTG
GAActgtaaaaatgaaaagcaaataattACTTCTATTAAATATACGAACTATGAA
>NC_000007.14:114418093-114418483
TAAACTCCAGTGGTTATCTGTTTTTGTAGAGGTTTGGACTTGGGTAATTTTCTTCtggaaagattatttttcttctga
ccAATTTCTCCTGTTATGTCCTAATTGGTTACATAAATCTTGTCTGGTATGAATGAGAAATGTTTCTGTG
TTGTCAAGTGTAATCTTCACCCTTATTTACCAATTCATTAAGATCTATTGATGCAGGACTGGTGAGAG
GGAATGACAACATAAATCAGCCCTCCAACATAATGACCCTAATCAGCTAGAAATAACTGGAAACGTCA
TGATGAGCTATGTCTCTGTATTACTTCGGCAAGATTCTGTGGCTAGCGAGGTTTGTGTCTGCTTCGAT
CATTAACTTTACATCCACTCACCTTCTTCTAAAGAAGT
>NC_000007.14:114422907-114423363

GGCCTAATTGAGCTATGTCTTTATATCCTgctatttgtacaaataaaagaCTACATCTTGatgcaaaataaacacaaaag
caCTGTTGTGACTATAACAGGGAGTCATATACTAATTTATATAATAAGTGGTTGTGCACGCTTTATTCA
AGATTATGGCTGCAAGAAATGGCATGAATTGCTGGGTGTGTTTAATGCTGGATTATAGCAATTTGAAA
CATGTCAAACACATGGCTAATGCCTCATGCTTCACTCATTCTTGCGTAAACCTGCATAGCCACCATCC
ATTATACCCTTGGTAACAACACAATTCTGCAAGTAAATATTGTTACACTAAGTAACCTTTCAATTCAAG
TTGTTTAGGAAGAGAAAATGCAATCTTGTGCAGTCAGTTCCAGTGTCAGTTTAATGTTTCAAAAGGGA
AGTGGTGTAGAGAATAAGTATCTGTACAGTGACt
>NC_000007.14:114425517-114425951
TTGGCAGGACATCTCATTGCTATCCTGAAAGTACAGTATATTTATGCagtgaaaatgataaaacatttatTACTGT
AGAGAAGGTAATATGCATAATTTATGCTGTTTTTAGCACAATCATTAGTGATATTCTTGATAGATTTTAT
TTCCAGCTTTCATATCTTATACATGCctggaaaattaattttatatctttcatttatttgcctATGTTAAAATTGAGTTTTAAG
TCATCTTCAAGTGAACAGTTTGATTGCTGCTCATGCATAAGCTTAATTACTTCCCAGGAAGGAcagtatta
aatgttttaaatgtcagaaaaataaatgaatatgagctgtttgattaaaaaaataggcaATATCTGACGTGTTTGCAGCAGGAGCT
TTTTCTTAAAATGCCTCCAAGGCAAAATTTTATTtagtcacaaaaaa
>NC_000007.14:114425977-114426160
GGTTGGTACCATATGATTTGTAACACCcttacaaaattttaattttgtatgatTAGCTGTGCATCATTAAACAACAGC
CTTGCATAAGATGTGCTGTAAAATTCTGACAGAATCAAGAtagtgaatatttaaaataaggctGTATAGTCATCCA
TGGTTGTCAAAGGTAGATAATAGGAAACA
>NC_000007.14:114426405-114426660
AAGATGCTGTCTCTGTAATTGGCAGAGAGGGACATCTTGATAATGGAAGTGTGAAGGTGTAACGTGT
GTTAATTGATACTTCTTAATCACTTTTAGGTATTAAGTCATGATGCAGGAATCTGCGACAGAGACAATA
AGCAACAGTTCAATGAATCAAAATGGAATGAGCACTCTAAGCAGCCAATTAGATGCTGGCAGCAGAG
ATGGAAGATCAAGTGGTGACACCAGCTCTGAAGTAAGCACAGTAGAACTGCT
>NC_000007.14:114431074-114431670
ATTCACTATAGGTACAAGTGGTAGCATATTCATTGTGCCTGTCTTGCATGAAAGCATTTACTGAAGAA
ATATGACAAGAACATGTTCAGCTAATGAGGTCGAATGGTTGTTACATTAAATTTAGGCGGTGTTTTCT
ATTAGgttttatttatctgtatttgCATTCAATCCTTTCACATTTGTTATCTCTCGCCCAATTCCAGGGATGGGGCG
CCTTTTCTCATTTCACAGCCAATTGTTACTGTGCGCTGACTCTGTAAGTTGCTGGTGAATGTAAAAGT
GACTGCAATTAACCTGACTTTGTTGCTTGAGGAGAATTTGAGATGAGAAATCTGACAGCAAATGACAG
GCATTTTTCTGGGTTTAGCACAGAAGAAAGTCTAAGTAACATTGCAAAGAGAGGCATTAGGGAGTAG
GTACCTGCAAATTAAGTGGCTGACACTGTCTATTGAGAATTTAGATAGTAACGGCTGGAATTTTTATG
TTAAgttcacattattttatattcatagcAAGCTTCCTTTTGAGCATTTCCTTTGTGACtgtgcatgatctcatttattgtaatttgtatttt
ttggttgATCAGAAGATGG
>NC_000007.14:114432101-114432403
CCACACAGTATGCAACATTTATTGTATTTGTCAGGTGTTTAAGAGGCCATTGGGAGTTTTAATTAATGT
CAGTTAGGAAGATACATTGTTCAGTTGGAAGCAGCTTGCCAAGTTCTTCATTTTCCTATAATCCATATA
GTCTACTGTCTTTAGGGAAATATGCAAAATTAAAAGTTACACTGCATGCTGTTGTCTTTACAACATTCA
GCTCATCAAGCAAAACTCTCATGCAAATTTTAATTTGCCCTTGAGCATAATTTAGAAACCATAATCATT
GAGGTTTTACACAGCACAGATAGGAA
>NC_000007.14:114432727-114433117
AACTTTTTACTGTTTTCAGTAATCCCTAAAGAAGTGTACACTTGGCCTGACATATAGAGTTGTCGTCTA
CCACATGATGACACTAAAGATATATGATATGACTGACAGTGTAGTTGATGTGCGTTGTCTTCCCTAGA
CAAAACAGATGGTATTGTTCTATTATCATCCACAGTTATTTTAATGTACTGTGTCCATCTGTGTTAAGA
TATATTACATGGTTTCATGGAATTTACAGTTCACTCCTGCAGTAACTCGATTTCACTGTTTGGTCTCTA
GAGAGTTTGCAAAATGTCAAGCTGTagcttgaaaaagaaaagtgaaatctACTGGAAACTAATACTTATGACCAT
TTCATGCCTGATCTGCTTTGTGGTGTTGTTTAGAAGCTAG

Homo sapiens chromosome 7, GRCh38.p14 Primary Assembly

NCBI Reference Sequence: NC_000007.14

GenBank FASTA PopSet

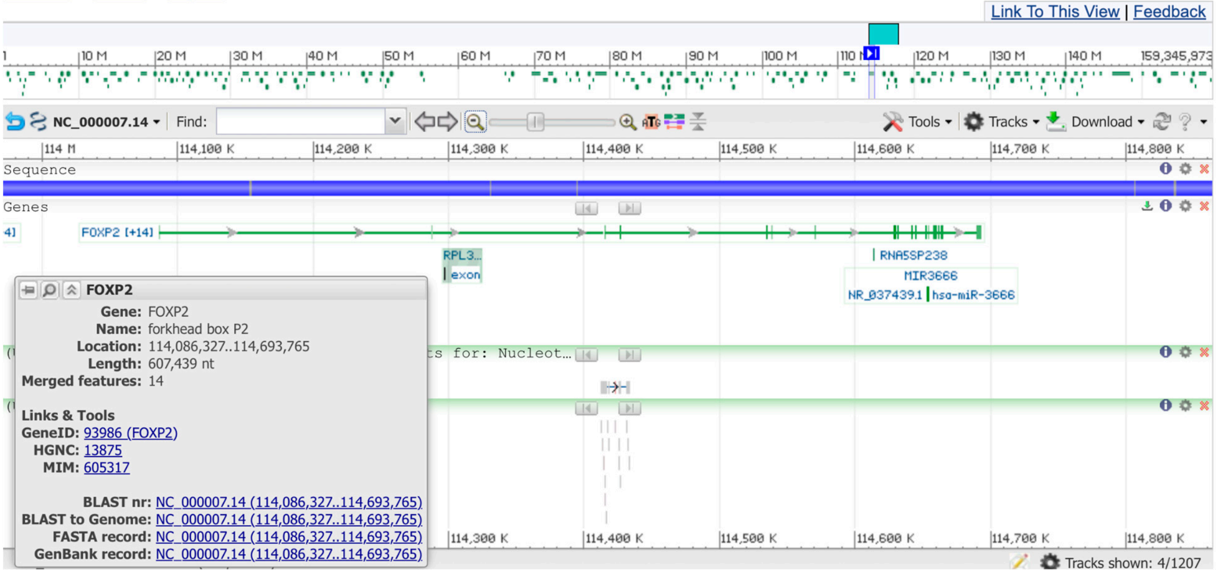

Homo sapiens chromosome 7, GRCh38.p14 Primary Assembly

NCBI Reference Sequence: NC_000007.14

GenBank FASTA PopSet

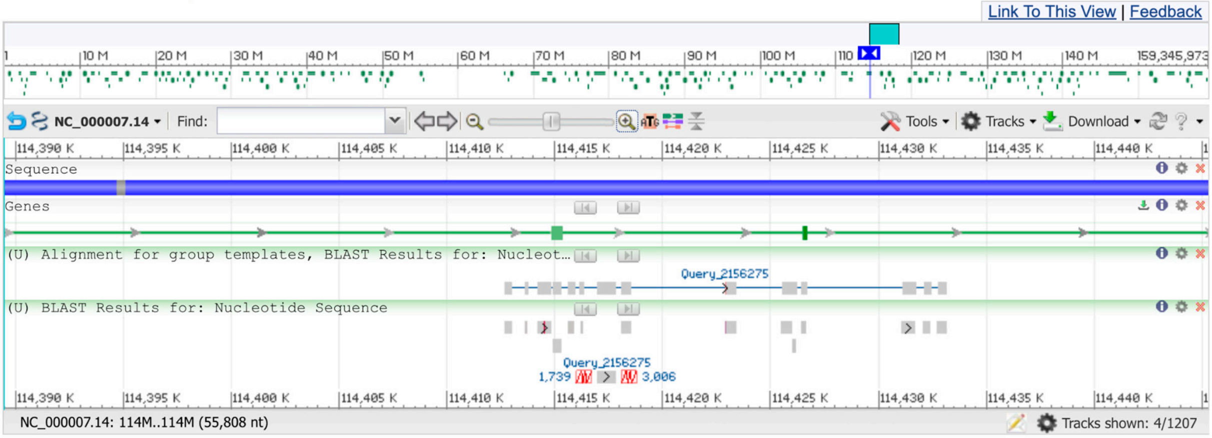

chr7 CLUSTER 31 positions on BED file: 5864 - 5875

>NC_000007.14:114620311-114620515
CAAGTCTTACACATGCAATCTGATTATTCTGACCAAAGTGTTAATTGGATCCTAACTGCCAGATCTGA
TATCTGTGGTCTTGTAGTGGGAAACCTTTCAAAGTGATGAACTGTAATTCACTGAGCTGCTTGAAGCA
CTTGTAATTTACTGCATGTCATGCTATGAAGAGCCTGACCTAATGGACTTTACATTACCTATAATGCT

>NC_000007.14:114622245-114622621
aaaaaaattctgcaattCAACCACCATTCCTCTCTATTCACCACACTTGTCAATCAGTGAGTTGCTGTATCTAC
AGGAATGGAGGTGTTATATTCTGGCAATTATCATGTCATATActtgaaaatgtaaatgaatgtgtgtgtaaaattaaattta
tgggTTGTCAGCGAAGAATAAGAACCAGGAGAGCAATTACTTCTCTGCTTTCATCTTTTGAGCTCTGAT

ACATCAAGCAGAAAACCATGCAGCTAAATGTTGGAATTAGCATTGTGCAGGTCCTGGAATGAATGGG
CTAGTGTCTTTTGCTGCCTTTTCTGACTCTTTGTTTTGGAGAGCAGTGAAATTAAGATGATTATCATCC
ATCAATCTATGAGAG
>NC_000007.14:114622622-114622734
GCCTATGAAAGCATGAAAGGATCTTccttctcatatttattttgctttcttagaGAGTTGTCTTTTATCAGGACAGCTTG
CTTTTTGttgaaattaatattatttggGCTA
>NC_000007.14:114623223-114623478
ATGATTTTAGGAGCCTAATGAACAATGGTGCAGATTTGTTTGAATATGAATGTGCAGTGGGATGGAGT
TTGACCTTCAGCTGTGATTTCAGTTCGTCAGGATTAGTCAAGCTAGGCGATCAAGACTGCTAATGCAT
AatgatttataaaatgaaaaatgtgtagTGACCATGCAAATGTTTCAGCTAATCAAAGAGGGGAACTTTAgctttgagaa
gaaagaaaaacaagggcATGTCAAGGCCTAAACCAAAGA
>NC_000007.14:114629748-114629887
TTTATAATACGTGAAACTTTTGCCTTTATTTATTAAAGTCAAAATGGTTTATTCAGCAACAACTACAAGA
GTTTTACAAGAAACAGCAAGAGCAGTTACATCTTCAGCttttgcagcagcagcagcaacagcagcagca
>NC_000007.14:114630518-114630655
GGCTTCTCAGGCCTAGCTTGCACTTGTCAGCAAACTGCatcaaatataaacataatacaaACAAAACATGTTGA
AGTAGTTAAATTGATCAGCAGGTATCAGAGCCGTTGTCTTCAAGCTGCCCATTATGCAAACGT
>NC_000007.14:114644449-114644648
TAGTTTTTCATCCTTTATTAACAGTCATCTTTGGCTCTATTTCTCCTCTAATTGGAACATCATCTAGCCT
TGGCAGTTGAACTATTCAATAGAACGATTAAATTTTTCTGACTGCTCTGAGCTGAATTGACCTGTTTTG
ACCTGTTTGTCACTGATCGTAACCTGACAGGCGCTAGCAGCCTGCAACGATTATAGCTTT
>NC_000007.14:114645608-114645753
TGTTGTCATGGTGCAGCTAACAGGGTGAGTGAACTGTTTCATGCTTCATCAACAATTAAGTTAATTAG
CTCATTTGAAATTGCACTGCTTTGTTGCCAGGATGTTGGCAGAAACATCAAAAAGATGTGTTTACAAA
TGGAAGACT
>NC_000007.14:114648220-114648432
AGGCATTCTTTGAACTTGTCAGAATGTTGTTTCTTTTGGTGACAGTGCCCCCCCATCTCTGAAAACTA
AGGCATCTCACATAATCAACTAAATGAATAGTGACAGctaacattttgtatttaaaCTCCTGTCAGTGTTTAAACA
TAAAAAGCTTGATGCAAAATAAATCAAACACCAGTGATATGCAAGTGAAACATTTTGTTAAAACAGTC
>NC_000007.14:114648736-114649139
tgcagtttTGAAAGAACGTCAGTCGACCACATCATTTATtccattttgaaaacaaaaatcaatatacCTATAAAGTGACC
CATAAAttagaattggctcacacaatgtTTGTTCAGTGCTGCCTTTCTGAAGGCACCCTTTGCATTTTTTCTTCAC
CTCTCTGTTTGAACTGCAGTCTCGGTAAGAGGCTCATTAGGGGACATTAGGGACTGGAGCTAACCAG
AATTCCAGAGCTTGCTTTGCTCTTATTAATGCATAGTAGAGTAGaatgcatttaaatatttcataggcATTCAGTAA
TTTGTGTGTAATCGCCTTGTTAGCAGACCAGTAGAAGTAGAACAGGCCTGTGAAATGGTGTTAGTGA
TTGCATGGGTAATTTACAGACCTTTGCCATCAGAAA
>NC_000007.14:114649297-114649539
TGCATAAGATAGTATTATGGAGCATTCATATGAAAAGGGTATTTGCAACATTAGTTCCAACttcctgcaagg
gacatgattaAATGATTaatgaaatgtccctttgcataTGCATTTTGAAACAGCAATTAGGCACTGACTGAGAAAATC
CACCAATCCTCTCATTTTTCAGTATTATCTCATTCTTGATTTATAAATCATAGAGAATTTTTGAACAGTA
ATATGTAGTACCTGAGATagtt
>NC_000007.14:114649690-114650138
AGAGGGAATGAATAGGCACTATTCTTTTACATATCACTGGTAAACAATAGcgaggaaaggagaagggagatgT
GGCAGAGGTATGTATGTGTTTTTCAAGTTCTCAGTAATCCACTGGAGGCTGTTCTATAAATGATCATT
GAAGGGCTGCAAGCTAGCCTATAATTACAGGAAAGAAAGTGGCAGCTCTGGCATTTCATAACTATGT
GTCCTCGAAAAGTGCTTTGTGAGTTTGTCACCAATGATAATTTAGATAGAGGCTCATTACTGAACATC
ACAACACTTTAAAAACCTTTCGCCTTCATACAGGAGAATAAAGGACTATTTTAATGGCAAGGTTCTTTT
GTGTTCCACTGAAAAATTCAATCAAGACAAAACCTCATTGACTATTATTGTAGTCTACAGTCTCTATTC
TAATAAAAGCTGCATAGATAAATTGAATAGGGCT

Homo sapiens chromosome 7, GRCh38.p14 Primary Assembly

NCBI Reference Sequence: NC_000007.14

GenBank FASTA PopSet

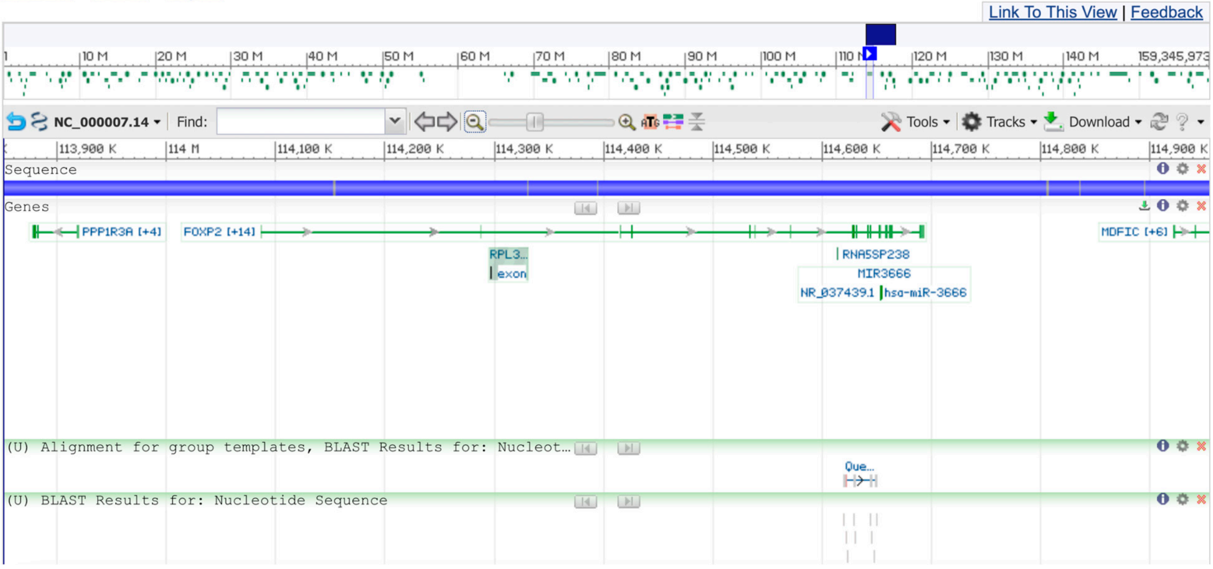

Homo sapiens chromosome 7, GRCh38.p14 Primary Assembly

NCBI Reference Sequence: NC_000007.14

GenBank FASTA PopSet

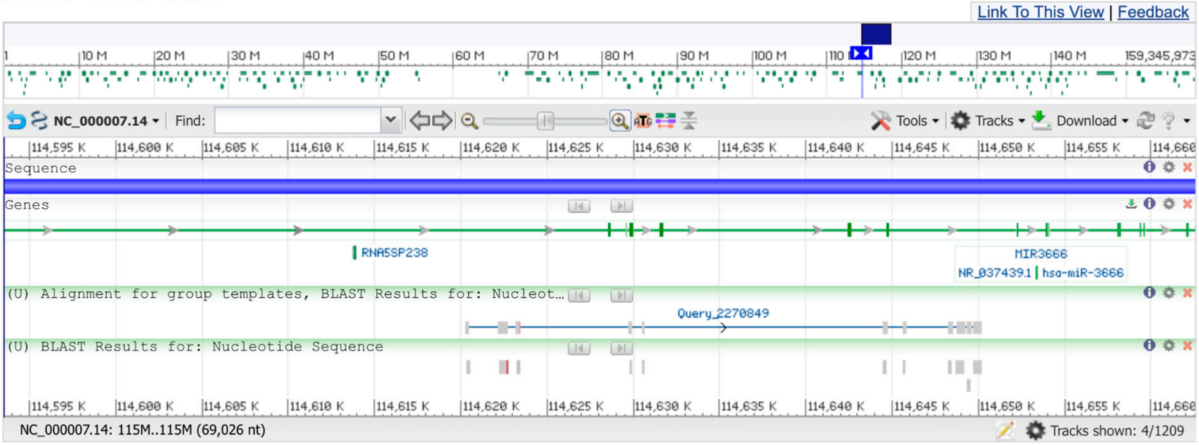

The same FOXP2 gene as previous cluster

chr7 CLUSTER 32 positions on BED file: 5875 - 5891

>NC_000007.14:114649690-114650138

AGAGGGAATGAATAGGCACTATTCTTTTACATATCACTGGTAAACAATAGcgaggaaaggagaagggagatgT
GGCAGAGGTATGTATGTGTTTTTCAAGTTCTCAGTAATCCACTGGAGGCTGTTCTATAAATGATCATT
GAAGGGCTGCAAGCTAGCCTATAATTACAGGAAAGAAAGTGGCAGCTCTGGCATTTCATAACTATGT
GTCCTCGAAAAGTGCTTTGTGAGTTTGTCACCAATGATAATTTAGATAGAGGCTCATTACTGAACATC
ACAACACTTTAAAAACCTTTCGCCTTCATACAGGAGAATAAAGGACTATTTTAATGGCAAGGTTCTTTT

GTGTTCCACTGAAAAATTCAATCAAGACAAAACCTCATTGACTATTATTGTAGTCTACAGTCTCTATTC
TAATAAAAGCTGCATAGATAAATTGAATAGGGCT
>NC_000007.14:114653410-114653534
GCGTTTCACACTGCCTGGTCTCTGTCACTCTATTGAATTAGATTGATGATGGCAGATTGCAGGGGGC
ACTAACTGGACCTCAGTGGGAATGCATGAAGTGTGTAGACAATCCTGGCAGGCACTT
>NC_000007.14:114653535-114653734
GCTTTGAGAACCCTTCACCCCTTCACAGCTGTTGGCACTAGTCCATTGCTAACAGTGTCCACAGGAC
TTTAAAGAGACACCATGGGCCTTCTAATTTATGGTTTCAGTAACTTGTTACTGTCGAGGCAACTGTGA
CCTCAATTCTCTTATTGACAAAAAGATGAagtgttatttgttttctctgacCTTTTAACATAAG
>NC_000007.14:114653940-114654045
GAACGTCTTCAAGCAATGATGACCCACTTGCACATGCGACCCTCAGAGCCCAAACCATCTCCCAAAC
CTGTAAGTGCATATTGCTTTATAAACAGTAAATAGCTC
>NC_000007.14:114655218-114655522
TAATCACAAGACAGAATAATTGTCTCATAAATCCATCAGGAATCTGCAGCTTGTTCTTTTCCAATTAAT
TATTCAatcagtaaattttttaaaactccagcAGCATGGTTACATCTACACTAATAAAGAATTTTCAGCCTATAATCC
AAAACAGAACCTGAATGAATAATGTAATTGAAGTATTACATTACATAAACCACACATGAAAGAATTGCT
AATTGCGTTTGATGGTGGGCAATTAATACAGAATCTTAGATGAGCGATTTAAAATGAAACCACAGTTT
AAAGTTCCCTTTGGAACTAG
>NC_000007.14:114655604-114655972
accctaaaatttataCACTGTACAAAAGAAGCTTCAAAATTGATATGATAGTAGAGATTTATGGTCtcatgtgcagt
ggctcagttGAGACGGCCCacacatttgataaatattaatagCATGAATTTATTACCAAGGAGAAATGAGCTCTGTTG
GTTCATCACTGCACCCTTAACAGCTATCAGTACATGAACACAAGGTGCAACCGCACTGTCTATTATAT
GACCCCATTTACTCTCTGAATGGTACTTCATTAAATGGTACCTTTTGGCCATGCTATGGATGGATGAA
AATCAAAGTTATCAAGGCTGCGAGTCCTGAATAATGAGTTTCACCCAACCTTGACTATATTCAGATGA
GTCGAGGTG
>NC_000007.14:114656642-114656937
CAGAATAACAGTTTGTGGTTTATTGGGAACAGATGCACTTGGGGATGCCTTCAGGGTGCCAGTCGTT
ATTATTAGACTGCTAATGTGCTTCTTCTGGCTGCTGCCCAAGAAAGAAGAACAATTAGCTGGcatatgtta
atttttgtttccctAACAAATCTCTCTACTGACTAAATGTGTAAAACAAATCCACAAAGAAAGATCAATCAATGC
TGTACACATCATTTTCTCCCCATTAAAAAAAGGTTTATCTTAAGAAGTGTATTTGGGATTTAAAAAGAC
AGGTGTTACTAAA
>NC_000007.14:114657247-114657633
CTGTTAAACAGCTTGCATACATtctcaccttttttcttttttctaccctGACACCCACTTTCCAGGGTACATCAGCATTA
AGTGACACTGTaatcataaattgaaaatgatACTTCCAGAGGAATTGGCAAACTTGACATTTCATTATATTTGTT
AACACATGCCACAAATGATTGTTAGTGAGGAAATTCCAtttccctctctccatctccaatcagatttaatttgaaattttcagcc
tCCTCCGGCTAATTTGCAATTAAGACAATTTTGTTTGAATATGTAGTAATGTCATTACCATTCCACCAAA
TTAGCAAGGAGACTAAAGGTCAGTGTAAAATTTTCCAGCTGGCTGGAGCCTCTCAGCTGAGATTTGG
GActacga
>NC_000007.14:114657855-114658287
AGATTATTAGATAACCTCTAACAGAATCCTCTAATTAGAGTTCTTATGATACAATTTCATCCTGTAATTA
GTTGAGATTGGCTGCTTCCTTTTGCAGCTTATTAGTGGCAACACAGCTGTAGAAGTGAATCCACTCTC
ATTTGTCAAAcctttttaagtctttttctcTTGTCTCTACCTTTTTCCTGCCCTTCTCTTGGGCCTTTGCAGCTAAAT
CTGGTGTCTAGTGTCACCATGTCGAAGAATATGTTGGAGACATCCCCACAGAGCTTACCTCAAACCC
CTACCACACCAACGGCCCCAGTCACCCCGATTACCCAGGGACCCTCAGTAATCACCCCAGCCAGTG
TGCCCAATGTGGGAGCCATACGAAGGCGACATTCAGACAAATACAACATTCCCATGTCATCAGGTAG
GATATGAATGCTCAGT
>NC_000007.14:114659548-114659679
CTATGTATCTTGTCTCATTTCAGGCTATCATGGAGTCATCTGACAGGCAGTTAACACTTAATGAAATTT
ACAGCTGGTTTACACGGACATTTGCTTACTTCAGGCGTAATGCAGCAACTTGGAAGGTAACT

>NC_000007.14:114659719-114660071
CAGATAGCACAAGGAACATTTATTTACATGACATAAGCAGATTAGTATCTGCTTCCCCTCTTTTTAACC
TGCCTCTTGAATGGAATGAATTCCCTCTCTCAAAATGACAAGTGAAAGAATAATTTTGCCTCTGATATA
GTTTTCTAATTTGGTGCAATTAATAGCTGAGGCTTGGCAGAGAAACGAGGGCCTGACACACTAATTA
CAGTGTGAGCACTCAATCATCAACACCTTTGTTAGTCGCACGATATTACTTTTTCTCTTGCATATTATT
GGCTAATTagtttgaaaaatgtctgtttggCTTGTGCAACAAATGGAGGCTGTAGGTTTTGTCTTGGTCTGCGCC
TTT
>NC_000007.14:114660112-114660252
GGAGTGAGGGCCATGGCTACTCAGCTGGAACTAAAAGAAAGTAAAGTGAATATTATCCTGTCAGAAC
ATAAATGCAGTTTATTTACTTAGACAAAAGGGTTCATCTATATCCCTGTCCAAACAACTTCATTGTCTG
AATC
>NC_000007.14:114660283-114660481
TGCATCTATTTTTGGAAAAGAGCAACGTCTGGAAAGATAAGGGCACTCAGCACAGACTAAGTgaactgttt
tattttcatttcaaaaagcaGTCAGAAACATCAATTAGCCACAAGCCATTTGGTACAAAAGGGGAAAATGTTTGT
AGCTGAGAAGAATAGAAGCAGCAAAGCACTCATCAGCATACTGAGAATTTTA
>NC_000007.14:114660786-114661064
AATGTAGTGAATGTTCTGTCATTTGATttactgaaagaaattttaaaagaaatgaaagatgattTAATATCTTAGCTTGG
TCTCATCTCCACAGCTCGGGGATCCAGCAAAACTCATTCTGAACAGCTTATTAGTTCTAACTATATCG
CATGCATAATAAAACTGCTCATTTgctcattaatattaaaattatcatattCAGAGCCATGGGCATTAAGATCAATCA
AATCCTTGGATTTCAGGTCAGATGCTAGATCtgcttatgctttttttttt
>NC_000007.14:114661519-114661861
TTACACTGTAACTTGCATATGTCATCTAATAATACAGAATAAACAAACTCTGTCATTTCATTATTATAGG
GATGAAAAGAATGCCTTTGAGCTTATTTGCATAAAGCTTTGTGCAAAATTAACACTTGTCACTGTAGC
CTTTTTAGCATTTTAATACCTCAAGCAGGACTGGTTCCTTTGGACATTTCAACCATCCCTGAGTGCTT
GATCAGGGACACAACAGAGCTGACCTAATTGTTCTGGCATTTTCAAAGATACTGTAATTTGTACAGAT
ATTGCAATTTAGACCAGTTCAATGAAAGGAAGGTTTTGAcacagctattattaaaatagCTTGGAAGG
>NC_000007.14:114663787-114663960
CTATGCATTAATATTTATCCAGGCTGTCATAAATGATTTCACATCATAGTGTTGGGGAACATGGTGCTT
GTGGcaaaaactttaaagtttaattataatatataatttttatgcagAGGAGGCAAATGTCAGGACTTCTTTATTTTGAGTTA
AGTtctggtcatttttaaaaa
>NC_000007.14:114664878-114665113
GTTTAACTGTTACCCACAGTTTACATAGAAGCTAGTAAAGTGTAACTTTTTGCCTGTCTTATAAATCAG
TGTAATTATATATTCAACTTTAATTACTGGTGCGTCTGTATTGGCAAGGTTTATAAGTTATATTTTATGC
AAGACTACGATTCCTATTAATATTTAGCAGCTAACTATATCATTAATTACTCTAAAATCTTTCCCCTACC
ATCTTTTTAATGGAGCTGAGAGAGTA

Homo sapiens chromosome 7, GRCh38.p14 Primary Assembly

NCBI Reference Sequence: NC_000007.14

GenBank FASTA

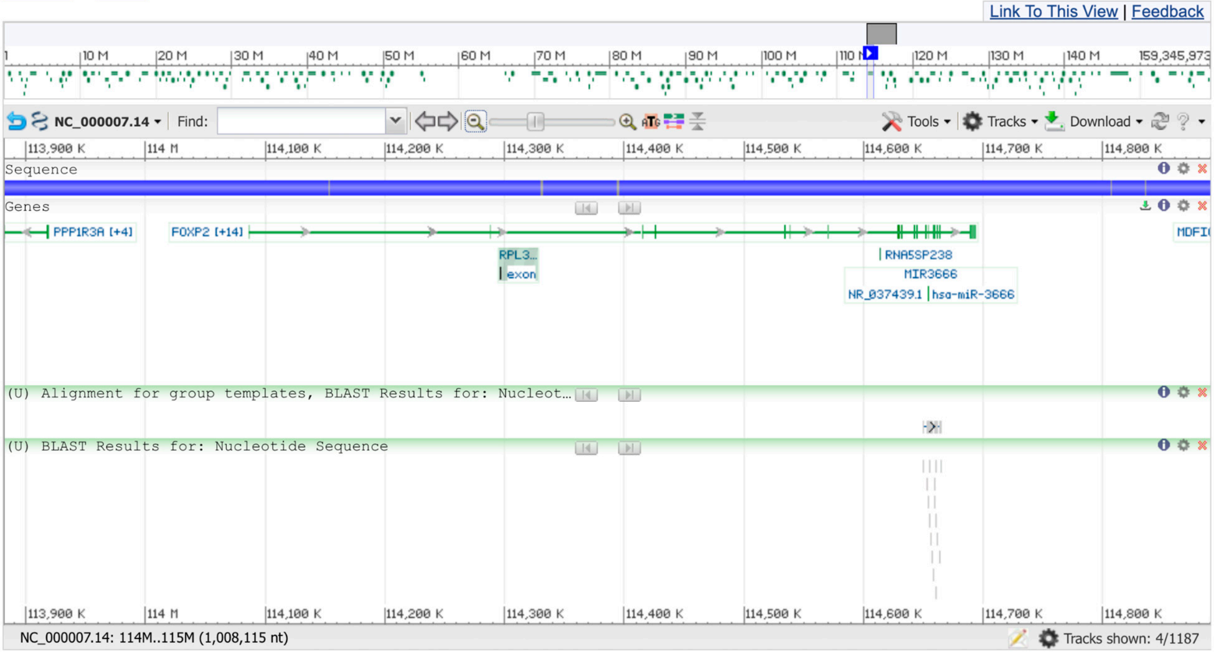

Homo sapiens chromosome 7, GRCh38.p14 Primary Assembly

NCBI Reference Sequence: NC_000007.14

GenBank FASTA

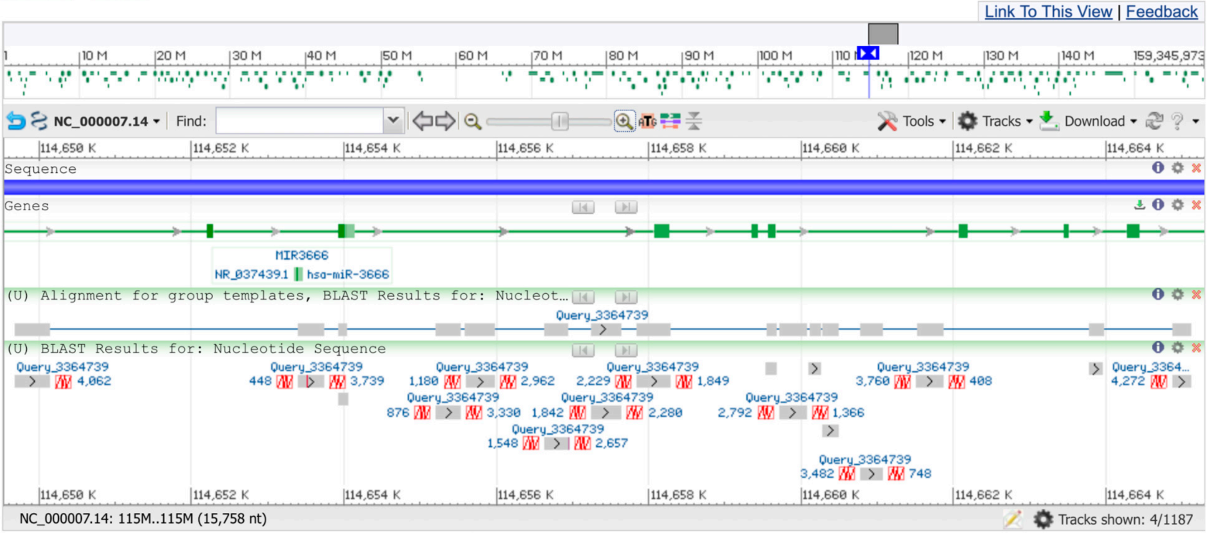

The same FOXP2 gene as the previous two clusters

chr8 CLUSTER 33 positions on BED file: 6064 - 6074

>NC_000008.11:25909452-25909574

GCTAGGCCTATTTCCCTTTCTGTTACTATGACAACGAAATTTTACCGCAGTCCCAAAGTCCCTTCAGA

ACTCTCACATCCATGTCTTGAATTAGAACTTTAAAGGAAAAGATATACATTTTC
>NC_000008.11:25918299-25918676
AGTGCATTCTCTTGCTAAGACAGGACACCATCCAATGTTTGTTAGCATCATTAAACTAAAACTGACTTT
TCACCTGCTCCCATACCATATGCAGCTGCAAATTTCTAAATTAAACTATGAAGCCATgcataaatttacatatat
tacagACTAGCCTCTCCATTTAACATGCAAATGTACTCCAATGTTTCAGAACACCTTCTGTCGCTAATTA
ATTTGCTGAATTATAATTAGACTAATCCTGCATAATTAAtaagcacagatttttttttaatttgtaagcaGCTTGAGGAG
ATCATTCACTTCCTTCTATAAACTGCTAAGtaagtgttaaaaaaaatctggaaagaacATGTGTGAAAAGGTATGTT
TGATGT
>NC_000008.11:25918870-25919013
GAAAGGATTAAAGTAACCGTATGTTGTATTAGGAGAGATAATGATATTAACACTCCACTGTATATCAAA
ACACTCTGTGCATGCTGGGTGCTTATAAGCTCCTTTGATGGTATTTGGAATCATTTTCATTTTGGCTG
CCAAAA
>NC_000008.11:25931161-25931277
CACActataaatttggaaaaataaataaatgcttcagAACTTCCACTTGAAAACCACagctaaaaatgaaatcaaatctgACCCA
cccctttttcttttaaattgtggGAACAGACT
>NC_000008.11:25931561-25931704
ACCTAGCTAATATTACCATCAATATTTTGTCCAGCTACATTCCTTCCAATTTAAAAACAGTTCAATATCC
CTTTTACCTTCAAGTAGCAGCACTTAATAAACAGTGAtggatggtttttatttttgttcgcATTTCAGTCTTC
>NC_000008.11:25931813-25931931
GCCCATCGGCTACACAAACATACAACTTCCCATCAATTAGGCAACACAGAGATGTTGTTAAAAATCAA
TGAgcttattattaaatatatttttttcatgtagcAATAATAGTTATTGC
>NC_000008.11:25932641-25932796
CCGCAGCTGATCTGAACTGAGTCCCCCGTAATTACAGGAGAAGGTGGAACAGGTTTGCCGCTAATT
GGAAAATTCTGCAAATGGCAGCACTGAAGTAAATTGACAGTCGTTAGAATATTAGCAAAATGAGAGTA
CTGATTAGTAAAAAGCTCaga
>NC_000008.11:25935271-25935496
AGCAAGTCACTTTCAATCAAGTGTCACACCTTTAAATAGTGGTTAAATCTTTGCATTATGCAGCTCATC
CCTTTACAACATGGGGTTCCCTTAGAGAATTCCCTGTTTACACTGGGAATTCCTCCAGGGCCGCATC
AATAAAGCTGAACAGATGTCAGGGTGGAATGCCAGCGGCAGTGGCTGTTGTCAGGGGTACTGGGGA
GGGAGGCACAGAACAATGAGAAA
>NC_000008.11:25935766-25935879
GTCGAAAGTGGGATGTAGATGtccttctttttaattaaaaacatgattGATGAGATTTAACTGACAGTaggaaatgcaattta
aatattCCTTAAAGAGGCACTAACCCTGTT
>NC_000008.11:25937731-25937844
GCTCTACCACTGTGTACATTAAATAGTCATGTAGTTACATTAAACAGTGgggagttcacacttcacaagcACTTG
GTGTTTTAAAACAGATGTGGGCTGTCTGTTGTCAGACT
>NC_000008.11:25938103-25938363
GGGTGTCGTGGGATTTACTGACAGGAGAATTTTTGACAGTTGATGGACAGATGAATTCCGTCATGGA
AGCGTTAGTGGAAAGCAGCAAGGCCAGATGGAAACTCACAGCTTGtctttaatattaattttctttttttcaagagatC
AGAATTCCCTAATTCCCTAAAATGTTTCAATGCATCTTGAGGTATTATTTTTCAGATGCCTTTCTCCAC
CTATTCTTAGTAAGAATGTTCCTTTGTGTTAATGTTATTTTTCC

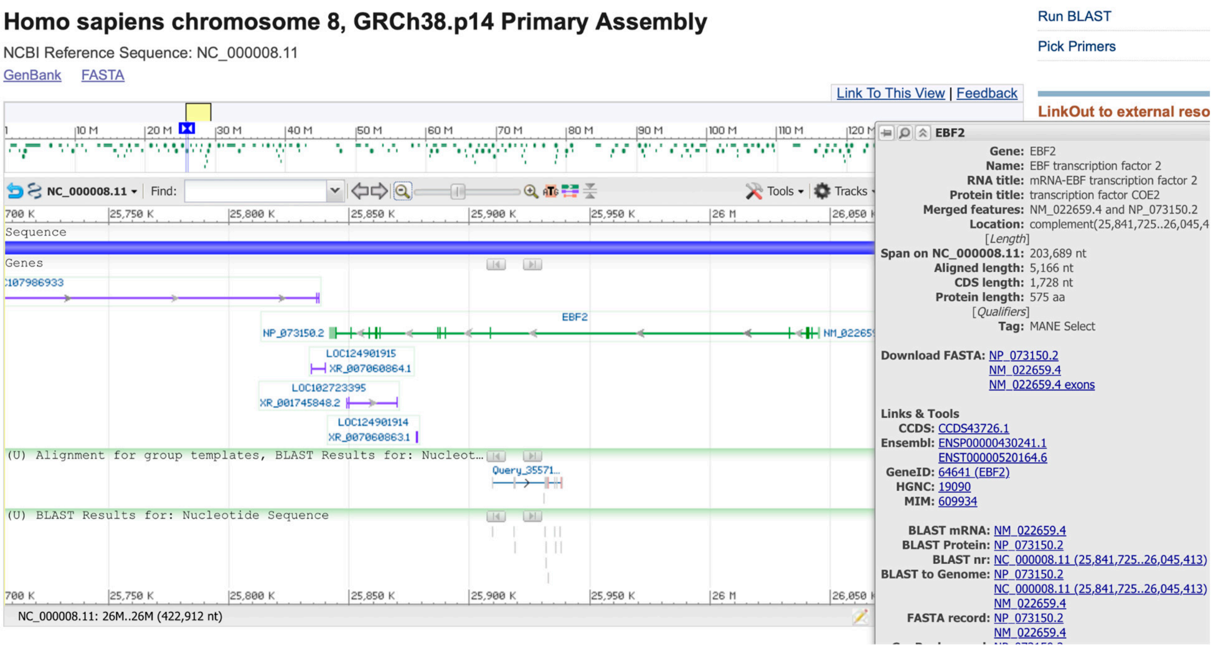

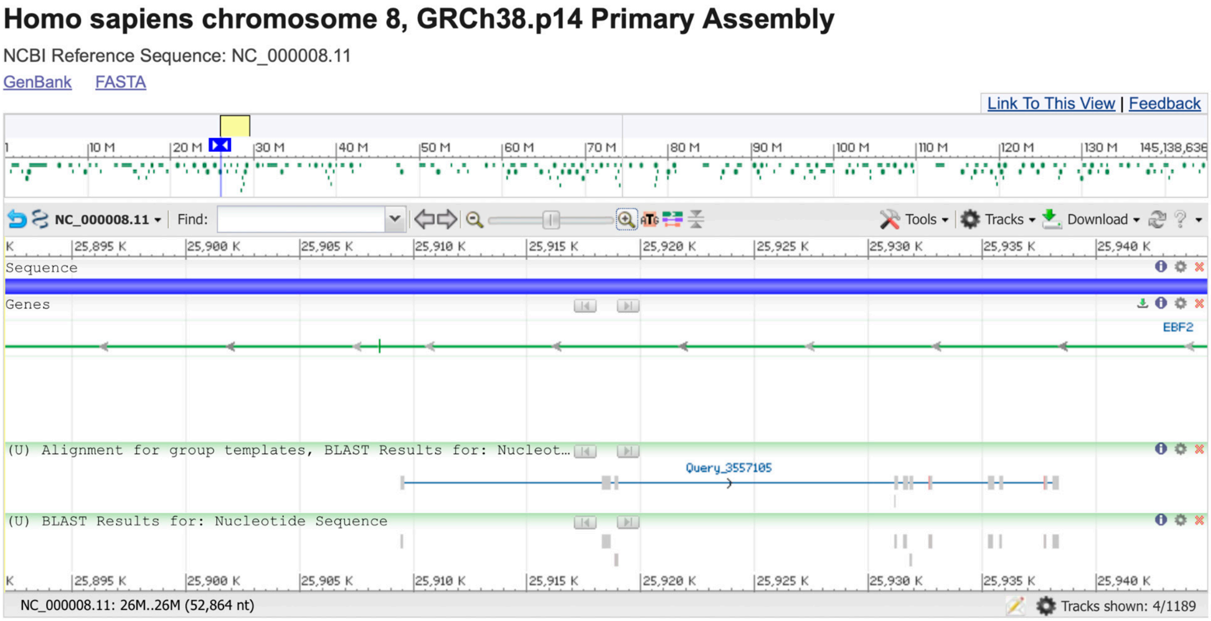

chr8 CLUSTER 34 positions on BED file: 6323 - 6336

>NC_000008.11:76663683-76663815

CTCTTCTTTGCAGTAGAGGTTTTTAATTCAATACAGAGCTAGAATGAGATGGAGCCAATTTCCTGGTC
TATATGTGTGACAAATGCTGAAAGTGGCACAAGATTAACAAATTAGTTGAAGTATAGatcttaa

>NC_000008.11:76665410-76665608

AGCTTCCCAGGCTCATGCTCAGCTCTATTTCTTCCTATTGATTATCTTCCTCACTCCAGCAGGTAGCT
AGCCAGCGAGGCTGTCCTGAGAAATAGCTTCCAATGACTTTATACGCACAATTTACACGGAATTGTTT

AAAGAAGATATTAGCCTCAATACAAATCAGTGAACCTCATTTAGTCAATACTGCACAATTCC
>NC_000008.11:76665767-76666024
CTGAGCCATGTGatagtaatatatttgaaatataaaaacctgagattaaatgtttgatttttgacagtgttcttaaatataataaattcaaAAGG
AAATACTGTTAAGTTAAAATGGAGTGTTTTTCATTAAGTCCAATCAAATGGTAGACAACAAAGGATCTT
TTATTTGAGAATatgaagtgaaaatgaaaaccagaaaacctTTACTAAGAAAAGGTCCAGCTAAATTATGTTGGAA
GGAGGTCTCCAGATTG
>NC_000008.11:76669966-76670233
CCAACCAACCTCTTCTCATGCCTAGCTGCACTGAGTGACACTATTTGTGATGCCCAGTGTGCTGAAG
CTGAGCAGGTTGCTGTCAGTTTAAAAATGACTATAATAAAAGTGACCATGAAGATGGATCTTGTGATT
TTGCGTTTCTAATCAGTCATCTGTGAAGAACACTTTTGCTTTGATTCAGCAGACTGGTGACAGCCATG
ATTGATAGTGCACGAGGATGAAAAAAGGTTATTAACTGTCTTCCTATTAAGCTCAATGTATTAC
>NC_000008.11:76674776-76674933
GAGGTTCTTCCATACATGTCAAAGGGTTGTTGAATTATTCATTATTGTAATTACTATCATCATTGTCATT
ATCCCTTTCCAtgtggaaataattttattatcgTAATTTGATATCTGAAGAATTTCCTGTTAGTCGTttgataatcattttttct
g
>NC_000008.11:76674935-76675096
gaaaagaaaagttttgtttataaatgagatagagaaaatgttttaaatgctttGATGTTTGCTATAGATTTCCGTTTATCTAATCG
ATCGGAATGCCAATAAAATCGATTAAAGGTTTAGTGTTTTACAAACAACCATTTTGTGCTTTTTGATTT
TACGTT
>NC_000008.11:76680110-76680269
attcAGTAGGGGGTATTTAAAAGTATGTCTATTCCTAGTTTTAGTTAATTATCTTTTAATGAAAGTGTCAC
TCATATTCACATCTGTAATATTGGAAAGAGATAATTAGATTTTCAAATGAGAATCAGCCACCTTAAATT
TTGCACCAGAAAATACCAA
>NC_000008.11:76681119-76681458
GGCTGCGCGTCATTTGCAGTCTGGGGAATGTTGATCACCTCACTAATTAcacctctctctccccccctctccctctccc
tctccctcgcTCTCCTCTCTCATTCCCAGAGTGCATATCGGGAATAGACACACAAAGACATGCGCACTCAA
CTTAATCAgccattttttaaacagggcTAAAACGATAATAATTAGCAGAATAAAGACATATcggatttcatttcctttcctcct
tttcccaACCCCTTCACAACCAAACAGCGAGACCGCGGTCGGCACATGCTTTAACTCCTCCCGGACCCC
CGAGGACCGCTCCATGCCCCCCACTTTCTGCTCCA
>NC_000008.11:76681524-76681740
cCTCTCGTTTTACTTGGATGTGATCAGctgtaagtaaaataaaagcaaaacaaaaaagaggcgAAGATCGAGTAGGA
ACTGCAGGGGAAATGGAAAGtaagtttttcttaacttttattgagTAGTTTCTGTTAAATTTAGGTCGAGTATCGATT
ATCTTTCCAGCCTGATCTTGCACAATCTTTGATATTtcgctccctctctctccctctcc
>NC_000008.11:76683886-76683986
CGATTGGGACGCGACAGTGACACGGGCATCGATCGCTTCCATTGAGAGCAATGCAAAAACACAGCC
CGGGTATGTAACACTCTCGGTGCCATCAACACtt
>NC_000008.11:76684893-76685415
TCCATTCTGACCATGAGCCTAAATTTACACAATGAAATTTGATTATCTCTAATAACAATATTAGATCCT
GCACTGGATGGATGAGAATTGAGGTGGTTTTGTTGATTGCAAGGGTTTAATGTTGCATTTCAGAATCT
GATGTGGTTTGGCCTCCCCTCCATTAACTGGAAAACCCCTGAGGTTTTACAATTATTTCTTAAAGATA
ATACATTTAAGATTGCATTGGTAGGCTGAAGAAAAGTCATAAAGGCAAGAAGGGAAGCCCTTAATAAC
TCTTGAGATTCAGACATGTTCAGCAGTTAGATTGGCTCTGACTTCACCAAGGGTCGACAATGTGTCAT
TTCCACGAAGCCAAATTGCCCTCTGCCTATATGATATTAATGTGCAAATCAATATTTCAGGGATTAAAT
TTCCCtccttcatttttatggtttctaCCTCAATAAGTCTCAAGTCTATTAGAAGAGGCTGGATGATTTAAAATCTTT
CACTGCAGAATGGCTTGTCGTAGTCTTGTTTCA
>NC_000008.11:76686200-76686311
GTACACCAGACTCGCTTAATTTAGTCTGTTAAAAGAAATGCACATGAAAAAGGACAAAAGGCTCTTTA
ATCTCTGCaagctgtttgtttttcttcaagaTTCCTGTCTATT
>NC_000008.11:76686446-76686605

TAATCTATCCTTATTTACAATTAATCTCAATAAAATTAATCTTAACTATGCTttattcagtggcttaaaatatcTAATT
ATATTCAATTAACTGCTGTCATAGTAATTAATATTGCTTAATTGACTctgcatatatttatgtaaagCTCATTAGGT
GCT
>NC_000008.11:76686997-76687378
GCGCCCAGGGAAAGTGCATGTGAATATGTACAAGTTTCATGTAATTAGTGCTTAATTATATTAACATAT
GCAAATTGTCAACTTAGGAGCTTGTTGAGCTGGCAAAGATCAGCTAGGCACAATTGCAGTATTGTTCT
GAAACAATAATGGATTTCAAGTTGGATTGTGGAAACACATAACTAGCTATCTAATTTAACTCATACCGT
GGTGAAATTTCATTAGTCTCCATGGAGACTGGTTTAATTGTGCTGACTAATGTTCTGGGCTGCAGAAT
GTCCTAAAAAGAAAGCGTTGTTTGCCTAATCCACCTTACAATGACACTTGTTTGTACATGTTTCCCTTG
TGTGAGAATttgcatatgcaaataaatagtTTCTCTGC

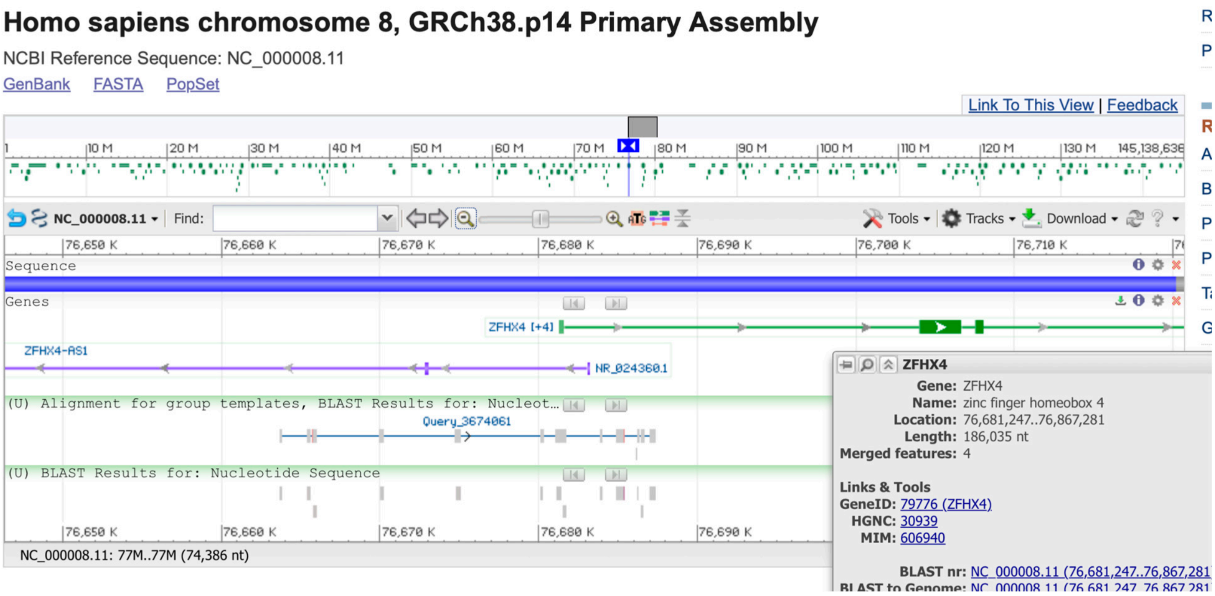

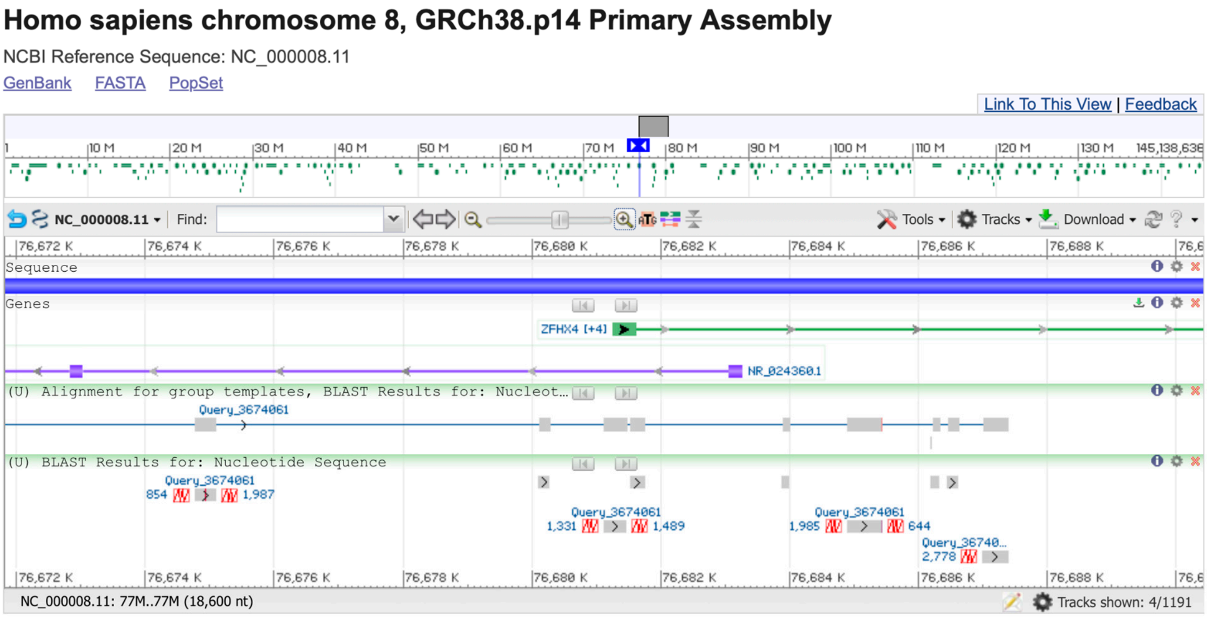

chr8 CLUSTER 35 positions on BED file: 6336 - 6346
>NC_000008.11:76686997-76687378
GCGCCCAGGGAAAGTGCATGTGAATATGTACAAGTTTCATGTAATTAGTGCTTAATTATATTAACATAT
GCAAATTGTCAACTTAGGAGCTTGTTGAGCTGGCAAAGATCAGCTAGGCACAATTGCAGTATTGTTCT
GAAACAATAATGGATTTCAAGTTGGATTGTGGAAACACATAACTAGCTATCTAATTTAACTCATACCGT
GGTGAAATTTCATTAGTCTCCATGGAGACTGGTTTAATTGTGCTGACTAATGTTCTGGGCTGCAGAAT
GTCCTAAAAAGAAAGCGTTGTTTGCCTAATCCACCTTACAATGACACTTGTTTGTACATGTTTCCCTTG
TGTGAGAATttgcatatgcaaataaatagtTTCTCTGC
>NC_000008.11:76695826-76696049
gtttaaatgtataattatcaTTGCATGTCATTTGATAGCTGCACTAATTGCTGCCAACTATTGTCTGTTTGCACTT
AATGCTGAAACCTGATCAACATCCTGCTTTGGTTGATGTTTGGTGGTAAACTTTAATTAACTCTTATTG
CATTGAAGAAGAGTTCAAGCTTGGTGCTAACTAATCATTTACCTTCATTATGTCCTGACAGCTCCACT
TGTTCTTGG
>NC_000008.11:76696344-76696497
attatttttcctgagatACTTTTCCTGTAATGATATTGAAGTTAAAGTAATCAGGTTACCAGCAGATCATGTAAAA
TTCAACTCAGAATTGCTGGCTGCTTGATTTTAATTAGTCTGACATCAAGTTTGTTTGAAAAGGGATAAT
ATGTGGGT
>NC_000008.11:76698957-76699162
GTGTAACAGAAGAGCCCTGAAACATCAGCAGGAAGCTCTAAGCTGGTTAATGCAGTATAATTGTCTG
GACATAGACTGGTCTTTGTTTGTGTGGTTCACTCCGAATCAGCTGTAATTAACTCCAGAGTGTTTCCA
GATTGCcagcaaagaaaaattacagcttttctgttttaaattgtaAAGCTCACAAACATTAACTACGCAG
>NC_000008.11:76699791-76699892
tctgaatttAACAATAAGGAATTTAAGCCTCAATGTGGCTTTAGCAACCAAAGAAAAGAAGCTCTTGGTGTC
TGCATAATACCACTTTGatggattttttt
>NC_000008.11:76699900-76700118
CTCTGTACGTGGCAACAAATAAACAAGTATAATTATACTTGTGAAGGTCTATTCATTGCTTTGTCAGGA
TTAGATATATGTGCCGTTTTCACACTGCGCCTCTCTTTTGTCTTTGCCTAACTCACTATGACATATTGA
TAGAGGATTTGGAATGGGGAAAAGGCCACAGCAGAGACAGTGATGACCTTTTGGAAACTTGATATAA
TTcaaggattttt
>NC_000008.11:76703951-76704227
GTTTACAGCAGCTGTAAATTAGTGATGGGCTATTAGTGAGCTGtgtcattatttaataaaaatggctTCTCTCACCT
TATTTTTTATCCAGGTCCCTGACAGGCTGGATGAAATGAGATCCCCATGTAGCAATTGCCATGGAAAC
CTGTGACTCCCCTCCTATCTCAAGGCAGGAAAATGGGCAGAGCACATCAAAGCTATGTGGAACGACA
CAACTTGATAATGAGGTGCCAGAGAAAGTTGCAGGGATGGAGCCTGACAGGGAAAACAGCTCCA
>NC_000008.11:76704641-76704793
TGTCGTTCTACCCACAGATCATCAACACTTTTCATATCGCTTCATCCCTCGGGAAACCATTTACAGCC
GATCAGGCTTTCCCAAATACCTCAGCATTAGCAGGAGTTGGTCCTGTGTTGCACAGTTTCCGTGTCT
ATGATCTCCGacacaag
>NC_000008.11:76705042-76705158
TCCGCCATAATACAGGGGATTGGCAAAGACAAAGAACCTCTTATAAGCTTTCTGGAACCAAAAAAATC
CACTTCTGTTTATCCCCATTTTTCTACTACAAACCTCATAGGACCCGA
>NC_000008.11:76707307-76707413
AAAATACAAATTTCAGAATGACATCATGCCCAGTAGGAGTAATTAAAGCTATCTGATGAGGGAATTTA
GAAGTTGAAAGGGATGATTGTAATTGATCCTGATTGAA
>NC_000008.11:76707529-76707738
tttttttttcctgtagtaaATAATGAGCTGCCGCCTGAAATCCGGCTTGCCAGTGGTCAGCTAATGGGTGATGAC
CTGTCCCTCCTTACTGCAGGAGAGCTGTCACCTTATATCAGTGACCCAGCGCTGAAGCTATTCCAGT
GTGCTGTTTGCAACAAATTCACCTCTGACAGCCTGGAGGCCCTAAGTGTGCATGTGAGCAGTGAGC

G

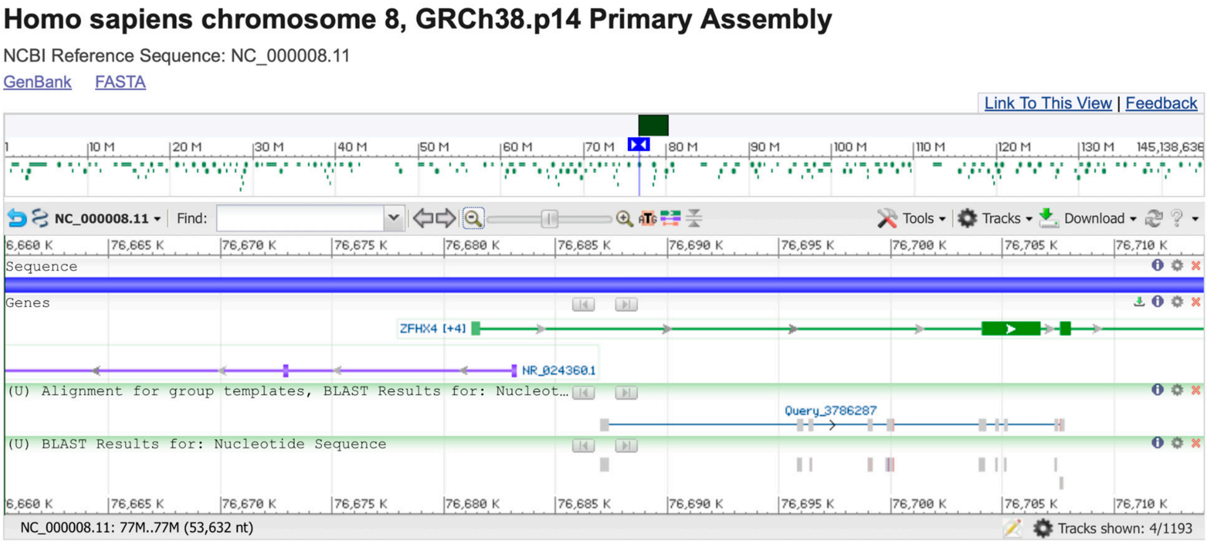

The same ZFHX4 gene as the previous cluster

chr8 CLUSTER 36 positions on BED file: 6360 - 6371
>NC_000008.11:76842903-76843031
CACTACTATTCATTGCATGTGCATTTTGCATGTGATTTCTATCAGTAGCCTCCCATTGACCTATTTTTA
ATCATAACCATCTGACAGAATAGATGTGGATAAGTTGAAGAATATTACAGGACGACCag
>NC_000008.11:76845205-76845324
gttatatttgttgaatattcaTTCATAATAGAAAAGCCTTCTAATGCTTCATTTATATTGTCATTTAAATCTAGTGCC
ACTTTAGATTAATTATAATCCAAATTATAGCCCTGTGCAGT
>NC_000008.11:76847925-76848110
GGTCTGATAGCAATCAATGGTTTAGCTTCATTGCATTTCAAGATGTATTTGCACCCAGTTGTCCAGTG
ATAGTGTCCTCACCCTGGAAATAATTTAGTTGCAAATTGACTGGAAGATGGTGAaaattgaaagttaaaattag
aaatactCATAATAAACAGCAGAAAAAAGGTCAGATGATGCCA
>NC_000008.11:76864088-76864312
AATGAAGCACTTAGCCAACACCTCCAGTCAAGCTTGCACAAAGAGAAAACAATCAAACAAGCAATGA
GAAATGCCAAAGAGCATGTTAGATTATTACCTCACTCAGTCTGCTCCCCTAATCCTAACACCACATCT
ACCTCGCAGTCTGCAGCTTCTTCTAATAACACCTATCCTCATCTTTCTTGCTTCTCCATGAAGTCCTG
GCCTAATATCCTTTTCCAAGC
>NC_000008.11:76864313-76864596
TCTGCCAGGAGAGCTGCTTCtccccctttcttctcctccttccctttccttgccTTCAACGGTTACCTCAAGTTTGTGCAGC
ACCTCAGGGGTTCAAACCTCACTACCCACAGAAAGTTGTTCAGATGAGTCTGACAGTGAGCTGAGCC
AGAAGCTAGAAGACTTAGATAATTCTTTGGAAGTGAAGGCTAAGCCTGCTTCTGGCCTAGATGGTAA
TTTCAATAGCATCCGAATGGATATGTTCAGTGTGTAGGAGTGAAGACAGGATCCcgtgcttaaaaaaa
>NC_000008.11:76864613-76864754
aaaaaaaaataagactttaaCTGCAGTTCCAAAGCTTCTCTAACCCAAAAATTACAGTACCAAATGATTGACTC
AGGATTGTTTTTCCCATATTGATATGCTGGCAATATAGGATGGTATGTAATGGACAGAACTGATGCA
>NC_000008.11:76864798-76865124
aaaaaaaaatctcacaagtTCTTTTGGAACTTGTTTCAAgccaaaaactctcaagaaaGCAAATTGCACCTCAGCTGG
ATTGATTTCCAAATGCTAGCATGTACTGTATGGGAGGATGATCCAGATGTTTCAAAGAGAATTTCTCT

TAGTTTAGTTAGGTGTAATTCAGTAGCTTTAAATTCTCAGGTCAGAACATAACATTTCTCATTTGTTAA
AAGCAGCAAGAAGCCTGGTAAAACTGTGACTTTTCCCCAAACGTCAATCTTTATTAGAAAGCATTTTC
TAGGTGTGTTTAGTGTACAAAGAGACTTTATAACCCTTACTGG
>NC_000008.11:76865201-76865472
TGTGTCTGCCCTGTGTATTGCAGTTTGTATTGCCACAAGCTATATTTATACCAGTGTCACCCTTTTCTT
GTAGAATATACTAATAATCTGTGCCAACTCTACCTTCTCACTTTTACCTCTGAcgtcattcttttttctgaaagagg
TAATAATTCTAGTTTTGATAGACTCTGAGGATTATGTGAACAGGacatttttcatttgtgaatTTAATGCTATACTG
TCAAGGTACTTGCTTGTGTCTGAACTCTAGTGCACTTATGATTTTGT
>NC_000008.11:76865587-76865708
AACAATTGGTGCATAAATGAAAGTAGTACTGTATACTTGAAACTGTTTAAGTACAAGTTgaacaaaaattatg
aaaaggtATATTTGCTTCTCGGGAAAGCAAAgaagctgctttaaaaaa
>NC_000008.11:76866127-76866346
GAAGGTTATTTGTAAGAAAGTTAAAGGCTTGTGAACAAAGAAAGCTAAGCTGTTGTACATATTTGTAG
TTGGCTGTGCATGGtacaaatttattaatatgaagaaatgcaaaatgtattGCTTTTGATATTTCTCTTCCGAGATGAAC
AAGTAGCATGTAATGCAACTGTTTGACAGTTTAACTCAAGTCATGCTTCAAACTGttttaatgatcaaatc
>NC_000008.11:76866462-76866742
tttttttttttagtattttaacatttattttaatcctGAAGACACTTTTTTGATTGTGTTTCGTAAGAGACAACATGGCCTCCTA
AGGTGCAATCCTGCCGCTATAGTGAGCTAATGTCCTGAATCCAAAGGCTTCAGAAAATTGCTTTTGC
CTTTTTCATGAATGTTAAGCAGCAGCATTGTGAGATCGATCTGTCCTGGCAGTTAACACGATGTGCAA
CAGTGTGTTAGCATGGAACAGAACGCTTTTCACAAAACAAAGGACTGTTTTACAAATGA
>NC_000008.11:76866847-76867289
aaataaaaggacagcCTCCAAAGGTTGAGAATGAGAATTGTTTTTTCCTGGATATCAAAGGGATTATCACA
GCGCAATCATTGTCTACACAACATGTACTCTCAACGCCTGGGTTACATAGGAAATGCACCCTGAGGT
TTTAATAAAAGCCCCTATGGCTATAACTTTAAATAAACTAAACCAAAAATGTTATTGATGTTTTATATAT
AGAGAGTAGTCTCATTAGTTTTTGTTACTGTAATGTTTGAAGTCTCAAATGCACCGTATTACGGTAAAT
AACAtggttttgaaaacttttttttattttgtcacagACCTGTTGTCATAGTTGAAATGATGTTTATTGTAGATGGTATTTG
AACTTATTCTTCTGGAAATAGTTCATCAAGTATGTTTGTTGCTCATTGTGATACATTAAAAACTGTATCT
ACATATTTActat

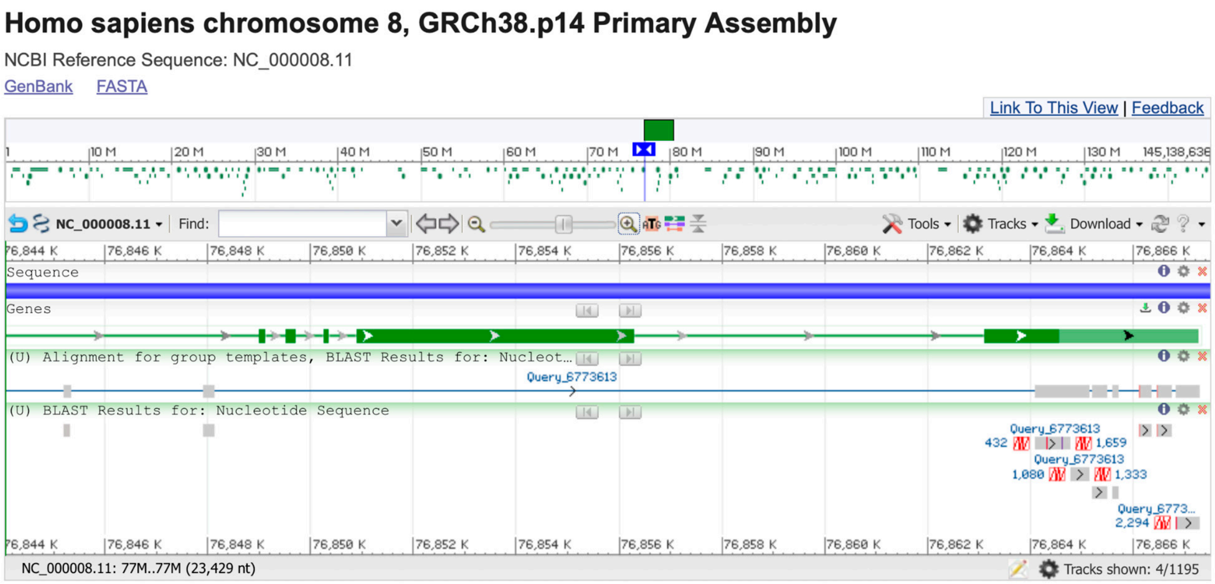

The same ZFHX4 gene as the previous two clusters

chr9 CLUSTER 37 positions on BED file: 7065 - 7075
>NC_000009.12:123769660-123769909
AGGAGACAGATGAAGAAATATGGGCTTTATCAATTACATGGGAGGAGTACCAGGCAGTcaatcatgagaa
ataataaacatgatcaaacttcattaaaaatgaaaaattaatcaaGAATCACACATGGGCAAGCAGCAGGAGCCATGCAG
GAAAGCTCTGAAGGGAGGGAGGCGGTTGGGTTGCTGAGTGTTAACACCCCTCCAGGGAACATCTCC
AGCCTGACCATATGGATCAGAGTGAATAACACC
>NC_000009.12:123770831-123770932
GAATTGATGCTTCtcgcttcatttatttttaaaaccagttcTATCATTCAAGAAATAAACCACATCAATCTAATAACCCA
CTCAATGAAGCAGAAAGCACA
>NC_000009.12:123772800-123772927
TGTCACCACTGGCTTTAGGACATTCGCCACCTGGTTCCATGTCCCCTGGTTCCAACTCTTTTCATAAT
CAAAACACTGCTGACATTATAAACCCCAGACGGGTTTTCTAAAATCCCTCCCAATCAAT
>NC_000009.12:123775697-123775874
taaacttaattgtGCCTCTATTTCTTAGAATTCACACCCCAAGGCCAAAGCTTTCCTGCCACCAGTCCTTCAA
ATCTGACTTGGACATTACACGGGAGGAGGCTCCAAGCCTCCCTGAGGTCTCACTCCTTTTACAACAC
ACGAAAAAGCAGTCGATAATGGGCCCATGCTGAATTC
>NC_000009.12:123775900-123776012
CACAAGCCTAGCAGATGTGATAAAAACTGTGTCAGAGAAGTGGCTCTAGTTTGTGCCTTAATGACAA
CTTAAACCTCTAAACCAATACAACCAAAGGACTAACAGAACTGGC
>NC_000009.12:123776708-123776876
ttttttcagaactgGCATAAGAAAAAAGTGATCTTTGTCAGACAAGGACAAATTTATGCAAATCTTTTATAAAc
attcacaaacatttatttagcttTTGAAATGTTGGCCTCATTTGAGAGCATGAAAATGAGGGGGAAGTAGGAAAAC
GCAAAGGACAGCTTAGA
>NC_000009.12:123776883-123777119
ACGCTACATAAATATTCAGAAGGAAGATTATAGGATTCATGTTAGGCTGTCTTAGGCTTCTCCAAAGT
CCACACAGAATAGCAAAGAACAAAACTAACGAGAAGTACAGCAGATGTTAGACAATGCATcagcctcttttc
ctctctcactTGGCTATACTTGGAcgcttttatattttctttcaatatggTTAGGTCATTCAAGGATTCTCCTATAGACTCTG
TATTGTGGCTACA
>NC_000009.12:123777237-123777436
GGAGTATTGGAAGCCCCAGCCTTCATTTCTGTATAGCTGTGTTTGCCTCAGGGCCACGCTGCTTGTT
TTGACAGTGGATCCCCCTAGATAGTTGGGATTAGTCGAGCTTGTTTAGATAAACTTGAAATCAAGGCC
TTCTCTAGGTAGACAGCTAGATGTAGCTACCGAGTTTCCAGCTGCCTCTTCCTTTCATCCCAAA
>NC_000009.12:123777441-123777593
TGCAGCTTCACAGTAGCTATCACAGCCACAGGCTATGTCACATACATAAGTACAAAATTAAAGGCTGC
TAAAAGAAGCTTTTGTAAGTCTTAGTGCTAAAATAAGGAATCTGTACTGCTTGGTTTATACAAATTATT
CTAGCACTACCAATA
>NC_000009.12:123789486-123789898
GATCATTTATTCTGCTTCTAAACGAGAAGCAAAAAGATGAAACTGATGAACTTTTACTGCCCATTTGTC
TTTAGCAGACAATTAAGACAGAGAAGATCAAAATGGATTATCATCCCACGCAGACAACTGGCCCAAC
AGTAATACATCTCCGGGACCACAGATTTGTAACTCTCTTTTATCCCTCCCCTTTCTTCTTACTTCTTTT
TCCACTCTAAAGGCAGTGCTAGCTTTGTGGCTCAAGGAGAGCCAAAAGGCTGGTCATCATTATTCAG
TCACAGATGTCAATCTAACCTCCCTTTTTTCTCAGTTTCTAAAGCAGTGAGCCACAATAATTTGTGACA
GATTCTTTTACTGTAAGGTACAAAGAACACACAGAATAAAACATCTAGCAGCAGCAAGCGTCTCTGAT
CAA
>NC_000009.12:123792507-123792782
GTAAAAAGAACAGTAATAATATGTTGAACAACTCTGAAATAAATTTTGTCATGTAAAAAATTAATTACG
CATTCCGAACCTGGTCACTGTAGTCCTCCGGGAATTGCCTCTGCACCTCAGGATCTGTAAATAATTG
ACAGATAATATTAATTGGCTTTGGATTAGTGAGCAAGCAGAGGATCACACATTAATATTTGATCAGAA

GATGATAGCAGCCCTGGCAGGGGATCCAAGGGGGGCAGCTGAGGCTGCTATGTTGATGAGCACAA
GGAAGG

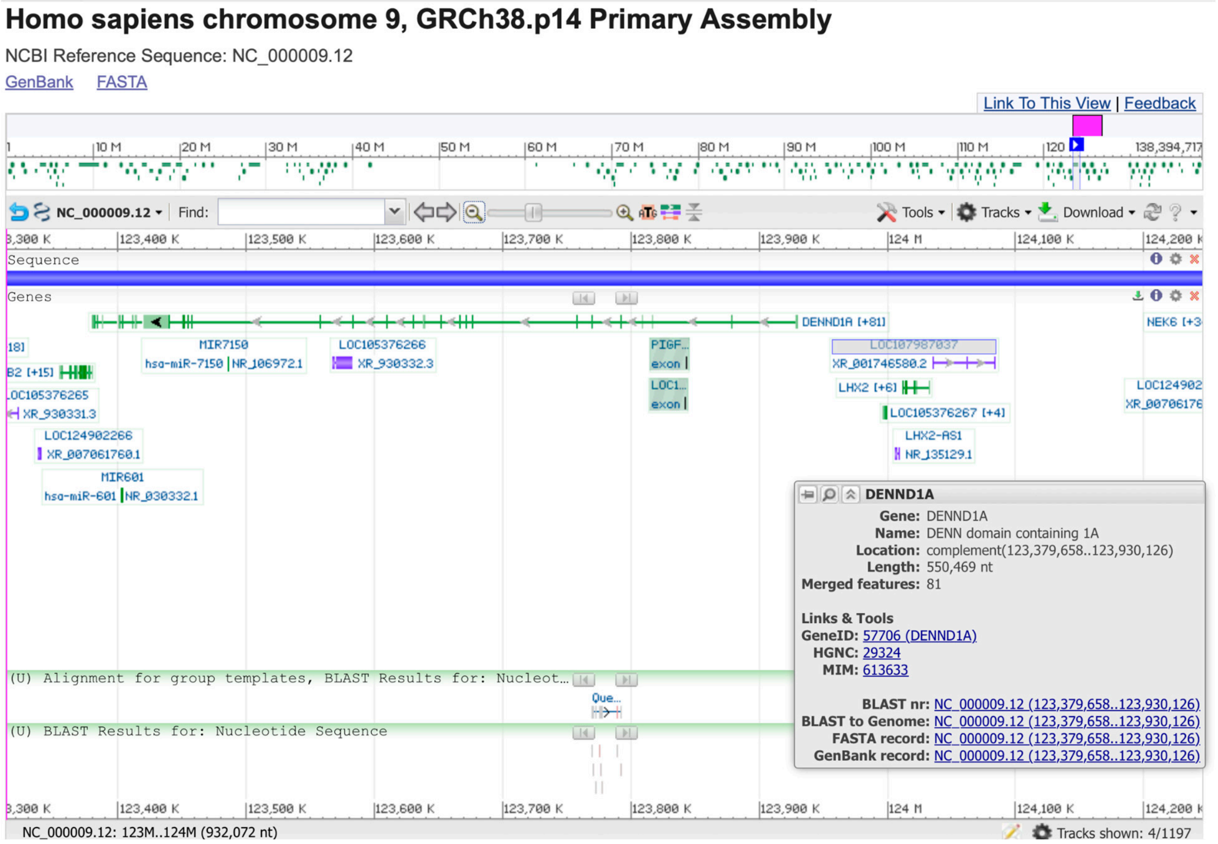

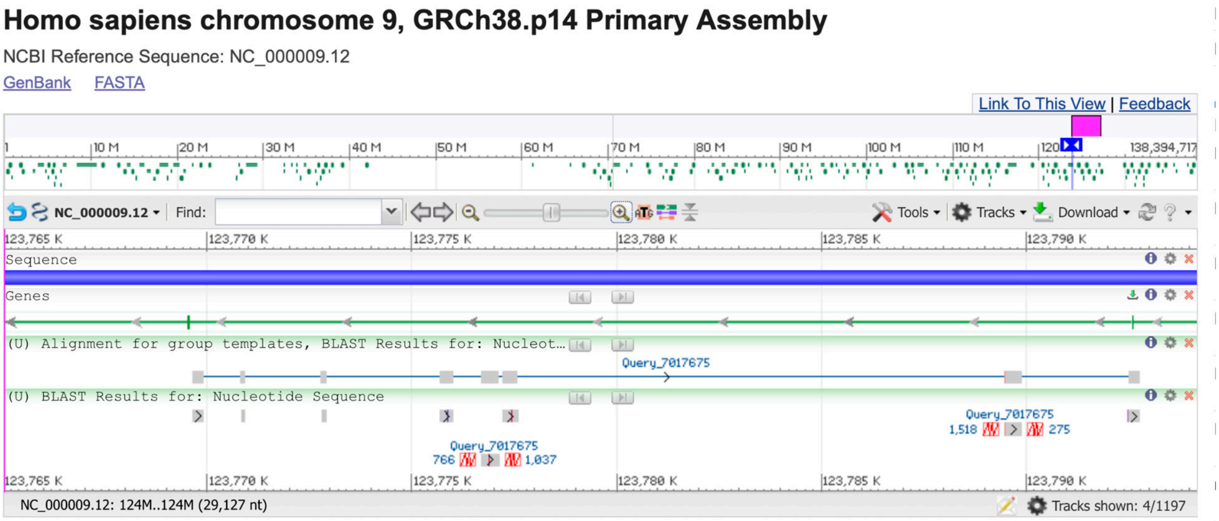

chr10 CLUSTER 38 positions on BED file: 7373 - 7386
>NC_000010.11:61892224-61892480

gaactattaatGTGATGTTCTGGTTGAAAATTTAAACTCTCAAAAAATCTGGAAATTCAAAGTCATGGTACC
CATAGCAACCATAACTCTGCCCCCTTGCATGCCCAGTGTGGAGTATTTTGTATTGCAGTACATGCTGT
GAAATCTATGCCTAAATGTGCTATATAGCAGAGTTACAGTTGCTGTGGGAACTGTAACTTTGAATTGC
CTGCCTTTTGTGTCATTAAACTTTCAGCTGAAGTATTGCATTAAAGGa
>NC_000010.11:61897821-61897960
AAGCTGCAGTGGCAGATTCGAGCCACATGCGGTAGTGTGTAGCAATTAGTAGATAAAATGCTGACTA
AAGTGCTAAAACATGAAGTATTATTGTAGCCAAGGTCAAAAATGCTTCAGTTGTCTTTTGCaatttggggga
>NC_000010.11:61898549-61898740
GGTGTCTCAAGTCTTACATCTGCTCTGTGATTGATGGAGCCTTGAATCCACGTTATTACTCTCTCACA
AGCAGAAATCAAACAGGCTATTAACTACATTACTTCAAAGAACTGTTTCAATACAATCTCCTTATCttaatt
gaaactttctttgtaTGGATATTTGCTACACAGATAATTTACAGGTGCCTA
>NC_000010.11:61899994-61900188
TCTGCCGGGTGTATTAACTTCAGCCATGtgaatataattttcattaaagTAATCCTGTTTCAACTAGATGCTGTGA
CTAGAGGTGAAACGTTGCGAAAGCGTTAAAAATAGCTTACTAGTAATTTCAACATCCTGTAATTTTATC
ACAGATCAAGTTTCAACTGTCATACTATACAGACCTTATTCCCCATATA
>NC_000010.11:61900396-61900580
TGCGAGGGCTTTGCAGTCTCTGCCTGTTCCTTGTTCTGTCGCTCTTAAGTTTCTCTGTGTTTGCATAA
TAGCCGTGCATGCAAACCTCGCAATGCTCCAGGGCTCCAGaacaataaatatacacatttaaagcCTTTATTGTC
TTACGGTTCATGCCCCCGGTTTTCAACAGCTTAATTTCTGG
>NC_000010.11:61900667-61900815
tgggggtggggtaggaGTCGGTATATTATTCCTGTAGCGCTGGGTTATATAAACACATTATCATTTGAGAGC
GCCCTTGGCGTCCATCAGCATTGTAAACACGTACTAGTGTATATACTTCAAAATTAAAGAATGCACAC
GCAGAACC
>NC_000010.11:61900991-61901265
GCGAAGCGAACAAAtgcattaatatttatatgtttatataatcgATAACCCACTTTTCCCTTCCATGTAAATAGGCATCA
AAAGATAATTTAACAGATTAGTTCTCGAAAACATGGCAGTGAGGAAGCGTAATTTAACTATCAAACAA
ATCTACATGTCTAATAACAGCAAATCTGCTAAGAAGCATTAGAAAGAGGAGCAGCGCCCCGGAATCT
CTGCAGGAAATGTTCTTTATATTAGACATATACAAGCAGGTCCTGTCAGATGCACAGTG
>NC_000010.11:61901620-61901764
AATGCGTTTCCCTGCCAGGACCTGATGCAATCCATTCAAGCCAACAAGTTTGGAGAGAATGTTGAGT
TCAATCAATTCAGAACGTCGAGATGGAGCCCAACTCACTCCAGGTATTTCGCTCTCCTCCGCTCCTC
CGATCCCGGC
>NC_000010.11:61902148-61902427
ccccctGCAGTGGGTCGGCTCACCGTGTGGCTTGCACGGACCTTACATTTTCTACAAGGCTTTTCAATT
CCACCTTGAAGGCAAACCAAGAATTTTGTCCCTTGGCGACTTTTTCTTTGTAAGATGTACGCCAAAGG
ATCCGATTTGCATAGCGGAGCTCCAGCTGTTGTGGGAAGAGAGGACCAGCCGGCAACTTTTATCCA
GCTCTAAACTTTATTTCCTCCCAGAAGACACTCCCCAGGGCAGAAATAGCGACCATGGCGAGGTGGT
AAACCTGTC
>NC_000010.11:61902529-61902654
CCTTTTTTAAAGGGGTAGCGCCACTAGCTGGGGCTCGAGTATTTTGCCATTGTCAGAGTTTGGCTGT
CAGCTTTACAAAGAGGATCCAACTGCTGATTTTAGTCAAGATCAAATAACTTGTTCGA
>NC_000010.11:61902857-61903053
aagtaAGTATTTGATATGTTTAAGAGGGCGGAAATTACGAAATGGAGGCAGAATGAGATCAAAAGCTAT
TGAGTTGGCAAAGACGTGTTGAATAAAGTGATAAATGACAGGTACTGGAATAATACATTCAAATAACT
TGCAGATCTGCCCGGGCGGATTTCAAAGCGGTGGTGTAACAGGCACAAACACGTTAAGC
>NC_000010.11:61904640-61904810
CTAATATTATgcatgatttgtttttaatgctaACACGGCTGGTAATTAGCTGTATGATTATACTTGTATATTTCATTA
GGACTTTGGCTGATGTCATTGTGAATTATTCAGccatattaaaacaattttcaatTGAGATAATGACACGCCTCAA
AATTATGCAAATGCA

>NC_000010.11:61911149-61911329
GCTTTCTACACCCTCAAGCATGTTGAGCTACTGTGCAagctggaaaataaaaatgttctaattgTGTCTTTTAAATA
AGCATAGGAGTGAATTAATTAGACCATATTTTGTCTTTAGTTGCTTAGTTACACAATAGTATTCCTGAG
CACGAGTAAGATAAATTGGCTCTTTTTGTGCTGC
>NC_000010.11:61911461-61911594
AAGAAGTTTTATTGTGGTGGTAACATTTGCATTAGTTGCTCAAAGATGTAGTTCTGGGAAATACATAAT
TGAAATGTTTTTGAATTGCTTTTTCTGGCCTTTTCAATCTTATTCAAAATAATAGTGCTTCCAG

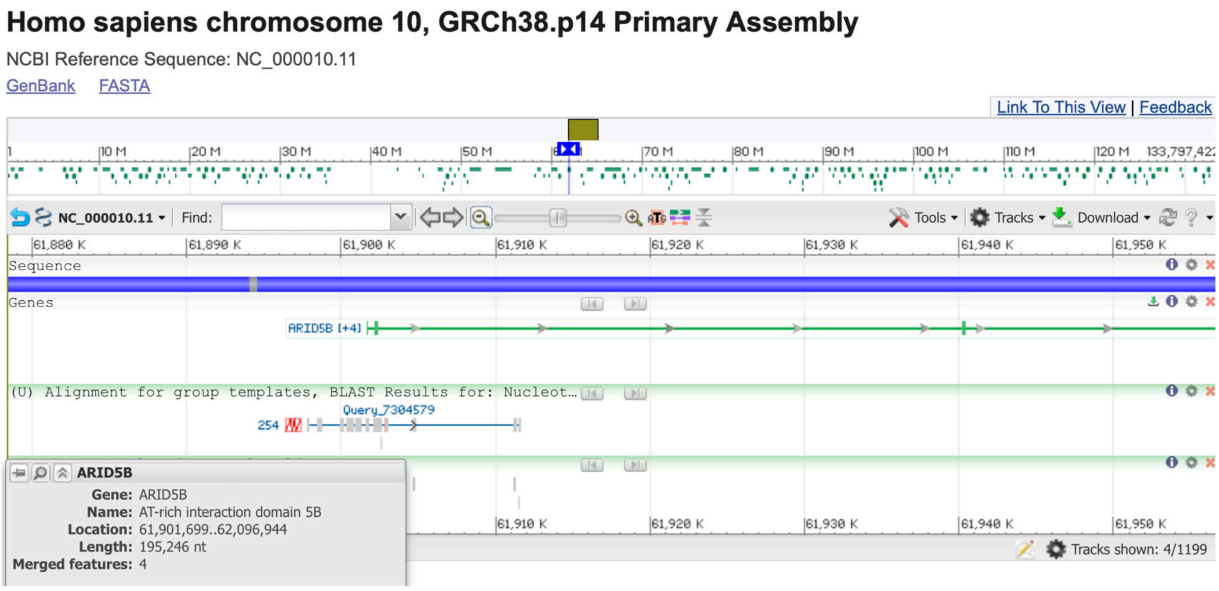

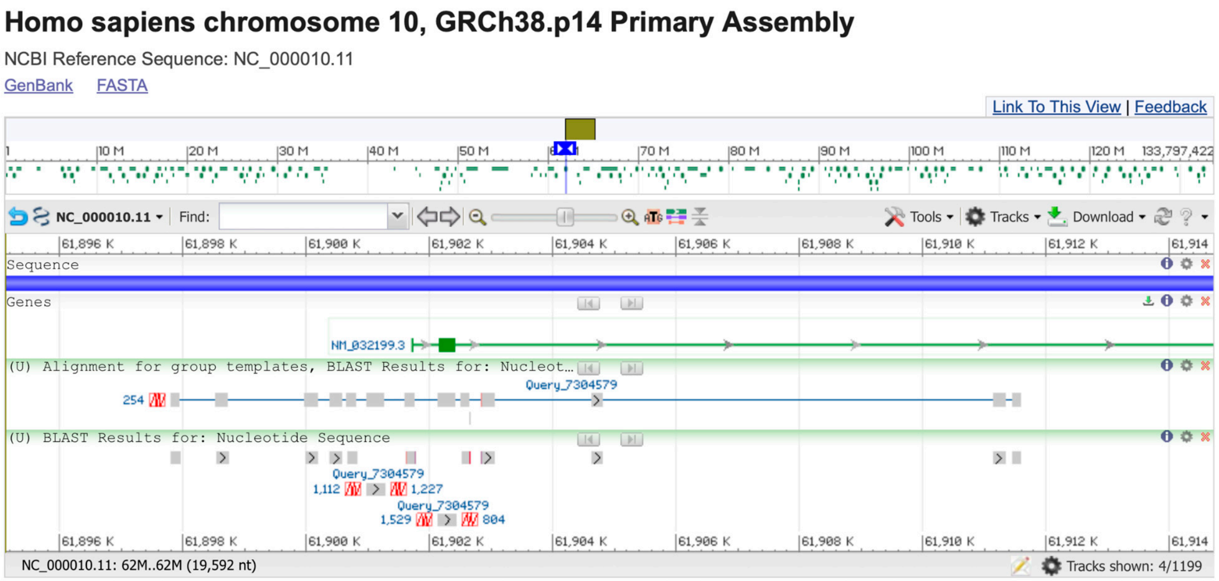

chr10 CLUSTER 39 positions on BED file: 7707 - 7717
>NC_000010.11:101439001-101439335
TACAGCACCCCAGCTGGCAGTAAGCTCTCCCTAGGTTTATGAATGTAGTTAGATGGTCCCCAACTGT

TTACAAGACAGCAGAGGTGAGATATTTGATTGACAAGTCAGAAATGCATTAGATCATTCTCTCTGCCA
GTCTCTGGTTCATGCTGTTAAAAATGTCAGCCTCCAAGTTTGAATGGCAGCCTGAGGGCCTTAgaagctt
tttgttttctgagatggaaacACAAATTGGATGTGTGCCTGAGCGCTTCATCCTGGGCTGTAATTTCATCTTTTAG
CTAAGAACCACAAGGTACACGAGCAAAGCACCCTGTAAACAATAAtactgtgg
>NC_000010.11:101444483-101444725
TTTTTCACAGAGTGTGGTAACTCCTTGCCATAGTGCTGTAGTGTTAAAATTcacattggccaggttgttctGTAA
ATTAAACACTGACAGCTACCCTCAGGGGAACAGCCAATGTATTTATGATGATGTGCTGATTACACAAA
ATACACTGCAAGCAGAAAACAGCTGCAGAGAATAAACCTcttaaaaagaatacaattacAAATAGCACCCTCA
GTATTTCTAGTGGTGAGTGTAGAATA
>NC_000010.11:101445819-101446101
GAGGATGAAGTGCAGTTTGAATTATCTGCTTTTATTTGCTTAATGACGGGGAGGGTGTTAAAGTCAAA
TGTTTAATTAACCCAGCAATGGGCTGCGTTGTCACTTACACACAGCTGATTGAGATATCTCATGGAGA
ATACTGAATAGGAATGATAATGTGGCACTGGGACGGAAGATTGCTTTTTCAATCCATTTGCTGGGTCA
CTGCTTTGAATGTCTTATTGGATGAAAGGCATTCAGCCTGGAGAAAAAATTCAGCATTTAAGTACATT
TTGTTCTctt
>NC_000010.11:101447107-101447243
GCAAGTAATAGACCAAATATAGAGCAGCTATTTCAGGAATCATGTAGGTCATTTAATTATCCATTATGA
TTGAGTGGCTAATGCTCTTTTGGCAATTACATGACCTCTGTTATTCTTGAAACCTATCCAGGCCCTC
>NC_000010.11:101451650-101452197
CCCTGAGGTCCCCACACACCTAGTTTCCCTCAAGCTCATCATCTCTGTCAGGTTAATCAATAGGCAC
CGTATGGCAGAGGGTCAGTAATCAGTGTCATCGTGCCTTTAAATCGTGTTGAAAATTTGCTTAAAGCC
TGGGCCAATTAACATCGAGATGATGAATGGATGGGAAGATGGGAAGAGCCTGCCGCTCAGGGGCTT
TGTGAGGAGGAGATGCTCAGCTGTTGAGCAGCAGACACCAGCGAATAAAATCAGCCATTCATGTGA
GCTCAGTCCACCCACTCTTAATGATAATGTCAATCTagcaaaatatatacacattggAGTTGTCACGAGTTCAT
AAATCAAAGGAGTTTGTCAAAGGCATTCAGATTAACGAGGCAGCAGCAATAACAAGTCAAATTTGCAT
AGAGAATTGCTCGAATCTCAGTAAATTATGATCCTgtttttcatttgattatttgCTAATGTCTCAAGAGGGAGAAT
GATTATATTAGCATGCAAAATCTACTCCAGAAGTAGAAATCTGAGGTGTAGAAGACCAGTG
>NC_000010.11:101452198-101452498
ACTATGACAATTAATCAGTTTCTGCATACTAGCATAAATGCACAAGGCCCAGAAAtgaacttctttttattattatttc
tctttgctaCTGATCCAAATGGTTCAGCTTGACAGAGACTTTATATTGCTTTAACAGTGTTCTGAATTGTGC
CTGCAGGCAAGACATAGTTATGCAGCTATAACCAACCTATCCATCTGCCAACCAGATTGGAATTCTCC
TATCCAAGGCTTCCATTAACCCCCACTGACAGCTCCAAACAGGATTAAGAATTTGGAAGCATAAAAAA
TAGTTGTTCTTT
>NC_000010.11:101461930-101462069
ATAAGATTGAAGATAATGAGAACTGAATTAAAGCTTACTTTCTTTCACAGAATTCCTCAGAGAGAGAA
GACTGTAATAATGGCGAACCCCCTAGGAAGATAATACCAGAGAAGAATTCACTTAGACAGGTATGAA
ATTC
>NC_000010.11:101464140-101464384
CAAAAGTTTTGGCAACTTTATTTAAGCTAACAAACGAATTTTCTCTCTGCAaagttataaattaattttaataggcTG
ATCCAGATGGTCCATTTGGAAAATGGATAGGCTGATCTGttgaaaattaaactaaatttatcTCTCTCATACTTCA
AActgaagcaagagagagaatcaTGCCTATTAAGTCCAGCACATACAGATGGACCAGCAGGTCACACATGGG
ACTAATctgattcatttt
>NC_000010.11:101466344-101466521
gtattctttctcTTGTGTAGCTGGAACCCTGGGGATGCAAAGCCATGAGAGAGGCCAAGAAGCTAACAAAGT
AGCTGTCTCCCTTGTTAGCTTGttcctttattctttaaaaacaagataaaaaccaTCTGGCTCAGGGCATTACATTTTC
CTCCCCCATGCACCTCTATAGATGGA
>NC_000010.11:101467747-101467880
CTGGGTAATGTTTTGCAGCTTGACTTAATTTTAGTCATCTGTTTTAATTACCTATTTATAAAAATGCCTT
TGCATTTGTAGACATTATTTAATACTTTGGAAGAAGCACctcatttgaaataaataaataaat

>NC_000010.11:101468815-101469030
CAAGTCTGGCAATCAATCCCTTTTAGTGTGTTATATGTTTCACCTGACAACTAAACCTTCTATTGATTA
GCCATTACTTTGTTAGATAATCCTATGTCATTGAATCCAttgtaaaaaattaaactctttaaaatgttttacccTAGCAGC
AGTGTGAAAATTGAAAACTTGTTCAGAATTACAGCCTGCTTTCACAAAGAAGGTAGTTTTCAGCA

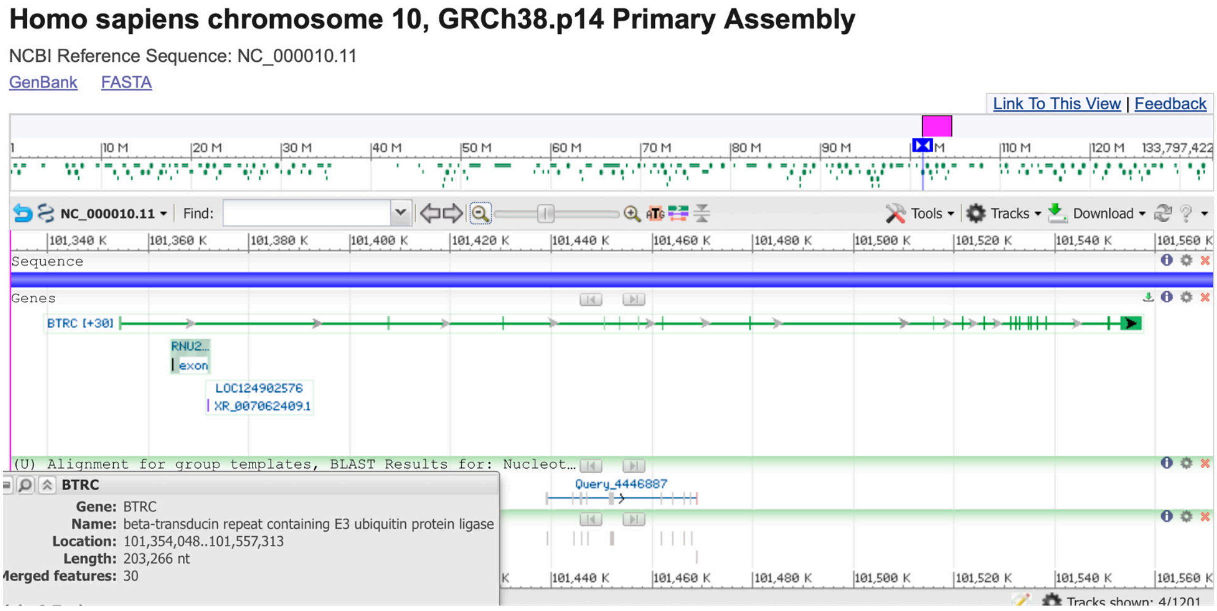

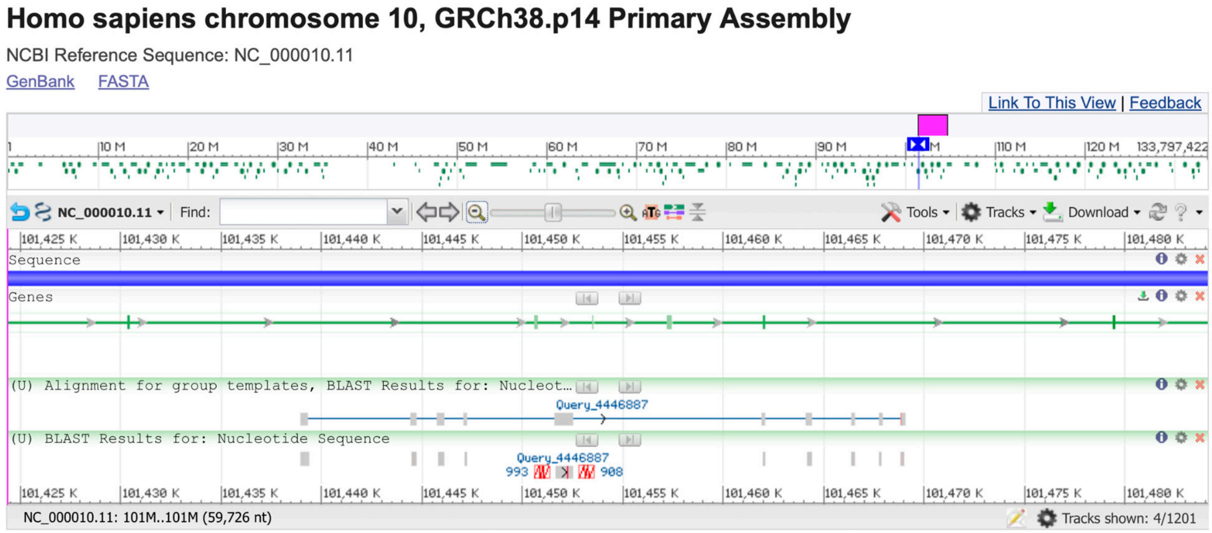

chr11 CLUSTER 40 positions on BED file: 8167 - 8177
>NC_000011.10:31784772-31784907
CCACTGTAGCATAAACACATACTGTATACAGCTTTTATTCAGAATTAGAATAATTGAATCAATGACAGT
GATTTGCCAGGATGTCAAATCTCTCCATCATATCCTGTCACTAccagtttatttttgtgatCAAA
>NC_000011.10:31789184-31789287
ATATTCTTgtcaaatataaatgaaattaacttTATTATAGAAATCATTCTGAGGATTTCTAGGGAAGACAAATACTT
ACATTTTGACATAAAACAAATTGGAT

>NC_000011.10:31795273-31795381
aaaaaatatttatctagtttttatttgaattctgCACAGTCAATTTCATTTAAAGGTTGTGAATTTAGAAGTGCTCGCATTG
TTCTGCAGTTTTATTCAACTATTGTA
>NC_000011.10:31795388-31795568
GCTTCGCCTTGACACCTATTACGAGCACAGCTGTAATTATATCATCACCAAGAACAGGCTATAATTgct
gaaaatggaattttatatttagataaaAGGACTGTCCATTCTTTGTAATGTGTTGCACCAGAGAAAAGtaagatttcttttaa
atttttaacgtgtaattttaaaaaagaa
>NC_000011.10:31796304-31796430
cgcGGACAACAAAAGGATTCTCTTGAGGCGTGCAGCGGGCCACATTGTGTTACAAGAAGCCCAGTCA
ACAGACTTTTCAGTGAAGTGTGTTAACCCCTCTGCTCTGCTATCATTAATCACTGTCCG
>NC_000011.10:31798020-31798121
gCTGGGAAATAAAATCATTCCTTAGTTTCTTAGTGTCTTAATCAGTGAGGCGGAAAACAAGCAAGAAA
AGATAGCAACCCGGTTGCGGCACCTTTTCAGCT
>NC_000011.10:31799373-31799663
AGCGCTTTGCTCCCTATCTTCCCAGTGTCCGTCCTATATTGTTTTCTGCTCTTAAAACTGACATGTCTA
ATTGGCAATTGGTGCCGAATCGTGTCCAGAGGTCTCTTGTGGAACATTTCTACAGCTGTCTCCTTCAA
CTAGACGCTTATTCATGTCGCCTTAATGAGAAACAAAACGTTCTCTAATGAGCAATTACAGAGCGACA
GGATTGTTCCCATAACAAATTCTTGCGAGTGACAGAAGCATCCTTTGTACCAGATATTTCGCATACTG
TTACCGATTTTCCCTTC
>NC_000011.10:31800755-31800864
GTCTGCCCGTTCAACATCCTTAGTTTATCATACATGCCGTCTGCGCCCATCTGTTGCTTTTCGCTAGC
CAGGTTGCGAAGAACTCTGTTTATTGATGACACCTGCAAAT
>NC_000011.10:31801564-31801954
GGTATGTTATCGTTGGTACAGACCCCCTCGGACAGTAATCTGTCTCGGATTTCCCAAGCAAAGATGG
ACGGGCACTCCCGCTTATACTGGGCTATTTTGCTTACAACTTCTGGAGTCGCTACTCTCGGTTTACTA
CCACCGATTGCCCTGGGTCTGATGGAGCCAGTCTCGTAATACCTGCCCAGAATTTTACTCACACATC
CGTTGGACACCTGCATAGGGGAAGTGGACAGAAAACcacattattaataatttcaagacaaaaataaaattgtttaagtaT
GCATTAAACAATGACAAGCTTACGTTTTGATTGTCCAGCACTTGGACTTTTGCATCTGCATGGGTCTA
TAACACAAAAATATACCTTCAATGGTATGAGAACTTACT
>NC_000011.10:31804066-31804348
CTGAGCTTCTTAGCAATAAATAAGCTCCAAATATAGAAGAGGGGGGAAAATATGATGAGCCTCTCTGa
catttgtctttaaaataaaactagctGCACGTCAAGTTTGAGCTTAATTTCTGGAAATAGGCGGAAGTCGCTCCGG
ATCATGCATGGAAGGCTAATTGAAAAGATCAGTCGGGGCGCTTGTGGCAGCCTTGTCACTTTGTGAC
AGTGCTCCGCTCCGGGAAATTGCATCGTCACGACAAACGGGACCGTGATAAAACGACCCTTTCCGT
CCTTA
>NC_000011.10:31811060-31811374
TGGAGAGTGAGAGATAAAGAGTGTGGGTGAGGGAAGTGGCTGCAGCCAGCACACCTATGCTGATTG
GTGATGGCTCAAGTGTGTTAATGTGTGTGTGCCGGCGCCCGGCCTCGCCTCCACCGCTCCTCACTG
GCCCATTAGCGAAGCCTGACCTCTGTCATCATCCTCCAGCAAAACACTTCCTCCTGCGCCTGAACCA
GAGCGGGAAATGAGGCCGAGCCACGGTTCCCTTTTCAAACCCACTAATCACTCCGCAACATGCAAAT
GCACCGCTCGCTCTCACACAAAATATTGCTTTATGCAAAGCAGCGCCG

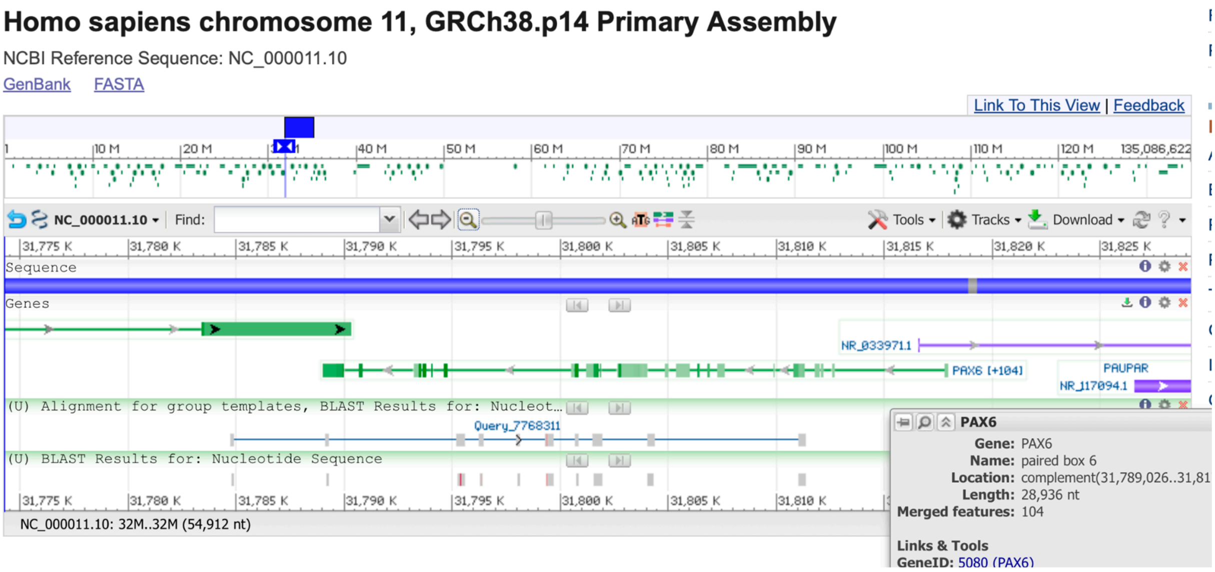

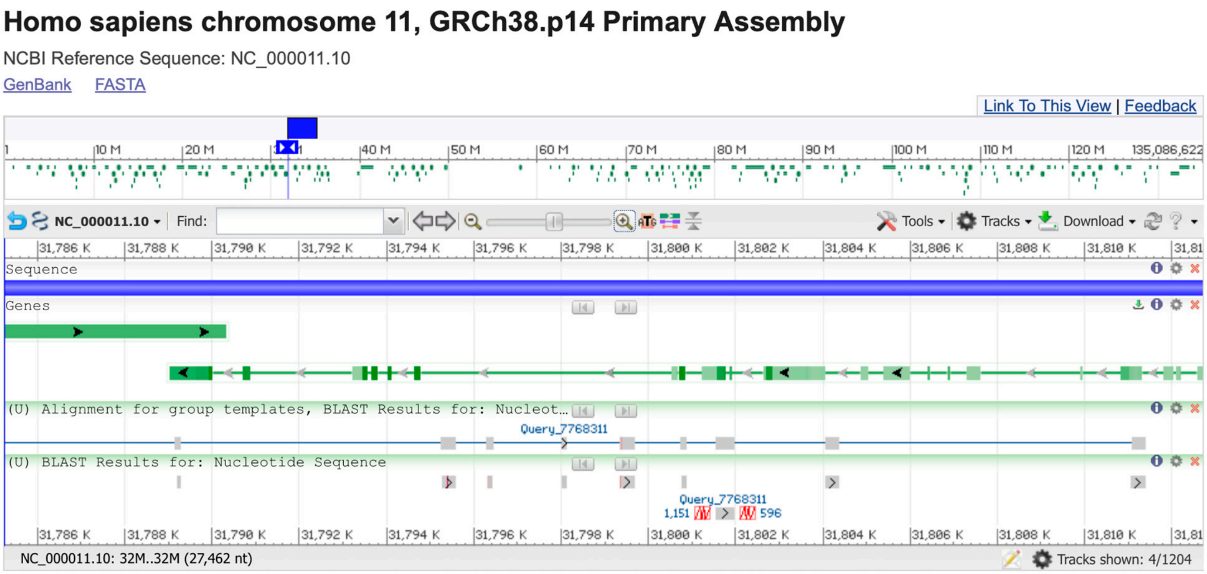

chr12 CLUSTER 41 positions on BED file: 8718 - 8728
>NC_000012.12:53972305-53972430
CATTGATTGACAATAAAGGCAGGCATGAAATATTCACCCAGGGCCACTCTCTCATACTAAATATTTAA
CACAACATTAGAGAAGGTTTTTCCCAGACTTCGCCAGGCACCGGCTGGGCGGCTGGC
>NC_000012.12:53972971-53973090
AAAACGAATACAAATCTGCAATTGATTTTCATAATGTTTCTGCGGTGTTTGCAAACCAATCGCCTGAA
CGTCCCCGATCTTACCTAAGAGAGAACCCCTCCTACGTCTGCGAAGTGCTC
>NC_000012.12:53974019-53974149
TCGTGTTTTGGGTCAGTCCGATTTTATGTGGAGTTTTATAAGCATTCAAAGGATTTTATTATAACCTAC
AAAGCCCTCTCTCCCAACGACTCGACTTTTTACGatggagaaggggtggggagggagggaa
>NC_000012.12:53985198-53985315

aaaaaaaagatgtcagctCCTCCGCTGTAGTATTGCTCCTTAAAAACCCCTCTCTCTGAAAATGACATGCCCT
CGCAATGTAACTCCGAACTCGTACGCGGAGCCCTTGGCTGCGCC
>NC_000012.12:53989133-53989451
AGGGATACTTAtgtaaataatgttatttttaacagaGGAGATAAAGGCAGAAAACACCACAGGAAATTGGCTGACA
GCAAAGAGCGGAAGGAAGAAGAGGTGCCCCTATACTAAACACCAGACGCTGGAATTGGAGAAAGAA
TTTCTGTTCAATATGTATTTGACGCGAGAGCGCCGCCTGGAGATTAGCAAGACCATTAACCTTACAGA
CAGACAAGTCAAAATCTGGTTTCAAAATCGCAGAATGAAACTCAAGAAAATGAACCGAGAGAATCGG
ATCCGGGAACTGACCtccaattttaatttcacctgaGAGCG
>NC_000012.12:53990390-53990638
CGTCTCTTGGCGGGTTTAATGTAAGTGAGAGACCATAACGTTGATTTAAATATTATCCAGGTGACCAC
AATAAGTCAAGGTCATAAAACAGTAATGTCAGGACGGTCTTGGTGCGCGCGAGAGGTGGATTTTATG
ATCTGCAAATATAATGTGGTGCTGCAGTAAAAGATGCATTTAAAGGTGACTGGAGgagggaaagaggcaga
gagcAAGCTGCAATCTTGAGGAGCAGACGGATCTGATTGCCT
>NC_000012.12:53991742-53991858
GCTCAGCTGATCTGTGGCTTAGGTAGTTTCATGTTGTTGGGATTGAGTTTTGAACTCGGCAACAAGA
AACTGCCTGAGTTACATCAGTCGGTTTTCGTCGAGGGCCCCAACCCACC
>NC_000012.12:53993889-53993995
GCAGCCCAACTCCAGAAATCGCAATAAAAGTTAAAACCTCccctactgttttttttttaattatgatatGGGAAAGAGGA
GCAGGATTTATAGAGCTGAACTCTCA
>NC_000012.12:53998962-53999089
CCGTTCCAAAAACAGCCtacaattttatttaatgaaagatACATTTATGAACAATCAAATGATCctcataaaattttattgag
gGACGTAAAAACAAGCTACTTGCCCACGACCGAGGTCGCTCTT
>NC_000012.12:54000000-54000280
aaaaaaagccCCTGCGCATTGATCCGCGCCGTATTTTTGGGTAAATACGATCACGTGGGGGCCGGGGA
GCCAATGAGCTGTGGGGAAAAGGCTGGAAAAATAATTACCTGACTTGATTGTTCTGTGAGCAGATAA
AAAGTACATATACAGTTCATACAATAATCTTATGTATGTAAAACCCCGTTACGATGTCGGCGACGGGG
CCCATCAGTAACTATTACGTGGACTCGCTCATCTCTCACGACAATGAAGACCTCCTAGCGTCCAGGT
TTCCGGCCAC
>NC_000012.12:54000416-54000562
TATCACCCGTAcggcccccagccccacctcgGCGCCGACACGCGCTACATGCGGACTTGGCTCGAGCCGCT
GTCCGGCGCCGTCTCCTTCCCCAGCTTCCCGGCCGGGGGCCGTCACTACGCCCTCAAGCCGGACG
CCTACCCCGG

Homo sapiens chromosome 12, GRCh38.p14 Primary Assembly

NCBI Reference Sequence: NC_000012.12

GenBank FASTA

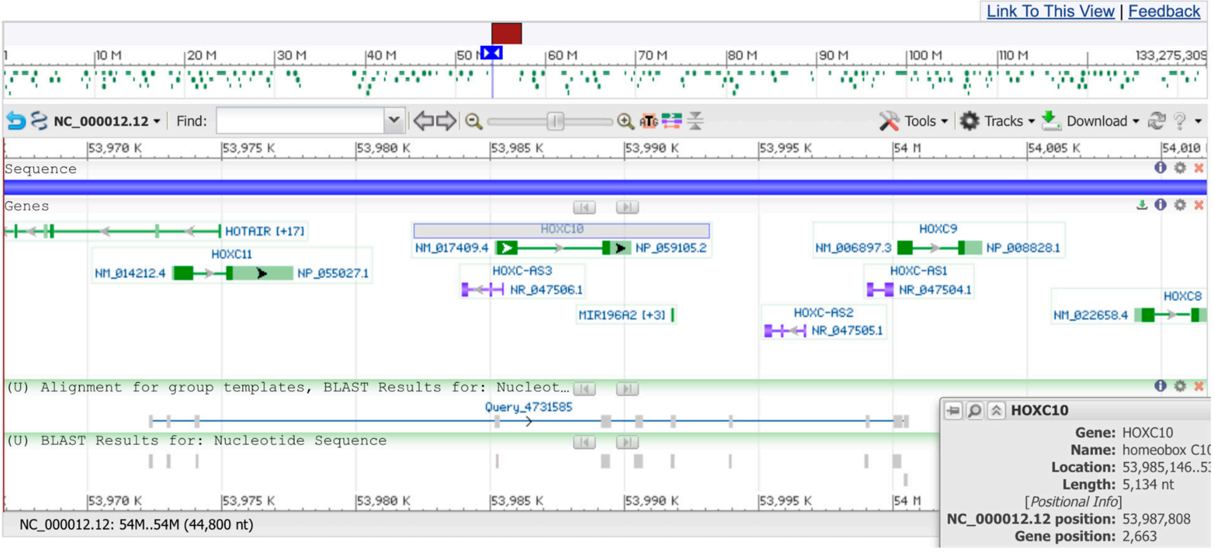

Homo sapiens chromosome 12, GRCh38.p14 Primary Assembly

NCBI Reference Sequence: NC_000012.12

GenBank FASTA

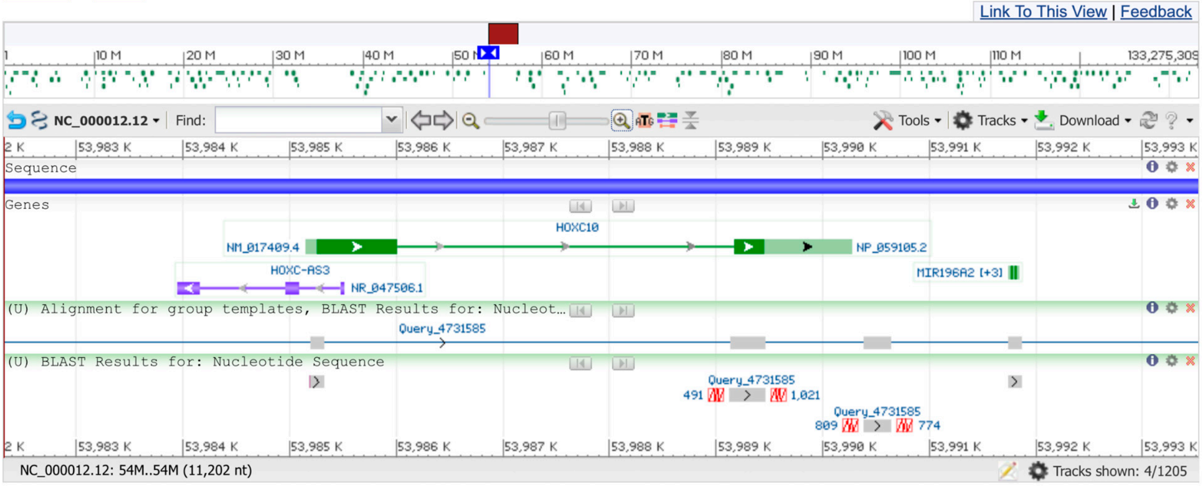

chr13 CLUSTER 42 positions on BED file: 9111 - 9123

>NC_000013.11:71751172-71751316

TCACTGCAAGAACTGTTTGCATATTCAGTTATTGTTTCTGCTGCGTCAGTCATCTGTTGAAGTTCACTC
TAATGTGGCCCTGATCAATTCTGCCAGCTGTGCTTAAGTGCTGTTTACTATAATAACATCACACTGCA
TTCTTAT

>NC_000013.11:71753291-71753411

CTGCAATATAAGAAAAAAGGAGCTGATATTAGTCTACACAGTGTATTACAGATGAGGTACATTTCTTC

TTACTGCACTCCTGCTGAACCAAAGCAATAATTCACAGATTCCTTAAAAGGT
>NC_000013.11:71758934-71759111
tttttttttcctgtggtccTGTTTAAATTCTCACCTTGCTCTGCCCTTGTCATCAAGTCGTTCAAGCACAAAGCAG
GGAGCATGGGGAGTGTTTACTCATTTCCCAAGTCCATTATCTTACACATTAATCACTGGTCTGGAACT
TTTGGACAATGCTGAATCTgttacctttttt
>NC_000013.11:71759594-71759731
AAAAAAGCAGGACAGCTGGAGGCTCTTGAACTATAGTTGTCAAAAGATTTGTTTGTGAAACATGCATT
TTATTGCCTTGTATGCATGTTATAATGAGGCATGGACAACTGCAACATTACACTTAATGACTGCATTTC
>NC_000013.11:71759805-71759949
aaaaaaaaaatctgaaattcacatAAATGAGCAATGTCAATAGTTTAGAAAGAGTTTACTTAGAGAACAAAGGGC
CCTTATGAGGCACTAgtgagaagaaatggggaaaaaatgagtCCTCACAATGTAAGCCTTTTCTTAAAG
>NC_000013.11:71760102-71760236
GGCGGCCCCAGGTGCCAATTTACTCCTCTTagttcttttcattctgtttgctGATTCTGACAACTGGAGTTCTCCTC
TCAGTCCAAGGGACTTTTCAATTGGATTTGCTCGTGGTGACAAAATTGTTGCTTTGC
>NC_000013.11:71760256-71760416
ATTGAGCGTCTATCATGCTGCCATGGGAAAGCGAGAGGCGAATGATTCTCAGTGGCTTCTGATTTCC
AGTATTTGTTCAGCATTAGGCCCACTTATCTCCAGAAGAAAAAGCAACATGTAGAAAGAAAGTGACAG
GCAGAAACACACTCACTTGCTCGTT
>NC_000013.11:71760472-71760607
GATCAGCCTCACTCAATTATCACAGTTAATCCTTATAGctctataaagtaaatatttcattACAATTTCAAATAAACA
GTCACCCAATCTAATGAAGCAAGAACTCAAAGTGGCAATGTCATAGAACTCGTTACC
>NC_000013.11:71767642-71767837
CAAAAAGCTCTTTATAGCAAAAAGCATGGTTGTTATTTATGGCTCAGATGCAAAGAATGAGTTTGGGG
AATTTATCAGAGATGAAGGTATGAAATGTTTTAGCTTGTTCTTCCCTCTCTAATCCATAAACCATTTTC
TCAGCCACCCAAGTGAATTCTAATGGaacagaaatgatgaaataatattttcctagCC
>NC_000013.11:71770020-71770162
CAGTGCATTAGAATACTTTTAGTacagctttaaaattttaaatcaattcTGCCATTCACTCCATATTATTTATCTTCAA
TTTCTCCATATTTTAAATGTCACTGTGTCATATTaagtaataaaaagtttaaaagattaTAAC
>NC_000013.11:71770934-71771089
CTAGGACTTACTAATAGGATTGCTAGCAATTATTATACTGAAATGAAATCAGCACAAACTATTAGATCT
GTTtaaacaagttaaaaataaaccttGATTAAATGTTCTTTTATAAGAAGCTGCTCATTAGGGCTACATGGCCTT
CAGAAAAATTG
>NC_000013.11:71772316-71772737
GTGTGTATtttgcacaatttttaaaagtttaacctCTGTAGATCAATGTGCTTCCTTAAAAGACTTTAATAAATTAAAG
TGCAGAGAGCTTTTTTTCACCAGGCAGAAAAAGCTGATGAGACAGGTTTAGTTTAATCTCAGGCAATA
GAAGTCATTAACCATTCTCTCAGGGATTAAAATATAGCTTGcccttttcttctctcacttATCATTAGCATCACTTT
AATCTCCTAGCGTTTCACACTGCTATTTTAATCATTACTTACTGCCTTTTGACAAGTGCAGAACATGCT
TTTTCTCATTGAGGCAATATCCTGGTTTTAGCTGACAAATGTAGGACATAATGTAAAAATATCAAAGTG
ACATCTCTTAAACACACCTTCATCATAAAAACTCTAAAGTTACAATATTCCTTTGTCTA
>NC_000013.11:71773641-71773830
CACTATATGTTTCGACAATAAAATCCCTTCTTAAGCTTCACAAAGCAGAACAATTCATTATTTGATCTC
TTTCCAGGTTCAAAGTCTGTCTTATTACAGTACTTCAAAGAAATATGTAATCAAAGACAAACAGCTGTG
AAAACAatcttttattactttattatcaTTGAGAATATATCTTTGGAGTAA

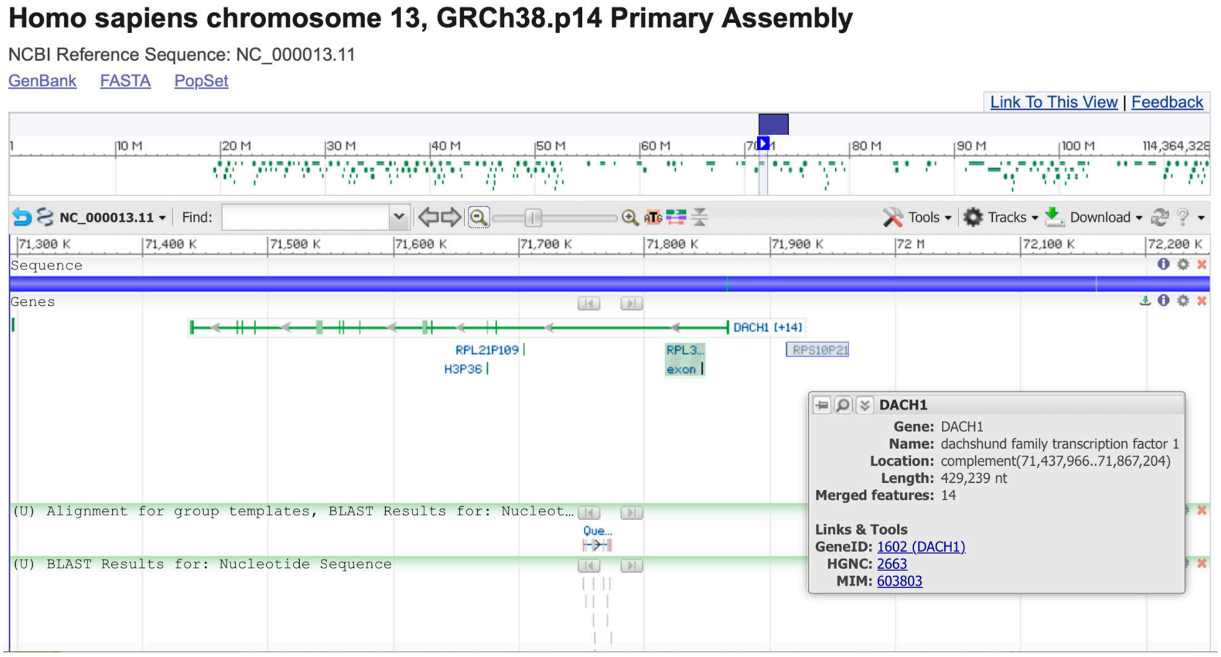

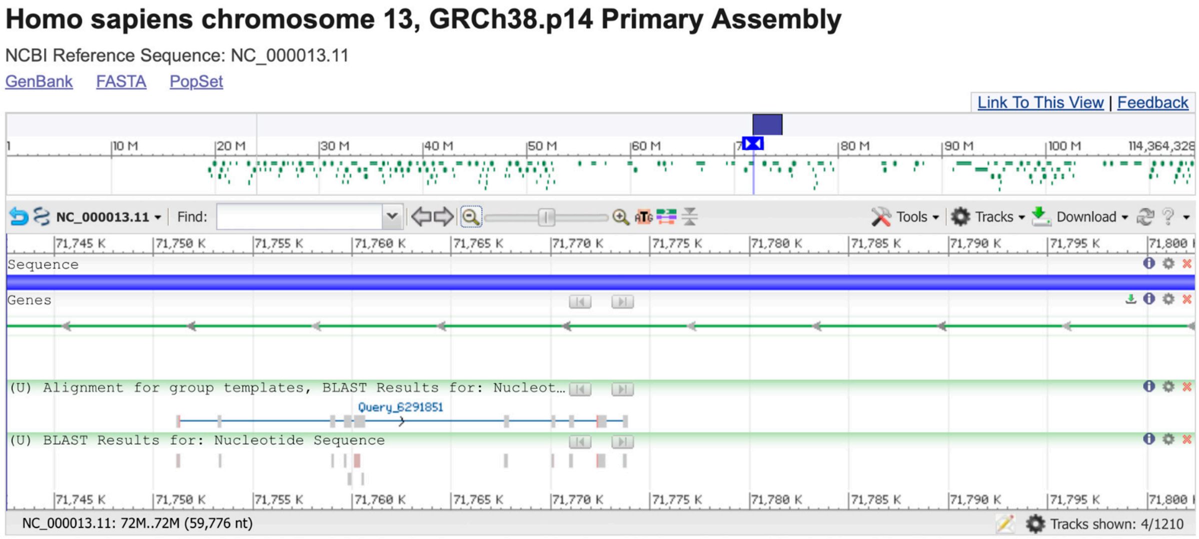

chr14 CLUSTER 43 positions on BED file: 9512 - 9522
>NC_000014.9:33619802-33620021
AAGATATCCATTTATCTCATATATGTCTTGTTGAGGCAAAGCTGTCAGAAGAAATCTTATCACATGGTG
CCCCATTTGTTTTCTGTGGTGATTTATGATGGTTTTGAAAAGGTCTTGTGATCCCATAGGCTGCCACG
ATTCTCTGACTTTTCTCTGTCAATGGATCAGATTTTTGACATTTTCTGAGagcattcagaaaatatttgataaatca
GCTTG

>NC_000014.9:33630402-33630517
AGTTTCAGTTCTTAGTATTCCTTAATTATCCCACTGAGATGTTTGAGAAGCAACTAGTAGTAAATGAAC
TTGGCAAATCAGCTATTACCCAAGTaatgaaatacaaatacatatg
>NC_000014.9:33630806-33630932
AAGGATGCATATCAACTTCACATCTAACCTCAAACAAAGCAGAGATCAGCTCTACTGAGAAAATTGAT
TTTATTCAACAGTAAGGCAAGGGAAATTAGTAAATTCAATATTTGTAGAAAGAAGATA
>NC_000014.9:33631470-33631704
TACCAGTTTAAACAGTCTGCTGCAAGAAATATTGACAGGTCAAGTACAGCTGGGAAGAGCACGTGTC
CCAGTGGCCTTTACACTGACAGTTTCTCCCACATTAAAGAGTTAACTAGCAGCCAACCAGCTCGAAG
TGGGAGCTAATGAATGAAATGGAGATTGATATAGACTCTCTTTGGCGGTGCATCGATCTGTATCTTGT
GAACTCAGCTTAGGAATTCTGCAGGCAGCATT
>NC_000014.9:33638749-33638882
GCAAGCATTTCAAAGCAATTATTTACAGTGGTAATGGATGTGGCAAATGTGTGGATTTTGTTGGGATT
TCTTCTGACAGCCATGAacacagatatattttaaagtaatgtaACTGTCCTAAATCTCGAGCAGG
>NC_000014.9:33641910-33642216
GCAGAAAGTTTTTTACCCCAAgtaaatttctcatttgtttttagAGTTTGTGTTCAATAATTCATCGATTCCCAACAG
CAGGGAAGAGTGAGAGATTTATGGTGTATGATTGTGTCATGTCTTGTTTTATCATGTCGATAGATAGA
AAATTGACCACAACTACAAAAGCCTATTTATAATTCTATTTGATCACCATGGGAACTCATCAAAACAGA
GGGCAGCGCTGAAGTTTCATTAACAGATCATCTCGGATCTCCTGCTGTGtcctgtcttcattttcttttcacatcATC
CTTTTGCTTAGA
>NC_000014.9:33642248-33642374
GTAATGGCACATTTACCATTCCCCACCATCCCCCAGATTAATATtaaactgttaaaagaaaatgagagtttATTGAT
TGTTTTAAGCCAGGCATGTTCTAGCAGCGAGCCGCTATTCAGATTCAGC
>NC_000014.9:33642433-33642537
AGAAGGGAGAGAGAACAATGGCCCAGGAATGTTTAATCTTTCTTATGGCACTAATCGTTACTTTTGCA
ATTTGTGATTGTTTATAGATCATTTCTTTTAAGTCT
>NC_000014.9:33642960-33643090
AGTGTCCATGGATCAGAGAAATGGCTTTTATCTATAAGCCTTGTCATCTCCTCTCGCAATAATCTTCAT
ATAAGTAATCATCTCATCATATATAGATCAATCAGCCTTGTTAATGCTCTGATCTCAGCTC
>NC_000014.9:33643122-33643319
ctattttctttgtaatttgagGTTTTGATTTAGTGGTCACATTAGTCTTGTCAGTTTGTAGCAGCATACCATGATGTT
ATTCATAACCAGTGATAGTATTATCTGCTATAAAGTTGCACTGTAAATCAGGAATAGTTGTCAACTTAA
ATATTTATGCATGTCTAGGGAAAGTAATTGTTAGGCAGACACCCTTTGCT
>NC_000014.9:33643516-33643787
TAAGCTCTCGGAGTTTGCCATTTGAGAGCTCCTTTGTGAGTTGGCTTTTTGGTCTTTGCTGTCACTGG
TTCTGAAGGTTGAAGAGAAGAGCAGTGTCAGAAAGCTGATTGGTAGCACAGAATTTTCTGAGACAAT
CTGAATCACATATTCAGCTACTTTGCCACAGTGGATTAAGATGATCTAAAGGGGCTTTGTGAGCTCGA
TAAAAATCAGGCAGGAGAGTGTATGCTGGGAGAAATTCTACCCTTTTTGTCCTTCCAAGTTAATTGTT

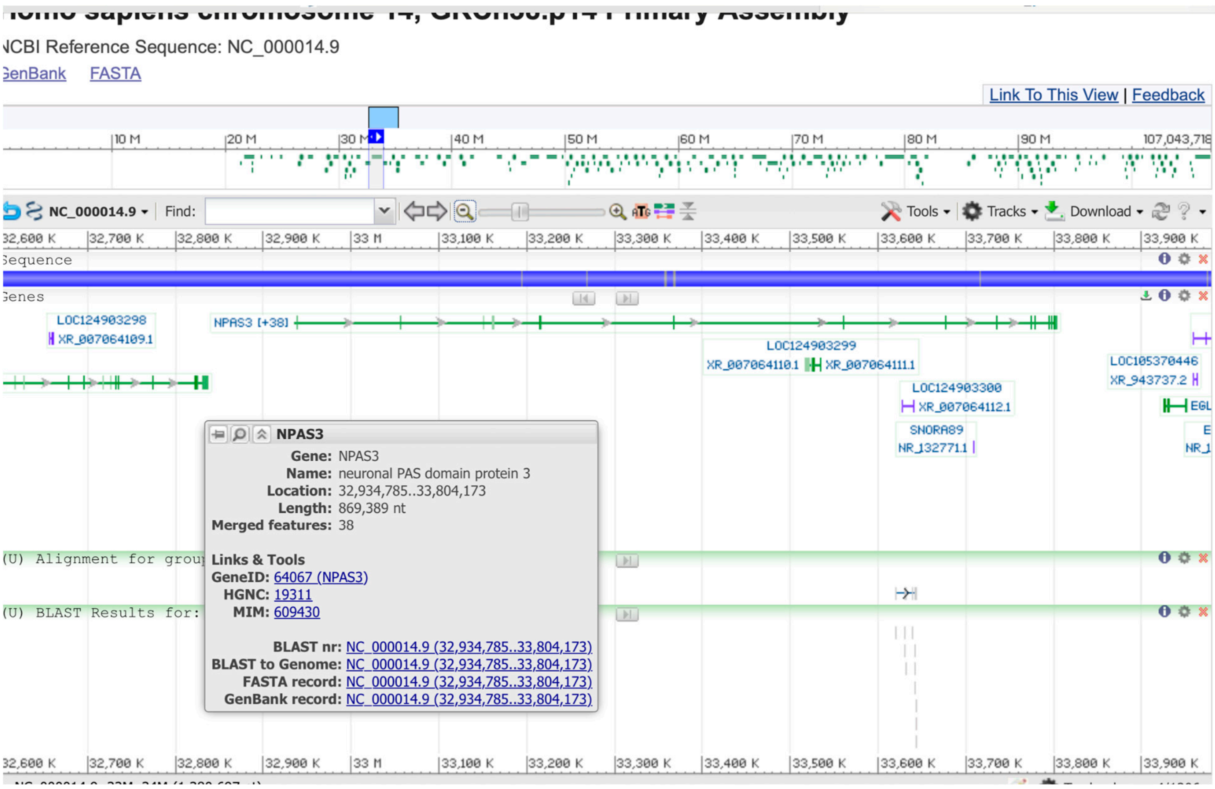

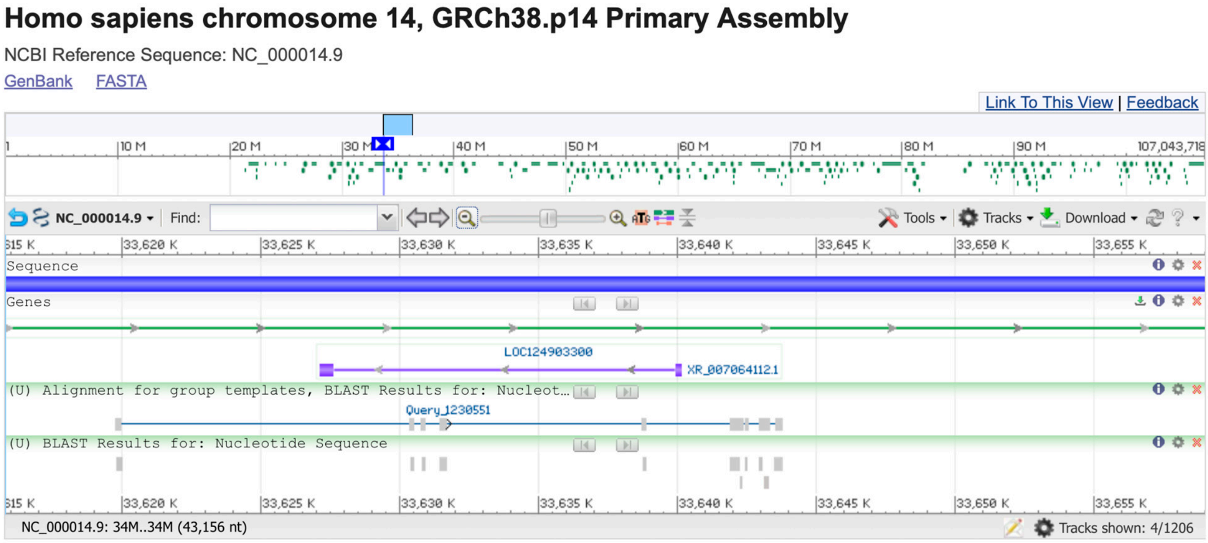

chr15 CLUSTER 44 positions on BED file: 9945 - 9959

>NC_000015.10:36884650-36884791

aaaaaagagaaacaaatctgCCAGTCATTTTAAAGGAGACAGGCACTATTGAAGTGTGAAGAAATCGGCTCTAATTCAAAAAGGCAATCGGGCAGAAAAAGCCCTCAACATTACCCTGCTAGTTATTAATCAGAAAGGGCT

>NC_000015.10:36887161-36887397
TGTTATTTCTGTATCGGTTTTTGGTATTATAGAGGAGTAAAGAAGTTTTTGTGTGATGGTTTTTCATTG
ATCAGTCACTCTGAGCTAATGTAATTATCCTCATCAATAGGAGGCTGCAGAGAGAATCATTATGTGGG
TGACATTGATAAATGATCGCCCAGGAACGGCCCTCTCGCGGGCAACCCCCACGTCTgaccctcccacaca
cacaatGTAGTCATAGAAACACCTAGAGA
>NC_000015.10:36889180-36889356
aaagtgttttatattaCTCTTGTTTTGGAGAACATAAGCAATTTTATTCAGACTACAGTATTTCCTTTAACACTA
TATGGTTGTCAAAACACCATTCCCAGTTTCTTTGCTTTTGACAGCTGAGTCTTTCTTAAATATACCCAA
ATCGGAACTCTTTACACTAAATAGTAAACCC
>NC_000015.10:36891075-36891369
ttttttttaacataacgTCACAGTCCTCATACaagtattttaatgtaaatttgaCAAAGCTTAAAGGTAACAGCATTTTCTTC
TAGTGAGGAACACGTGCTGAGAAAAGAAGAATTCATGGACATACAATACCAATTCCACAGCAGATCT
GATACTagcaaaaacattcttttttttcaattgaggtAAACACATAGAATATCTAACATGAAACAATTAATAGACCGAA
CTCTGTACGAAGTTTGTTACAGTATTCTCTTGCTCCTTTTTATCCCCCAAGCTTTGAGTTTC
>NC_000015.10:36891834-36891972
tttttttctcttctaaaactaTTTGAGGCAACATAACGGCGTGATCAACAGAAATGTACAAGTTTAACTTAGTTCCT
ATATTTATACTACAGTAGTTATAACTCTCGGAGTCTTTTTCCAGAGGATCTTTACATGGA
>NC_000015.10:36892031-36892134
GTTGCTTGATGAAAAatgacaaaagtaaaaaataatcacAGCTGTCTGGAATTTCATATTAAGTGTCAACATCTG
GTCAAAGTTCAGAAAGTCTTAAAATAGT
>NC_000015.10:36892540-36892735
aaaaaaaaaaacaacagacactgcaAATCTTATACCTAAATTTAGCTGCAGATTCTTGCCAATGTTTGCAGACA
TTCAAATTGTAGTTTGCATTTAATTTCATCGCACAGCAGTATAATGCAACACAACAGAACTAAATATTT
AATGCAACTAAATGTCATAAAGTggaactgtttttcaaaatggtaTTCAGTA
>NC_000015.10:36893384-36893757
GCTGCAGTCATTTTGTTAAGTAGATCCGCAGTCCATTATTAACAGAAGTGGCAAAACCATCTAAAGAC
AATAACACAGCCTGCATAGTCCCAGCTCTCTGTTTATTCTACCACAGCAGCCTGCTCAATGGATGATG
ATAATTAAGTACCAAATATACCATATTGTGTGGCTGCATAATCAAATCAAGAGAGGATAATATTCTTCT
GTATTAAGCTATTAATGTAATTGGTCATGAAGCATAAAATATGTTATGTAATTAAATAGGCTAGAAATT
ATATGGCCTATATTAAGAACTATCCAAGCTGAGCAAATGTTTTCTCTATAGCTGTTTTAGGGAACATTG
CTTTCTACATTTTTTGACTGCATtagtaaa
>NC_000015.10:36893969-36894348
ctctgaattttttcagatgTCAAAAACACTTGAAGCTTTAAAGTCTAATGGGTTTCTTAATCCAACTGCAGCCATT
CAGCCTCAGTGAAAAATCCATTCCTTCATATTCAGTTGCTCCTAAATGTCTTTCCCTTTAAATTTATTG
ACTGACTTTCCTGTCTTTCTGTCTCATGTCGGAAGTCAGTAGGCACatgtaaaaaataatcaaaacacaCCGAC
TCCCTAGTGAAGAATCAATGGTGTCACTGCTGTCATATATCTAGACTAGGCATAATGAACTGCAGCAT
TTCATCTTTGCATATAAGATAATTTGGGCATCAATCGTATTCTGACAGATTTATGTCACTCAAAGGACA
gttctacttttcctttttttt
>NC_000015.10:36894709-36894832
TACTTACTTCCCCCTTGCTTTGCGATTGCTTTACATGATGAAGGTTACATGTAGTGCCATTGCCCATC
CATGCCCATATTCATGCCCATTCCACTCATAGGTCCTAGAAAGGAGATAAAATCC
>NC_000015.10:36895224-36895324
GCTGCTCCTTGGCTCACTGAAGGATCAAGAAGAAAACCTGATTGTGGTCATTCGAGGacacagaggagaa
agaagaaaagatatacCAAACAATCAGCAA
>NC_000015.10:36897096-36897247
TCGGCTTTCACAAGGTATTGTTAGGTCAGCAAAGCCATCAATTAGGCTGTCTGAAGTTTTATCACCAA
CACAGCAGTAATAAGTGCTGGCCAGAAAGACTTCTGCTCTTCGGCAACTCCTGCAGTTTAAGCCTGT
AAAGTTAGGGGTCATT
>NC_000015.10:36899768-36899995

ATATTAAATGGGccttataaaataacattttgatgaGTTAAGTGTAAATGAATCAGACACATATGGATTAAGTTATC
CTTGAATTTCACACGGGTTTAAAAAATAAGGGGAAAAGAGGGTCTTGGCAAAAAACATTTTGGACTTT
GCCAAAcccaaaacatttgaaaaattgctGAAGACATCCAACTAACCAAAATGCAGCAAGCTGCACCCAACTTTA
ATGAAAA
>NC_000015.10:36903987-36904201
GAAGAAGATGTAGGCTGATTCTCCCATACAGTCTAGCAGGCCAGCTGTTAGCTGCCTGTCAAGCCCT
CAAGTGGACAAGTAGTCCAGGAGCAAAAATGTCACTCCTTTGATCTGGCAAGGAGCAGCTGCTGATT
TATAGAGTAGGACTTTGTTCTTCCgttctattttactttctttcctaAGGCAGAACCACAaaatccaggagaaaaaaaaa
>NC_000015.10:36904325-36904445
catttTTTGCAATGCAGTAGTTATTGTTTAATTCAGCTTCTGTAAATGGAATGGACTGTGGCCAGCAAGC
CTGGGGCATAAACACACCTGTATGAAAGAAGCCTTATTGTAGCAACCATC

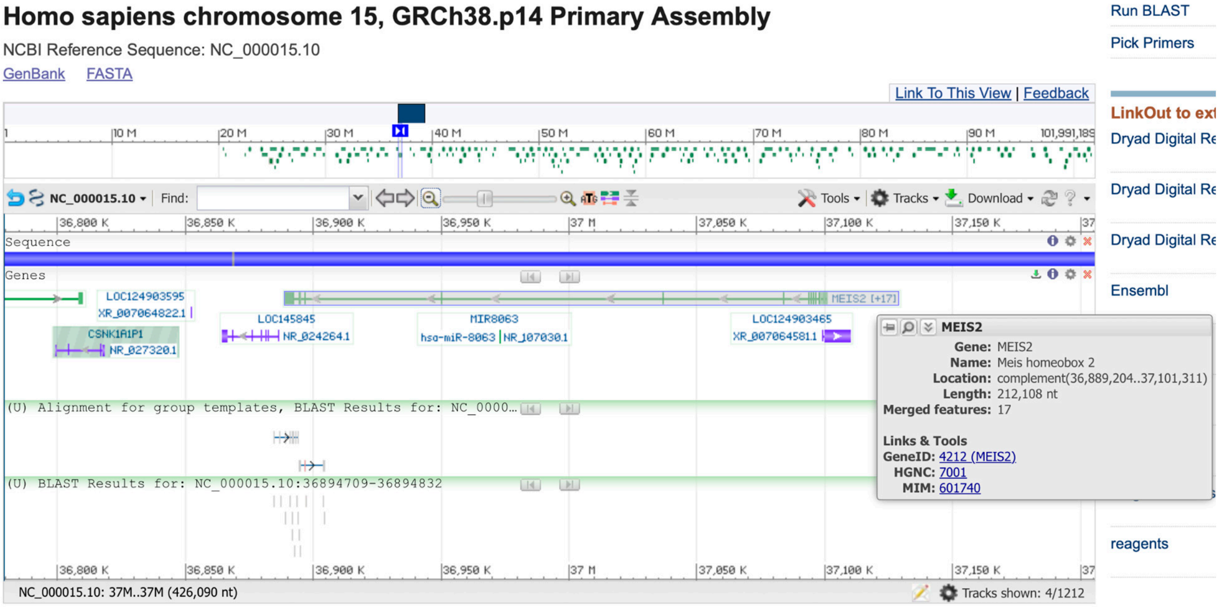

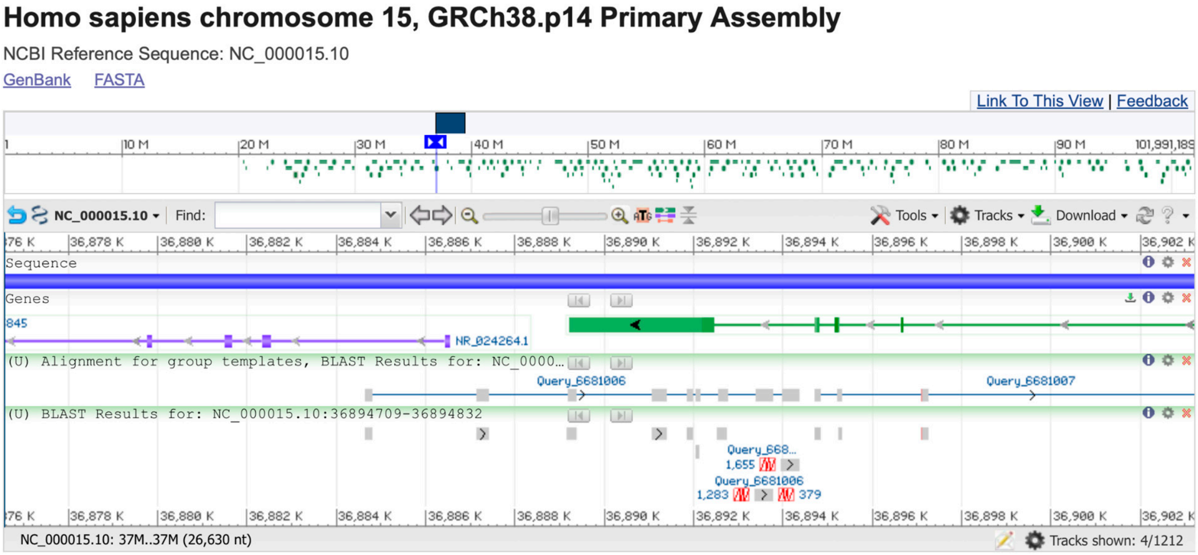

chr15 CLUSTER 45 positions on BED file: 9968 - 9978
>NC_000015.10:37035234-37035546
TCACTCGACTtccaaattaaaaacattttacaggTTAGTAAATCTCCAGCTGAGGTGATGTAGCAAAGAACAGCT
GTGAAGTAATTTTGATAATGATGAGAATAATAACCACATGGCAAGGTTCAGGGCTTTGCTGTTGCAGC
TTCAGTGCTCCTGAATAGATAAAAGTGATTAAATACTCCTGATTATCCCTGACCAATATGTAATGGATT
TACAGCCCTTTCAATTAGTCAGTATTTGCTTAGGACTCATTATTTTGTCTTTGGTTAAGTAATCAGTCC
AGAGACCGCATGTTCTGTCTAATTCCTCTGA
>NC_000015.10:37037047-37037198
TGAAAGAAATCCATTAAGCGAGTATATTCTCTTTCATTAAAGTATAATTGGAGACACAAATatgtaaaagac
aaaattaaaccAGGCCTCTTCAaatgttaatttgtgtgtctCACACTTGCCCATATCATTAATTCAATTATAACTGTG
>NC_000015.10:37037612-37037945
TGCTTTTCTCTGGCATTAATTGTGCATTAGCAAAAATCATTTACTTTTAACAGtcatagttatttttcttgccCTCT
GGCAAAAATGCCTTGAAAGCCTGCCTGGAGGGCATCTAAATTATGTATCTGAAAAACTCTTCTGGCA
AGGCATTGCAGGACCCCTACTTTCTTAAAATTCATACGAGCACGTCTTCCTGTTTTTGGGAAGGCATC
AATCAAGAGCAGCAATATATGCCATATCCCTACATTAATATTTGAactaataactttaaaaaaaggaaagaaacaag
atcCGACTTGTGGCATaatcaaatgcaaaataaatgaagaata
>NC_000015.10:37038099-37038232
CTGAGATGGGGGTTTTTGTAATTTAAGAATAGACATTCATTAGCAGTAAATGCCATAAATCCCATGCT
AAGTAAAAACCTTGTCCAAGAAAGCCTAAAACAATAAACTCTGTGCTCAATGTAGCCTGAGCTGG
>NC_000015.10:37038499-37038633
GCGATACCCTCCATCACATGGAAGAGATGACCCTCACTATTTACAGACTGACAGGCCACTTCATTAC
AACCACCCTGCCCCAAGGCAAAGCAGCTCCCTTCCATCTGCCCAAACACTAGAGCATGACCCCGAA
G
>NC_000015.10:37046473-37046592
tcagactgcAGCTTGGATGAAAACCACTTCACCCACTGGTTGgaaaaataaagtgtaaaaacAGACAGATGGACA
CATATGTAAATGTGGCTGTGTTTTAGGCTTTAGCTGGCAATGTG
>NC_000015.10:37046595-37046710
GTCGTAGTATAGTTTTGCACGCCTGTATTTTCCATATCATGTTGAATCCTGTTAACTGTTGAACAGAAA
CATCCTCATACTGGTCCTTGACCAAATCAGTCCCCTCAATTTACCC
>NC_000015.10:37047758-37047996
CAATTTCACTTAGCTATGTTTCAGTACAAATTGTGAAAATTATGCAAATAACAATTTTGGCACCTTTGC
TAGAggaaaacattattaaattatGCTCTAAGTATTTTGTCTGCTTTAGTAATTACTCTCATGTAACTGTAAATAT
AAATAGCTGTtattctgtttaaatttttaatcatgCAAAATATTGTCACACAAAATGTCCTTCTGTTGCAGTCTaactggg
attttcttttt
>NC_000015.10:37055935-37056475
CATACCATAATTGACATAAAATATCTGTCCCATCAGTGAATATCTTTATTAGCATCAAAATCACTCACA
ACTAGTGTTCCTCAATGTTATTAAACATAACTTGTTCTAGGTGTTGCTTTACTAATTAGTCAGGATGAG
CCCGAGGAAGAAAATCTGGGTCATGTTAAATTAATTGGAATGAGCATGAAAATTAGCAGAGCAAACT
GCATCAATTTTCTATAGAGAGTCTTTCTTCATGTTGAGAATAAAAATGGATATTATGAGGCATTAACCA
AACTGCCTTCTGTGACTGCCAAGAGGTGTGTTACAGTAGCCCAAAATATCATGCTCGCAGATTTTCA
GAAGTTAATGAGACATTTCCAGGAATGGTAAATACATGGTGTTAAAATGGGTGTGCCTCTGATGGGA
AAATGCCAAGTCATTTCATGTTTATTCAGGCATAACAAGCTCTTCAAAATGAGCATAATTTAAGAGGTG
AATAGTGCAAAGAGGGCTAACATTCAACTAATTTTCCttcagaaagaaaagattaaattgaac
>NC_000015.10:37057103-37057251
TCACGGCACCTAGCCTGTAACTGGAAGATTACCGAATTGATGGATCCTTAGTTTAAAATGCAGCTGTT
TTATTCTGCTTTAGTAATTAATTCTTTTTCCAATACTCTATTGATTAAGCGTAGACCCTGATAAAGTTCA
AACTGAGAAG
>NC_000015.10:37057279-37057396

GGAGAGTCAATGAAAACTTCCTACAATTCCACCTGCCTCCTCAGAGTGCTGATCCAATGAACAGAaca
gtttttttctcataaattagtCAGGCCATGTGGAGCTGTATTAGCTGAT

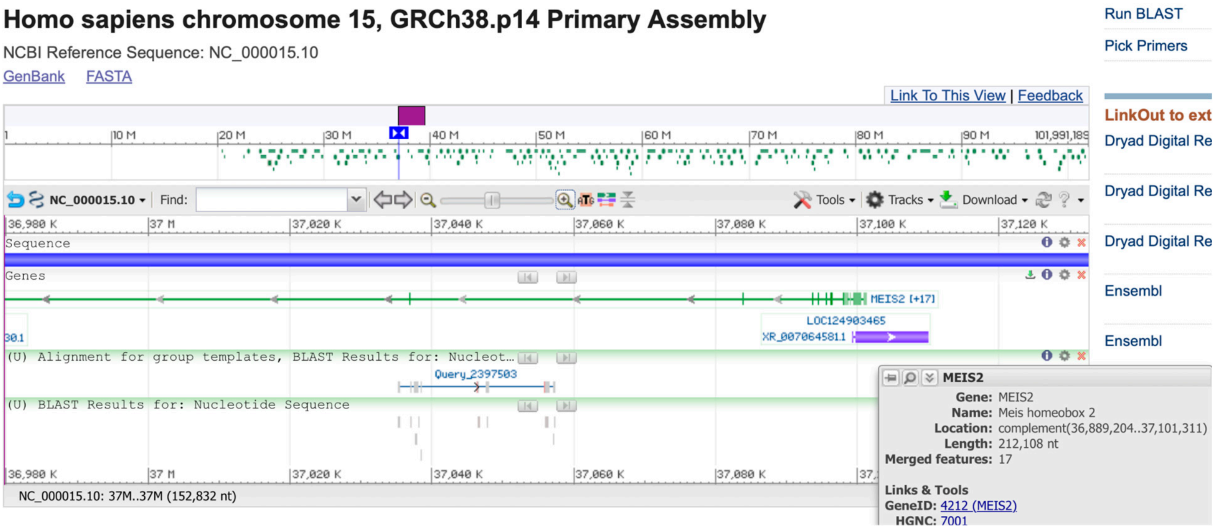

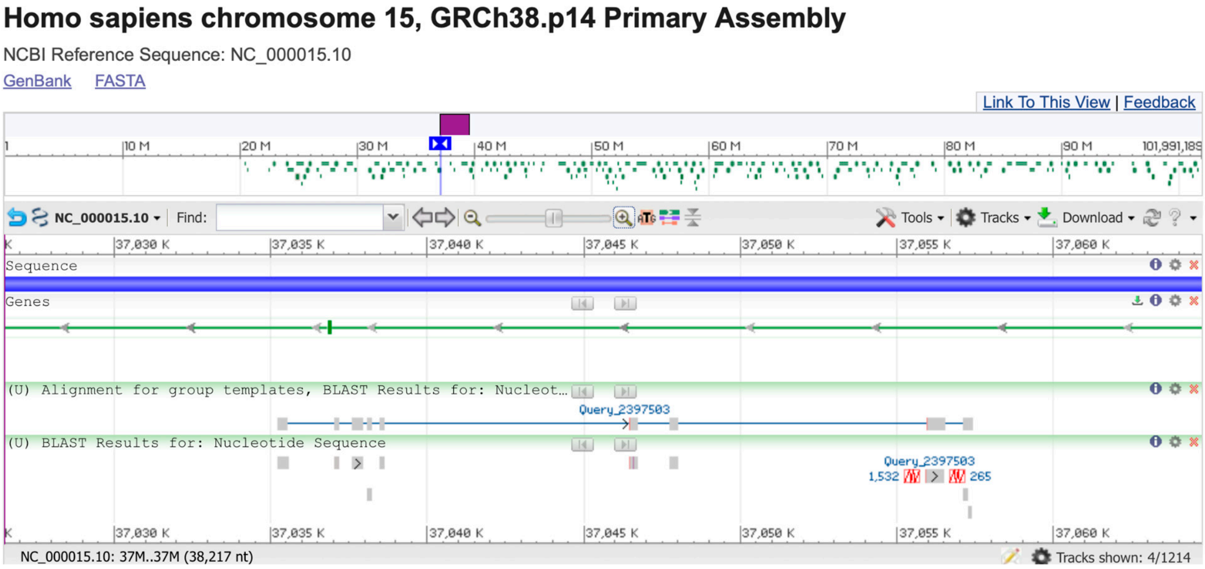

chr15 CLUSTER 46 positions on BED file: 9979 - 9991
>NC_000015.10:37077540-37077712
ttttcccCATGTCAATATGAATATTAGACTGACCCAATTAATATGAAATGCTTCTATGGTGCTGGAACAGA
CAGTGCTACAGAAAGAGCCATTTGCACAACCTGTTCATGATTATGGGCCATAAGGACACAAAGATGG
CACACACATTTGTGGACCATCTGAATTCTTATAc
>NC_000015.10:37077748-37078094
AGCTTGACTGTCCTGGGTCATGTTTTTTAATCCAGAGTATTGGACATGTAACACAGCAACAAGAGCTG
ATCATTTTGTTTAATCACCTTTTGGATCTTTGCCAGATGGTCTCTATTGAGAGCGCTGCAAACAGAGA
GAGATGAACTGCATCTAACACGCCACCAGAGCTTTCCCGTAAATGTCTGAGAACTGATTCCAACAAG
AATTTAGTCTCAGAGCAGAAAGGGATACATTTAAAAACTATGTGCATTAACTATACTTTTCCCCATAGA
ATCCTCACTTGGCATGAATAATTTACCCACTCTTATGCTAATGGGATCATGAAAGTATCCCTCTGTCTT
TAGTG

>NC_000015.10:37078674-37078871
TCCATGTTAGGTAGTTCACTGTGTAAAAATTATCAGGCTAAAACATCTGGGAAAGAAAGCTATGAAAC
AGCAGGGAACGTGGCTggtttattgtttatcttttgtttatGAGGCAGTATTCAAGTTTAATTACTACTCTAAATTAAAC
TCTAACTGGCATCAGGAGACATCCCTTTACAtgactcagttaaaaaaa
>NC_000015.10:37079299-37079439
GGATGAACTTAGctctaggaaaaataaaagttagaagcAACTAGCAAAAGATTATGCCAGATGGCATCTGTCATA
TTGTATTTAgggttattttaaagagattgcCGCCACTATGAAAACATTCCATTGACAGAGTTTTC
>NC_000015.10:37079893-37080177
GGTTAACGTGCGTGTGGTGTGCAGTGTGAGCTCAGCAGTTACTGGGCCAACTGTCTCGGTATTTCTG
GGAATCCTGCCTATTCACAGTGCAGGGATTGTTCAATTGCTGCAAAATGTACAAAatgaattttacaaaaatga
attgtAAGGAAGGCCCCATTACAACGATTTTACAGCAAAGATGTCAacatcaatttttctttcatgcATAGCTAATGA
TTAAAACAGCATATTCCATTTGCCTAAGACAATTTATTTCATATTGGCACatcaaaatattgaaa
>NC_000015.10:37080227-37080379
ACCATTGTTCaaaccaggaaaaataaaacaaacttggCACGAAATACTTAAAACAAAGGCTCATCAATATTCTCC
AATTTGTCAACATCTCAATTACAGCactggaaaaaatgtaaatttcatgtGCTCTAAACACTGAGGGGTCTATTCT
C
>NC_000015.10:37082062-37082231
AACAGAACCAGtcagttttaaatttcagttgGGACCTTGGTGACCTGTTAATGAATCTACAGAGAGGAGAAAAAC
TCACAGTGTTGAGTTATGCCTGGTGTTGCTAGCTGACTTGGAGTCAGAAATGCTTGAAGCGTTAACG
TAATTGCAAGAATGTGAAAAATGAGGC
>NC_000015.10:37096355-37096489
CCAGGTTCCCGGGGAGTGCAGGTCGCCAGCTCGCACTTCTCAAAGACCAGAGCTAACAGAGGAAAC
AACGGGTGCCTAACGGGCAGCGCCacgcagagacacacacacagagacggGGTGCAGAGGGGAAGGAG
>NC_000015.10:37098890-37098997
TGAAAGTTACCAGAAACATTATTTAGCAGCGGATTCCCTGCGTCCTTGTCAATTTCAGAGACAAAACA
AGCAGCCCAGGCTAGTAGTCTAGGCGGCGGCGCAGCGGC
>NC_000015.10:37099402-37099552
CCCTCTTAGGAGAGGATTTATATCTAGGTAAGTGTTGGAGATTTAAACCTACCCTTTGCGCCATCAGT
CTGCGCTCCAATAAACTCCTGGATGTGTCGTATATTTAATATCCCAGTCCGGATAAGAAAGTGATCTA
GGCtgaagattcct
>NC_000015.10:37099841-37100028
aaaaaaattgtcaagcCCCCGAACTGAAGGTTACGAGCGGCCCGTCAGCAGCCCACATGTAACCGAACTGT
CGTGTTTCATGGCTTTTTGCCACTCCAGCTGTCAATCAAAGCCCGGAATGTCAAGGTAGTGAATGAA
TGATGGTTGATGATGATGAAGAGGAGGAATCTTTCAGACCCGTTTTTTT
>NC_000015.10:37100324-37100524
ctctgggagATGAGTGAGTGTCAGTAGGTGTTGGCAGGTTGGCTGCTGCCTGGCTTATTGAAGAGCCTC
AGTTTGGCGGCGCGGGTCGGCGGCGGGAGAACGAAATGACGGAACCCGGAAGTCGTGGTGGCCA
TAGCCCGTCGTACGGCGGAGACACATCCCGGGAGTGGGGAGCGGGAGCGAGGAGGAGCGCGCCG
CGC
>NC_000015.10:37102308-37102449
CTCCGGAGGTCAATAAAGCCCTCCAAAAATGATGAATGATTGAGCACctgtgatgatgatagtgattaCCAGAG
CAATTAAACAGTTTGATTAAATGAGCCATCATAACAATGATGTCTGCGGGAAATCGGCCCTCACATA

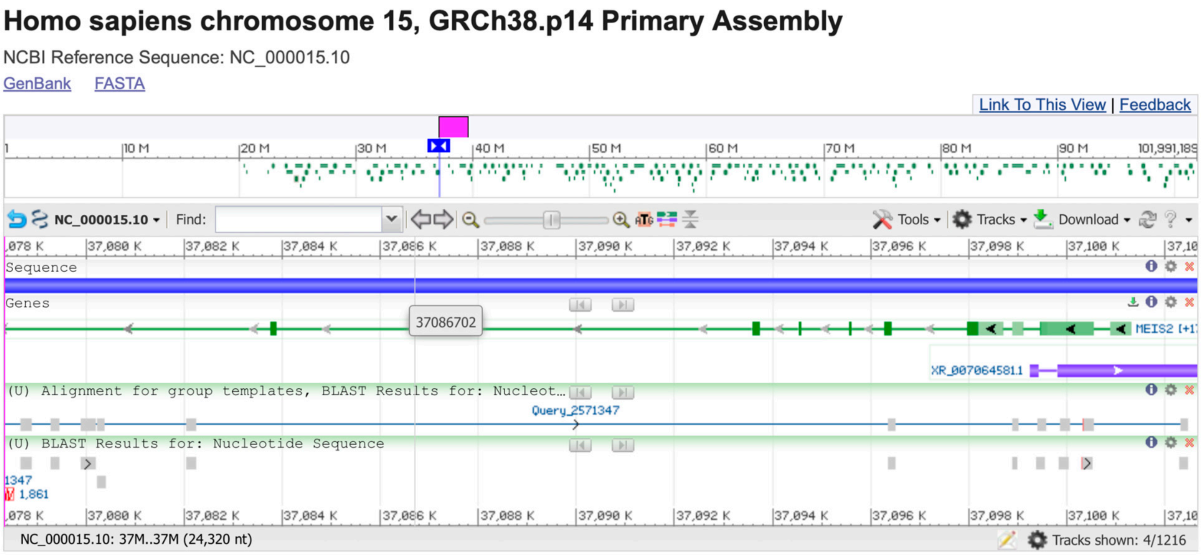

The same MEIS2 gene as the previous two clusters

chr15 CLUSTER 47 positions on BED file: 10321 - 10335
>NC_000015.10:96312041-96312237
CCATTATCCTTTTCCATTTTGAATGAATGAGGCTAATTAATTTCAGGCCATACTTGATGAGGTCAGCAC
CATACGAGGGGTGCGTGCTAGACATTGCTAATCTCTTTCAGCTGAGAGTGATGAATTAAAGATGAAG
CATGCCTTTGCAGGCCTAATTTAGAATGTATATCAAAAAACCTACAGCAAATAATACTAT
>NC_000015.10:96315373-96315572
aattttctgattGCAGATAACTTCTCAATTTGATTTAGTGTCGAACGATAGTGATAGGGTTGATAAGTGAGCA
TTCATCGGTAATTCATCTTGTGCTGGTAGAAATTTACAGCAAAGCAGTGCTTTCCCAACAGTGACAAG
CCCTATTAATCTTCTTCAGACACATTTCAATAGGTGTTCCCATCTGTGGCCAACATCT
>NC_000015.10:96317058-96317164
CAGCCTAAAGAGAAATCATTGAAACAAATTAATCCTATGCTGTTTAAATTGACATGGTAATCCATCATC
AGAGAAGGCAAACTCTGAAAGCAATCTAGAATCTGCC
>NC_000015.10:96323820-96324014
aaaaaaccctgcccaGTGCTTATTGTATTTAGCACCAATACAGGCAGAAAATGTAGCACAAGTTTTTGGTTG
AAAGAGCACCTCTGTGCCCATTGAAGGGCGTTGATCCGAAAAGACAGAAAGGACAAGTAAAATCGAT
TTGAAGCGACCCTCTCTGTCCCTTTCACACCATTCAGAAACTCCATTCCTTCTAA
>NC_000015.10:96324889-96325166
GGAAAAACTTCTGTAGCCCATATTTATGGTATTAATGAATAGTTAAAAAAGATTTACACTTCTTATGAT
CAATATTCTGAGTTATTTTGCTCAAAGTATATAATTACAGTGTCTTCAGAGTTTTCATCATTGTTTGAG
GTGGCAAAACCATTTGTAATTTTCATTAGCCAGTAAACATGTAATTAACATTCAGCACTGTGTTTAACT
TAATTCCTGTTTAAGACAAGGACAAGAAGCAGCCGAAGAGCGTTAGTTCCATTTGTCAAGTCTGACAT
AC
>NC_000015.10:96325816-96325982
CTAAACTCATTAAGTTGGGAGATTTTTTTCAATTGGACGGGTGTTTTTAAAGTCTCGTCTTTCCAGCCC
CAAACAAGGTGTAACAACGCACTCTTCCTTCTAAGGAATGAGATGAGAGACAAGGATCACTCCAGAC
ATCTCCTACCTacggtttggggttttttt
>NC_000015.10:96326256-96326360
GCATTTATTTCACCCGCCAAACTAAAGGAGAGTTATTCCAGTTTAGGAGGAAGATGCAAGCGGTTTG
GGACCTTGAACAAGGCAAATATGGTTTTGGTATGTTT
>NC_000015.10:96327439-96327605

TAATCTTAAGCAATTTTTTCCCTCCCAGCATATGCTCTGATTTAGCACTGTAAATTTTTCAGAGGACCT
AACTCTGACAGCCCCTGGTGATTTTCAAAGTACCACAAGCCAGTGGTTAAAAATTGCATTGACTTGGA
AGTTCAGAGGGACACAATGGAAGCCCCTT
>NC_000015.10:96330570-96330833
GCGCGCGCGCCTGGCCAGGCCAACCCCGGCTGCTGCCTTATAAGGCGCGCCGTCGCCATGGCAAC
GTGCGCTAAGTTGCAGCAGTCGTGTCAAAGTTCACTATATAGAGAGCTCAGTGAGCTGATCGCGGA
GAAGCCACTTCTGCCAGCCCCGGCGCCTATAAATCGCATTCCCTCCCGCGCCCCCCTTTTTAGCATA
TTTGATCACTTTgattctctgttcttttctctccgcggtgtgtgtgtgcgtgcgcgcgtgtgtgt
>NC_000015.10:96332330-96332560
cacatCGAGTGCGTGGTGTGCGGAGACAAGTCGAGCGGCAAGCACTACGGCCAGTTCACGTGCGAG
GGCTGCAAGAGCTTCTTCAAGCGCAGCGTGCGGAGGAACCTGAGCTACACGTGCCGCGCCAACCG
GAACTGTCCCATCGACCAGCACCATCGCAACCAGTGCCAGTACTGCCGCCTCAAAAAGTGCCTCAA
AGTGGGCATGAGACGGGAAGGTATCGGCCTCTC
>NC_000015.10:96336725-96336867
AATGAACAGAGAGATCACGGTTTGCATGGCTGCCCTTCAATAGGCTAAACTGACAAGATCAGTTTTAA
AGGGCCACTTAAATCAGTTTCAAAGACTGGAGAAAAATGAGTCGCCCTCTTTGGGGGGAATCTGAGG
CTGGATC
>NC_000015.10:96337490-96337626
ACCGTCTCCTCCTCAGTCATAGAGCAATTGTTTTTCGTCCGTTTGGTAGGTAAAACCCCCATCGAAAC
CCTCATCCGGGATATGTTACTGTCCGGCAGCAGTTTTAACTGGCCGTATATggcaattcaataaataa
>NC_000015.10:96338345-96338482
ACAAacctagcttttaaaaaatattttaagaaagagaatgaacTGTGGAATTTATTGGCAGCCAAGGAATGTGTCCAAGA
CACATGCTGAGGTTTTGAATAAAAAGTGAACTTTTGTAATTTGAATTGGGTCCCGCT
>NC_000015.10:96338565-96338665
tttaaaaaataccattgaAATCAGCATGACAAAAATAACACTGTTGGCACTTATAGGTAACGTGATTGATTCAG
TATCTTAGAGTTTAcagtttgtgttt
>NC_000015.10:96338680-96338821
tttttttttaagtgcaacaTTTCTGTATACTGTAAAAGTTATAATAACTGAACTGTTTGGTCGAGTCTTTGTGTGT
TATATTCCAAGGAAAATTGAAAgtattcagaaattaaaatattatttgatatcTGAAACCTGGC

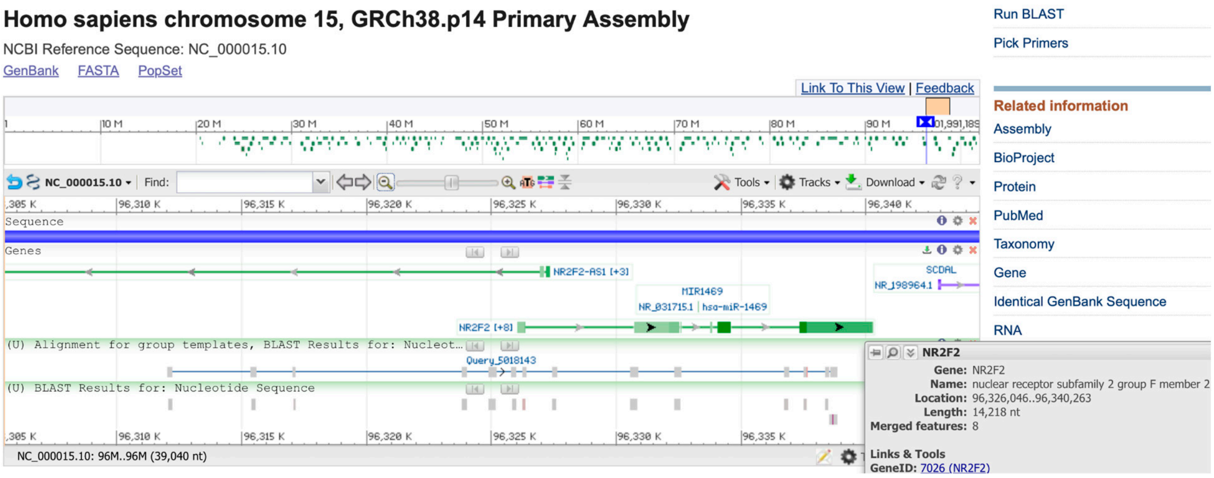

chr17 CLUSTER 48 positions on BED file: 11098 - 11108
>NC_000017.11:48568829-48569008
ACACAGAGCACCGTGTGAAGAGAAATGGCAGGAAATGAAGATGGCTATTTGTCACATTTTACGACAA

TAACATTAATAACAAACAATAAATTTACATGGACATATAAGACGCGGTAGGTAGTGAGAAATCCCTTC
TACTTACAATACCCCAGCCCGCGGTGTGGCTCCGGCTGCGGGGC
>NC_000017.11:48574005-48574157
GGAGATTTGCTGTTGAATAATAACAAAGGAGAGAGGATAcgtatttaaagaaaagaagtgatTAATGGTTTTCTG
TATAATTCTCACATTTTTCTTAGCTCTTGTCTACACAGAATGGCTGTGAACTTTAAGCTCcatagaaatcttttt
tt
>NC_000017.11:48574818-48574929
ATACTTtctatgagaaagaaaatgaaaattaatactGGAGCAAGGCTTCACAAAAGAGGCCATTTGTCTTCTTGATT
ATCTATTTGCTTGTCAGAGTTTGAGCTATGGCAC
>NC_000017.11:48578260-48578428
CATGGAGGGAACTTGGGGTCGACATAGTTTGAGTTGATCAAAAAAGAACTCATAGCCATTAatttctggga
attgcccacaaaatatactaaaatttattCCGACCCCTGACTCGTTTTCCTGTTTCCGAAAGCCCTCCTACTTACTGT
CAAGTGAACAAAGTTAGGC
>NC_000017.11:48579830-48579962
aaaaataAGAGCGGAGTGTTTATGTCAACTACATATTCCCCTAGATACGAATTTGTGACCACAAAATTCC
TTACACAAATTCGGATCTACAGGGTATATACAGAAGACAGACAGATCTTCTTGGCCCAGAAA
>NC_000017.11:48586522-48586667
CCCCGTCTGCTGACCTCTGCATGATCCCGGACTCTATGAATTATTGATGAGATATGAGCGTTGATTTC
CCCTTTCAGGATGCAAACTCcattatattgttaaaatggcgaTTTAATCGTTGAGAATAGCTTTGGTGTGGGTTTT
T
>NC_000017.11:48590225-48590449
GAGCAGTTAAAGTGTCACTTAACATTCTGGAGAATGTGAAATATACTGCGCGGTGTCAACTCCCCAA
AACCATAAAACTAACTTTATGGACCTCACGTGACTTTCTCGAGCCAGTGAGGGGTATCTGTCTGACTT
CTCGGCGATTTTTACGATCTAACTTCGAGATAAAACCCCTATCCATTTGACATCTAAATGTCATAGCG
ACTTTTGGGATAGTTTGCTAT
>NC_000017.11:48593452-48593819
GGGAAGGCGCGCGAGCTCTCGCCCACCGCCCCAAAGTGGCTGGAGGAGGCCGAGGAGCGGTTGA
CGCTGAGGTCCATCCCATTGTAATTGTAGCCGTAAGAGCCGGTGTGCATGGCAGCGGGATCCCTGT
AAGAGCCGCTCAGAGAGCTGCCACTGCCATAATTTAGCAACTGATAGTCCGGGCCATTTGGATAACG
CCCCGAGAAGGAGTTTACAAAGTACGAGCTCATTTGGGTGATTTTGGAGGGCTTGATTTGTGGATCG
TGGTCGTTATAACCCTGTGCTTCACGATTTATGATGTATTAATGAATTATAGCGATGCACTGTACTTCG
TTTTGCTATGTATGCGGCCAAATATGGGGGGGGG
>NC_000017.11:48593821-48593975
TGGAAGGGGGGTGGGGGGCGTTAGGGAATCACGTGCTTTTGTTGACCAGTCGTAAATTCTCGCTGA
TGACCTCAAGAGGTAATTCATGCTCTATGGTTACAAATAATGACGATCCGAGAATCGTTAGGGCCGA
TTCAATGCGAGCCTCcgagag
>NC_000017.11:48595828-48595966
atcgaaGTATTTCACGTCCAGAGCTAAGACAAGACTACAAACACAACacatagaaaattaataaaatagaactttgttttc
ttctgaggcTCCTCTTCTTACTTCTAGGTAGTATGTGCTCCTTCCAGTGGctttggg
>NC_000017.11:48598163-48598392
GCGCGCGGCCGCTGCTGCTCCGCCGGGTTTATGATTTGTTGTGTTTTATAGTCCGAGCGCCGCGCC
TATTAGTAGTATATCCGAGATTGGGTTTTAGCTGAGGGGGGATGGCGAGGTGCCAGTGAGGGCCAG
AAGGAAATACAACCATCTGATCCAACACTGACCAATGGCAGAGGCAGGAATTGTCAAATAGCACCCA
GGAGGAGCGCAGCCCGCACGAGGGACCCGC

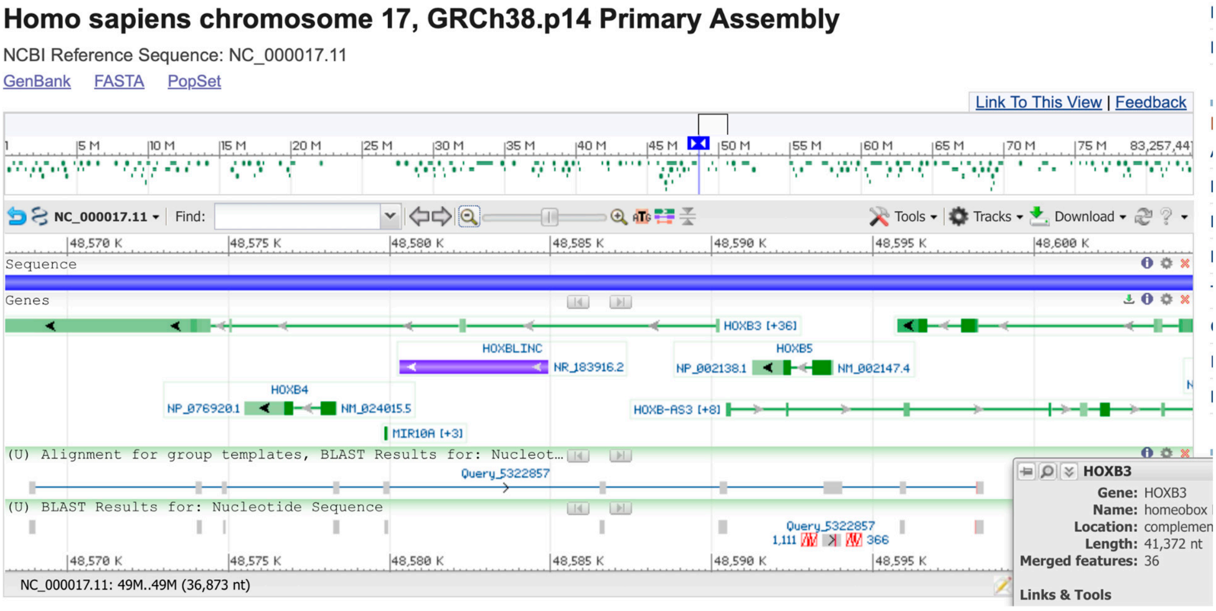

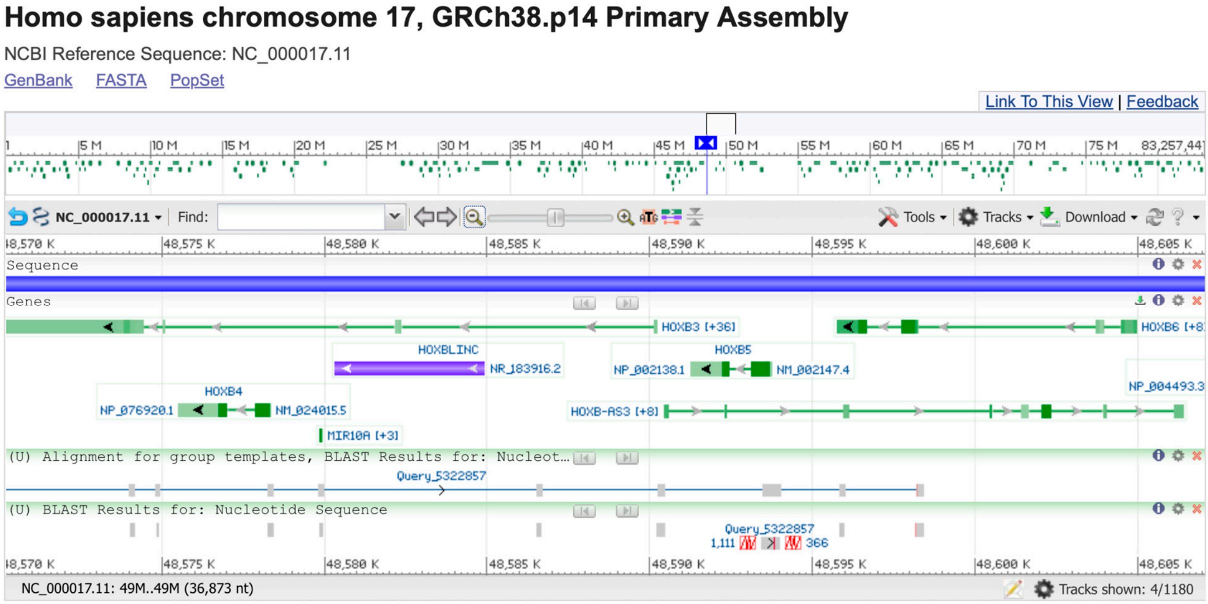

chr17 CLUSTER 49 positions on BED file: 11108 - 11118
>NC_000017.11:48598163-48598392
GCGCGCGGCCGCTGCTGCTCCGCCGGGTTTATGATTTGTTGTGTTTTATAGTCCGAGCGCCGCGCC
TATTAGTAGTATATCCGAGATTGGGTTTTAGCTGAGGGGGGATGGCGAGGTGCCAGTGAGGGCCAG
AAGGAAATACAACCATCTGATCCAACACTGACCAATGGCAGAGGCAGGAATTGTCAAATAGCACCCA
GGAGGAGCGCAGCCCGCACGAGGGACCCGC
>NC_000017.11:48604910-48605171
GTACAAAAATATCAAGAGGCTGGAGCCCTGAAGGACGACAGTGCTCCGACCTAGGTGTGGTGTCCA
AAAGAATGCTGCATTATAACCACCAGGGAAATGATAAAAAGTTCATGTTCACGATCGCCCGGCCACA
TGACCGGCGCCGGCCAATCGCTGGATTCAACCACTCATAAACTTCTATCACAAAGTTGTAAATTTTCA

TAAAACAACAAGGAATTTATTGCATTTCTTCATGGCTGCTCCACCAGCAACCCTTTTCTC
>NC_000017.11:48610850-48611049
GTAGCGAAAACCGAACTTGAGGCtggatatttagaaaataaagtattcGCATAATACAATGAACTCATAATTTGGC
CGGATGATTTGTAGGCAGGGACGTTTTAGTGTCGGTTTTACGAGATTCCTTGATATATTACAGAATTA
GAGTCCAGATTTACACCAAAAAGGACCCCCTTTTTCCTCTCCGGACCACGTGACC
>NC_000017.11:48614575-48614739
GCTGGGACCGTACACCACGGTGGGTCGGCCGCCCAGGTCCTGGGCGAAGCCGCAGTCATAATAAT
TGGGGCGCAGGGACTCCCCGGTTTTGTATTTGGAGAACAGTGAGTTGACGAAATAAGAGctcattttattga
attttgaggcggcggcggcggcggagg
>NC_000017.11:48615002-48615219
cccccaccccccaaacaaAAATATACCACCACTTAAAGAGGTCCTCGCTTTACATTTCGAAGAAGGGATGCC
AGTTCATAAACAGTTTTCGGTGATTTACTTCCCTGCAAATGAGTTGTTTCATATTTTGCACTGTCTTTT
CATGATCATTTGCATCCATTAGAGACCCCGCATCCTATTGGCTTCTTCGTACTCCTCCCGGACAGAAC
GCAGAGCG
>NC_000017.11:48615575-48615814
TTGGAGTCTTTCTAAATGTCAGGGTCGCTTcggaaaaatgtaaaagaacacggTTTAATTGCGCCGAGTTTTAA
AAGGTCCTATAAATGTAATTGGTATTTTAATACAAGTTATCAAATCATACAGCTTCCTCTCCTAAACGT
ATTTTCAAACAAACGAGGTAGTCATATTTAAAGCTTTAATGATCAATTTCCTTATTATTGATAAAGGTTT
AACTATCGTGTGGAGGGAATTGGCTG
>NC_000017.11:48615830-48615964
AAAAACGGCTGTAAACTCTTTAAGAAACTATAAAACGCAATAAAGCCGGAGTCTGTTGTTGTTTATGC
CGCAGCGCATAAAAGCTGTTAGCATTTTACGAGTTCTTTCCTCTACAGCTATAAAACTGCAGCTTC
>NC_000017.11:48619561-48619665
TCGCTGTTTGCTTTGCTGCTGTTTAGTTTCTTAACCTTCAGAAATTTATACGCTATAAACTTTTATAGC
GgtcatattattttattgctgCGCACACGGCATTG
>NC_000017.11:48622915-48623103
TccttattcattttcttcattttcatccgCCGGTTCTGAAACCAGATTTTGACTTGTCTCTCACTCAGATTGAGGAGTCT
GGCCACTTCGTGCCTACGGTCCCTGGTGAGGTACATATTGAACAGAAACTCCTTCTCTAGCTCCAGC
GTCTGGTATTTGGTGTAGGGACAGCGCTTTTTCCGGGAAGA
>NC_000017.11:48625722-48625870
CTGGGTATTTCCTCACTTTTTATAACTTACTTTGATCCGGCCTCTCTTTGTCCTCGCTTCCTTCGCAAA
TTTTATTGTCCCCGTAGCCGGGTCTTTGATTAGACAGCACGGCCTCCCTGCCCGCCGAAGTTTCCAA
ACTGTACTCGGG
>NC_000017.11:48626412-48626517
gctcaACTTCTCAGCCAACAAAGTACAGTGGAGCAGCCCGTCTCAGCTCTCCTTGCAAAATGATTGGTC
AAAGTTTTTCCGACTGCCTGATAAAGCGTCAGCTCC

Homo sapiens chromosome 17, GRCh38.p14 Primary Assembly

NCBI Reference Sequence: NC_000017.11

GenBank FASTA PopSet

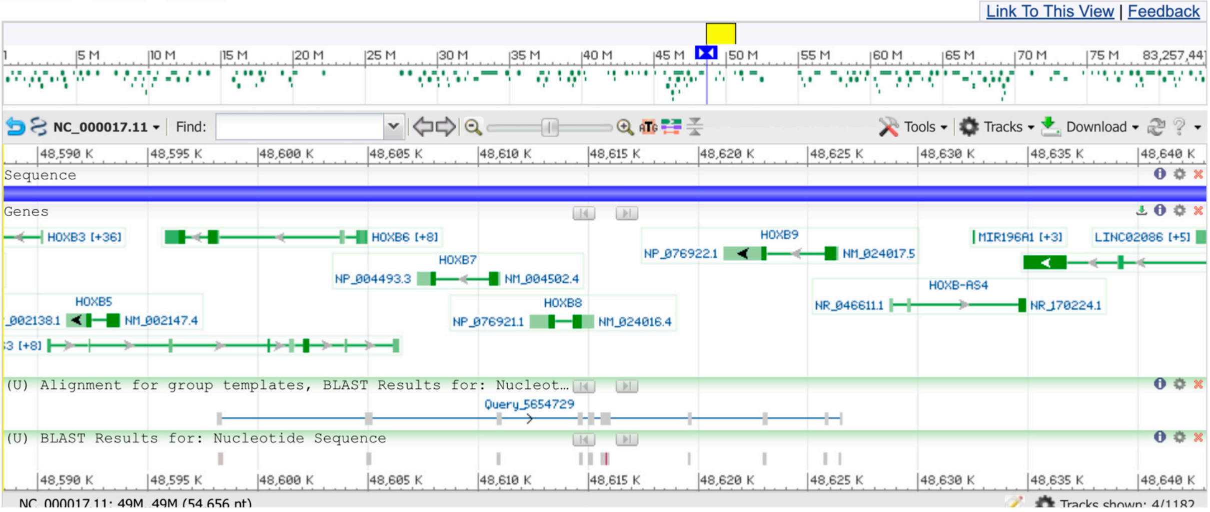

The same HOX genes as the previous cluster

chr18 CLUSTER 50 positions on BED file: 11322 - 11332

>NC_000018.10:25163456-25163603
TTTGGTCCGACACCATCCCCTGCAGTGTGTGGAGACAGGCCAGGAGTGTGAGGGCTGCAGAGCAG
AGCAGGAAGAAAATCAACTGTGAAATTGGTAATTTTATTGAGTTTCATGGGGGGCTTTGTTGTTAAGA
CACAATGCAATTGG
>NC_000018.10:25165225-25165418
GGGAATTAGATTTTTCTGAAGTCTGGGTTGAGCATGCCCATAACTCTGACAGAGCCCTGCAGGCTCT
TGTCTTCACCTTCAAATCATAAGACAGAGAAATCAAGCTCCCTCTGTGCTCTCAATTATACCAAAAAA
CACGGAGCAGAAGTCTTCAGTAATTCAATCCCTGTGGAAGCTTCTTCACACTGTTTGC
>NC_000018.10:25168008-25168162
ATCTTTTTTGGTGCTCAGAATAGCTCCAAGGTTAAGAGACTCCGAGTCAGTCTTCTGCAGATCCATTT
CCTCTCTCCAGGCTTCTCATGTTAACCCAGTGATTTAGTGCTCTGCATTAagagcaattcacctgcctctggTGT
CCCTGTGGCCC
>NC_000018.10:25168163-25168481
TCCCTCTAGGGAGCGAGGTGGAGAATAAAAAGTTCCACATATGGTAAAGTCAAGCTGCTTGGAGCCT
GCTGAGTGTCTGCTGTGGGGATAGCGGCTCCTGAAAGCCCCAGCTGGTACTTGGAAGAGATTCTGC
ATTACTACACCTCAGTGCCTGCCTGGTTCCCTTCGGAAACTGTTGGCTCCACATACGACAGCCATTTT
AGATCTCTTCAACCCTTTTCTCCCGGTTCTGTATCCAACTGAACAGTTGGTCCTCTACTGCTAGATAA
TCAAATTCTTCCATCTGGTCCTATCCCTGTGGAACTGAAAAGGTGTAGT
>NC_000018.10:25174727-25174991
AGGCTCAGGAGACTATAAACAACATCTTTCACTGCTCTGACCCCCATGCAAATTTAAATCCCCCAACC
TTTCTGAGCACTGTTGGTGAGCAGCCCCATTACATCTTAATGACTGTCATTAATTTCTAAAGAGTgttaat
ttcagatttttaatcACCCCTTAactatttaattaaatttcaataaaatgttaagtaCTTACAAGTCTTCTCATTTTTCCTAAAATA
CTTACCACATTAACATGACCAAGGTCCAGCGGTAAAAG
>NC_000018.10:25175953-25176694
TTCCTGTAAATAATGAGGCCCATGCCTTACACACAATACAGCTAGAAACCACAGCACAGCTCAGTTTT
AAAATGATTAACTGCTGCATACAGCTATGACTTTATTTGTAAGGAGCAGTTAGGAGAGGCAAAATGAG

TATGCAGCTTTCCATTATATACAGGCATATTTTCAATAGCCGTGTGAGTCTTTTTATGGCTCCATTTAT
GTCAATGTCCTAGTGTCATCTGTAATAAACTGGCAGCAATTAGAGCCACAATAAACCCCATAATGCAA
CACAAACAACAGGAAGTCTCCCAGAACCCCAACGCTCTAAATTTACATCTCCCCCTCGAAAGTCTATT
TATCACCAGAGTTTGCAAGCCCGTCTGCTAAAGAGCGCTCTAATTAAGATGTATCTGGTGAACAAGT
GTCTGCTTTTCACCCTACTCTTTTAACACATCATGTATGCACTGAGCAATCTTCGTCGGGTCTCATAAT
GAGAAAACTGTGATATGCAAAAACTCTGTGAAATCTTTTATCCTCCCAGGAGACCTCCCTTGATGCCA
GGCATTCATCATCTAGCCTCTAATATCAGTTATTTTGTGCCTCCTCTGCTTACACCagaattaatttttgctttaatt
acTCTCTTCGCTGGAATGCTGATGAAGGAGAGGGACTCAGCATTTGGGGACTTGGGCCTCATTCCAC
TGCTTTAATTTAAATGAATTCCACTAGGAGAGGCAGGCAAACAACTGGCAG
>NC_000018.10:25182240-25182441
AATTCTGGCAAAGttctctcttcacagatgatGCACAGCTTAAGCTATAATGAGTTTTTGGGTTACAAATGCTGA
ACGCATTAATATTCCTTGATGATAATTATCACAGTATAAGCACCTTCCCCAATGTGAGTAGCATAAAG
CAACTGAAGCAGCATTTTTACTATATTTGAAACAAGctggaaaatgggattttctaCT
>NC_000018.10:25186289-25186392
CAGATAATTGGATATTAACCAGAACATACTTGACTGGGCAGGAGGTAGAGCCCTGCTTAAACTAATC
CTGAAATATATTAATCACCAATTAATGAAAGGGTTT
>NC_000018.10:25187727-25188182
GCTTAAAGACTGCATTTAATTGACTGATAGACAGTAGCTTCTTTGTTCACAGAAGACAGCGAAGTAAT
GGCAGCTTGAAAATCGTACCATCTACAGGGTCTCCTGAGACTAGTAACAGGGGGATATGTgtcaggaata
aataaataatctaattatTAAGCCACTTCCTGTCAAGTTAACTCCAATTTGTTGGCTTTTGAAGGCAACAAAAC
GAGGCATTTTTCCTCATTATGCTTCTCTTTTTATACCAGTGGTAATCACAGGCGGATTAAAGGGTGCC
TGATTGGTTTTTTTGTCAAGGAACTGAGAAGCAGCCAACAGTACAGAAAAACAGAGTTGGGGTTAGA
AGAAATTTCATGCTTGTCTCCTTTCCAGCTCCCTAACAGCCCACACGGCTGCCCCATACAGAGCTGC
CAGCTGTACAGCAAATGTGTACCCCTCCAACTGAGGCCTAC
>NC_000018.10:25189830-25189934
TGGTGTTTGTTATTCTGCCTGGTTCACTTGGACCAACTCtaagaaataaatagaattatcTGTGTAAATCGATGA
ACATCTATGAAAAACAACACCCAGCAGAA
>NC_000018.10:25189939-25190129
AGCTTGCTCCATTACCTACGTTAATATCATGACAGCTGAGGAGAGATGACCTGGCCCACTTCTCATCT
CTCCTGTACAGATGGCTATTAAAGAGAAAGTATCCCTTTTGTATGGTCCATGTAGAAACCCTTTATGG
GCTTTTCAAATAAGCCAGCATTGCCCACATGAGGGTAGCTTTCTGTTACCATTA

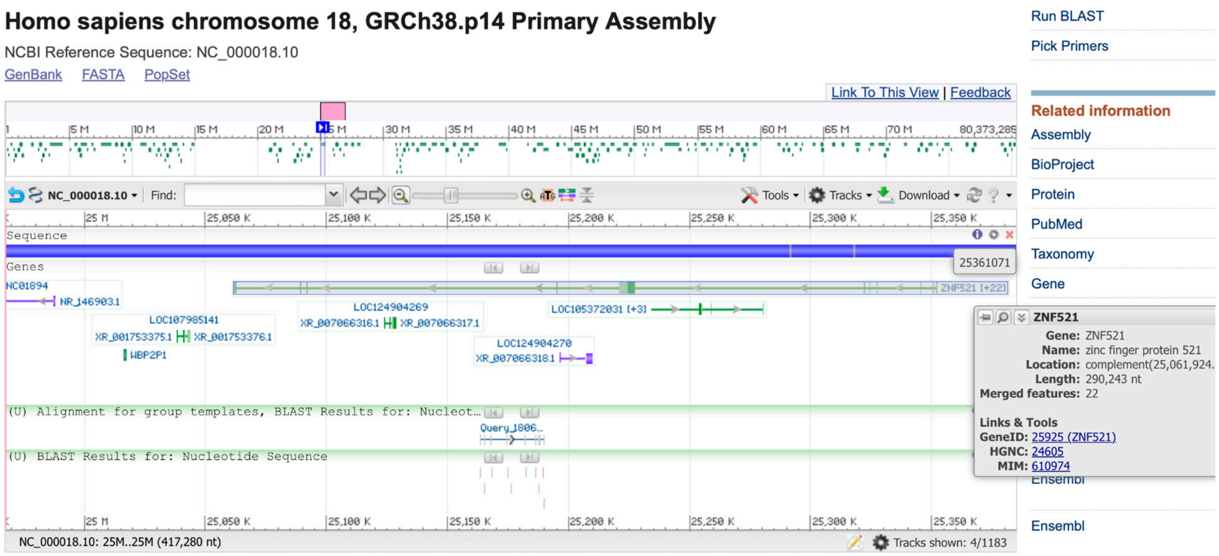

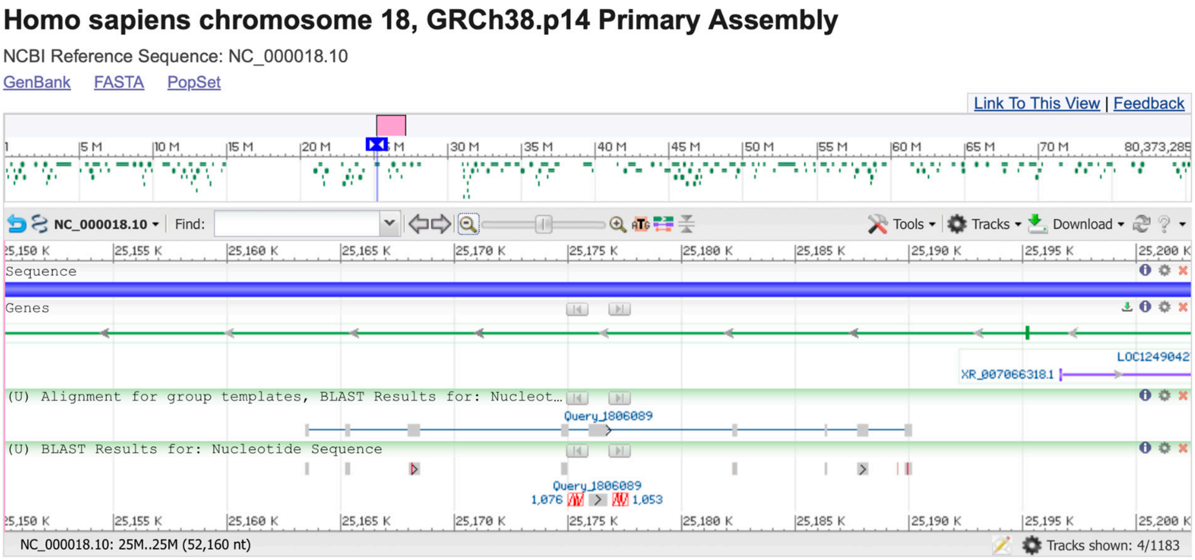

chr19 CLUSTER 51 positions on BED file: 11788 - 11798
>NC_000019.10:30350237-30351083
GGCGTTTAAGCCTGCATCACTCAAAACAGCTTTTGTGGATTAGTAAGCAGGTGAAATCAAAAATCTCA
TTTCATATCTGACATTAATAACTTTCAGGGGGAATGAGCATTGGCATTTATGTCTGGACCAGAATCGA
GTCCAATGTCCTAGAAACACAAGGGCAGCAGACAAACCTACTCACCAGATAGGAAAATAAATCATGC
ATAATGCAGAATTTCTACCTTACATGTTAATTAATGTGTGCAATCAATTACTGATGTTACAATATGAGA
AAGAAACTGGTGGGGAATGGGGTAATAAAAAATACCTATAGATTTATCtgcttctttttctatttaaacaaaTAGTC
TTCAAGCCTGTGAATGCTGTGTTGAGACATGCTCTGAATAATAAGCATCATAAAATCTAATCAGATTTA
TAAGAGGAATTGATTGTTGTGGCAGGAGGAGAGGGTGGAAAATAAGCTGAAATTTAAGTGTTAATTTT
TATGCTAATCCACAGTGGCTTCATATGAAGCAATTTTGTTCTGTGCCACAAATATAAAATTGTCCCTTG
CAGAGAAATGTCAGCTTGCTAGACCTGAAAGCAGTAATTTATTAGGTATGTTTCTATTAGTATGTGTC
ACAGTTGTTAGCAGGCTATACACACAATGGTGCTTCATACTTATCCTCCAAGTCTCAGAATGTTTCAG
CTCAGTGGCTGGAACGTGGGGCATTAATACATTATCATATGCGGGGTGGTCTGTGCCCTGTGGACA
AAAGGCTATTTAATACAATTTAATGTCATTCTGAGAAGAAGTGGAAAAATAAAGACGGGGAATAAAAT
GGCATCTGTGTGTTTCACTG
>NC_000019.10:30351274-30351577
GTGACAGTGACCTTGTTTCAGAATCCTTGGAACCCTGTCAGCTTTGCTTCCAGTATATGGTTTCAAAG
AATGCACTTTTTGGACAATATCTAGTTACTGGGTAGTGAATTAATAGAGTTAAGGGTTCTGATGCAATT
CACATATAGGTCAATTACATGCGCCAACATCTGCTCTGTCACTATCATTTGCATATAGCATTCATATGT
TAATGATGCCTCTTTTTACTcaagtaatatatttaaaaaacaagatttgCATATCAGTTTGGATCAGTATATTACAAG
CAATTTATCATTTTCTCCT
>NC_000019.10:30351664-30351803
GAAAGCTTCCATTCTAAAGCTGCTTAAAGCCTTATAAATCAATAATGACACAGATGGACTGGTGATAA
TCTATTTAGAATGCCTGATAGCAAATATTGTTGTACAGATGATTAGAGTGGTAACTGTTTTAGCAGAAA
GC
>NC_000019.10:30352059-30352194
GCACAAGGAGTGAGAAAATAAAGTGGAGGTTGTCAGAAGTGGCAGCTGCTTGGAATCCTGATAAATA
TAAGCTTTTATTGATCTGATGAAAATGAAAGATCCCAGTGCTAGGCAGTATTTAGCTTCTTGTCTACA
>NC_000019.10:30360290-30360441

TGCAACATGGAGTTTCTGTGTGtgtaatattatttgtttttatttctgtaatccCTTTATTGTTTGCACAACTGCTGTCAGA
CTGTTGCCTGAAAGTATCACAGGATAATTGCAGAACGCCTGCATAAGTAATTTCTTATGAAATCGGGC
C
>NC_000019.10:30360818-30360957
AGTCTAACCCTGCTGTTATCAAAGTAACTTCCAGCTTATTAGCGACAAAGGACTTTTCTCCTCTCTGC
TGCCCTGTTCGCCAGGGCTTCCTGAATTAAACTGCTGTTTCCTGTCCTTTATATGCATGACCACATCT
GGG
>NC_000019.10:30360975-30361179
GCTGCTTAATAACAATCACCTTCCCTTTCAgtctctgttgattgtttctaaTTCACTCTGACAGATAGTGCCTGACA
ACTTGCCGGCTTTTCCCTCCCTCCGTGACCTGAAGTTACTCACAGCACTTATATAGTctgtcatttttaatatttg
atttcattttaagtTATGTGTTTTACTGAGAagatgaaaccaaaaaaaaag
>NC_000019.10:30370952-30371255
GTGTAAAAAATCACGTTCATTTCAAGAAGCTGTTATGGTATTGATTGTGGTAAAGACTTCCATTAACTG
ACCAATCTAAATCTCATAAGTGATGTCAGGATAGTTATGCATTTGCTTGGTTCAAGTATTCAGCCATTA
TGTTAGAATGCAGGCAGTGTCTGAATATGGctgttaaattatttctaaataccGAACCAGAACAGCTTAACAGTTC
TGCAGTTTGAGACTGACACAAAAATGACAATCTGCCacctctttgtttttaaatattaacgCTGTATTGGGATAGTA
TTTTTGGAGTA
>NC_000019.10:30371342-30371526
TCTCATCACTCCAGTATGTTAATTAGCATTCTGTTGTGGCCGCAACACTGAAAACATGTAATCTGTATT
GGTGTACAGTACAGCTGtgaacacacattctttttgTGAGTCCCTGGAGTTAGCCTTGCCATTGGCATATGCGT
TTGGGCCAAAAGTCAAGCCATCACCACTAAAATAGCACAC
>NC_000019.10:30373534-30373650
CTAGTGGTAAAAGTATATAATTAATAGGAGAATTTTCCTCCAGATGTGGCTATACTATAATAGAGCCTT
ATAAatgctacagaaagaaaaattgcttGGAACGAAGGCGTCCTCAA
>NC_000019.10:30374413-30374518
GCACTAATTGCTCTGTACTAATGTGTTTTTCTGTGATATCCAGTAGTGCTAAGAGAACAGAGATCCAT
AACCAATTaaatatagtgtttttttttaatgttaaaa

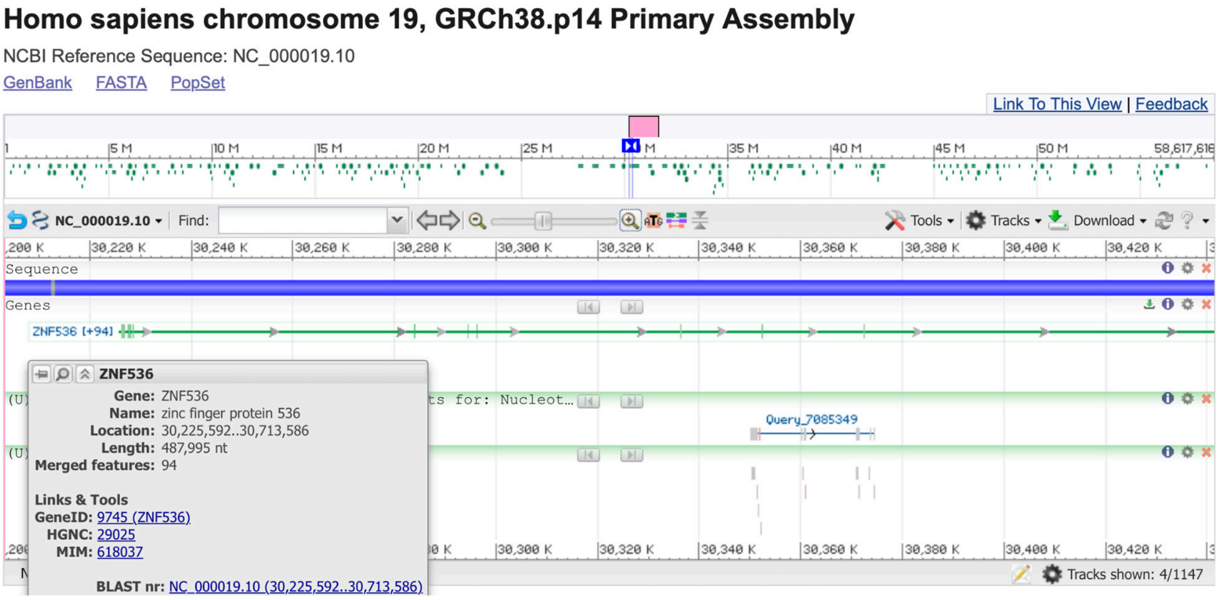

Homo sapiens chromosome 19, GRCh38.p14 Primary Assembly

NCBI Reference Sequence: NC_000019.10

GenBank FASTA PopSet

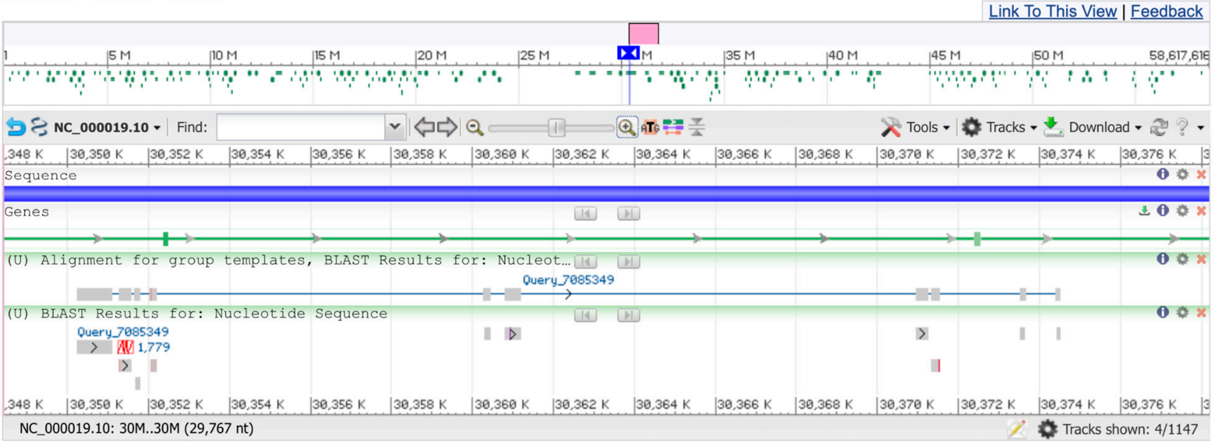

Chicken and mouse have the same cluster in the same Zinc finger protein

Gallus gallus isolate bGalGal1 chromosome 11, bGalGal1.mat.broiler.GRCg7b, whole genome shotgun sequence

NCBI Reference Sequence: NC_052542.1

GenBank FASTA PopSet

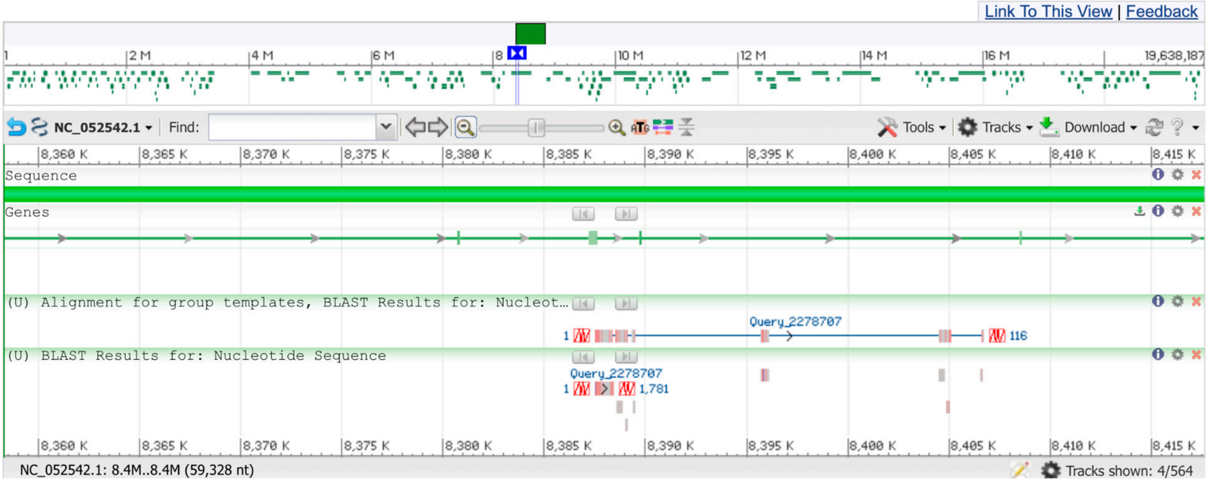

Gallus gallus isolate bGalGal1 chromosome 11, bGalGal1.mat.broiler.GRCg7b, whole genome shotgun sequence

NCBI Reference Sequence: NC_052542.1

GenBank FASTA PopSet

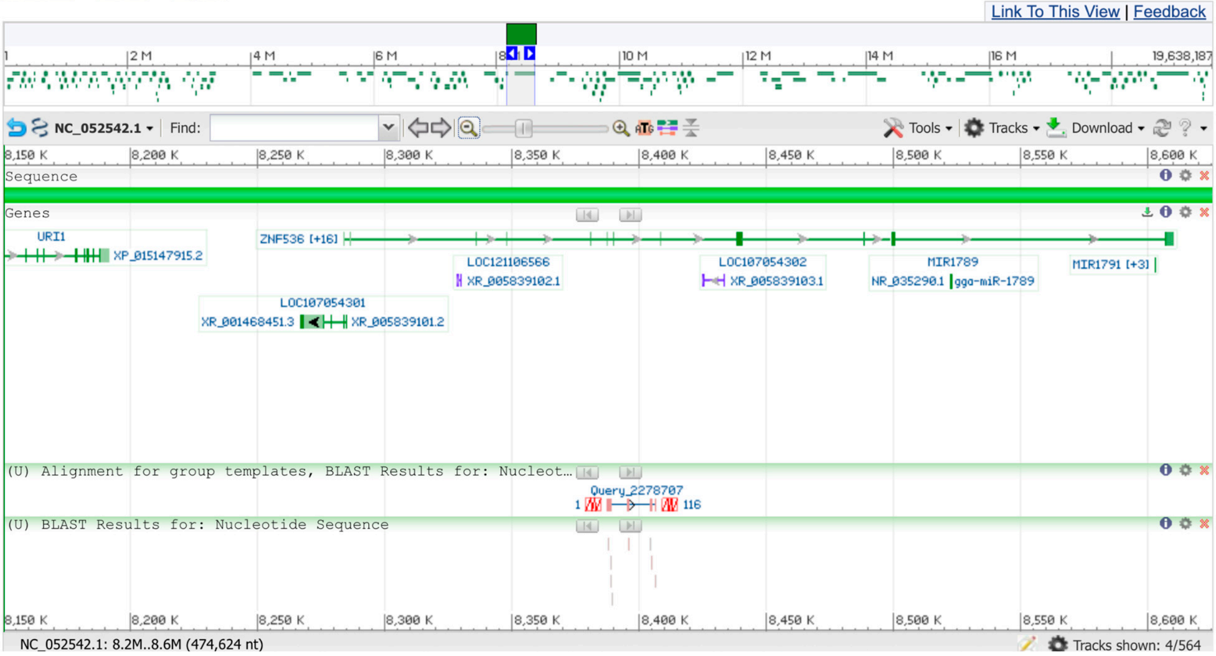

chr21 CLUSTER 52 positions on BED file: 12207 - 12217

>NC_000021.9:8209703-8209815
GGTACAGTGAAACTGCGAATGGCTCATTAAATCAGTTATGGTTCCTTTGGTCGCTCGCTCCTCTCCTA
CTTGGATAACTGTGGTAATTCTAGAGCTAATACATGCCGACGGG
>NC_000021.9:8210009-8211502
GCCGTGCCTACCATGGTGACCACGGGTGACGGGGAATCAGGGTTCGATTCCGGAGAGGGAGCCTG
AGAAACGGCTACCACATCCAAGGAAGGCAGCAGGCGCGCAAATTACCCACTCCCGACCCGGGGAG
GTAGTGACGAAAAATAACAATACAGGACTCTTTCGAGGCCCTGTAATTGGAATGAGTCCACTTTAAAT
CCTTTAACGAGGATCCATTGGAGGGCAAGTCTGGTGCCAGCAGCCGCGGTAATTCCAGCTCCAATA
GCGTATATTAAAGTTGCTGCAGTTAAAAAGCTCGTAGTTGGATCTTGGGAGCGGGCGGGCGGTCCG
CCGCGAGGCGAGCCACCGCCCGTCCCCGCCCCTTGCCTCTCGGCGCCCCCTCGATGCTCTTAGCT
GAGTGTCCCGCGGGGCCCGAAGCgtttactttgaaaaaattagaGTGTTCAAAGCAGGCCCGAGCCGCCTGG
ATACCGCAGCTAGGAATAATGGAATAGGACCGCGGTTCTATTTTGTTGGTTTTCGGAACTGAGGCCA
TGATTAAGAGGGACGGCCGGGGGCATTCGTATTGCGCCGCTAGAGGTGAAATTCTTGGACCGGCGC
AAGACGGACCAGAGCGAAAGCATTTGCCAAGAATGTTTTCATTAATCAAGAACGAAAGTCGGAGGTT
CGAAGACGATCAGATACCGTCGTAGTTCCGACCATAAACGATGCCGACCGGCGATGCGGCGGCGTT
ATTCCCATGACCCGCCGGGCAGCTTCCGGGAAACCAAAGTCTTTGGGTTCCGGGGGGAGTATGGTT
GCAAAGCTGAAACTTAAAGGAATTGACGGAAGGGCACCACCAGGAGTGGAGCCTGCGGCTTAATTT

GACTCAACACGGGAAACCTCACCCGGCCCGGACACGGACAGGATTGACAGATTGATAGCTCTTTCT
CGATTCCgtgggtggtggtgcatggcCGTTCTTAGTTGGTGGAGCGATTTGTCTGGTTAATTCCGATAACGAA
CGAGACTCTGGCATGCTAACTAGTTACGCGACCCCCGAGCGGTCGGCGTCCCCCAACTTCTTAGAG
GGACAAGTGGCGTTCAGCCACCCGAGATTGAGCAATAACAGGTCTGTGATGCCCTTAGATGTCCGG
GGCTGCACGCGCGCTACACTGACTGGCTCAGCGTGTGCCTACCCTACGCCGGCAGGCGCGGGTAA
CCCGTTGAACCCCATTCGTGATGGGGATCGGGGATTGCAATTATTCCCCATGAACGAGGAATTCCCA
GTAAGTGCGGGTCATAAGCTTGCGTTGATTAAGTCCCTGCCCTTTGTACACACCGCCCGTCGCTACT
ACCGATTGGATGGTTTAGTGAGGCCCTCGGATCGGCCCCGCCGGGGTCGGCCCACGGCCCTGGCG
GAGCGCTGAGAAGACGGTCGAACTTGACTATCTAGAGGAAGTAAAAGTCGTAACAAGGTTTCCGTAG
GTGAACCTGCGGAAGGATCATTAACG
>NC_000021.9:8212568-8212670
TACGACTCTTAGCGGTGGATCACTCGGCTCGTGCGTCGATGAAGAACGCAGCTAGCTGCGAGAATT
AATGTGAATTGCAGGACACATTGATCATCGACACTT
>NC_000021.9:8214151-8214321
CCGGGTCTTCCCGGAGTCGGGTTGCTTGGGAATGCAGCCCAAAGCGGGTGGTAAACTCCATCTAAG
GCTAAATACCGGCACGAGACCGATAGTCAACAAGTACCGTAAGGGAAAGTTGAAAAGAACTTTGAAG
AGAGAGTTCAAGAGGGCGTGAAACCGTTAAGAGGTAA
>NC_000021.9:8215319-8215583
TCTCCAGTCCGCCGAGGGCGCACCACCGGCCCGTCTCGCCCGCCGCGCCGGGGAGGTGGAGCAC
GAGCGCACGTGTTAGGACCCGAAAGATGGTGAACTATGCCTGGGCAGGGCGAAGCCAGAGGAAAC
TCTGGTGGAGGTCCGTAGCGGTCCTGACGTGCAAATCGGTCGTCCGACCTGGGTATAGGGGCGAA
AGACTAATCGAACCATCTAGTAGCTGGTTCCCTCCGAAGTTTCCCTCAGGATAGCTGGCGCTCTCGC
AGA
>NC_000021.9:8215599-8215965
CACGCAGTTTTATCCGGTAAAGCGAATGATTAGAGGTCTTGGGGCCGAAACGATCTCAACCTATTCT
CAAACTTTAAATGGGTAAGAAGCCCGGCTCGCTGGCGTGGAGCCGGGCGTGGAATGCGAGTGCCT
AGTGGGCCACTTTTGGTAAGCAGAACTGGCGCTGCGGGATGAACCGAACGCCGGGTTAAGGCGCC
CGATGCCGACGCTCATCAGACCCCAGAAAAGGTGTTGGTTGATATAGACAGCAGGACGGTGGCCAT
GGAAGTCGGAATCCGCTAAGGAGTGTGTAACAACTCACCTGCCGAATCAACTAGCCCTGAAAATGGA
TGGCGCTGGAGCGTCGGGCCCATACCCGGCCGTCGC
>NC_000021.9:8216130-8216377
CCCCGCGGACGCTACGCCGCGACGAGTAGGAGGGCCGCTGCGGTGAGCCTTGAAGCCTAGGGCG
CGGGCCCGGGTGGAGCCGCCGCAGGTGCAGATCTTGGTGGTAGTAGCAAATATTCAAACGAGAACT
TTGAAGGCCGAAGTGGAGAAGGGTTCCATGTGAACAGCAGTTGAACATGGGTCAGTCGGTCCTGAG
AGATGGGCGAGCGCCGTTCTGAAGGGACGGGCGATGGCCTCCGTTGCCCTC
>NC_000021.9:8216637-8216843
GTAAATCTCGCGCCGGGCCGTACCCATATCCGCAGCAGGTCTCCAAGGTGAACAGCCTCTGGCATG
TTGGAACAATGTAGGTAAGGGAAGTCGGCAAGCCGGATCCGTAACTTCGGGATAAGGATTGGCTCT
AAGGGCTGGGTCGGTCGGGCTGGGGCGCGAAGCGGGGCTGGGCGCGCGCCGCGGCTGGACGAG
GCGCCGCCGCC
>NC_000021.9:8217426-8217861
GGGCCGCGGTTCCGCGCGGCGCCTCGCCTCGGCCGGCGCCTAGCAGCCGACTTAGAACTGGTGC
GGACCAGGGGAATCCGACTGTTTAATTAAAACAAAGCATCGCGAAGGCCCGCGGCGGGTGTTGACG
CGATGTGATTTCTGCCCAGTGCTCTGAATGTCAAAGTGAAGAAATTCAATGAAGCGCGGGTAAACGG
CGGGAGTAACTATGACTCTCTTAAGGTAGCCAAATGCCTCGTCATCTAATTAGTGACGCGCATGAAT
GGATGAACGAGATTCCCACTGTCCCTACCTACTATCCAGCGAAACCACAGCCAAGGGAACGGGCTT
GGCGGAATCAGCGGGGAAAGAAGACCCTGTTGAGCTTGACTCTAGTCTGGCACGGTGAAGAGACAT
GAGAGGTGTAGAATAAGTGGGAGGCCCCCGGCGCCCCCC
>NC_000021.9:8218016-8218612

CGGCCGGGCGCGACCCGCTCCGGGGACAGTGCCAGGTGGGGAGTTTGACTGGGGCGGTACACCT
GTCAAACGGTAACGCAGGTGTCCTAAGGCGAGCTCAGGGAGGACAGAAACCTCCCGTGGAGCAGA
AGGGCAAAAGCTCGCTTGATCTTGATTTTCAGTACGAATACAGACCGTGAAAGCGGGGCCTCACGAT
CCTTCTGACCTTTTGGGTTTTAAGCAGGAGGTGTCAGAAAAGTTACCACAGGGATAACTGGCTTGTG
GCGGCCAAGCGTTCATAGCGACGTCGCTTTTTGATCCTTCGATGTCGGCTCTTCCTATCATTGTGAA
GCAGAATTCACCAAGCGTTGGATTGTTCACCCACTAATAGGGAACGTGAGCTGGGTTTAGACCGTCG
TGAGACAGGTTAGTTTTACCCTACTGATGATGTGTTGTTGCCATGGTAATCCTGCTCAGTACGAGAG
GAACCGCAGGTTCAGACATTTGGTGTATGTGCTTGGCTGAGGAGCCAATGGGGCGAAGCTACCATC
TGTGGGATTATGACTGAACGCCTCTAAGTCAGAATCCCGCCCAGGCGGAACGATACGGCAGCGCCG
>NC_000021.9:8218833-8218934
GCTAAACCATTCGTAGACGACCTGCTTCTGGGTCGGGGTTTCGTACGTAGCAGAGCAGCTCCCTCG
CTGCGATCTATTGAAAGTCAGCCCTCGACACAAGG

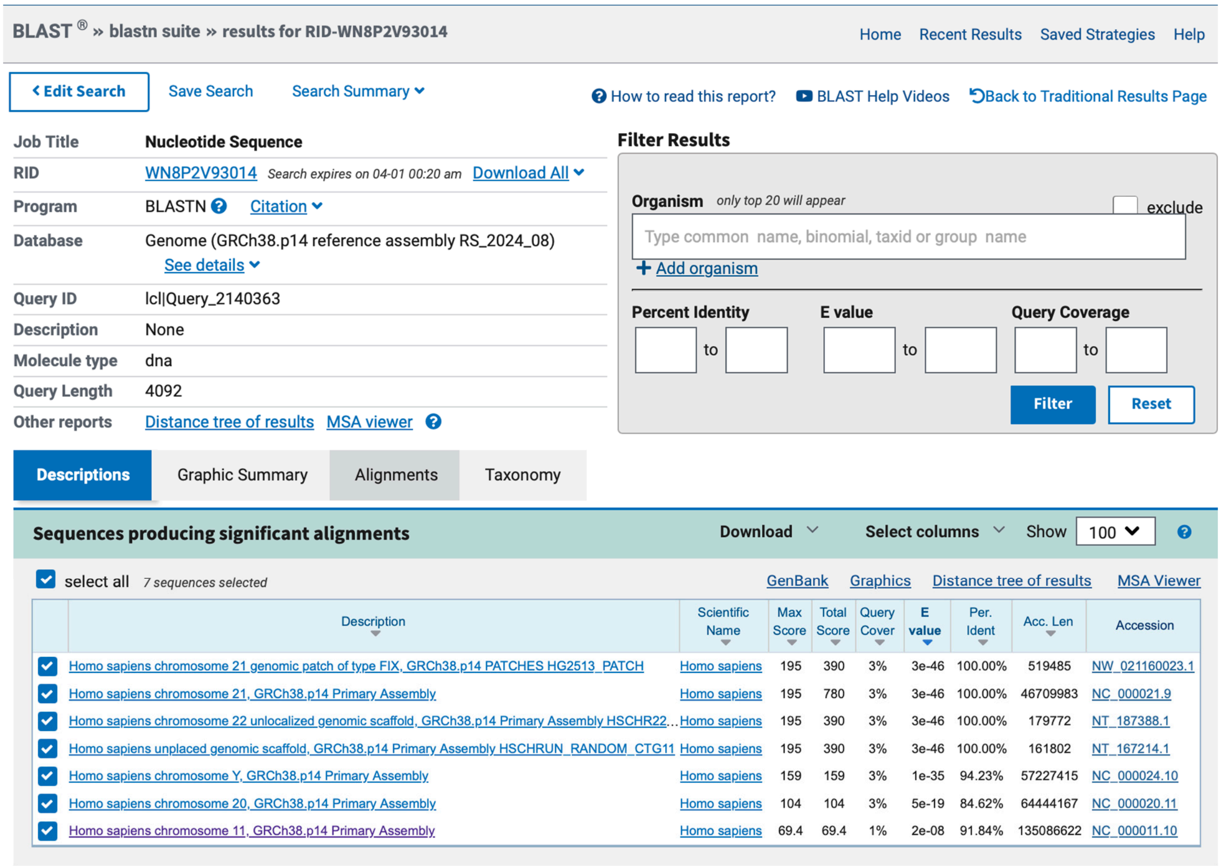
BLAST ® » blastn suite » results for RID-WN8P2V93014

Home Recent Results Saved Strategies Help

Edit Search Save Search Search Summary

How to read this report? BLAST Help Videos Back to Traditional Results Page

Job Title	Nucleotide Sequence
RID	WN8P2V93014 Search expires on 04-01 00:20 am Download All
Program	BLASTN Citation
Database	Genome (GRCh38.p14 reference assembly RS_2024_08) See details
Query ID	lcl|Query_2140363
Description	None
Molecule type	dna
Query Length	4092
Other reports	Distance tree of results MSA viewer

Filter Results

Organism only top 20 will appear exclude

Type common name, binomial, taxid or group name

Add organism

Percent Identity to E value to Query Coverage to

Filter Reset

Descriptions Graphic Summary Alignments Taxonomy

Sequences producing significant alignments Download Select columns Show 100

select all 7 sequences selected GenBank Graphics Distance tree of results MSA Viewer

Description	Scientific Name	Max Score	Total Score	Query Cover	E value	Per. Ident	Acc. Len	Accession
Homo sapiens chromosome 21 genomic patch of type FIX, GRCh38.p14 PATCHES HG2513_PATCH	Homo sapiens	195	390	3%	3e-46	100.00%	519485	NW_021160023.1
Homo sapiens chromosome 21, GRCh38.p14 Primary Assembly	Homo sapiens	195	780	3%	3e-46	100.00%	46709983	NC_000021.9
Homo sapiens chromosome 22 unlocalized genomic scaffold, GRCh38.p14 Primary Assembly HSCHR22...	Homo sapiens	195	390	3%	3e-46	100.00%	179772	NT_187388.1
Homo sapiens unplaced genomic scaffold, GRCh38.p14 Primary Assembly HSCHRUN_RANDOM_CTG11	Homo sapiens	195	390	3%	3e-46	100.00%	161802	NT_167214.1
Homo sapiens chromosome Y, GRCh38.p14 Primary Assembly	Homo sapiens	159	159	3%	1e-35	94.23%	57227415	NC_000024.10
Homo sapiens chromosome 20, GRCh38.p14 Primary Assembly	Homo sapiens	104	104	3%	5e-19	84.62%	64444167	NC_000020.11
Homo sapiens chromosome 11, GRCh38.p14 Primary Assembly	Homo sapiens	69.4	69.4	1%	2e-08	91.84%	135086622	NC_000011.10

PROBLEM WITH THIS UCE cluster 52. It matches different locations on chr 21. I do not see cluster on the Graph

chr21 CLUSTER 53 positions on BED file: 12226 - 12236
>NC_000021.9:8392738-8392850
GGTACAGTGAAACTGCGAATGGCTCATTAAATCAGTTATGGTTCCTTTGGTCGCTCGCTCCTCTCCTA
CTTGGATAACTGTGGTAATTCTAGAGCTAATACATGCCGACGGG
>NC_000021.9:8393044-8394537
GCCGTGCCTACCATGGTGACCACGGGTGACGGGGAATCAGGGTTCGATTCCGGAGAGGGAGCCTG
AGAAACGGCTACCACATCCAAGGAAGGCAGCAGGCGCGCAAATTACCCACTCCCGACCCGGGGAG

GTAGTGACGAAAAATAACAATACAGGACTCTTTCGAGGCCCTGTAATTGGAATGAGTCCACTTTAAAT
CCTTTAACGAGGATCCATTGGAGGGCAAGTCTGGTGCCAGCAGCCGCGGTAATTCCAGCTCCAATA
GCGTATATTAAAGTTGCTGCAGTTAAAAAGCTCGTAGTTGGATCTTGGGAGCGGGCGGGCGGTCCG
CCGCGAGGCGAGCCACCGCCCGTCCCCGCCCCTTGCCTCTCGGCGCCCCCTCGATGCTCTTAGCT
GAGTGTCCCGCGGGGCCCGAAGCgtttactttgaaaaaattagaGTGTTCAAAGCAGGCCCGAGCCGCCTGG
ATACCGCAGCTAGGAATAATGGAATAGGACCGCGGTTCTATTTTGTTGGTTTTCGGAACTGAGGCCA
TGATTAAGAGGGACGGCCGGGGGCATTCGTATTGCGCCGCTAGAGGTGAAATTCTTGGACCGGCGC
AAGACGGACCAGAGCGAAAGCATTTGCCAAGAATGTTTTCATTAATCAAGAACGAAAGTCGGAGGTT
CGAAGACGATCAGATACCGTCGTAGTTCCGACCATAAACGATGCCGACCGGCGATGCGGCGGCGTT
ATTCCCATGACCCGCCGGGCAGCTTCCGGGAAACCAAAGTCTTTGGGTTCCGGGGGGAGTATGGTT
GCAAAGCTGAAACTTAAAGGAATTGACGGAAGGGCACCACCAGGAGTGGAGCCTGCGGCTTAATTT
GACTCAACACGGGAAACCTCACCCGGCCCGGACACGGACAGGATTGACAGATTGATAGCTCTTTCT
CGATTCCgtgggtggtggtgcatggcCGTTCTTAGTTGGTGGAGCGATTTGTCTGGTTAATTCCGATAACGAA
CGAGACTCTGGCATGCTAACTAGTTACGCGACCCCCGAGCGGTCGGCGTCCCCCAACTTCTTAGAG
GGACAAGTGGCGTTCAGCCACCCGAGATTGAGCAATAACAGGTCTGTGATGCCCTTAGATGTCCGG
GGCTGCACGCGCGCTACACTGACTGGCTCAGCGTGTGCCTACCCTACGCCGGCAGGCGCGGGTAA
CCCGTTGAACCCCATTCGTGATGGGGATCGGGGATTGCAATTATTCCCCATGAACGAGGAATTCCCA
GTAAGTGCGGGTCATAAGCTTGCGTTGATTAAGTCCCTGCCCTTTGTACACACCGCCCGTCGCTACT
ACCGATTGGATGGTTTAGTGAGGCCCTCGGATCGGCCCCGCCGGGGTCGGCCCACGGCCCTGGCG
GAGCGCTGAGAAGACGGTCGAACTTGACTATCTAGAGGAAGTAAAAGTCGTAACAAGGTTTCCGTAG
GTGAACCTGCGGAAGGATCATTAACG
>NC_000021.9:8395603-8395705
TACGACTCTTAGCGGTGGATCACTCGGCTCGTGCGTCGATGAAGAACGCAGCTAGCTGCGAGAATT
AATGTGAATTGCAGGACACATTGATCATCGACACTT
>NC_000021.9:8397189-8397359
CCGGGTCTTCCCGGAGTCGGGTTGCTTGGGAATGCAGCCCAAAGCGGGTGGTAAACTCCATCTAAG
GCTAAATACCGGCACGAGACCGATAGTCAACAAGTACCGTAAGGGAAAGTTGAAAAGAACTTTGAAG
AGAGAGTTCAAGAGGGCGTGAAACCGTTAAGAGGTAA
>NC_000021.9:8398353-8398617
TCTCCAGTCCGCCGAGGGCGCACCACCGGCCCGTCTCGCCCGCCGCGCCGGGGAGGTGGAGCAC
GAGCGCACGTGTTAGGACCCGAAAGATGGTGAACTATGCCTGGGCAGGGCGAAGCCAGAGGAAAC
TCTGGTGGAGGTCCGTAGCGGTCCTGACGTGCAAATCGGTCGTCCGACCTGGGTATAGGGGCGAA
AGACTAATCGAACCATCTAGTAGCTGGTTCCCTCCGAAGTTTCCCTCAGGATAGCTGGCGCTCTCGC
AGA
>NC_000021.9:8398633-8398999
CACGCAGTTTTATCCGGTAAAGCGAATGATTAGAGGTCTTGGGGCCGAAACGATCTCAACCTATTCT
CAAACTTTAAATGGGTAAGAAGCCCGGCTCGCTGGCGTGGAGCCGGGCGTGGAATGCGAGTGCCT
AGTGGGCCACTTTTGGTAAGCAGAACTGGCGCTGCGGGATGAACCGAACGCCGGGTTAAGGCGCC
CGATGCCGACGCTCATCAGACCCCAGAAAAGGTGTTGGTTGATATAGACAGCAGGACGGTGGCCAT
GGAAGTCGGAATCCGCTAAGGAGTGTGTAACAACTCACCTGCCGAATCAACTAGCCCTGAAAATGGA
TGGCGCTGGAGCGTCGGGCCCATACCCGGCCGTCGC
>NC_000021.9:8399164-8399411
CCCCGCGGACGCTACGCCGCGACGAGTAGGAGGGCCGCTGCGGTGAGCCTTGAAGCCTAGGGCG
CGGGCCCGGGTGGAGCCGCCGCAGGTGCAGATCTTGGTGGTAGTAGCAAATATTCAAACGAGAACT
TTGAAGGCCGAAGTGGAGAAGGGTTCCATGTGAACAGCAGTTGAACATGGGTCAGTCGGTCCTGAG
AGATGGGCGAGCGCCGTTCCGAAGGGACGGGCGATGGCCTCCGTTGCCCTC
>NC_000021.9:8399671-8399877
GTAAATCTCGCGCCGGGCCGTACCCATATCCGCAGCAGGTCTCCAAGGTGAACAGCCTCTGGCATG
TTGGAACAATGTAGGTAAGGGAAGTCGGCAAGCCGGATCCGTAACTTCGGGATAAGGATTGGCTCT

AAGGGCTGGGTCGGTCGGGCTGGGGCGCGAAGCGGGGCTGGGCGCGCGCCGCGGCTGGACGAG
GCGCCGCCGCC
>NC_000021.9:8400464-8400899
GGGCCGCGGTTCCGCGCGGCGCCTCGCCTCGGCCGGCGCCTAGCAGCCGACTTAGAACTGGTGC
GGACCAGGGGAATCCGACTGTTTAATTAAAACAAAGCATCGCGAAGGCCCGCGGCGGGTGTTGACG
CGATGTGATTTCTGCCCAGTGCTCTGAATGTCAAAGTGAAGAAATTCAATGAAGCGCGGGTAAACGG
CGGGAGTAACTATGACTCTCTTAAGGTAGCCAAATGCCTCGTCATCTAATTAGTGACGCGCATGAAT
GGATGAACGAGATTCCCACTGTCCCTACCTACTATCCAGCGAAACCACAGCCAAGGGAACGGGCTT
GGCGGAATCAGCGGGGAAAGAAGACCCTGTTGAGCTTGACTCTAGTCTGGCACGGTGAAGAGACAT
GAGAGGTGTAGAATAAGTGGGAGGCCCCCGGCGCCCCCC
>NC_000021.9:8401054-8401650
CGGCCGGGCGCGACCCGCTCCGGGGACAGTGCCAGGTGGGGAGTTTGACTGGGGCGGTACACCT
GTCAAACGGTAACGCAGGTGTCCTAAGGCGAGCTCAGGGAGGACAGAAACCTCCCGTGGAGCAGA
AGGGCAAAAGCTCGCTTGATCTTGATTTTCAGTACGAATACAGACCGTGAAAGCGGGGCCTCACGAT
CCTTCTGACCTTTTGGGTTTTAAGCAGGAGGTGTCAGAAAAGTTACCACAGGGATAACTGGCTTGTG
GCGGCCAAGCGTTCATAGCGACGTCGCTTTTTGATCCTTCGATGTCGGCTCTTCCTATCATTGTGAA
GCAGAATTCACCAAGCGTTGGATTGTTCACCCACTAATAGGGAACGTGAGCTGGGTTTAGACCGTCG
TGAGACAGGTTAGTTTTACCCTACTGATGATGTGTTGTTGCCATGGTAATCCTGCTCAGTACGAGAG
GAACCGCAGGTTCAGACATTTGGTGTATGTGCTTGGCTGAGGAGCCAATGGGGCGAAGCTACCATC
TGTGGGATTATGACTGAACGCCTCTAAGTCAGAATCCCGCCCAGGCGGAACGATACGGCAGCGCCG
>NC_000021.9:8401872-8401973
GCTAAACCATTCGTAGACGACCTGCTTCTGGGTCGGGGTTTCGTACGTAGCAGAGCAGCTCCCTCG
CTGCGATCTATTGAAAGTCAGCCCTCGACACAAGG

The same PROBLEM WITH THIS UCE as in cluster 52. It matches different locations on chr 21. I do not see cluster on the Graph

BLAST ® » blastn suite » results for RID-WN91AWV7014

Home Recent Results Saved Strategies Help

Edit Search Save Search Search Summary

How to read this report? BLAST Help Videos Back to Traditional Results Page

Job Title	Nucleotide Sequence
RID	WN91AWV7014 *Search expires on 04-01 00:26 am* Download All
Program	BLASTN Citation
Database	Genome (GRCh38.p14 reference assembly RS_2024_08) See details
Query ID	lcl|Query_2488963
Description	None
Molecule type	dna
Query Length	4092
Other reports	Distance tree of results MSA viewer

Filter Results

Organism *only top 20 will appear* exclude

Type common name, binomial, taxid or group name

Add organism

Percent Identity to **E value** to **Query Coverage** to

Filter Reset

Descriptions Graphic Summary Alignments Taxonomy

Sequences producing significant alignments Download Select columns Show 100

select all *7 sequences selected* GenBank Graphics Distance tree of results MSA Viewer

Description	Scientific Name	Max Score	Total Score	Query Cover	E value	Per. Ident	Acc. Len	Accession
Homo sapiens chromosome 21 genomic patch of type FIX, GRCh38.p14 PATCHES HG2513_PATCH	Homo sapiens	195	390	3%	3e-46	100.00%	519485	NW_021160023.1
Homo sapiens chromosome 21, GRCh38.p14 Primary Assembly	Homo sapiens	195	780	3%	3e-46	100.00%	46709983	NC_000021.9
Homo sapiens chromosome 22 unlocalized genomic scaffold, GRCh38.p14 Primary Assembly HSCHR22...	Homo sapiens	195	390	3%	3e-46	100.00%	179772	NT_187388.1
Homo sapiens unplaced genomic scaffold, GRCh38.p14 Primary Assembly HSCHRUN_RANDOM_CTG11	Homo sapiens	195	390	3%	3e-46	100.00%	161802	NT_167214.1
Homo sapiens chromosome Y, GRCh38.p14 Primary Assembly	Homo sapiens	159	159	3%	1e-35	94.23%	57227415	NC_000024.10
Homo sapiens chromosome 20, GRCh38.p14 Primary Assembly	Homo sapiens	104	104	3%	5e-19	84.62%	64444167	NC_000020.11
Homo sapiens chromosome 11, GRCh38.p14 Primary Assembly	Homo sapiens	69.4	69.4	1%	2e-08	91.84%	135086622	NC_000011.10

chr21 CLUSTER 54 positions on BED file: 12237 - 12247
>NC_000021.9:8436948-8437060
GGTACAGTGAAACTGCGAATGGCTCATTAAATCAGTTATGGTTCCTTTGGTCGCTCGCTCCTCTCCTA
CTTGGATAACTGTGGTAATTCTAGAGCTAATACATGCCGACGGG
>NC_000021.9:8437254-8438747
GCCGTGCCTACCATGGTGACCACGGGTGACGGGGAATCAGGGTTCGATTCCGGAGAGGGAGCCTG
AGAAACGGCTACCACATCCAAGGAAGGCAGCAGGCGCGCAAATTACCCACTCCCGACCCGGGGAG
GTAGTGACGAAAAATAACAATACAGGACTCTTTCGAGGCCCTGTAATTGGAATGAGTCCACTTTAAAT
CCTTTAACGAGGATCCATTGGAGGGCAAGTCTGGTGCCAGCAGCCGCGGTAATTCCAGCTCCAATA
GCGTATATTAAAGTTGCTGCAGTTAAAAAGCTCGTAGTTGGATCTTGGGAGCGGGCGGGCGGTCCG
CCGCGAGGCGAGCCACCGCCCGTCCCCGCCCCTTGCCTCTCGGCGCCCCCTCGATGCTCTTAGCT
GAGTGTCCCGCGGGGCCCGAAGCgtttactttgaaaaaattagaGTGTTCAAAGCAGGCCCGAGCCGCCTGG
ATACCGCAGCTAGGAATAATGGAATAGGACCGCGGTTCTATTTTGTTGGTTTTCGGAACTGAGGCCA
TGATTAAGAGGGACGGCCGGGGGCATTCGTATTGCGCCGCTAGAGGTGAAATTCTTGGACCGGCGC
AAGACGGACCAGAGCGAAAGCATTTGCCAAGAATGTTTTCATTAATCAAGAACGAAAGTCGGAGGTT
CGAAGACGATCAGATACCGTCGTAGTTCCGACCATAAACGATGCCGACCGGCGATGCGGCGGCGTT
ATTCCCATGACCCGCCGGGCAGCTTCCGGGAAACCAAAGTCTTTGGGTTCCGGGGGGAGTATGGTT
GCAAAGCTGAAACTTAAAGGAATTGACGGAAGGGCACCACCAGGAGTGGAGCCTGCGGCTTAATTT
GACTCAACACGGGAAACCTCACCCGGCCCGGACACGGACAGGATTGACAGATTGATAGCTCTTTCT
CGATTCCgtgggtggtggtgcatggcCGTTCTTAGTTGGTGGAGCGATTTGTCTGGTTAATTCCGATAACGAA
CGAGACTCTGGCATGCTAACTAGTTACGCGACCCCCGAGCGGTCGGCGTCCCCCAACTTCTTAGAG
GGACAAGTGGCGTTCAGCCACCCGAGATTGAGCAATAACAGGTCTGTGATGCCCTTAGATGTCCGG

GGCTGCACGCGCGCTACACTGACTGGCTCAGCGTGTGCCTACCCTACGCCGGCAGGCGCGGGTAA
CCCGTTGAACCCCATTCGTGATGGGGATCGGGGATTGCAATTATTCCCCATGAACGAGGAATTCCCA
GTAAGTGCGGGTCATAAGCTTGCGTTGATTAAGTCCCTGCCCTTTGTACACACCGCCCGTCGCTACT
ACCGATTGGATGGTTTAGTGAGGCCCTCGGATCGGCCCCGCCGGGGTCGGCCCACGGCCCTGGCG
GAGCGCTGAGAAGACGGTCGAACTTGACTATCTAGAGGAAGTAAAAGTCGTAACAAGGTTTCCGTAG
GTGAACCTGCGGAAGGATCATTAACG
>NC_000021.9:8439819-8439921
TACGACTCTTAGCGGTGGATCACTCGGCTCGTGCGTCGATGAAGAACGCAGCTAGCTGCGAGAATT
AATGTGAATTGCAGGACACATTGATCATCGACACTT
>NC_000021.9:8441409-8441579
CCGGGTCTTCCCGGAGTCGGGTTGCTTGGGAATGCAGCCCAAAGCGGGTGGTAAACTCCATCTAAG
GCTAAATACCGGCACGAGACCGATAGTCAACAAGTACCGTAAGGGAAAGTTGAAAAGAACTTTGAAG
AGAGAGTTCAAGAGGGCGTGAAACCGTTAAGAGGTAA
>NC_000021.9:8442582-8442846
TCTCCAGTCCGCCGAGGGCGCACCACCGGCCCGTCTCGCCCGCCGCGCCGGGGAGGTGGAGCAC
GAGCGCACGTGTTAGGACCCGAAAGATGGTGAACTATGCCTGGGCAGGGCGAAGCCAGAGGAAAC
TCTGGTGGAGGTCCGTAGCGGTCCTGACGTGCAAATCGGTCGTCCGACCTGGGTATAGGGGCGAA
AGACTAATCGAACCATCTAGTAGCTGGTTCCCTCCGAAGTTTCCCTCAGGATAGCTGGCGCTCTCGC
AGA
>NC_000021.9:8442862-8443228
CACGCAGTTTTATCCGGTAAAGCGAATGATTAGAGGTCTTGGGGCCGAAACGATCTCAACCTATTCT
CAAACTTTAAATGGGTAAGAAGCCCGGCTCGCTGGCGTGGAGCCGGGCGTGGAATGCGAGTGCCT
AGTGGGCCACTTTTGGTAAGCAGAACTGGCGCTGCGGGATGAACCGAACGCCGGGTTAAGGCGCC
CGATGCCGACGCTCATCAGACCCCAGAAAAGGTGTTGGTTGATATAGACAGCAGGACGGTGGCCAT
GGAAGTCGGAATCCGCTAAGGAGTGTGTAACAACTCACCTGCCGAATCAACTAGCCCTGAAAATGGA
TGGCGCTGGAGCGTCGGGCCCATACCCGGCCGTCGC
>NC_000021.9:8443399-8443646
CCCCGCGGACGCTACGCCGCGACGAGTAGGAGGGCCGCTGCGGTGAGCCTTGAAGCCTAGGGCG
CGGGCCCGGGTGGAGCCGCCGCAGGTGCAGATCTTGGTGGTAGTAGCAAATATTCAAACGAGAACT
TTGAAGGCCGAAGTGGAGAAGGGTTCCATGTGAACAGCAGTTGAACATGGGTCAGTCGGTCCTGAG
AGATGGGCGAGCGCCGTTCCGAAGGGACGGGCGATGGCCTCCGTTGCCCTC
>NC_000021.9:8443906-8444112
GTAAATCTCGCGCCGGGCCGTACCCATATCCGCAGCAGGTCTCCAAGGTGAACAGCCTCTGGCATG
TTGGAACAATGTAGGTAAGGGAAGTCGGCAAGCCGGATCCGTAACTTCGGGATAAGGATTGGCTCT
AAGGGCTGGGTCGGTCGGGCTGGGGCGCGAAGCGGGGCTGGGCGCGCGCCGCGGCTGGACGAG
GCGCCGCCGCC
>NC_000021.9:8444696-8445131
GGGCCGCGGTTCCGCGCGGCGCCTCGCCTCGGCCGGCGCCTAGCAGCCGACTTAGAACTGGTGC
GGACCAGGGGAATCCGACTGTTTAATTAAAACAAAGCATCGCGAAGGCCCGCGGCGGGTGTTGACG
CGATGTGATTTCTGCCCAGTGCTCTGAATGTCAAAGTGAAGAAATTCAATGAAGCGCGGGTAAACGG
CGGGAGTAACTATGACTCTCTTAAGGTAGCCAAATGCCTCGTCATCTAATTAGTGACGCGCATGAAT
GGATGAACGAGATTCCCACTGTCCCTACCTACTATCCAGCGAAACCACAGCCAAGGGAACGGGCTT
GGCGGAATCAGCGGGGAAAGAAGACCCTGTTGAGCTTGACTCTAGTCTGGCACGGTGAAGAGACAT
GAGAGGTGTAGAATAAGTGGGAGGCCCCCGGCGCCCCCC
>NC_000021.9:8445286-8445882
CGGCCGGGCGCGACCCGCTCCGGGGACAGTGCCAGGTGGGGAGTTTGACTGGGGCGGTACACCT
GTCAAACGGTAACGCAGGTGTCCTAAGGCGAGCTCAGGGAGGACAGAAACCTCCCGTGGAGCAGA
AGGGCAAAAGCTCGCTTGATCTTGATTTTCAGTACGAATACAGACCGTGAAAGCGGGGCCTCACGAT
CCTTCTGACCTTTTGGGTTTTAAGCAGGAGGTGTCAGAAAAGTTACCACAGGGATAACTGGCTTGTG

GCGGCCAAGCGTTCATAGCGACGTCGCTTTTTGATCCTTCGATGTCGGCTCTTCCTATCATTGTGAA
GCAGAATTCACCAAGCGTTGGATTGTTCACCCACTAATAGGGAACGTGAGCTGGGTTTAGACCGTCG
TGAGACAGGTTAGTTTTACCCTACTGATGATGTGTTGTTGCCATGGTAATCCTGCTCAGTACGAGAG
GAACCGCAGGTTCAGACATTTGGTGTATGTGCTTGGCTGAGGAGCCAATGGGGCGAAGCTACCATC
TGTGGGATTATGACTGAACGCCTCTAAGTCAGAATCCCGCCCAGGCGGAACGATACGGCAGCGCCG
>NC_000021.9:8446103-8446204
GCTAAACCATTCGTAGACGACCTGCTTCTGGGTCGGGGTTTCGTACGTAGCAGAGCAGCTCCCTCG
CTGCGATCTATTGAAAGTCAGCCCTCGACACAAGG
The same PROBLEM WITH THIS UCE as in cluster 52. It matches different locations on chr 21. I do not see cluster on the Graph

chrX CLUSTER 55 positions on BED file: 12466 - 12478
>NC_000023.11:24980469-24980669
AAGGAGAGggatgtttccttctttgtgtaaTGTGCAGTAGTTACAATAAACACAAGATTTCCAAATCTCAGAGGAC
TCTTCTTGACAGTTGCTTGATAACAGCTTTTTTCATTCCATAGTTTTGACAGTTTGTGTGTGTTGCCAT
ATCTAGTGTAAACTAAAACATCACtgtcattctgtttttcttttgtatgtggctt
>NC_000023.11:24981497-24981613
AGCTATTGTTAAACACTACAAGCTAGCTGacagtaaaattataaatacaggTGTTGTCACACTACACTATAATca
gaattttaatttgaattttcatttgtaaagcctttttttca
>NC_000023.11:24981615-24981806
tgaaaatgGGAATAGAATTAGTTTTATTGACAgtaaaaagacattttgaaagCTTCCTAAATCATTACCTGTATTTA
GCAGGAGGAAAAATTGTATATGTCCATGTAAAAAGAAGAGTTTTATTATCACATATTTACTCATTATAT
TTGGTGGTAAATAAAACGGGGAAGACATGTTTGACAAATGATTGA
>NC_000023.11:24982072-24982250
tttttttccctcaaggcATTTAGTGTCGTGTCTGCCAGATTTCAGTGATGAGCAGCGAATTGCTTTTAAAGTGG
GCATTATGCCAGTTCAGCAGCACAATGGAAAACCAATCTTCCACCATTCTACTTTTGATGCTGTTATT
GTAGCATTTTAGATAACTgctgccacaaaaaaaaag
>NC_000023.11:24990192-24991169
aaaaaattaaatatccaaTATCAATGGATTCTAAATggttttgatatttctttttgtcctttccCATGAAGAgtaatttatttccctttttaaagt
GTGGGCAGAATGATTACTAGCGCACTGTAATGTAATTGTGTTgcattgataaaataaaaattgtcctTTATCTGTG
TCCTGATAATGTTCAATTTACAGGCTGGCTTCTCTGCCACCTCTTCAGTGCTTTGAGTATTCCAGttctcc
tcccttcctgctcTGAGAGCGCACAGAACTGTTTGAAATCCACTGGTACAATTGTCAAATCAATTATTCATTC
TCTGCAATTATGCTCGCACAAAGAACATTTTGCTGGTCTGAATGATGATTAAATTAACAGCTATTCCA
GCTGCCTGATAACATCTAATAGAATATTCATAAGCCCAAAATGGAATGAATTATCTCCATTAACTTCAT
CATGCTCACTTAATTACATGCTTGTTATTGTATTTACACCTTGTTAGATACCGCTGAAGCTGATCCAGT
GGCTGGCCGGGAATTGGAAGCGTCTGTCATGGGGCAGTTGGAGCGCGTTTTGTAggaaatgctatttatttta
aatgctccACCTGCTGGGAGCCGAGGTTAGTCAGCAGCACTGAGATGAATTGGGAAACGGGgtgtaaaaag
aaataatgtgcTTCTGACAGGCTCCGTGGCTTTTAAGTTGCTCTCATTCAGCCACttcacaaaaaattattttattccatc
tCTCAGTGATGATGACATGATTGCTTTTTGGTAATCATTTaccattctgattttatttttgaagtaaatTGTCTGAAGTA
ATAGGTTCTTGGAATTACAGCGTGCCTTGCTTTTTTCTTAGAACTTTATTTAAGCTTGTCTTCCAGCAT
TTAACCCGAGTCCCCTCTTTCGTTTGATCTTCTAACTTTATTTATACAACAGTGCTTAATGATCCTGCac
aatgtgtttc
>NC_000023.11:24992342-24992583
TGGTGGGAAGCCATGCAATTAAAAAACTGACTGAAATTATTACCTGATTTCATAATTGACAGAATATTT
AATTCAGATATAGTAAATTGACACACCTCTACACAGAGACAATTATGACTGACTGTATAACACCTGAC
AGAATCAATTATAACTGACTGTTGTTTGATACCTTtgtacagaaataaatgaaacagacatTCAGAACAGAGACG

GCTGCTGCCTGTGTGCCTAAGCCTgcctt
>NC_000023.11:24999404-25000237
GTGGAGCTTCCTTATAGCAGTTTAATAGATTAACTCATCTCAGCCAGCCTCAAATTTCAGATTAAAATT
CCTTCATCCCGAAGGCAGCATTATCAGGTTGTTGGAGGCTGTTTTCAAACCTTGGTTTTGAATAGCAG
CGGCTCTGCATTAATTTAACCACTAAGCTAATAAGTAGGTTTCGTTTTGTTATGCTAAgctttattgcttttcttttg
atCAGAATGGTGTTGTTGAGCAAAGCAGCAGACAAGGCATTCTTTGATGAGGGAATTAGCGCTCCATT
CTTCCTCTATTATTCATAGCGCAGTGGAATATGTAAGTACCTGAGGGTGTCAAAGGAGCGCAGGTTC
TATTGCAAAGTGCTCGCCTTGTTTCTCTCAATAGCTGACTATGAGATGATAAACCGCTTAATACACTG
TGCTAATTGGATGAGAGCAAAAAGAGATGGGAATTAAGGCGAGGCAAAAGGAGAAAGTGCAACCAG
CCTTCAATTCACTCTTTCACCACTTTAACAGCACCAAAGGAGGCACAAGCTTTCTATAAGCAGACTAG
AGGTTTTGTGCAGAGATAAAATGAACGTGTTAGTGGATTCAAGTAATACACTAATTATTGCACAGTAT
AAATACCACTACTGGTTTTATAACAGGGAGGTAAACTTCTTTTGAGAGGGTTATAGGATTGCTTGGCA
GTGGTGTAGAACCTAATAAGGGCCCAGAGTAATAACCCCCTGGATTAACAAAATTGCTGCTTGTAGA
AGTATGATTCAGGCTTTTACGAAGATTTCCggagaatgaataaaaatgactgAATGACATTTCTTAATGTATAAC
CGG
>NC_000023.11:25001277-25001629
GTTCCCAGATATTGGGGCCAAATTGGGTTGCCATTCATTAATAATGCTCACAATAAGATTAAATCATT
CTGGAAAATCTCATAAAATCCCCCTAAGTACGCTCCAGTGGCTCTTTCCAATCCCCATCattcagcacttgg
ggaggtttggaaggagaagaaaattgGTTTCTTTGCTTTTAATGCTGCTTTGAAAAGATACTCAGAGTTTTTCATG
CGTGACATGTCACATGTTTAATGTGGACTTGCTGGAAGTCAGGGGGTCTTTAGCGTGTGATATTTAT
GGGGAATATTAAGTGCAGAATGAGGTCCCTCCAGCTGGGGAAAGTTGAAAGAATAGGAGGCCCAGG
ACT
>NC_000023.11:25001642-25001843
GCATCAGAATGACATAGGATTAAGAGTTTGTAAAACAAGGCGAGGGGTTCTCATTGGCCAGTAATAA
ATGTAATCCTTGGAGGTAATTTCACGCAATTTGCTTCTGTTCCATGGAAGggtcaaatgagaaaaaatgacttaa
aaaagaGTTCATTAACAGGAAGAATGGTACTGTTTCTCGTATACCATCCAACTAGCAGC
>NC_000023.11:25003824-25003988
TCTACAGTTAATAAGTTAAATAAAGGGCTTGAGTACATAAATATTTGTCTAGGAACCCTACCGTATCTA
CAACACAGTAAGAATCTACGGGAGGACAAACTTTTACCATACCACGCTGAGTGCAATTGCGTTATAA
CATTTCAAATATACaaagctctgttctt
>NC_000023.11:25003991-25004130
tttttttttaataacaatggTATGTACAGAATCAATAATCACAGTTTGGTGACTTCAACAGTCCATATTCTAGCAG
AGTATTATTTCCCTTTGGTTTGATATAAAAACCTTGGTTGGTGTATGATAAAGAAGAGAAC
>NC_000023.11:25009373-25009552
GAGTTGCTGTCACATCTCTGTTTGACAATGGAGAAATGTGTCAACAGAAAGGAGGGGACAGGCCTCA
TCATTAGCCCTCTAATGCAAGAAGCTGTTGTGTATTAAATGAGCCATATGGAATTTATTGTGCTTTATC
AGCGCTTGATTTTGACTGTAAAGTGATTCACTCAGCTCCTAAC
>NC_000023.11:25009620-25009928
CTGGGTTCGGAAATGCACTCTGGTATGGGGCTGCAAACACCTTTAATAGCATTGCACTCACTCCCTA
TTAGATCAATGTGGGTTCATTGCTATAAAAAATGGGAAATCAAATAGCATTAGCCAGCTCCTTGTGCG
GGGCGGAGGGGAAATCAAACAGCATTTTGCCTTCAAATGGCCCTGTTTGTTCTAGCACTAAGTGGGC
TTTTATGAAGCCCGATTGGAGACTTAATCGCAGCCAAATACCTGCTTGGAACTGAGCGGATTTGTCTA
ACAACCAAATCCATGCTCCATCCCATTATTTCAATCAG

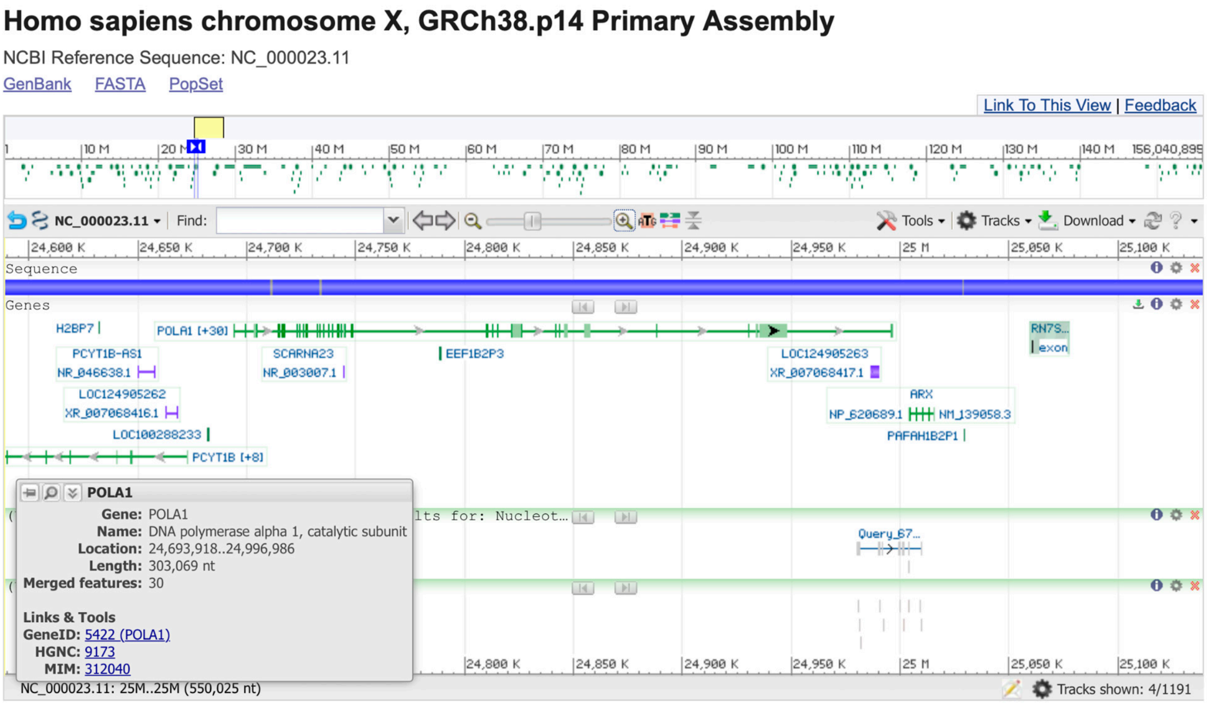
Homo sapiens chromosome X, GRCh38.p14 Primary Assembly
NCBI Reference Sequence: NC_000023.11
GenBank FASTA PopSet
Link To This View | Feedback
NC_000023.11
Find:
Tools
Tracks
Download
Sequence
Genes
H2BP7
POLA1 [+30]
PCYT1B-AS1
NR_046638.1
SCARNA23
NR_003007.1
EEF1B2P3
LOC124905263
XR_007068417.1
LOC124905262
XR_007068416.1
ARX
NP_620689.1
NM_139058.3
LOC100288233
PAFAH1B2P1
PCYT1B [+8]
RN7S...
exon
POLA1
Gene: POLA1
Name: DNA polymerase alpha 1, catalytic subunit
Location: 24,693,918..24,996,986
Length: 303,069 nt
Merged features: 30
Links & Tools
GeneID: 5422 (POLA1)
HGNC: 9173
MIM: 312040
Query_67...
NC_000023.11: 25M..25M (550,025 nt)
Tracks shown: 4/1191

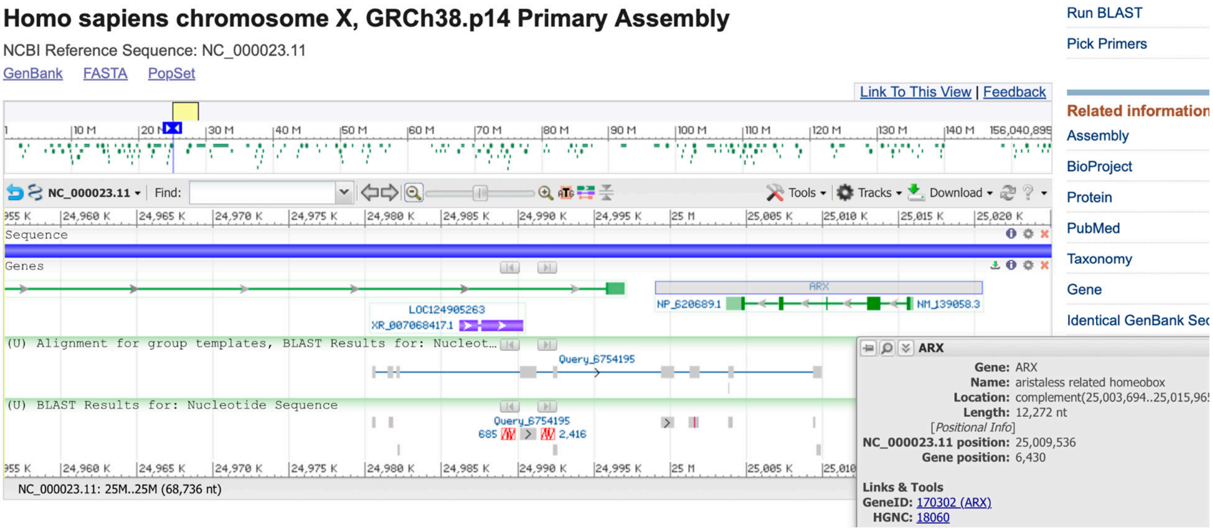
Homo sapiens chromosome X, GRCh38.p14 Primary Assembly
NCBI Reference Sequence: NC_000023.11
GenBank FASTA PopSet
Link To This View | Feedback
Run BLAST
Pick Primers
Related information
Assembly
BioProject
Protein
PubMed
Taxonomy
Gene
Identical GenBank Seq
NC_000023.11
Find:
Tools
Tracks
Download
Sequence
Genes
ARX
NP_620689.1
NM_139058.3
LOC124905263
XR_007068417.1
(U) Alignment for group templates, BLAST Results for: Nucleot...
Query_6754195
(U) BLAST Results for: Nucleotide Sequence
Query_6754195
685
2,416
ARX
Gene: ARX
Name: aristaless related homeobox
Location: complement(25,003,694..25,015,96
Length: 12,272 nt
[Positional Info]
NC_000023.11 position: 25,009,536
Gene position: 6,430
Links & Tools
GeneID: 170302 (ARX)
HGNC: 18060
NC_000023.11: 25M..25M (68,736 nt)
